# Molecular Catalysis of Energy Relevance in Metal–Organic Frameworks: From Higher Coordination Sphere to System Effects

**DOI:** 10.1021/acs.chemrev.2c00587

**Published:** 2023-05-15

**Authors:** Nina F. Suremann, Brian D. McCarthy, Wanja Gschwind, Amol Kumar, Ben A. Johnson, Leif Hammarström, Sascha Ott

**Affiliations:** † Department of Chemistry - Ångström Laboratory, Uppsala University, Box 523, 75120 Uppsala, Sweden; ‡ Technical University Munich (TUM), Campus Straubing for Biotechnology and Sustainability, Uferstraße 53, 94315 Straubing, Germany

## Abstract

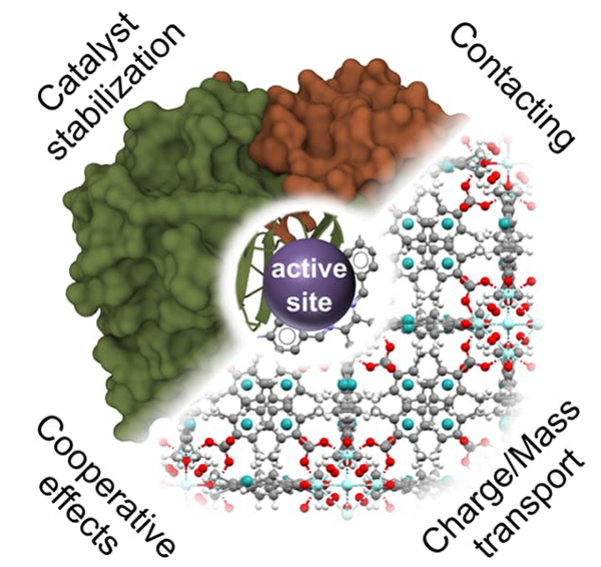

The modularity and synthetic flexibility of metal–organic frameworks (MOFs) have provoked analogies with enzymes, and even the term MOFzymes has been coined. In this review, we focus on molecular catalysis of energy relevance in MOFs, more specifically water oxidation, oxygen and carbon dioxide reduction, as well as hydrogen evolution in context of the MOF–enzyme analogy. Similar to enzymes, catalyst encapsulation in MOFs leads to structural stabilization under turnover conditions, while catalyst motifs that are synthetically out of reach in a homogeneous solution phase may be attainable as secondary building units in MOFs. Exploring the unique synthetic possibilities in MOFs, specific groups in the second and third coordination sphere around the catalytic active site have been incorporated to facilitate catalysis. A key difference between enzymes and MOFs is the fact that active site concentrations in the latter are often considerably higher, leading to charge and mass transport limitations in MOFs that are more severe than those in enzymes. High catalyst concentrations also put a limit on the distance between catalysts, and thus the available space for higher coordination sphere engineering. As transport is important for MOF-borne catalysis, a system perspective is chosen to highlight concepts that address the issue. A detailed section on transport and light-driven reactivity sets the stage for a concise review of the currently available literature on utilizing principles from Nature and system design for the preparation of catalytic MOF-based materials.

## 1 Introduction

The hydrogen and oxygen evolution reactions (HER and OER, respectively) are crucial for energy storage and at the heart of future energy systems, while the oxygen reduction reaction (ORR) is pivotal to fuel cell technologies. The carbon dioxide reduction reaction (CO_2_RR) takes a central role in the production of renewable, carbon-based fuels. Moreover, CO_2_ constitutes a C1 feedstock for value-added commodity chemicals that are essential starting materials for the chemical industry.^1,2^

**Figure 1:**
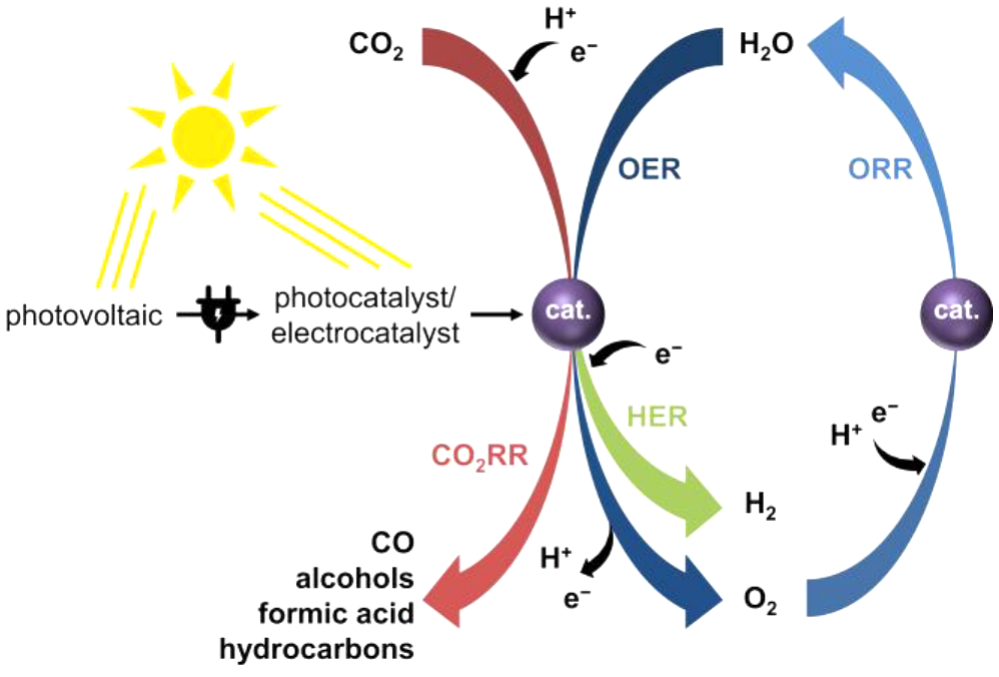
Schematic representation of reactions of energy relevance that are addressed in this review.

The above-mentioned reactions of energy relevance are energetically uphill redox processes and require an energy input either in the form of a chemical reagent, an electrochemical potential, or light (Figure 1) in order to proceed. While the latter energy input mimics principles of natural photosynthesis, and the products are often denoted as “solar fuels”,^3^ the former means to supply energy are more modular, and are not bound to seasonal variations or day/night cycles.

Irrespective of the kind of energy input, catalysts are required to lower overpotential requirements. Such catalysts are typically distinguished as being either heterogeneous or homogeneous, that is, in different phases or in the same phase. However, this type of differentiation has become increasingly blurred in recent years, and this review differentiates between catalysts which are atomically defined molecular species versus those that are not. The latter category includes, for example, metals or (mixed) metal oxides, alloys, nanoparticles, or other types of nanostructures. Such materials as catalysts of energy relevance impress with high technological readiness and durability, and have their rightful place in the ongoing transformation of our current energy system. At the same time, they fall somewhat short in options to fine-tune reactivity, in particular those of nonprecious metal catalytic sites. This is the stronghold of molecular catalysis, which offers almost limitless possibilities for synthetic modifications to fine-tune electronics and to control distances between specific atoms at the sub-Ångström length scale. In molecular systems, subtle modifications of not only the first but also the second and third coordination sphere around the catalytic metal center can be engineered. Controlling these parameters enables control over reaction outcomes, in particular if multiple products may be feasible at similar energy inputs. This is the case in the CO_2_RR, which can produce a variety of different products within a narrow potential window. This situation is further complicated by the fact that the HER is a thermodynamically competing reaction,^4^ putting additional requirements on the catalyst to favor one product over all the others.^5^

Catalysis of energy relevance is not only crucial for humankind but also in Nature. In fact, the oxygenic atmosphere that enables life on Earth as we know it is a direct result of photosynthetic water oxidation. Nature has evolved enzymes that can activate inert molecules such as water or CO_2_ using first row transition metals such as Mn, Fe, Co, or Ni in catalytic active sites. These metals are abundant in contrast to noble metals such as platinum or iridium that humankind often uses in this context. Through billions of years of evolution, Nature judiciously optimized environments beyond the first coordination sphere of the metal sites and developed enzymes that rival humankind’s noble metal-based catalysts in terms of overpotential requirements and activity.^6^

Inspired by Nature’s ingenuity, but also recognizing the necessity of immobilizing molecular catalysts to advance technological readiness, much research has concentrated on the incorporation of molecular catalysts into stabilizing support scaffolds.^7^ Ideally, such a support should not only structurally stabilize the catalytic site, but also offer synthetic flexibility to tune the chemical environment beyond the first coordination sphere. Examples of this strategy include supramolecular systems such as, among others, cages,^8,9^ polymers, dendrimers, or zeolites.^10−12^ Their preparation is often synthetically demanding, and, as in the case of zeolites, may be limited in terms of synthetic flexibility. The support is often conductive, resulting in molecularly functionalized electrodes^13^ and photoelectrodes. The alternative that is reviewed herein is based on the incorporation of molecularly defined catalytic function into metal–organic frameworks (MOFs), three-dimensional (3D) coordination polymers that consist of metal cations or clusters thereof, so-called secondary building units (SBUs), that are periodically interconnected by organic linker molecules.^14^ MOFs are highly porous and often exhibit high internal surface areas, both appealing features when designing highly active catalysts. They can also be electrically conducting, either by a hopping mechanism or band-like charge transport.^15^ In addition, their high degree of modularity combined with ample synthetic functionalization strategies using organic chemistry^16^ render MOFs an attractive platform for the systematic engineering of outer-sphere interactions.^17^

### 1.1 Principles of Enzyme Reactivity as Inspiration for MOF Design

Chemists have long been inspired by the beautiful orchestra that is enzymatic catalysis. Enzymes are the prime example of how outer coordination sphere effects can be leveraged to drive energy efficient and selective catalysis. Coordination spheres are classically divided into the first, second, and third coordination sphere, depending on the distance from the active site metal. The first or “inner” sphere describes those atoms which are bound directly to the metal center. This sphere is comparatively easy to mimic with inorganic coordination chemistry, and, indeed, an incredible array of homogeneous catalysts exists which rely on clever first coordination sphere designs. The second coordination sphere refers to atoms not directly attached to the metal center but near enough to interact directly with the metal center or with a bound substrate. A classic example from the inorganic community is the famous series of [Ni(P_2_N_2_)_2_]^2+^ catalysts in which a pendant amine not directly attached to the Ni center acts to shuttle protons to and from the active site during proton reduction and hydrogen oxidation, respectively.^18^ This approach is directly inspired by enzymatic chemistry, as will be discussed below. Last, the third coordination sphere refers to atoms or functional groups that influence catalysis without directly interacting with the active site. In enzymes, this can for example mean long-range ordering of water molecules in such a way as to favor catalysis.^19,20^ Also channels that regulate substrate and product transport, as for example in photosystem II,^21^ or redox active cofactors that regulate charge transport^22^ belong to this category. In this review, we will discuss “outer” coordination sphere effects, which encompasses effects induced by second and third coordination sphere design features. In context of MOFs, structurally discrete interactions between the molecular catalyst unit and the surrounding MOF matrix are an example of second, while measures that control and accelerate transport constitute examples of third coordination sphere effects. In parts of section 3.2 and 3.4, we will go beyond intra-MOF effects and take a broader system perspective on catalytic performance, as governed by the dimension of the MOF-based catalyst and its contacting to the macroscopic world.

**Figure 2:**
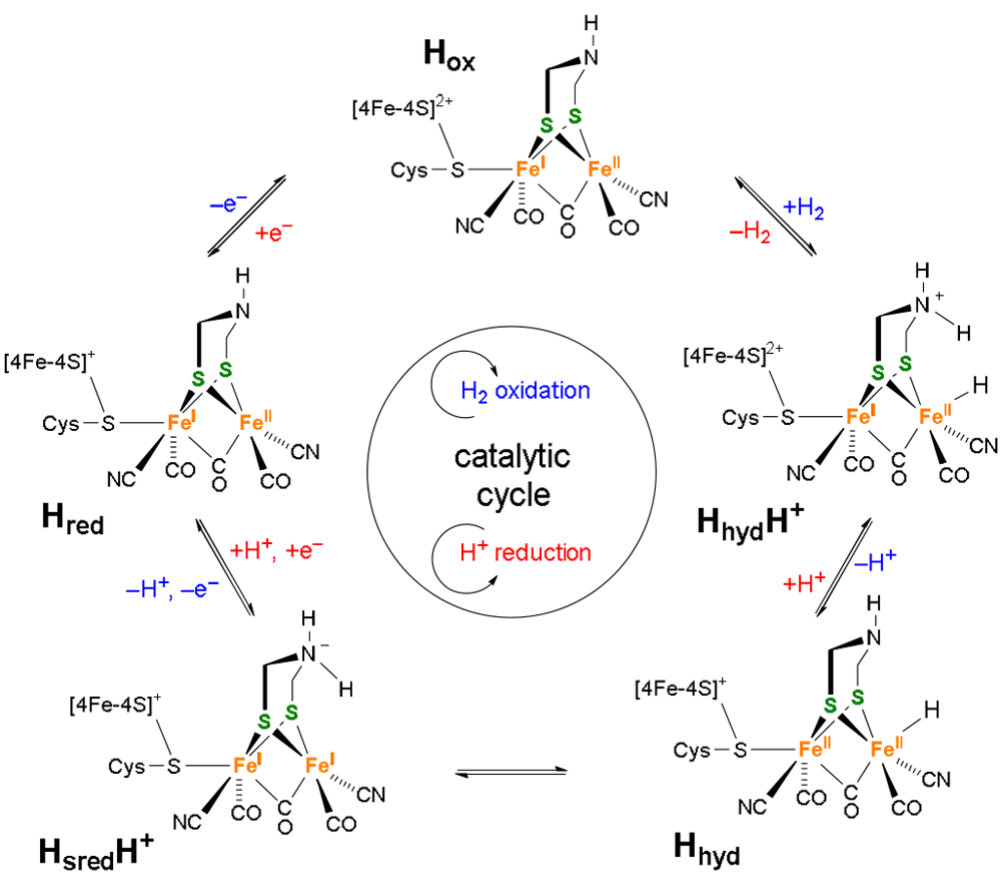
Proposed enzymatic catalytic cycle of [FeFe] H_2_ases for the oxidation of hydrogen (blue) and the reduction of protons (red).^23,27^

To set the stage for the discussion of outer coordination sphere effects in MOFs, we will first highlight some examples of how enzymes utilize outer coordination sphere effects, illustrated on hydrogenase (H_2_ase) enzymes. The catalytic cycle of the [FeFe] H_2_ases commences with a mixed-valent Fe^II^Fe^I^ species with one of the CO ligands in a bridging position, exposing a “free” coordination site of Fe_d_, the iron center of the [2Fe2S] subsite that is distal to the [4Fe4S] cluster (H_ox_ in Figure 2).^23^ Following the cycle in the clockwise H_2_ oxidation direction, substrate H_2_ binds to the free coordination site in a η^2^-fashion and is heterolytically cleaved (H_ox_ → H_hyd_H^+^). The proton is picked up by a nearby nitrogen base which is conveniently positioned above Fe_d_ as part of the azadithiolate (adt) linker. The presence and function of the nitrogen in the adt linkers has long been subject of intense discussions as it could not be unambiguously determined by X-ray crystallography. Spectroscopic work on both enzyme^24^ and model compounds,^25^ as well as in situ H_2_ase enzyme maturations,^26^ have clearly identified the central atom of the dithiolate bridge to be nitrogen, which functions as a proton relay toward Fe_d_.

In the case of some well-studied [FeFe] H_2_ases,^27^ the adt linker is locked in a conformation where the nitrogen is on the same side of the [2Fe2S] subsite as Fe_d_. The other conformation of the six-membered Fe-adt metalloheterocycle is sterically inhibited by the surrounding peptide. This outer-sphere effect that forces the proton shuttling base to face the active metal site is absent in the initial synthetic NiP_2_N_2_ HER catalyst developed by DuBois and co-workers.^28^ In this homogeneous mimic, the pendant nitrogen base can undergo inversion, resulting in a protonated conformer where the proton is physically tucked away from the metal site, and can only re-enter the catalytic cycle through a deprotonation pathway.^28^ Preventing this off-cycle conformation in a subsequent modification produced a considerably faster catalyst that turned over 100 000 times per second, as determined from voltammetric experiments.^29^ Returning to the catalytic cycle in Figure 2, deprotonation of the H_hyd_H^+^ state results in a charge shift within the H-cluster that enables a proton transfer from Fe_d_ to the adt-*N* and the formation of H_sred_H^+^. Two subsequent one-electron oxidations and *N*-deprotonation complete the catalytic cycle.

**Figure 3:**
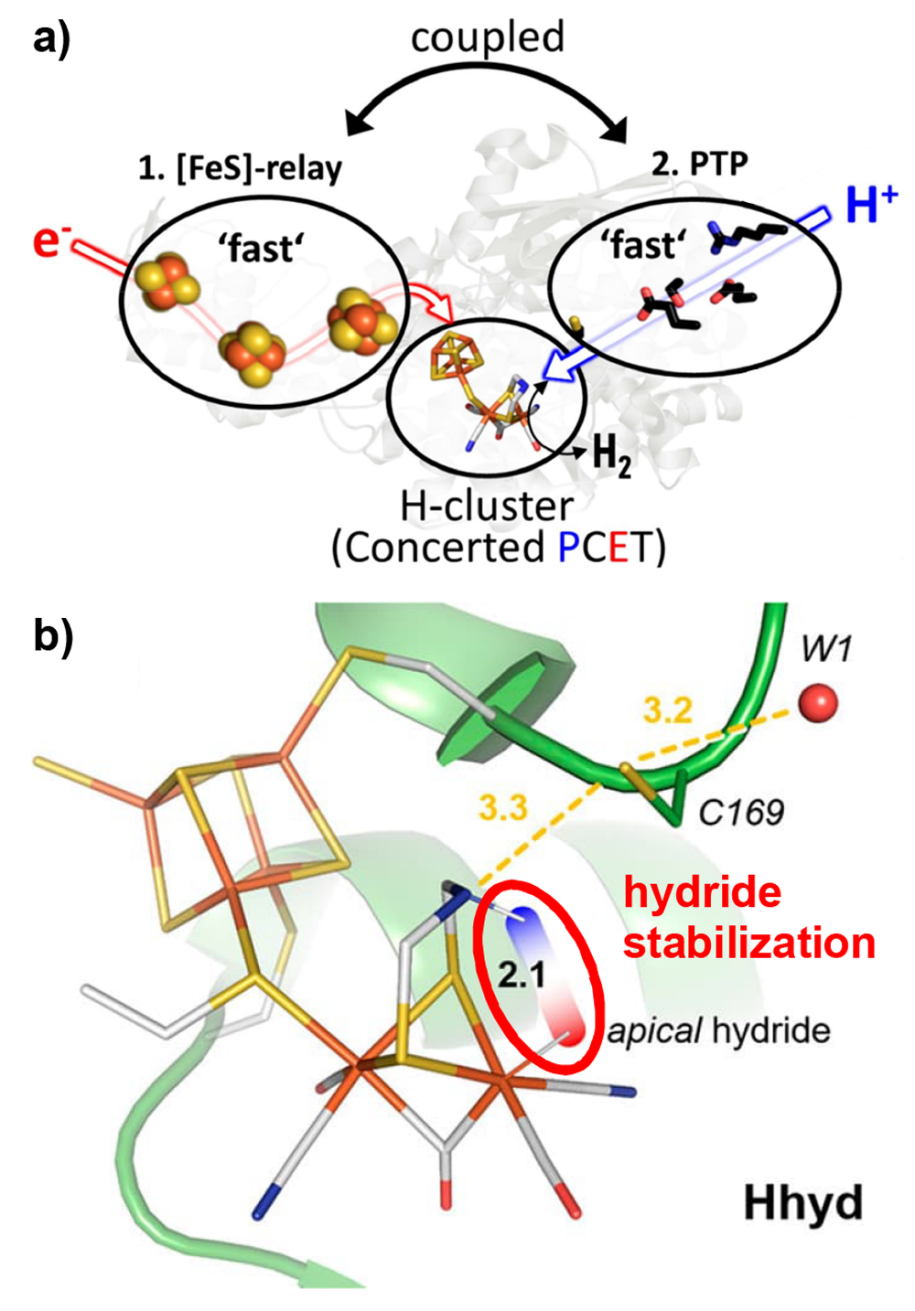
a) Depiction of the proton and electron transfer pathways of FeFe hydrogenase. Adapted with permission from reference (32). Copyright 2020 National Academy of Sciences. b) Active site of FeFe hydrogenase showing how the protonated bridgehead amino group can act, via hydrogen bonding, to stabilize a hydride intermediate. Numbers indicate distances in Å, the red-blue color scheme indicates negative and positive partial charges, respectively. Adapted with permission from reference (33). Copyright 2019 American Chemical Society.

Enzymatic active sites, in particular those that catalyze reactions of energy relevance, are often located in an enzyme’s interior to shield reactive intermediate states from detrimental side reactions. Indeed, decomposition pathways for synthetic molecular species are often bimolecular deactivation or ligand loss.^30,31^ However, if the active site is shielded by encapsulation, as is the case for proton reduction by [FeFe] H_2_ases, the active site is protected from such events. In turn, this however means that substrate and products (protons, electrons and hydrogen) need to be transported through the peptide matrix to/from the active site. This transport is highly regulated, and [FeFe] H_2_ases have dedicated substrate and product channels as well as an energetically highly tuned electron transport chain (Figure 3a).^32^

The degree to which outer-sphere effects influence enzymatic performance is difficult to quantify experimentally. Computational work is a valuable tool in this context, and it was used, for example, in the case of [FeFe] H_2_ases to calculate the energetic effect of the H-bonding interaction between the active site and the surrounding protein matrix. It was shown that the terminal hydride species, which is imperative to the enzyme’s function, is kinetically stabilized by hydrogen bonding interactions (Figure 3b).^33^ Formation of the thermodynamically more stable μ-H product is prevented by a high energy transition state caused by interactions with the surrounding peptide matrix.^34^ In other words, catalysis proceeds through a kinetically stabilized state before it converts to the thermodynamically more stable, and thus less reactive, ground state.

Experimentally, the most extreme showcase of the power of outer-sphere effects in molecular design was reported when a synthetic [FeFe]_H_ subunit model was introduced into the apoprotein of an [FeFe] H_2_ase.^26^ While [Fe_2_(adt)(CO)_4_(CN)]^2–^ is a very limited catalyst by itself,^35^ its incorporation into a tailor-made environment, that is, the apoprotein, triggers a number of structural rearrangements at the metal cofactor that result in a functional enzyme.^36^ This work is the most compelling illustration of the effect that delicate interactions between an organometallic active site and a surrounding environment can have on the performance of a catalytic active site.

**Figure 4:**
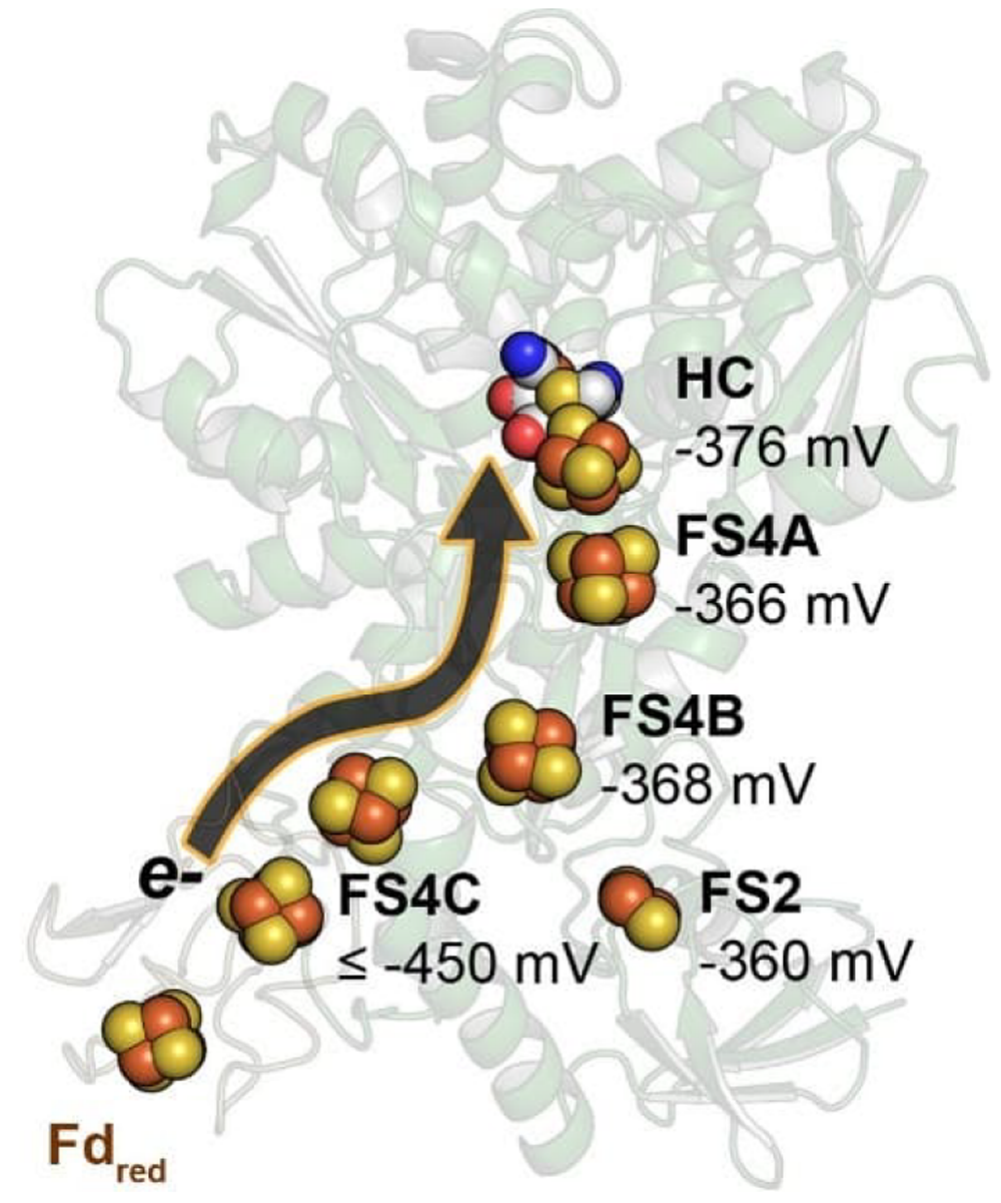
Locations and redox potentials of FeS clusters in the [FeFe] hydrogenase enzyme of *C. pasteurianum*. Reproduced with permission from reference (38). Copyright 2017 American Chemical Society.

Electrons are additional “reactants” that need to access the active sites of redox enzymes, a process that may be managed by dedicated electron transport chains. A common electron relay consists of iron–sulfur clusters, prototypically 4Fe4S clusters. In the case of hydrogenases, there are up to six FeS clusters between the surface of the enzyme and the active site.^37^ These clusters are not all identical, and tuning of their electrochemical potentials by the surrounding peptide matrix gives electron transport a directionality, either to or from the active site, and the enzyme a preference to act as proton reduction, or hydrogen oxidation catalyst. Careful potentiometric titrations coupled with electron paramagnetic resonance (EPR) measurements of the [FeFe] enzyme from *C. pasteurianum* revealed a redox potential gradient capable of “switching on” electron flow when a sufficiently high amount of reducing equivalents was available (Figure 4).^38^

The importance of this carefully tuned long-range electron flow has been demonstrated for hydrogenases. Within the cyanobacterium *N. punctiforme* is a nickel–iron (NiFe) hydrogenase that principally consumes hydrogen as an electron source. By substituting one of the 4Fe4S clusters with a 3Fe4S cluster, Raleiras et al.^39^ were able to reverse the preferred direction of electron flow to afford a hydrogen producing enzyme.

The above examples illustrate how the outer coordination sphere of enzymes has evolved to carefully tune product selection (e.g., proton reduction or hydrogen oxidation), manage electron, substrate, and product transport into and out of the enzyme, and stabilize favorable intermediates and transition states. Clearly, management and understanding of outer-sphere effects affords significant opportunities in the design of synthetic catalysts.

### 1.2 The MOF–Enzyme Analogy: MOFs as Peptide Surrogates to Host Active Sites

**Figure 5:**
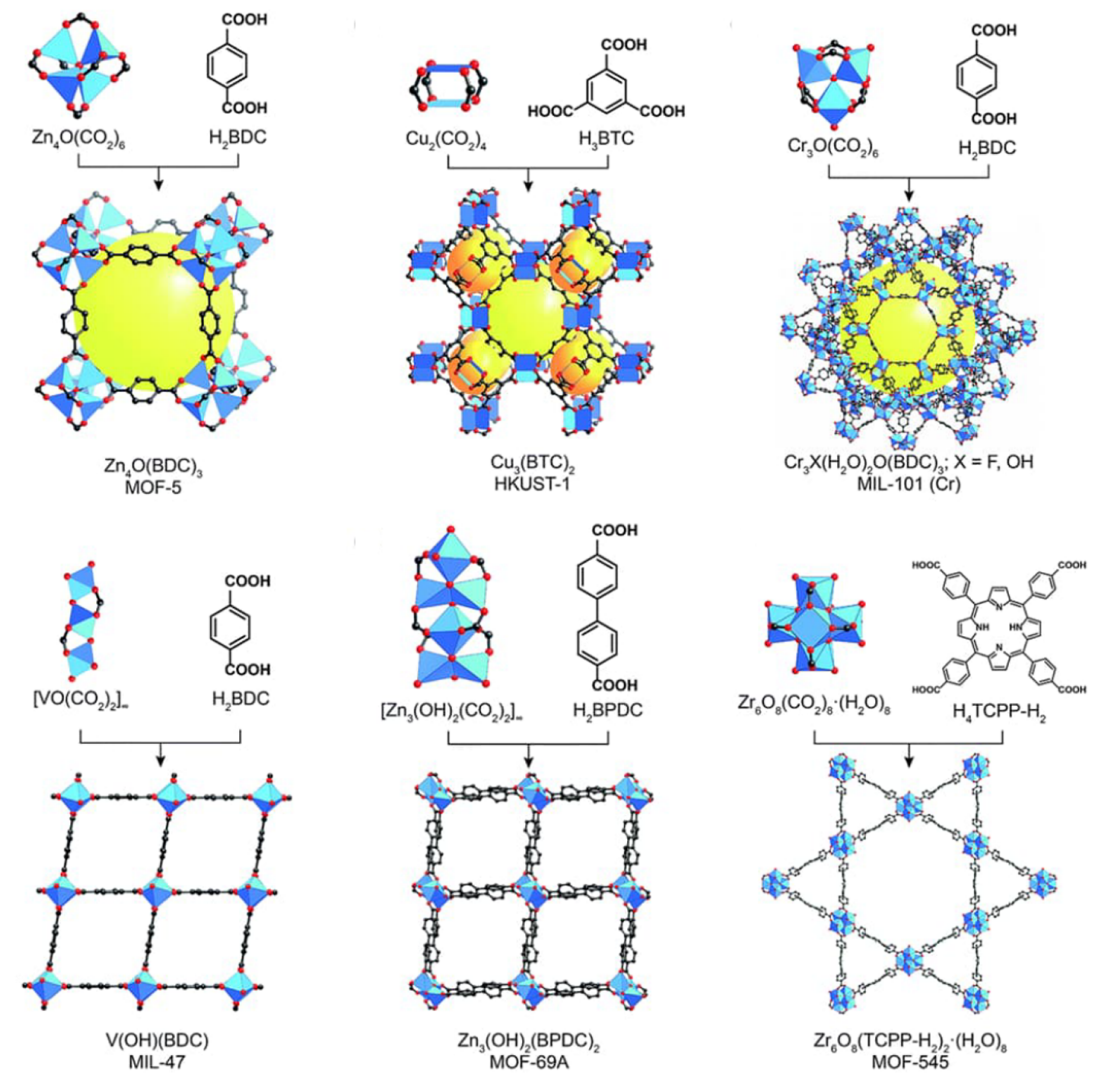
Representation of different SBU and linker combinations to afford a selection of MOFs featured in this review. Atom labeling: C, black; O, red; metals, blue polyhedra. H atoms are omitted for clarity. Yellow and orange spheres represent the space in the framework. Reproduced with permission from reference (53). Copyright 2017 Royal Society of Chemistry.

MOFs are typically built from metal ions or defined metal clusters (secondary building units, SBUs) that are periodically interconnected by organic linker molecules in two or three dimensions, forming a highly ordered crystal lattice of well-defined chemical topology.^40,41^ As illustrated by a few examples in Figure 5, the variation of linkers or SBUs can lead to diverse structures, making MOFs highly tunable materials.^42^ The large number of available SBUs and linkers enables the design of countless MOFs, showcasing unique structures and differing in topology, reactivity, stability, pore size, and internal surface area.^43−45^ Some MOF materials exhibit internal surface areas as high as 7800 m^2^ g^–1^, higher than that of any other material known.^46^ Consequently, MOFs have attracted large interest for gas storage and separation, a field that is still highly relevant today.^47^ The large surface area is, however, also relevant for many other applications, and MOFs have been explored as platforms for the incorporation of different functions.^48−50^ Reviewed herein are catalytic reactions of energy relevance, as large internal surface areas that contain accessible catalytic sites can lead to high activity.^43−45^ An important aspect to such endeavors is the development of strategies to lend MOFs high hydrolytic stability^51^ as well as stability under electrocatalysis conditions.^52^

Molecular and nonmolecular species with a desired function (e.g., catalysis) such as nanoparticles or even small enzymes can be integrated into a MOF.^54^ In general, the functionality can be introduced to the MOF either during its synthesis, often solvothermally, or in a postsynthetic method. The latter includes postsynthetic exchange where linkers or SBU-constituting metals from pristine MOFs are replaced by exogenous counterparts, postsynthetic ligation at a free coordination site of an SBU, postsynthetic metalation, or by using synthetic organic chemistry on linkers with suitable reactive groups.^44,55−57^

Catalysts of energy relevance have been introduced by most conceivable methodologies. In context of molecularly defined catalysts, the catalysts can in principle be held inside the MOF matrix either by a physical entrapment, nonspecific forces such as hydrophobic–hydrophobic interactions, coordination bonds, including H-bonds, or covalent bonds.^44^ Of course, the SBU, stabilized by the coordinating linker, can itself be catalytically active,^45^ as for example seen in many Lewis-acid promoted organic transformations.^58,59^

### 1.3 Scope of the Review: Outer Coordination Sphere Effects in MOFs

This review summarizes reports on molecular catalysis of energy relevance in MOFs. Whenever possible, analogies to enzymes are drawn by analyzing interactions between the MOF scaffold and the catalytic site. Such interactions may be in close proximity to the active site, that is in the second coordination sphere, or more remote in the third coordination sphere. The benefits that arise from MOF incorporation may be multiple, and the original research papers in the field have been organized by the most discernible MOF–catalyst interaction. Such interactions may be of simple structural nature, stabilizing molecular integrity of the catalysts under turnover conditions, giving rise to higher turnover numbers (TONs) as compared to those of homogeneous reference compounds (section 3.1).^43,44^ Given the microporous nature of MOFs, transport to/from catalytic sites that are remote from the MOF crystal surface can easily be limiting the overall efficacy of the material,^60,61^ and works on facilitating transport issues are addressed in section 3.2.^62^ The presence of substrate and/or electron transport pathways bears resemblance to corresponding channels in the third coordination sphere of enzymes. In the absence of such engineered intra-MOF transport channels, limitations can be overcome at a system level by simply decreasing the dimension of the catalytic MOF at hand. Such examples will also be reviewed in section 3.2. Zooming closer into the catalytic site and its secondary coordination sphere, specific and atomically well-defined interactions between the catalytic site and the MOF matrix are discussed in section 3.3. Such cooperative effects may, for example, lower the transition state of a rate-determining step of the catalytic cycle, thereby accelerating catalytic turnover.^63^ The final section 3.4 reviews contributions on different means to interface electrocatalytic MOFs with electrode substrates. This section again addresses catalytic efficiency at a system level, but has its counterpart in the enzyme world where the way by which redox enzymes are anchored on electrode substrates has a great impact on observed current densities.^64−66^

It is important to point out that different aspects of the present review have been summarized previously.^43,44,67−72^ The interested reader is referred to excellent reviews on MOF-based biomimicry,^45,73−77^ MOF-based oxidation reactions,^78^ in particular OER,^79−81^ MOF-based HER,^82−84^ CO_2_RR,^85−87^ and ORR,^88^ often comprising not only molecular but also material catalyst systems.^89,90^ Drawing parallels between biological systems and catalysis of energy relevance in MOFs, the present review is exclusively focused on molecular catalysts. Catalytic units that are not molecularly defined, as well as entire enzymes that are embedded in MOFs are outside the scope of this review.^91−93^ The redox equivalents for the reactions may be provided either chemically, electrochemically, or photochemically. The source of redox equivalents may not necessarily be from molecular oxidants/reductants or photosensitizers (PSs) but could also originate from heterogeneous materials such as quantum dots for light harvesting, or conducting substrates in the case of electrochemical processes. Redox equivalents (holes or electrons) and substrates (CO_2_, water, or O_2_) come from different sources, and hydrogenation reactions are outside scope of this review.

As higher coordination sphere effects generally aim to accelerate the catalyzed reactions, this review also contains an extensive survey on the kinetics of molecular catalysis in MOFs (section 2). This is important because acceleration of the catalytic reaction may more often than not be masked by other limiting phenomena. In chemically or electrochemically driven catalytic processes, this may be mass or charge transport limitations, while light-driven catalysis comes with the additional complication that sacrificial reductants or oxidants are often present in the MOF only in small amounts. Moreover, productive light-driven charge separations are often reversed by back electron transfer pathways, thereby short-circuiting catalytic mechanisms. All of these phenomena affect the observed rate of product formation of the whole system. In order to benchmark catalytic MOF systems, and to make meaningful and informed improvements, it is thus imperative to discern the limitation under which a specific catalytic MOF system may operate.

## 2 Kinetics of MOF-Based Molecular Catalysis

A fundamental difference between homogeneous catalysis and catalysis supported in porous materials is the heterogeneous nature of chemical reactions, where mass and charge transport cause substrate, intermediate species, and product concentrations to not only depend on time but possibly space as well. In these situations, concentrations inside the porous support matrix change over the length of the particle or thickness of the film and may differ significantly from bulk concentrations at the boundaries. In the context of biomimicry, introduction of higher coordination sphere effects that may accelerate the intrinsic catalytic rate may not manifest themselves as clear changes in the observed rate of product formation or turnover frequency (TOF). If the substrate and charge transport are relatively slow compared to catalysis, differences in the overall activity as a direct result of outer coordination sphere effects may be difficult or impossible to distinguish. Therefore, to assess the benefit of the pore modifications mentioned above and identify the role of any second coordination sphere interactions, it is necessary to take measurements under conditions where charge and mass transport do not contribute to kinetic control. A detailed kinetic and mechanistic analysis considering both the diffusion of substrate and electron hopping is therefore a prerequisite to determine if molecular catalysis in MOFs can reach enzyme-like properties. In general, this can be accomplished by collecting experimental data over a large range of each operational parameter, as well-demonstrated by recent advances in homogeneous molecular catalysis of electrochemical reactions.^94,95^

To determine the effect of the framework on the catalytic reaction, it may be required to compare the heterogenized molecular catalyst within the MOF to a homogeneous analogue (if one exists). As has been re-emphasized recently,^96,97^ varying operational parameters (concentrations, light intensity, or experimental time scales) are used to establish possible mechanistic pathways and kinetic rate laws, while molecular catalysts should be compared based on intrinsic parameters (standard potentials, intrinsic rate constants). Establishment of the TOF expression for a given mechanism by kinetic modeling is consistently required, as it is usually a composite function of several rate constants for different steps in the catalytic cycle, especially where the catalytic mechanism is a network of multiple pathways.

For heterogenized molecular catalysts in porous materials, the transport properties of the framework are also important to consider when comparing catalytic systems. The relevant operational parameter is the geometric length scale (film thickness/particle size) of the finite domain where the reaction-diffusion process is occurring. Varying this will switch the observed catalytic rate between reaction-limited and transport-limited, from which transport properties can be measured (diffusion coefficients). When interpreting kinetic data of experimental systems, a clear distinction between operational and intrinsic parameters as well as between reaction-limited and transport-limited rates is important for drawing mechanistic conclusions, such as the involvement of through-space or second coordination sphere interactions. In the following sections, we summarize several physico-mathematical models that provide a rational basis for kinetic assessment and benchmarking of chemically-, electrochemically-, and photochemically-driven molecular catalysis within MOF particles or films, and point out possibilities and pitfalls. As many reports that will be reviewed herein do not discriminate between the apparent turnover frequencies (TOF^app^) that are a composite number and the true TOF^true^ that is an intrinsic catalyst metric, we consciously refrain from citing TOFs and TONs. This is to discourage comparisons of performance numbers that should not be compared because they may be influenced by different limiting phenomena. Instead, we provide guidelines that will help the reader to identify these phenomena, and to evaluate whether comparisons of performance parameters of different systems are meaningful.

### 2.1 Chemically Driven Molecular Catalysis in MOFs

**Figure 6:**
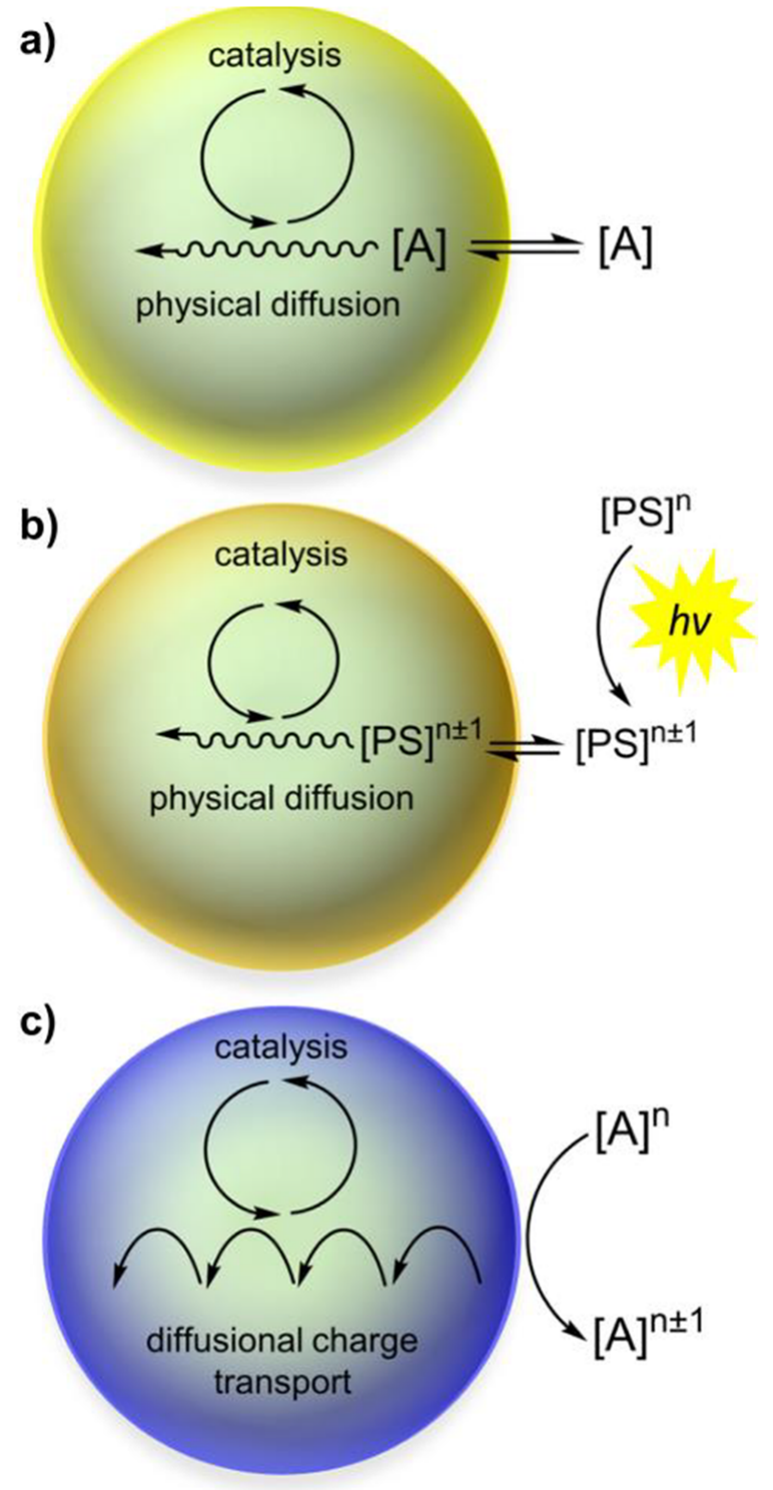
Cases for molecular catalysis in MOF particles. The scenario including a photosensitizer depicted in b) will be discussed further in section 2.3. Reproduced with permission from reference (98). Copyright 2020 Royal Society of Chemistry.

In many examples of MOF-based molecular catalysis, the reaction is driven by a sacrificial chemical reagent, which is added to a suspension of MOF particles. In the case of catalysis of energy relevance, this is a strong oxidant or reductant that delivers electrons or holes to the active sites in the MOF interior, but this situation could apply to thermochemical organic transformations as well. Assuming that catalysis occurs in the MOF interior and not only at exposed surface sites, both the sacrificial reagent and the substrate must diffuse into the MOF pores for the reaction to proceed (Figure 6a). In the case of redox reactions, the sacrificial oxidant/reductant could otherwise initiate a diffusional charge hopping process at the surface of the particles that carries electron or holes to the molecular catalysts within the framework (Figure 6b). Consequently, measuring the intrinsic rate constant of the incorporated molecular catalyst will require accounting for mass transport of the substrate/sacrificial reagent and possibly charge transport of electrons or holes.

First introduced in the 1930s independently by Damköhler^99^ and Thiele,^100^ the theoretical basis for catalytic reactions in porous media is well-established for basic mechanistic schemes. Several simple criteria can be used to determine the extent of substrate mass transport resistance and provide the intrinsic rate constant of the catalytic reaction. For a first-order reaction in a porous catalyst particle at steady-state, the competition between reaction and diffusion of the substrate is controlled by the dimensionless parameter ϕ, called the Thiele modulus (here we will refer to ϕ in eq 1, but the same quantity is also commonly denoted as the Damköhler number (Da) with ,
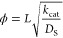
1where *L* is the critical length scale of the particle (e.g., radius for spherical particles), *D*_S_ is the intraparticle diffusion coefficient of the substrate, and *k*_cat_ is the first-order rate constant of the catalytic reaction. For ϕ ≪ 1, the reaction is kinetically controlled by the intrinsic rate of catalysis. Conversely, when ϕ ≫ 1, there is mixed kinetic control by reaction and diffusion, and the catalytic particle will display significant transport resistance. In this case, when ϕ ≫ 1, this means that the observed rate (*v*) is proportional to both the diffusion coefficient of the substrate and the (pseudo)first-order catalytic rate constant raised to the one-half power:
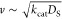
2

**Figure 7:**
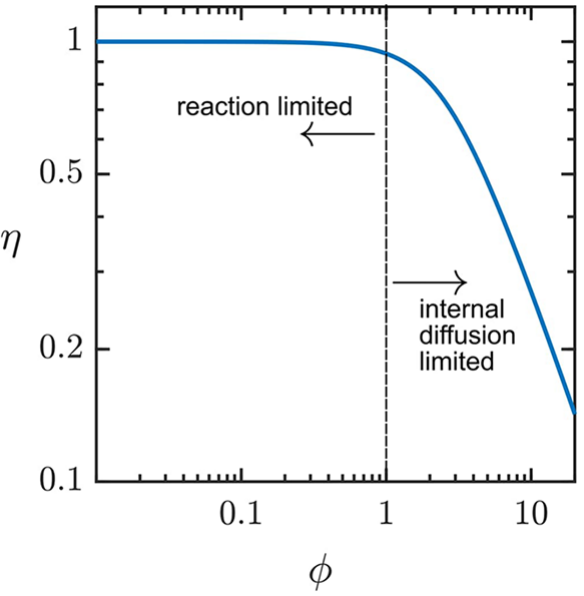
Plot of the effectiveness factor as a function of the Thiele modulus of spherical particles.

Kinetic measurement under these conditions will contain artifacts related to the mass transport of substrate through the particle. Experimentally, these two kinetic regimes can be accessed by systematically varying the size of the particle (given by *L*) and measured by the internal effectiveness factor, η, which is a metric for the extent of diffusion of the substrate in a catalytic particle. It is defined as the ratio of the observed reaction rate to the reaction rate in the absence of transport effects (when the whole interior of the particle is uniformly exposed to the conditions on the surface). A plot of the effectiveness factor as a function of the Thiele modulus is displayed in Figure 7.

Kinetic measurements taken in the reaction-limited region (η = 1, ϕ ≪ 1) will yield the intrinsic rate constant and should be used for meaningful comparison between heterogenized molecular catalysts. On the other hand, the observed rate in the internal diffusion-limited region (η < 1, ϕ ≫ 1) can be used to calculate important parameters for intra-MOF substrate transport such as diffusion coefficients (*D*_S_). We note that transport-limited rates can be useful in obtaining kinetic constants if an independent measurement of both the dimension of the particle (*L*) and the intra-MOF diffusivity of the substrate (*D*_S_) can be made. A physico-mathematical model for the operative mechanism can then be formulated to relate the measured product formation rate to the intrinsic catalytic rate constant(s), with the parameters associated with the diffusion of substrate (*L*, *D*_S_) being known quantities.

#### 2.1.1 Measuring Molecular Diffusion in MOFs and Implications for Chemically Driven Catalysis

As mentioned above, molecular diffusion rates for substrates as well as other coreagents are valuable parameters for analyzing kinetic data, as well as benchmarking transport limitations (via the effectiveness factor). This is quantified by the intracrystalline diffusivity (*D*_S_)^61^ measured under conditions relevant to molecular catalysis, typically with solvent-filled pores. Although the gas-phase self-diffusivity of guest molecules in MOFs has been investigated extensively, solution-phase studies of intracrystalline transport are relatively limited.

In a recent example, Wang et al.^101^ followed the transport of a luminescent probe (1,3,5,7-tetramethyl-4,4-difluoroboradiazaindacene) through the one-dimensional (1D) pores of **NU-1008** using confocal fluorescence microscopy. Measurements conducted in situ on individual crystallites yielded transient concentration profiles, which could be fit to an analytical solution of Fickian diffusion in a finite domain, including transport resistance (“surface barriers”) across the particle/solution interface. Experimentally obtained diffusivities were in the range of 10^–8^ cm^2^ s^–1^, approximately 2 orders of magnitude smaller than that measured in bulk solution of the same solvent. Significant pore confinement and host–guest π–π interactions were suggested as possible reasons for the slower diffusion through the solvent-filled pores of **NU-1008**. Ideally, microimaging techniques of single crystals, which provide the entire transient concentration profile of guest molecules within the framework, are more accurate at predicting diffusivities. Macroscopic uptake measurements, made by stepping the concentration of the guest molecule in the surrounding bulk solution or atmosphere and recording its change over time, were found to often lead to an incorrect estimation of such transport properties.^102^

**Figure 8:**
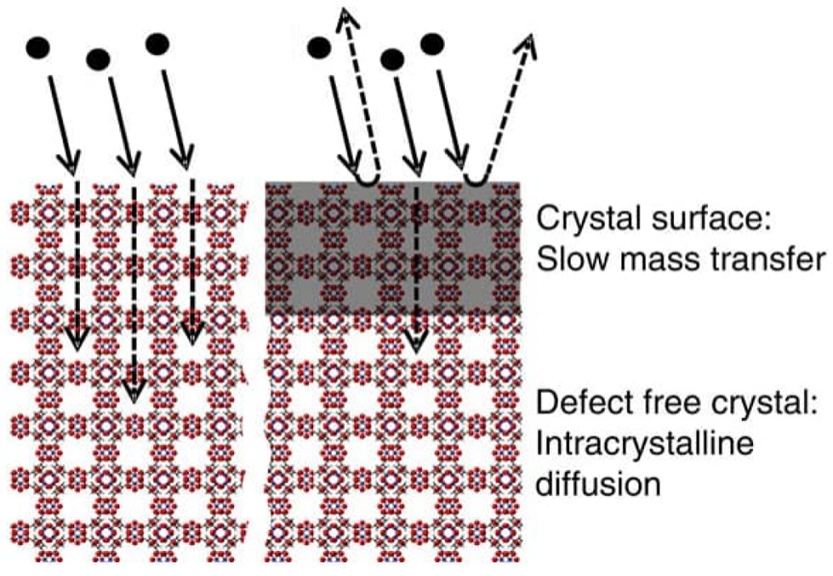
Schematic illustration of absorbate molecules encountering “surface barriers” at the crystal surface. In the case of catalytic reactions involving small molecule activation and energy conversion, this phenomenon would occur at the MOF-solution/electrolyte interface. Adapted with permission from reference (106). Copyright 2014 Springer Nature.

Furthermore, the phenomenon of surface barriers, as mentioned above, frequently has been observed for uptake of solution phase guest molecules into MOF crystallites. This can be described as a finite rate of guest molecule penetration from solution through the outer surface of the MOF.^103,104^ This kinetic phenomenon was identified as a possible rate-limiting process and subsequently accounted for in models of catalysis within redox-polymer-modified electrodes^105^ and thus has implications for molecular catalysis in MOFs. The exact physical nature of surface barriers in MOFs is unknown, but reports suggest that defects (possibly pore collapse) at the surface of MOF particles or films formed during solvothermal synthesis slow down mass transfer at the crystal surface (Figure 8).^102,106^

In particular, large linear 1D channels may provide advantages for mass transport; however, further research is required in the rational design of MOF structures that are tailored to enhance substrate mass transport for catalysis by considering factors like tortuosity and constriction. Current literature reports have explored a few variations on the framework topology (**UiO-6x**, **NU-1000**, **PCN**, **MIL-101**), many of which have design properties fitted to other applications (e.g., gas sorption), rather than solution-based catalysis, which requires facile mass transfer through solvent-filled pores.

Several examples exist where the dimensionality of the whole framework has been reduced specifically to enhance mass transport rates. Catalytic two-dimensional (2D) MOFs, sometimes referred to as metal–organic layers (MOLs)^107^ have been shown to have higher observed rates of product formation than the analogous 3D frameworks with the same catalytic active site. This takes advantage of the fact that a thin slab is the optimal geometric shape to minimize diffusion time of a substrate in a given volume. This strategy has also been employed for electrochemical CO_2_ reduction mediated by molecular catalysts stabilized within 2D MOF materials.^108,109^

#### 2.1.2 Relevant Kinetic Models for Chemical Catalysis in Porous Media

While the mathematical analysis of reaction-diffusion in porous particles is well-known for simple, single step reactions,^110,111^ small molecule activation and solar fuel production in MOFs are inherently multielectron, multistep processes. Explicit treatment of multistep catalytic reactions coupled to mass transport is highly relevant in this regard. Just as in the analysis of homogeneous molecular catalysis of electrochemical reactions,^96^ a derivation of the analytical rate expressions (approximate or exact when possible) for a given mechanism is important. This is because, for a multistep mechanism, the system of differential equations that needs to be solved is highly coupled and potentially nonlinear, due to intermediates being generated and consumed as well as owing to the presence of multiple reaction pathways. Certainly, each mechanism will have a range of possible solutions for the concentration profiles of substrate and intermediates and the overall observed reaction rate, which depend on limiting values of the intrinsic and operational parameters. Kinetic analysis of molecular catalysis in MOFs could benefit from an update of the single-step Thiele model, tailored for solar fuel reaction mechanisms (OER, HER, ORR, CO_2_RR, and so forth).

**Figure 9:**
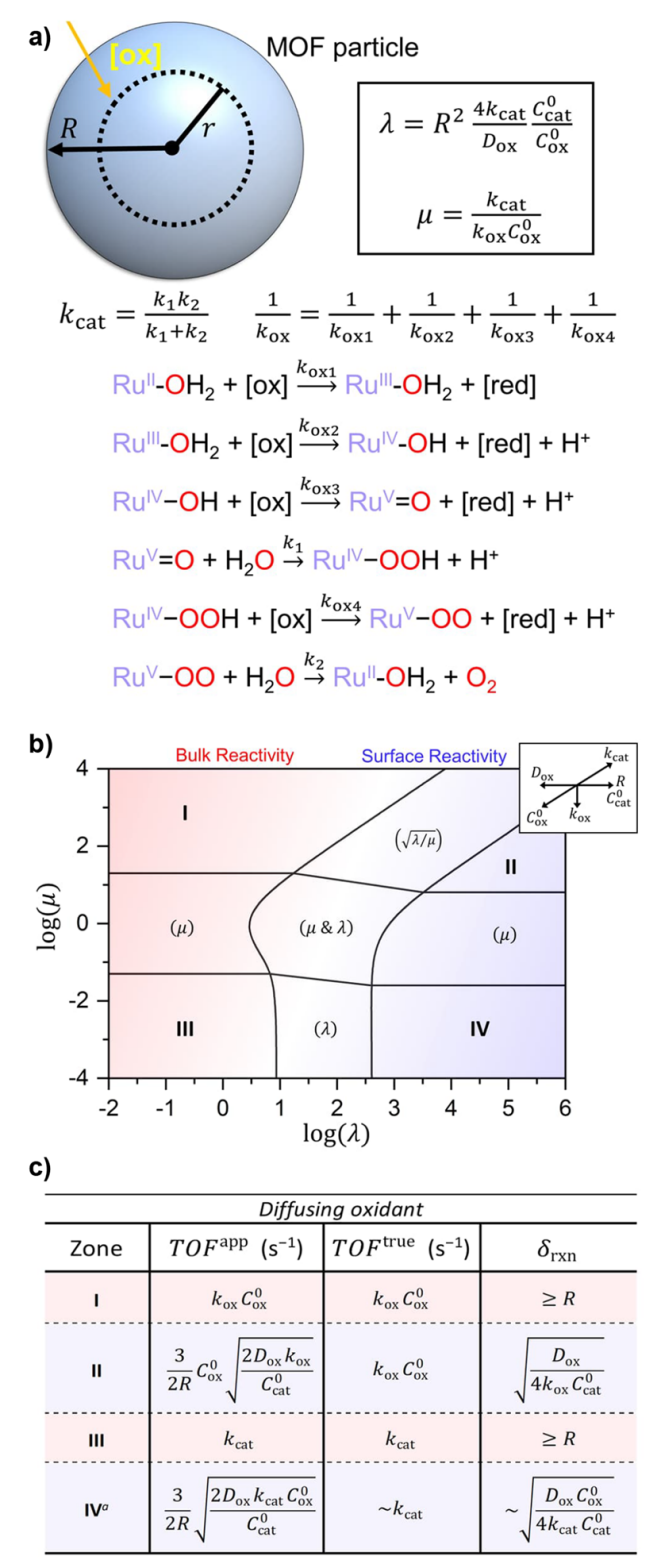
a) Water-nucleophilic attack mechanism with diffusion of the sacrificial oxidant through MOF particles (approximated as a sphere). The kinetic behavior is completely controlled by two dimensionless governing parameters: λ, a parameter that measures diffusion competing with the catalytic reaction and μ, a competition parameter for the rate-limiting chemical reaction step. b) Zone diagram summarizing all limiting kinetic behaviors with closed-form rate expressions. The upper right-hand compass shows how the variation of each operational and intrinsic parameter translates the system between reaction-limited (bulk reactivity) and transport-limited (surface reactivity) kinetics within the zone diagram. c) TOF expressions for each kinetic zone. Adapted with permission from reference (98). Copyright 2020 Royal Society of Chemistry.

For example, Johnson et al.^98^ developed a quantitative model for OER under steady reaction-diffusion in spherical MOF particles, catalyzed by a molecular Ru water oxidation catalyst (WOC), and driven by a sacrificial oxidant. It was shown that the diffusion of the chemical oxidant could become significant, where its concentration will vary significantly over the radius of the particle. Two possible OER mechanisms for molecular Ru catalysts (Figure 9a displays the common water-nucleophilic attack mechanism) were analyzed and found to both give rise to nonlinear reaction rates, which were coupled with diffusion in axisymmetric spherical coordinates. All limiting kinetics behaviors can be summarized in a zone diagram (Figure 9b). This analysis allowed for deriving expressions for the observed reaction rate as a function of various operational and intrinsic parameters. Importantly, two definitions for the TOF were proposed: an *apparent TOF*, which normalized the rate to the total amount of catalyst present and a *true TOF*, which appropriately considers only the amount of active catalyst in a catalytic particle:
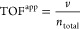
3
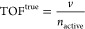
4where *v* is the reaction rate in mol s^–1^, *n*_total_ is the total moles of catalyst within the MOF particle, and *n*_active_ is given by
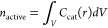
5the integral of the concentration of the catalyst over the volume of the particle *V*, where *r* is the radial distance in spherical coordinates. If diffusion is relatively fast, the active catalyst concentration is homogeneous throughout the particle, and TOF^app^ is equivalent to TOF^true^. However, if diffusion is slow enough compared to the rate of the reaction, the active catalyst concentration will be inhomogeneous across the spatial dimension of the particle (i.e., gradients will develop). When this occurs, it was found that apparent TOFs, as they are most frequently reported, deviate from the true activity of the molecular catalyst and are a composite function of many different parameters (Figure 9c). For example, when diffusional transport of the oxidant is kinetically significant
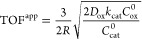
6where *R* is the radius of the particle, *D*_ox_ is the diffusivity of the sacrificial oxidant, *C*_ox_^0^ is the bulk oxidant concentration, and *C*_cat_^0^ is the total catalyst concentration. The catalytic reaction for the OER can be described by a pseudo-first-order rate constant *k*_cat_ = *kC*_S_^0^, where *C*_S_^0^ is the substrate concentration. Here, since water is the substrate and solvent, *C*_S_^0^ remains constant with respect to space and time (for more information on the mechanistic model, relevant assumptions, and derivation of eq 6 see the SI of reference (98)). This predicts that the calculated apparent TOF will not only depend on the size of the particle (*R*) and the diffusivity of a particular cosubstrate, but will also give a falsified negative one-half reaction order with respect to the catalyst. Significant errors will result if a value such as in eq 6 is inferred to represent only the molecular catalyst’s intrinsic reactivity. Therefore, apparent TOFs calculated using the total amount of catalyst should be viewed as mixed kinetic parameters, unless intra-MOF transport limitations can be exclusively ruled out.

Currently, only a handful of experimental reports of MOFs with applications to heterogeneous catalysis have investigated the relationship between the size of the particles and the observed catalytic activity.^112,113^ For MOFs, this may be due to difficulties with synthesizing particles of different sizes while keeping all other parameters constant (e.g., defect sites, excess modulator coordinated to nodes). Although, with appropriate reaction-diffusion modeling, it may be possible to interpret kinetic data and identify transport limitations. For example, Wang et al.^114^ investigated cerium ammonium nitrate (CAN)-driven water oxidation by an Ir complex incorporated into a **UiO-69**-type framework. The reaction rate was determined to be first order with respect to the CAN concentration through kinetic measurements that followed the consumption of CAN in the bulk solution by UV−vis spectroscopy. Fitting the experimental data to a reaction-diffusion model for the first-order consumption of CAN within the particles revealed that the oxidant only penetrates 11% in depth at steady-state, due to the slow diffusion of the oxidant relative to the fast catalytic reaction.

### 2.2 Molecular Catalysis of Electrochemical Reactions in MOFs

#### 2.2.1 Conduction Mechanisms

Conduction in MOF materials can occur through several different processes. These can be broadly categorized by the mode through which charge is transported. Band or ohmic conduction operates solely through migration, where an electrostatic potential gradient provides the dominate driving force for charge carrier transport through the MOF material. This requires strong electronic coupling between the linkers or nodes in the MOF, either through-bond or through-space, such that the electronic states are highly delocalized. It is relatively easy to verify this type of conduction when examining the current response of MOFs of this type deposited on electrodes. In the absence of any substrate or coupled chemical reactions, these materials will only display capacitive current as no charge transfer is taking place across the interface. Some MOFs have been reported to exhibit properties similar to semiconductors or even conductors.^115−117^ A typical example is the Ni_3_(hexaiminotriphenylene)_2_ 2D MOF reported by Dincǎ and co-workers.^118^ Upon addition of a substrate into the bulk solution, Faradaic current may be observed as the result of an inner-sphere electrochemical reaction between an active site in the MOF material and a substrate molecule. Electronic structure calculations can be used to indicate whether the MOF should be classified as semiconductor or insulator, where a semiconductor should have a large bandwidth (dispersion). Such calculations have shown that MOFs with carboxylate-based linkers are insulators, as they exhibit very flat and narrow bands and a total dispersion in the valence band of only ∼0.1 eV.^119^ Optical charge transfer bands that may appear in the absorption spectrum are a result of localized electronic states with little delocalization. This situation applies to a large proportion of MOFs discussed in this review, as many of them are constructed from carboxylate-based linkers.^117^ It should be pointed out that some authors incorrectly assume that such MOFs are semiconductors with band-like conduction.

Conversely, conduction may occur via electron hopping, formally a diffusion process, where the driving force for charge transport is primarily a chemical potential gradient. This gradient is classically generated through outer-sphere electron transfer at an electrode/MOF interface, but could also be present due to “localized” chemical oxidation or reduction of components of the MOF at the surface of a film or particle. This can occur if the chemical oxidizing/reducing agent is size-excluded from MOF pores and cannot diffuse further into the interior of the MOF particle/film. These MOFs have highly localized electronic states, characterized by a standard potential (*E*^0^). Additionally, the current–potential response of MOFs exhibiting this mode of conduction will display Faradaic current in the absence of a substrate, attributed to the outer-sphere reduction or oxidation of the molecular components of the MOF and subsequent diffusional electron hopping through the material.

This distinction in the type of conduction pathway is very important for an accurate interpretation of experimental kinetic data. Outer-sphere electron hopping necessitates stepwise mechanisms (ECEC, EECC; where E represents an electrochemical step and C represents a chemical reaction step). This allows for the concepts and tools developed for homogeneous molecular catalysis of electrochemical reactions^120^ to be utilized with modification to include diffusion of substrate and diffusional charge transport^121−123^ for analyzing the current–potential response of this type of MOF. On the other hand, MOFs with band or ohmic conduction will have inner-sphere catalytic mechanisms resembling traditional electrocatalysts.^124^ Microkinetic models for inner-sphere mechanisms typically rely on Tafel analysis of the current response at high overpotentials.^125,126^ When the steady-state catalytic current is large, care must be taken to avoid mass transport limitations within the material^121^ as well as in solution.^127^ For example, recently Unwin, Dincǎ, and co-workers^128^ compared data from planar rotating disk electrodes, gas diffusion electrodes, and scanning electrochemical cell microscopy to reveal O_2_ transport limitations at multiple scales for the ORR by conducting Ni_3_(HITP)_2_. It was shown that this MOF’s activity has been underestimated by several orders of magnitude due to mass transport limitations present in previous measurements.

In this review, we mainly focus on MOFs with electron hopping conduction since this type of conductivity also implies discrete molecular active sites, which are more relevant when considering rationally designed bioinspired or biomimetic higher coordination sphere effects. Biological electron transport in and between proteins typically occurs by electron hopping between discrete redox centers, over distances of up to 15 Å for a single step.^129,130^ It should be noted that some MOF materials may display conduction behavior with characteristics of both mechanisms discussed here (band and hopping), and charge transport can be described as a hybrid process. Recently, this has been shown to be the case for porous metal oxide catalysts for the OER.^131,132^

#### 2.2.2 Kinetic Analysis of Heterogenized Molecular Catalysis of Electrochemical Reactions

The kinetic analysis of heterogenized molecular catalysis of electrochemical reactions has a long history, starting in the 1980s with redox-polymer-modified electrodes.^133^ This subject has recently been well-reviewed.^134,135^ Indeed, these kinetic models can be applied to MOFs as well.^60^ Rather than presenting these in detail, in this section we will only focus on two specific metrics regularly used to compare the catalytic activity of electroactive MOF film electrodes, namely, the TOF and the onset potential, and briefly discuss their modern-day usage and potential utility.

##### 2.2.2.1 Turnover Frequencies (TOFs)

Many reports that will be discussed in this review present TOFs for catalytic reactions based on comparisons of two or more systems which differ by a certain structural aspect/property. As discussed above, when dealing with catalysis in porous materials the TOF is a function of the intrinsic reaction rate, but potentially also of many other factors, such as diffusion coefficients, particle size, or film thickness. In principle, it is also a transient function, changing dynamically over time and space as the catalytic reaction approaches a steady-state.^136^

If experimental conditions are controlled, it is possible to measure a steady-state current, which can be then used to compute the TOF. In the ideal case, the catalytic reaction takes place homogeneously throughout the film (the concentration of active catalyst and substrate are at their bulk values over the entire length scale defining the film). Converting the steady-state current density to a TOF, which can be used to compare catalytic systems, then only requires dividing by the total amount of catalyst in the film using Faraday’s constant as a conversion factor.

However, if at the steady-state the concentration of the active catalyst or the substrate is not spatially homogeneous across the film, converting the current to a TOF is not as simple. This situation will arise when the distance that a diffusing species travels into the film during the time scale of the catalytic reaction is significantly less than the geometric length scale of the film. Boundary layers develop where the active catalyst or substrate concentration is depleted within a small distance from one of the film’s edges. Consequently, the reaction only takes place in a small reaction layer of size , where *D* is either the electron hopping diffusion coefficient or the diffusion coefficient of the substrate, and *k*_cat_ is the intrinsic catalytic rate (s^–1^). This means that the amount of active catalyst is much less than the total, and simply dividing the steady-state current by the total amount of catalyst will result in an apparent TOF, which underestimates the molecular catalyst’s true activity (see also discussion around eq 3 and 4 above).

Varying the film thickness can be used as a strategy to diagnose when transport limitations are present and to position the catalytic system such that the measured current reflects an intrinsic rate. If the steady-state current varies linearly with the film thickness, transport limitations are absent. On the other hand, the catalytic current remaining constant as the film thickness is increased signals that the catalytic reaction is faster than diffusion. Again, TOFs measured under these conditions will underestimate *k*_cat_, and will depend on more parameters than just the catalyst’s intrinsic activity (diffusivities and film thickness). Generally, using suitably thin films such that  (where *d*_f_ is the film thickness) remedies these issues.

##### 2.2.2.2 Onset Potentials

**Figure 10:**
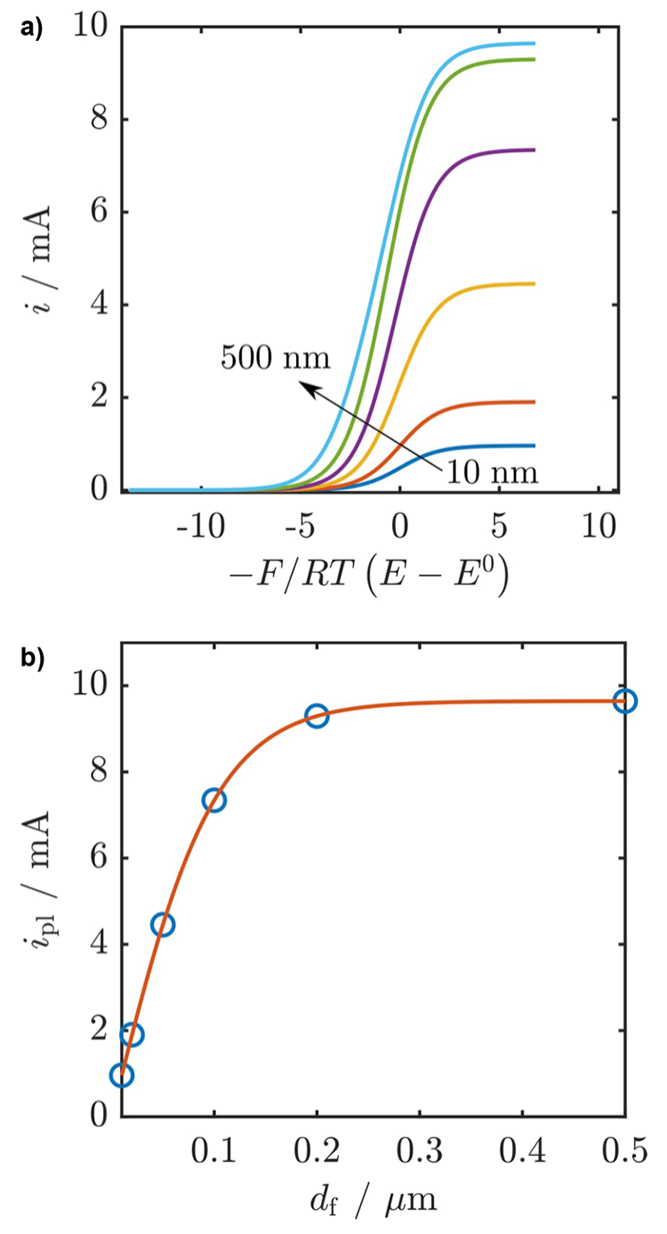
a) Steady-state cyclic voltammograms of catalytic MOF films with different thicknesses (10–500 nm) showing the transition from kinetic control by the catalytic reaction to mixed kinetic control by the catalytic reaction and substrate diffusion when the film thickness is increased (see reference (122) for derivation of the current–potential expression). Under these conditions, the catalytic half-wave potential *E*_1/2_ shifts positive as the film thickness increases, which is a result of the influence of substrate diffusion within the film. b) After an optimal film thickness is reached, the current reaches a maximum value and no longer increases if the film is made thicker. The apparent shift in onset potential to more positive values would suggest a more efficient catalyst when an arbitrary increase in the film thickness in fact makes the catalyst less efficient. Other parameters used: *k*_cat_ = 100 s^–1^, *D*_S_ = 10^–9^ cm^2^ s^–1^, catalyst concentration = 0.1 M.

Unless onset potentials are derived from a kinetic model including reaction-diffusion, they should not be used to infer kinetic or mechanistic details. Since the current at the foot of any catalytic wave is an exponential function of the electrode potential,^137^ it even may be difficult to define the onset without referencing another distinct observable in the current–potential response (e.g., plateau current, catalytic half-wave potential, or standard potential of the catalyst). The point at which the catalytic current emerges from the background (capacitive) current will depend on, for example, noise levels and potential step size of the data.^138,139^ Other challenges are encountered when the diffusion of substrate is considered. For example, onset potentials appear to change as a function of film thickness in Figure 10 when substrate transport limitations interfere with kinetic control. In fact, they appear to be more and more positive as the film thickness is increased and all other parameters are held constant. One might be tempted to interpret an arbitrary increase in film thickness as a better catalyst, when in fact the opposite is true and the kinetics are under mixed control by substrate diffusion and the catalytic reaction.

### 2.3 Photochemically Driven Molecular Catalysis in MOFs

Many examples of light-driven MOF systems for water oxidation or reduction of protons or CO_2_ can be found in the literature. This is of course coupled to the long-term goal of direct solar energy conversion to a fuel, as an attractive alternative to indirect methods using renewable electricity or biomass as intermediates. From a viewpoint of fundamental understanding, the example of photoactivated enzymes and enzyme complexes shows the power of phototriggered reactions to elucidate complex reactions and identify intermediates. The detailed understanding of photosystems I and II is much greater compared to nonphotochemical energy converting enzymes such as hydrogenases and nitrogenases. Light-activation of enzymes has therefore become a popular strategy, by adding a PS to, for example, ribonucleotide reductase^140^ or hydrogenases,^141,142^ following the pioneering work on dye-labeled electron transfer proteins.^129^ One motivation for covalently linking dyes to proteins is to be able to phototrigger electron transfer reactions with light pulses and follow the photochemical reactions on short time scales with time-resolved spectroscopy. From the viewpoint of solar-driven generation of fuels, there is also the idea that a linked PS may lead to more efficient charge transfer to the enzyme and thus better photocatalytic properties than a bimolecular reaction with a PS in the same solution.

Photoactivation of MOFs has been motivated by similar interests as those above. However, the evaluation and understanding of the performance of these systems are challenging, as the photochemical reaction cycles involve many reaction steps, productive as well as nonproductive ones. Typically, the quantum yield for product formation is less than 10%, which means that other, competing reactions dominate the photochemistry. It is well-recognized that recombination of the photogenerated charges is the most common and general challenge to efficient artificial photosynthesis. Moreover, product formation is also limited by a rather slow photogeneration of redox equivalents, compared to electrochemical or dark chemical redox reactions. Thus, the observed rate is often not limited by the intrinsic ability of the catalyst to turn over. Photochemically driven molecular catalysis can therefore be even more difficult to analyze than electrochemically driven catalysis as discussed above. These points are illustrated and discussed in this section.

#### 2.3.1 Photochemically Driven Catalytic Cycles

**Figure 11:**
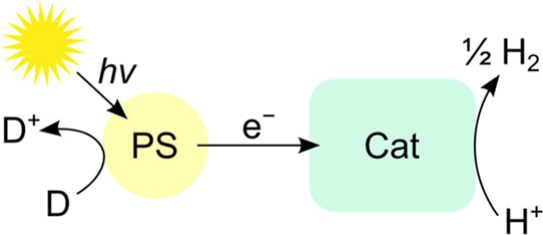
A common, simplified reaction scheme for photochemical proton reduction to H_2_, with a photosensitizer (PS), a catalyst (Cat), and a sacrificial donor (D). The simplicity of the scheme is deceptive, as it only shows a small number of the reaction steps that must be considered.

Photochemical production of fuels and water oxidation in homogeneous or heterogeneous systems is usually examined by measuring the amount of generated product as a function of time under constant light irradiation. The vast majority of systems examine only one half-reaction, instead of complete artificial photosynthesis, which is water oxidation coupled to reduction of, for example, protons or CO_2_ to a fuel. Most systems use an alternative sacrificial donor or acceptor instead. For simplicity, we will take photochemical H_2_ generation as example; see Figure 11 for a simplified reaction scheme of a type commonly used to illustrate the photocatalytic HER in a homogeneous solution or in MOFs.

The TOF is defined as the instantaneous rate of H_2_ production (in moles per time unit) per active site.^136^ The TON is usually reported as the maximum amount (mol) of H_2_ produced per mol catalyst at the time the experiment is stopped, often because the production of H_2_ has stopped or become very slow. In photochemical reactions, it is common that the reaction stops for other reasons than that the catalyst has degraded. Instead, the sensitizer may have degraded, the sacrificial agent may have been consumed, or the pH may have changed. In these cases, one can demonstrate the longevity of the catalyst by adding more sensitizer or sacrificial agent, or resetting the pH. Many authors therefore also report maximum TONs based on PS.

It is important to realize that the observed TOF in a photocatalytic experiment is not an intrinsic parameter of the catalyst, as was discussed earlier for electrocatalysis. What is evaluated is the entire system and not the catalyst itself. Usually, the observed TOF is limited by the rate of photon absorption and by charge recombination reactions that make reaction quantum yields much lower than unity. This is a consequence of the fact that the photochemical reaction of the OER coupled to the HER or the CO_2_RR is photosynthetic, which means it uses light energy to drive an uphill reaction, so that charge recombination to reform the starting reactants is a downhill process. This is obviously different from dark reaction methods such as electrocatalysis, which are intrinsically downhill, or photocatalytic conversion of wastewater to H_2_, which is energy neutral or slightly downhill. For the rest of this review, we will still use the term “photocatalytic” also for photosynthetic systems, although the differences between such systems are important.^143^ It is furthermore important to point out that even many “sacrificial” donors and acceptors (e.g., ascorbate) will lead to a large degree of recombination, as they are far from completely sacrificial under typical conditions.^144^ This typically makes the net delivery of redox equivalents to the catalyst rate-limiting, whereas the rate constants of the catalytic steps would often allow for much higher TOFs than those observed. The additional charge separation and recombination reactions make it more complicated to evaluate the potential performance of a catalysts by photochemical experiments.

**Figure 12:**
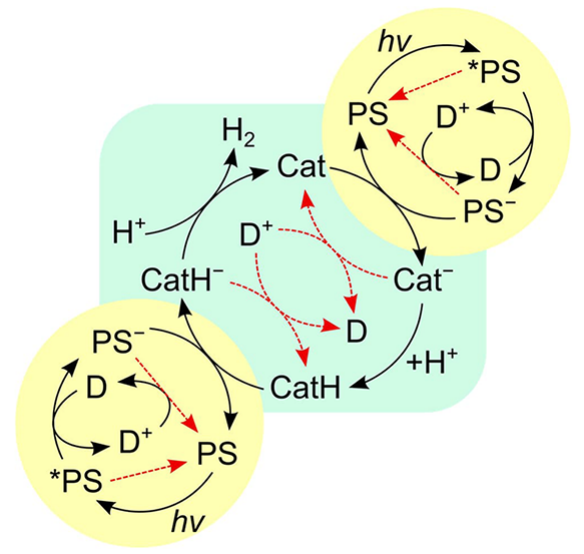
A more complete, but still idealized, reaction scheme for photochemical HER than in Figure 11. The primary reaction of the excited *PS is reductive quenching by the donor, and the catalyst is assumed to follow an ECEC mechanism. Productive reaction steps are shown with black arrows, and charge recombination reactions with red arrows.

To demonstrate these points with an example, let us expand the simplified reaction scheme for the photochemical HER in Figure 11 into a more complete, but still minimal scheme in Figure 12. This consists of a PS, a HER catalyst (Cat), and an electron donor (D) in the presence of protons; this could be a MOF system or a homogeneous solution. We assume that the excited *PS is reductively quenched by the D, and the reduced PS^–^ reduces the Cat. The Cat is assumed to undergo an ECEC mechanism, in which the second electron is provided by a second equivalent of PS^–^. If the D is completely sacrificial, so that every D^+^ decomposes without interfering with the other reactions, and all other side reactions can be ignored, the quantum yield Φ_*H*_2__ for the HER reaction is equal to 1:
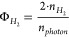
7where *n*_*H*_2__ and *n*_*photon*_ are the number of moles of H_2_ produced and photons absorbed, respectively (as at least two photons are needed to produce one H_2_, we multiply by two in the numerator). In this case the TOF will simply be equal to the rate of photon absorption, which contains no information on the catalyst rate constants. In practice, however, there are multiple charge recombination reactions and other side reactions, some of which are shown with red dashed arrows in Figure 12. Consequently, the majority of the published MOF studies show Φ_*H*_2__ ≪ 1, meaning that most of the absorbed photons and charges generated are not productive. Instead, they are involved in a range of energy-wasting charge recombination reactions—often not identified—and even irreversible redox chemistry that cause degradation of the PS, Cat, and/or MOF linkers. The observed TOF is then simply the rate of photon absorption (*Rate*_*hv*_) multiplied by the HER quantum yield:
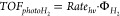
8None of these factors directly reflect the intrinsic reactions of the catalyst.

In the minimal scheme of Figure 12, the HER quantum yield is given by the products of the yields for photochemical charge generation, electron transfer to catalyst, and catalyst turnover. Each of these processes competes with charge recombination, as shown in red dashed arrows, and the resulting yields are strongly dependent on the competing reactions. Moreover, the catalyst cycles through several intermediate states, each with its own reactivity toward PS^–^ and recombination with D^+^, which makes the exact kinetics complex. Any change of reaction conditions, or modification of the catalyst, may affect several productive and competing steps. It is therefore not straightforward to determine how the rate constants of the actual catalytic steps have changed. In photocatalytic MOFs, the situation can be even more complicated due to transport limitations and resulting concentration gradients. These may cause the quantum yield Φ_*H*_2__ to have a spatial dependence within the MOF particle/film. Finally, it should also be pointed out that several MOFs exhibit photochemical instability. Such processes will obscure kinetic information, in particular on longer time scale experiments and under high-energy light irradiation.^145^

In a large majority of cases, for both homogeneous and MOF-based systems, the observed TOF is not limited by the catalyst’s capacity to turn over. Instead, it is limited by the rate of light absorption and a low quantum yield, and consequently the slow rate of electron delivery to the catalyst.^146^ An efficient PS is excited only on the order of once every second under full sunlight, and most catalysts can turn over faster than that if electrons and protons are provided. To match the rate of light absorption with that for the intrinsic turnover capacity of the catalyst, multiple PS molecules per catalyst are needed working as a light-harvesting antenna. As one example, Reisner and co-workers^147^ have used TiO_2_ particles, each with a large number of attached PS molecules and only few Cat molecules, and found that the photocatalysis rate was independent of light intensity. In most cases in the literature, however, the ratio PS:Cat is not that large. MOF systems on the other hand offer the possibility to implement an antenna function with multiple PS per catalyst, as will be discussed in section 2.3.3.

It is clear from eq 8 that the experimental rate cannot be compared to a dark kinetic experiment when all reactants are added at time *t* = 0 and the reaction progress is followed over time. In the photochemical experiment, one reactant is generated only slowly, via light absorption, and the reaction can never be faster than the rate of photon absorption. Whether a reaction is light-limited can experimentally be verified through varying the light intensity by, for example, inserting a neutral density filter. If the TOF changes in proportion to the light intensity, the TOF is light-limited. This straightforward test is quite informative and should be routinely reported, yet it is only rarely done. In one case, photochemical water oxidation was compared in homogeneous and heterogeneous systems by Bonnet and co-workers,^148^ who carefully examined the dependence of the TOF on the concentration of all reactants as well as on the light intensity. With [Ru(bpy)_3_]^2+^ (bpy = 2,2′-bipyridine) as PS, [Ru(bda)(isoq)_2_] (H_2_bda = 2,2′-bipyridine-6,6′-dicarboxylic acid; isoq = isoquinoline) as a catalyst, and persulfate as a donor, the TOF increased linearly with increasing light intensity until ∼1 sun irradiation was reached and the TOF maximized. Even if the reaction was no longer light-limited, the limiting step was still not catalytic turnover. Instead, they concluded that electron transfer from the catalyst to the oxidized PS limited the reaction. Only when using slower catalysts in the homogeneous system could they observe rate limitations from slow catalyst turnover. It should be noted that even in this case, the obtained TOF was lower than the intrinsic rate of the catalyst. The catalyst sluggishness instead reduced the quantum yield Φ_*H*_2__ because competing, nonproductive reactions had time to occur to a greater extent. Interestingly, when they used a liposomal system instead, in which amphiphilic analogues of their PS and Cat were bound to liposomes, they found that photogeneration of PS^+^ was limiting instead.^149^

To illustrate this argument, we can assume that the limiting step of a HER catalyst is the final protonation of the hydride state, that under the experimental conditions has a rate constant of 10^3^ s^–1^. This catalyst then has the potential to reach up to a *TOF* = 10^3^ s^–1^. But if the hydride state at the same time recombines with D^+^ with a pseudo-first-order constant of 10^5^ s^–1^ under certain conditions, Φ_*H*_2__ will be low and a *TOF* ≪ 10^3^ s^–1^ will be observed. This may then be interpreted as a slow catalyst, but the problem is rather the rapid recombination reaction. The TOF would only be equal to 10^3^ s^–1^ under conditions where the rate of photon absorption multiplied by Φ_*H*_2__ (eq 8) is equal to 10^3^ s^–1^ per catalyst, which is unlikely for most systems studied to date. The effects of synthetic improvement of the intrinsic catalyst rate may then be masked by effects on the charge recombination.

These reports of homogeneous photoinduced catalysis described above are examples that underline the importance of varying the experimental conditions to elucidate qualitative kinetic features. However, a quantitative relationship between reported initial TOFs or maximal TONs and the parameters of the system (light absorption, catalytic rate constants, back electron transfer, concentration of sacrificial donor and PS, and so forth) is required for benchmarking and reliably comparing photocatalytic systems. Costentin et al.^146^ recently reported a general analytical kinetic model for such reactions. They derive an analytical expression for the time evolution of the TON that takes the form of a relatively simple third-order polynomial. This is a function of several dimensionless parameters, which represent the competition between various steps in the mechanism. It was identified that in all situations the kinetic information that can be obtained depends on the rate of light absorption. Additionally, this analytical derivation demonstrated that under certain conditions, the rate of the catalytic reaction cannot be retrieved from the time evolution of the TON, as this tends asymptotically to a function that only depends on quenching, back electron transfer, and reduction of the catalyst, in addition to light absorption. An important realization from this analysis was that the TON increases nonlinearly with time due to accumulation of the oxidized donor and the resulting increasing rate of back electron transfer. As a consequence, even within relatively short irradiation times the concept of an initial TOF is no longer meaningful. Finally, by extending this general kinetic model to included deactivation of the catalyst or PS as well as a two-electron stoichiometry, the authors were able to analyze experimental data of light-driven HER by a cobalt tetraazamacrocyclic catalyst in water with ascorbate as the sacrificial donor and either [Ru(bpy)_3_]^2+^ or a triazatriangulenium organic dye as the PS. With the addition of the diffusion of the various reaction components, a general kinetic model such as this one that allows for the derivation of the TON versus time expression could be used to understand photoinduced processes in MOFs with molecular catalysts.

It is clear that also MOF-based systems may operate under quite different limiting conditions. A similar analysis as that for liposomes above, with a systematic variation of concentrations and light intensities, has to the best of our knowledge not been performed for MOFs. A few interesting cases exist where the rate of product formation was compared for different catalyst loadings, leading to mechanistic insights, as discussed below (see section 2.3.5 and section 3).

The above discussion may seem discouraging for attempts at gaining mechanistic information on a catalyst through photochemical methods, but it is important to appreciate that a catalyst is always tested as a part of a system. Under electrochemical conditions interfacial electron transfer is ideally assumed to be rapid and the chemical steps limiting. Even in the many cases where this assumption is correct, the same catalyst under dark chemical reduction may be limited by bimolecular electron transfer from the reductant. Moreover, mass transport may limit the overall catalytic process, as discussed in section 2.2.1 for chemical and electrochemical catalysis. As exemplified above, however, more detailed information can be obtained also with photochemical catalysis, provided that necessary kinetic investigations are conducted where the TOF is measured as a function of different reactant concentrations as well as light intensity. In section 2.3.5, we outline some important qualitative differences in photochemical performance as a function of catalyst concentration and light intensity, to illustrate the possibilities to distinguish between cases where different factors are limiting the overall reaction, which is obviously helpful for improved understanding. Moreover, with time-resolved spectroscopic methods, using pulsed excitation light, catalyst intermediates and their reaction steps can be identified and directly followed in a way that may be challenging with other experimental approaches.

#### 2.3.2 Time-Resolved Spectroscopy: Possibilities and Challenges

Time-resolved spectroscopy with pulsed laser excitation is very useful to determine the rates of photochemical reactions. Time-resolved fluorescence can give high-quality kinetic data for the decay of the initial excited state. Often the assignment of the fluorescent state is quite clear, but in more complex systems, such as MOFs, this may not be the case. Typical complications may be electronic interactions between nearby chromophores leading to excimers, exciplexes or larger aggregates. But even in the absence of such complications, the interpretation of the fluorescence decay is not straightforward. In the MOF literature, the quenching of fluorescence lifetime and/or intensity when the dye is introduced in the MOF is usually assumed to reflect the desired electron transfer reaction to or from the dye. However, many other mechanisms besides electron transfer exist that can quench fluorescence, so fluorescence quenching is insufficient evidence for productive charge separation. These mechanisms include energy transfer to either fluorescent or “dark” states, enhanced intersystem-crossing (for example by the heavy-atom effect) to form a nonfluorescent dye triplet state, paramagnetic quenching by radicals or transition metals, and formation of different types of nonfluorescent aggregates. Therefore, while fluorescence quenching is great for the acquisition of high-quality kinetic data, additional evidence is needed to safely assign the kinetics to charge separation.

Transient absorption spectroscopy can be used to detect the electron transfer products. In many cases where this has been used as evidence for charge separation in MOFs, the transient absorption spectra have only revealed the ground state bleach, which decay without formation of other detectable products. Since the species of the proposed charge-separated states in these cases actually have clear absorption characteristics, the data instead prove that productive charge separation did not occur, in contrast to the conclusions of these papers. In some cases, the authors used a so-called state-filling model, as used for semiconductors, to explain why the charge-separated state would not show a signal. Briefly, the model says that the transient ground state bleach is because electrons fill conduction-band states, but that valence-band holes give negligible contribution to the transient signal. However, this is not a valid model for an insulating MOF with local excited states. The absorption and fluorescence bands of the free dyes in solution are still clearly resolved in the MOF in many cases, for example the Soret and Q-bands of porphyrin linkers. This clearly shows the molecular nature of the ground and excited states in the MOF.^150,151^ Therefore, the charge-separated state in such MOFs, where the porphyrin unit is oxidized, would have shown a strong porphyrin ground state bleach and clear positive signals from the resulting porphyrin radical. The absence of such signals is evidence against—and not for—charge separation.

It is also important to use the proper reference system when assessing MOFs with transient spectroscopy. Insertion of a transition-metal ion in porphyrins or phthalocyanines introduces low-lying charge-transfer and metal-centered states that typically lead to accelerated radiation-less decay to the ground state (<1 ns). This is true for popular catalyst metal ions such as Ni^II^, Co^II^, and Fe^III^. That the same thing happens in a MOF should not come as a surprise.^150,152,153^ The porphyrin excited state, and its fluorescence, will be quenched relative to the free base, but this should not be taken as evidence for productive electron transfer to the putative catalytic metal in the MOF. It is just the intrinsic photophysics of the metal porphyrin.

Even in cases where quenching of the *PS is due to charge separation, covalently linked dye–acceptor or dye–donor dyads typically show rapid recombination of the charge-separated species. Long-lived charge separation usually requires the addition of further acceptor or donor molecules to form triads, tetrads and so forth.^154−156^ The rate constant for recombination is often faster than that for charge separation, so that no or very little of the charge-separated intermediate is seen. This is a very likely scenario in MOFs, too, and could explain the spectroscopic observations. It is important to realize that if the yield of long-lived charge-separated states is very small, this may not be observed in the transient absorption experiment. If, for example, 1% of the initially separated charges jump to neighboring sites and escape recombination, this can be challenging to detect. Nevertheless, it can still suffice to drive a photocatalytic reaction with ∼1% quantum yield, and many reports show quantum yields in that range or much lower. Thus, the absence of a detectable charge-separated state in a transient absorption experiment is not necessarily in disagreement with detection of photoproducts in the photocatalytic experiments. What the transient experiments then show is that the excited state is quenched and the yield of long-lived charge separation is small.

Some examples where long-lived charge separation in MOFs has been observed by transient spectroscopy include [Ru(bpydc)(bpy)_2_]^2+^-decorated (bpydc = 2,2′-bipyridine-5,5′-dicarboxylate) **UiO-67**.^157,158^ Santiago-Portillo et al.^158^ showed relatively long-lived products and by adding electron or hole scavengers they could attribute the signals to a charge-separated state with electrons on the zirconia cluster and holes on the bipyridine ligand. It is interesting that zirconia clusters are widely taken as electron acceptors in the MOF field, whereas bulk zirconia has a very high lying conduction band, and zirconia nanoparticles are used as noninjecting reference substrates in the dye-sensitized solar cell field, with the same type of carboxylate linkers.^159^ We speculate that there may be different defect sites on the smaller zirconia clusters of MOFs. Lin et al.^157^ used both the [Ru(bpydc)(bpy)_2_]^2+^-sensitizer and a [Ru(terpy)(bpydc)Cl]^+^ catalyst (terpy = 2,2′:6′,2″-terpyridine) as linkers and could detect rapid (21 ns) generation of oxidized and/or excited catalyst. From the slow decay of their transient, with components up to ca. 280 μs, it was clear that this decay was due to the oxidized catalyst.

Time-resolved laser spectroscopy initiates light reactions with a laser pulse that excites a minor part of the sample and triggers charge-separation reactions. The experiment is typically repeated several times to obtain data of good quality. Thus, the sample has to be regenerated by charge recombination between each laser pulse. If a sacrificial donor or acceptor is used instead, the sample will evolve to form more and more of catalyst in reduced (or oxidized for OER) states with increasing number of flashes, and the transient absorption results will change. With nanosecond flash photolysis one may get useful kinetic data in a single flash and thus track the changes in kinetics as the system evolves, but interpretation becomes increasingly complex. One usually has a mixture of catalyst states, with optical absorption spectra that are often quite indistinct so that their concentrations cannot easily be determined. This has been possible in some cases with some notable examples being TiO_2_ particles with molecular HER catalysts.^160,161^ In these cases, the different redox states of the catalyst showed sufficiently distinct absorption spectra for quantification. Another approach to follow later steps in the photoredox cycle is to prereduce (or preoxidize) the sample electrochemically, and do in situ laser spectroscopy, to be able to measure the reactions of a well-defined state. Finally, pump–pump–probe experiments have been performed, where a second laser pulse is applied at a selected time after the first, to achieve accumulative charge separation—i.e., successive photoinduced charge separation after sequential photon absorptions^162^—on the two-electron level, in nonsacrificial systems.^163−168^ Accumulative electron transfer has even proven possible in molecular systems under light levels corresponding to solar irradiation.^169^ These methods have yet to be applied to study solar fuels-forming photocatalytic MOFs.

#### 2.3.3 Coimmobilizing PS and Catalyst in MOFs

Many molecular dyads and larger assemblies, including MOFs, have been prepared and studied where both PS and Cat are immobilized at close distance, thus designing the reactive coordination sphere of the catalysts. One motivation for immobilizing the PS and the catalyst is to perform fundamental studies, where the linkage removes the need for diffusional steps in the reaction that may limit and obscure the kinetics of the PS–catalyst electron transfer itself. A more pragmatic motivation is the desire to increase photocatalytic performance, exerting better control over the electron transfer reactions with linked reactants, and to increase the electron transfer rate. It is true that a linked PS–catalyst system will typically result in faster electron transfer to or from the catalyst than for the corresponding bimolecular reaction, especially when low concentrations of reactants are used. However, this does not necessarily lead to faster overall catalysis (higher TOF), because this electron transfer step may not be rate-limiting. As discussed above, the observed rate of catalysis is given by the rate of photon absorption multiplied by the photoproduct quantum yield. Photon absorption is not improved by linking the reactants, and the product quantum yield is only favored by a fast electron transfer to or from excited *PS if this reaction competes with some other reaction, such as spontaneous decay of the *PS to the ground state. With an initial reductive quenching of *PS by a donor as an example, recombination of PS^–^ with D^+^ typically leads to large losses of efficiency, unless the sacrificial reaction of the donor is ultrafast. A fast electron transfer from PS^–^ to Cat can then improve the system performance, but only if recombination of D^+^ with the reduced catalyst is slower than recombination of D^+^ with PS^–^. Thus, while MOFs with a relatively short PS–catalyst distance have often been assumed to give a rapid initial electron transfer, this is far from certain to lead to efficient product formation.

If the primary reaction with excited PS* is with the catalyst instead of with a donor—such as in many MOFs where the PS is the linker and the Cat is the node—the electron transfer products (PS^+^ and Cat^–^) will typically recombine much faster than in a bimolecular system. This may therefore lead to much lower efficiency for the linked system than for the corresponding bimolecular system. In bimolecular, photoinduced electron transfer, the charge-separated products will have a lifetime >1 ms under one sun irradiation before they recombine by diffusional encounters. In linked systems, the charges typically recombine much faster than that. While photosystem II, with its optimized, well-ordered array of electron transfer components, achieves a charge-separation yield of about 100% and a lifetime of ∼1 s,^170^ such a performance has proven extraordinary difficult to achieve in dyads, triads, and so forth.^154−156^ Lifetimes and yields approaching these values have only been reached in heterogeneous molecular materials (e.g., organic and dye-sensitized solar cells). In this context, MOFs provide an interesting possibility for a molecularly designed and well-arranged 3D material to realize efficient charge separation and transport, coupled to catalysis. In section 2.3.4, we will discuss different topological arrangements of PS and Cat in MOFs.

A major advantage with linking the components in MOFs is that a high local concentration of PS around each catalyst can be achieved. A similarly high concentration in homogeneous solution would lead to extreme absorbance values, such that light would not reach far into the solution/suspension. It is also likely to lead to uncontrolled aggregation and precipitation. With a high concentration of PS on a MOF, they may act as antennas for the catalyst, so that the rate of photon absorption can match that of the intrinsic capacity of the catalyst to turn over. Thus, it is an advantage to have a high PS:Cat ratio, so that the catalyst molecules do not compete for photogenerated redox equivalents. An increase in catalyst concentration can lead to not just a lower TOF (per catalyst) but even to a lower rate of product formation, because the slower rate of turnover of each catalyst means a great probability that the redox equivalents are lost in competing reactions.

A high PS density in MOFs can enable efficient PS-to-PS energy transfer, and following a primary quenching with a donor/acceptor also hopping of redox equivalents can be facilitated. Rapid and efficient excitation energy transfer has been demonstrated in MOFs with either porphyrin or transition-metal complex PSs.^171,172^ The amplified photoluminescence quenching, where a small number of quenchers are sufficient to quench a large part of the MOF’s fluorescence, proves that efficient light-harvesting coupled to charge-separation centers is possible. Even in photocatalytic MOFs, light-harvesting energy transfer coupled to the CO_2_RR has been reported, and reduced intermediates of the [Re(bpydc)(CO)_3_Cl] catalyst were detected by in situ FTIR spectroscopy.^173^

For organic molecules,^174^ and recently a transition-metal complex,^175^ symmetry-breaking charge separation has been demonstrated, in which electron transfer between one *PS and one neighboring PS produces one PS^+^ and one PS^–^. This effect can be used to obtain efficient charge separation in materials with high density of PS, and principles and design criteria have been discussed in recent reviews.^176,177^ To the best of our knowledge, this possibility remains to be explored in MOFs.

A high density of PS can also lead to energy-wasting reactions, however, such as exciton–exciton annihilation (at high photon fluxes), and concentration quenching of a single *PS by neighboring ground state PS molecules.^178−180^ The exact mechanism behind the concentration quenching is often not identified, but molecule–molecule interactions can lead to excitonic states with short lifetimes.

**Figure 13:**
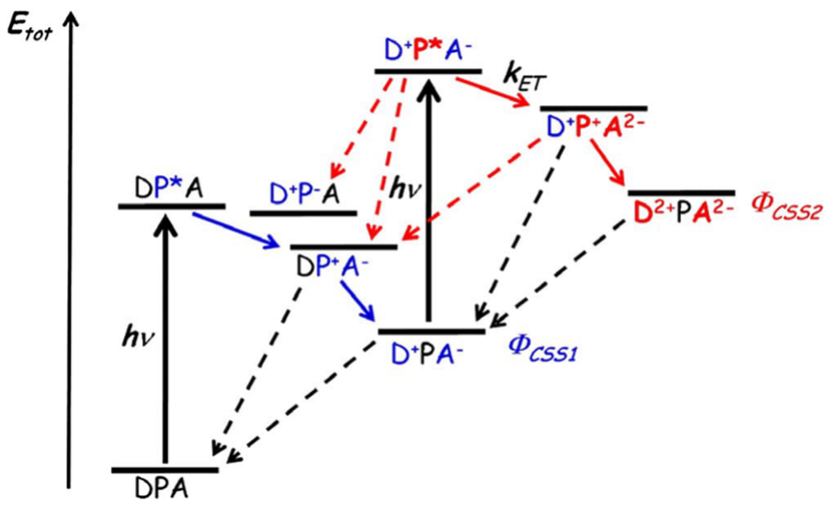
Reaction scheme for accumulative charge separation in a donor–photosensitizer–acceptor (DPA) triad undergoing successive absorption of two photons. Solid arrows represent productive reactions after the first (blue) and second (red) photon absorption; dashed arrows represent charge recombination reactions, where the reverse electron transfer reactions are shown in red. Reproduced with permission from reference (162). Copyright 2015 American Chemical Society.

As pointed out above, incorporation of a dye and a catalyst at short distance in the same MOF typically increases the rates of both charge separation and recombination. Moreover, additional difficulties and competing reactions exist when the catalyst has to cycle through different oxidation states, as has been outlined by Hammarström.^162^ Taking a general donor–photosensitizer–acceptor triad as an example (Figure 13), the initial photoinduced charge separation has to overcome the usual recombination reactions. When the second photon is absorbed, there are not only the corresponding recombination reactions (black, dashed arrows) but there is a large driving force also for reverse electron transfer (red, dashed arrows) where the excited dye sends an electron back to the donor that was oxidized by the first photon, and/or take an electron from the reduced acceptor. This means that the second photon absorption reverses the result of the first photon absorption. In addition, if the original acceptor/donor states are closed shell species, their resulting reduced/oxidized states are typically paramagnetic and have low lying electronically excited states, which may quench the dye in reactions that are not productive for photochemical charge separation and catalysis. These competing reactions are important to consider in the design of a photocatalytic system, especially for complete water splitting or the CO_2_RR coupled to the OER. In sacrificial half-reactions, the competing reactions of the dye excited state can often be counteracted through a sufficiently rapid initial quenching by the irreversible donor/acceptor, which then blocks all recombination reactions. On the other hand, the ultimate goal should be a complete artificial photosynthetic system, and then one cannot rely on this strategy. The progress on sacrificial half-reactions may therefore hide the difficulties involved in making a complete system.

#### 2.3.4 Design of MOFs for Photochemical Charge Separation and Catalysis

**Figure 14:**
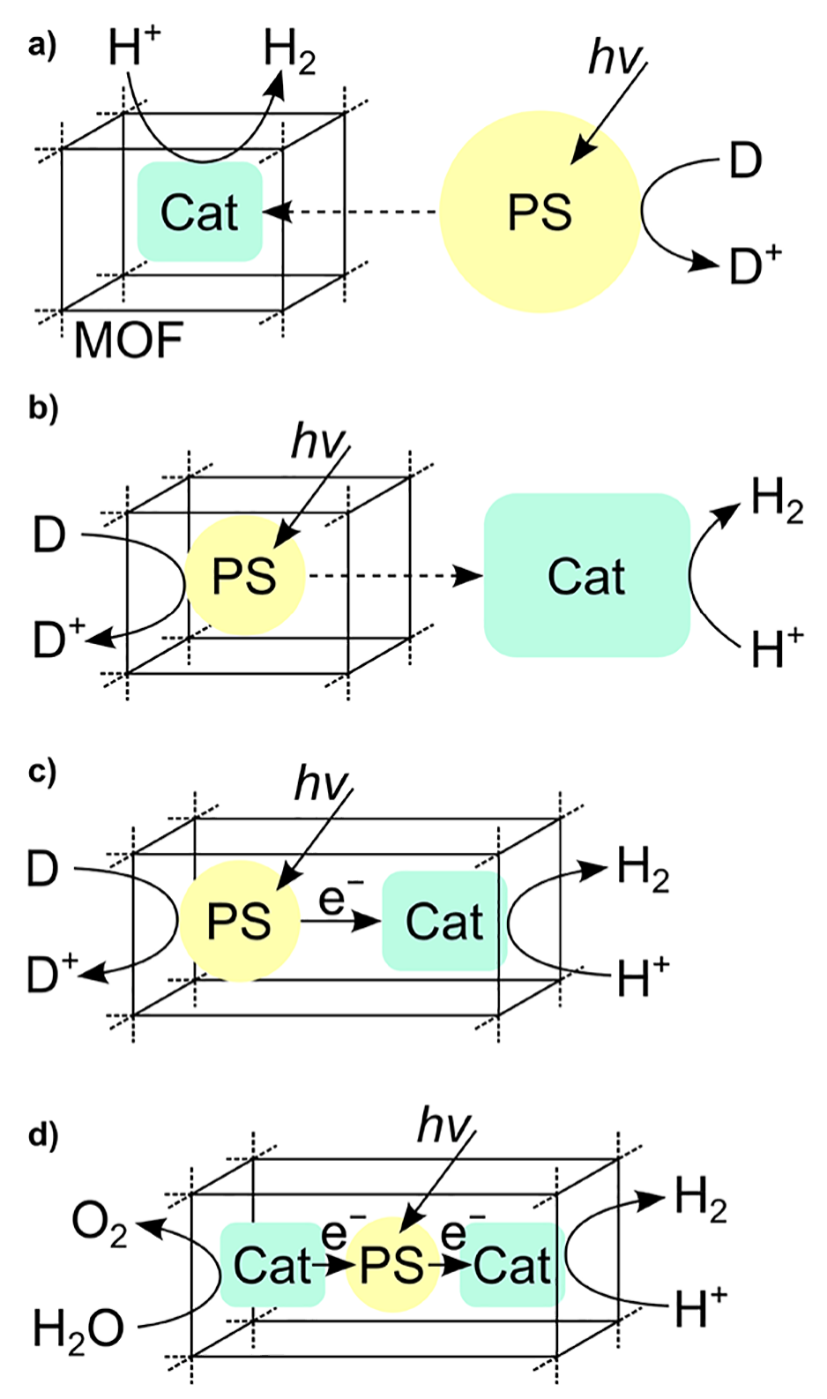
Different arrangements of a photosensitizer (PS) and/or a catalyst (Cat) within MOFs. a) Only a Cat within the MOF in combination with a PS and a sacrificial donor (D) outside the matrix. b) Only a PS within the MOF in combination with a D. In a) and b) electron transfer to the catalyst may occur by charge hopping in the MOF or a diffusional redox relay (dashed arrow). c) Both a Cat and a PS within the MOF matrix located but still in combination with a D. d) An arrangement where a PS is combined with two different Cats within a MOF to catalyze water splitting without the use of any sacrificial agents.

The design of photoactive MOFs for solar fuels generation can be based on different topological arrangements, with either the PS or the catalyst (HER is the example in Figure 14) residing in the MOF and the other in solution or on the surface (Figure 14a, b), or with both components inside the MOF (Figure 14c, d).

With only the catalyst in the MOF (Figure 14a), the PS is often too large to diffuse into the pores. Therefore, only catalysts at the outer MOF layer will react, unless electron transport over the dimension of the MOF is sufficiently rapid to kinetically compete with turnover. This transport can be through electron hopping through the MOF structure, mediated by the MOF backbone and/or through hopping between catalyst sites, or through an electron relay that diffuses through the pores. These transport properties may limit the overall reaction, similar to the dark chemical case discussed above.^98^ With an external PS and a 100% sacrificial agent, the difference to the dark oxidation case is only that the steady-state concentration of reductant/oxidant is always small, but is continuously generated. This can still lead to transport limitations and a case where mainly the outer catalyst layers are active, but if their intrinsic activity is sufficiently high, one would still only see that the TOF is limited by light generation of oxidizing equivalents. Reports of such systems are discussed in section 3.1.

Systems where only the PS resides in the MOF (Figure 14b) typically contain nonmolecular catalysts on the MOF surface, and these are outside the scope of this review. Their electron transport is limited by analogous limitations as case a) above, but several MOFs show efficient energy transfer between sensitizers that may transport excitons to the MOF interface. Here charge separation and catalysis can occur efficiently with comparatively small transport limitations (see the following for some examples of energy transfer in MOFs^171−173,181,182^).

Many systems exist with both a PS and a catalyst in the MOF (Figure 14c). They may be introduced at different concentrations in a native MOF structure, as linkers or inside the pores. In other cases, they constitute the entire MOF, often with the linker being the PS and the nodes as the proposed catalyst for either the HER/CO_2_RR or the OER. Electron transport between the sensitizer and the catalyst may occur via hopping through the MOF or via a diffusional relay. In many cases with a high density of PS and catalyst sites, however, charge separation is believed to occur directly between the sensitizer and a nearby catalyst, either through electron transfer from the excited state or through direct excitation into a ligand-to-metal charge transfer band. Typical examples of the latter are MOFs with *meso*-4-carboxyphenyl porphyrin ligands and zirconia nodes. Nevertheless, transport of the sacrificial donor/acceptor and the substrate, as well as of the resulting products, are still necessary. This does not exclude that one could run out of donor/acceptor when irradiating for several hours. Even with modest catalyst bulk concentrations and TONs, the quantum yield is often low so that a large amount of donor/acceptor has been consumed.

The short distance between the sensitizer linker and the catalyst node is favorable not only for rapid charge separation but also for rapid charge recombination. It is also important to remember that at least two charge-separation events are needed to complete a catalytic cycle. This design therefore relies on a rapid regeneration of the sensitizer, through electron transfer to or from electron relays or neighboring sites of the MOF, to avoid recombination and also allow for absorption of a second photon. Even if this is achieved, it is important to realize that the catalyst is now in a different redox state than in the dark, and may quench the sensitizer in other ways than by productive charge separation; this was discussed in section 2.3.3.

An ideal system (Figure 14d) includes both the OER and the HER or the CO_2_RR in the same MOF, which is complete artificial photosynthesis, and is thus independent of sacrificial donors or acceptors. This requires that electron transport in the favorable direction is rapid enough, compared to charge recombination, so that a high product yield is obtained. There are robust guidelines, based on electron transfer theory, how to control electron transfer in terms of energetic factors and electronic coupling (super exchange and hopping transport).^183−185^ However, from decades of work on artificial photosynthesis, it is clear that this is not easy to realize. Nevertheless, several reports of complete artificial photosynthesis in MOFs have appeared in recent years.^150,186−191^ The total product formation has in most cases been 100 μmol g^–1^ or less, which, assuming a reasonable molar mass of 2000 g mol^–1^, would correspond to 0.2 turnovers per SBU, or, more specifically, per molecular formula. With such low yields, it is particularly important to verify that the products really originate from water splitting and CO_2_ reduction, and not from degradation of the MOF or other components. Therefore, most authors report isotopically labeled ^13^CO_2_ and H_2_^18^O to prove the origin of the detected products. Even if the yields are low, the fact that some product can be detected is interesting, and by comparison there are no homogeneous (molecular) photochemical systems that have been unambiguously shown to perform complete artificial photosynthesis. Instead, semiconductor systems, colloidal systems as well as electrodes, with or without molecular catalysts, have demonstrated complete artificial photosynthesis, in some cases with quite impressive yields and energy conversion efficiencies.^192,193^ Classic photoelectrochemical systems with semiconductor/liquid junctions may show quite good performance. The systems with highest efficiencies rely on photoinduced charge separation in well-developed photovoltaic materials, which are isolated from the solution and coupled to catalysts via an ohmic contact layer.^194−196^ As discussed above, most MOFs used in this field are insulators and not semiconductors, and charge transport would occur by electron hopping or carrier diffusion. It is interesting to speculate why any charges at all escape recombination to instead form products. It is possible that the heterogeneous nature of the system leads to strong kinetic heterogeneity of charge recombination and that some low-probability events can actually accumulate redox equivalents on one catalyst and achieve turnover before recombination occurs. There will always be defects in a MOF that may act as charge traps, which may be favorable for photocatalysis under the right conditions. A further difference to homogeneous systems is that the local concentrations of sensitizers and catalysts are very high in MOFs. When at least one linker per pore is a sensitizer, the local concentration is in the order of 1 M. This is several orders of magnitude larger than in a typical homogeneous experiment and will lead to a higher rate of charge generation per unit volume in the MOF, which should favor multielectron catalysis.

To promote charge separation in MOFs, it would be interesting to introduce redox gradients in MOFs, to retard charge recombination. One particularly interesting report on complete artificial photosynthesis in MOFs used separate MOFs for the HER and the OER, respectively, which were coupled via electron relays.^189^ This system showed an apparent quantum yield for complete water splitting of 1.5%. It was carefully designed to improve charge separation and counteract recombination by spatially separating them in different regions of a liposome solution.

#### 2.3.5 Analysis of Reactions by Variation of the Catalyst Concentration and Light Intensity

**Figure 15:**
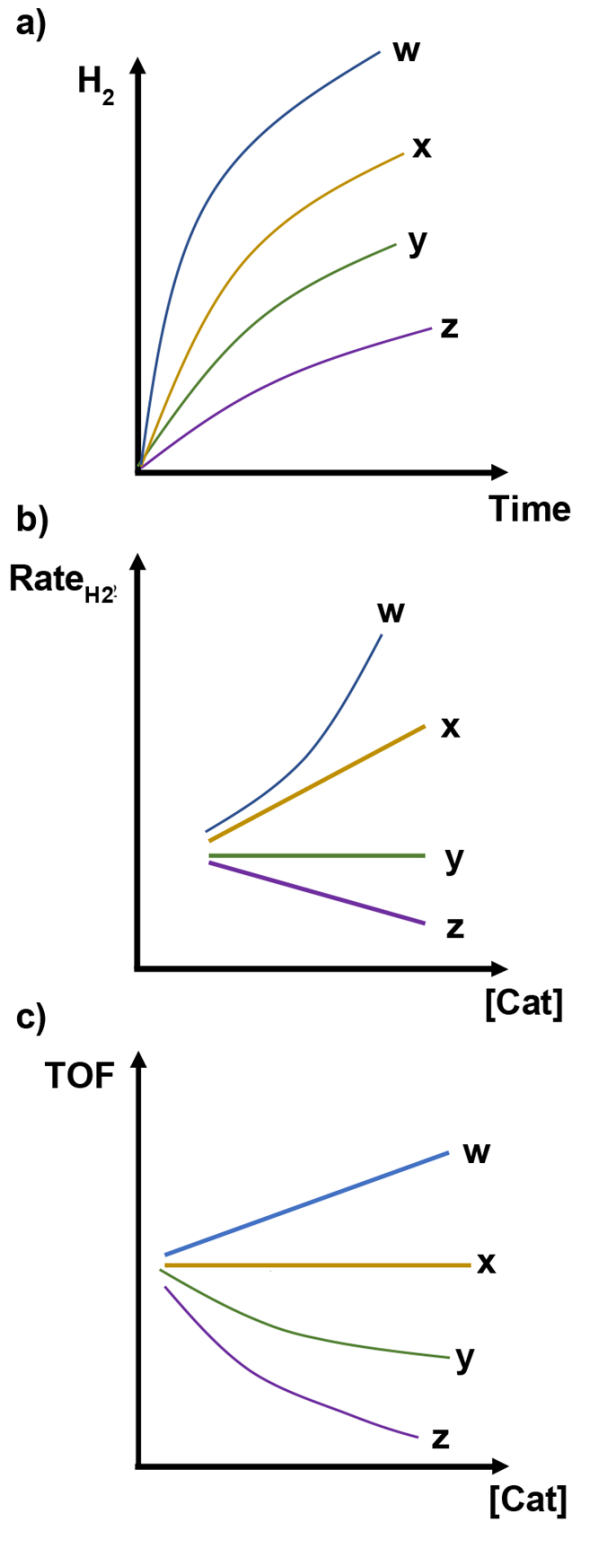
Qualitative illustration of the HER for four hypothetical MOF systems that show different TOFs and rate dependencies on [Cat]. a) Rate of H_2_ production as a function of time at a fixed [Cat], b) maximum (initial) rate of H_2_ production as a function of [Cat] and c) TOF as a function of [Cat] (data corresponding of panel b, with TOF = Rate/[Cat]). Note that trends shown may hold in a limited range of [Cat] values investigated, but, for example, the rate must go to zero as [Cat] goes to zero.

Mechanistic insight may be gained by varying not only the light intensity but also the catalyst concentration in a MOF. This is similar to our point made in section 2.1 in that it is imperative to vary reaction parameters to get viable information in electrocatalytic experiments. Figure 15a illustrates a thought experiment of photochemical H_2_ production as a function of irradiation time, for four different, hypothetical MOF systems **w**, **x**, **y**, and **z**. The four MOFs show different initial rates, which means different quantum yields, and it is of interest to understand the origin of this difference. With only the results of Figure 15a at hand, we can only speculate based on our ideas of how the MOFs are designed, but not determine the origin of the behavior with any certainty. Further experiments with variations of conditions could be helpful in that regard, such as variation of catalyst concentration and light intensity.

In Figure 15b and c the maximum rates and the TOFs, respectively, of the same MOFs are plotted as a function of catalyst concentration. The MOFs show qualitatively different behavior. MOF **w** (blue line) shows a strong increase in rate with increasing [Cat], and even the TOF increases. This may be explained by cooperative catalysis for a case where the actual catalytic steps are rate-limiting, but it may also be caused by increased MOF conductivity by hopping via catalyst sites. For cooperative catalysis, this behavior would disappear at lower light intensities when the catalytic steps are no longer limiting, while limitation by electron hopping would still remain. Thus, a combination of variations of [Cat] and light intensity could offer mechanistic insight. In one example of cooperative catalysis, Choi et al.^197^ measured photochemical CO_2_ reduction in **UiO-67** with some linkers substituted for the photocatalyst [Re(bpydc)(CO)_3_Cl]. When varying the photocatalyst loading they found that three Re complexes per unit cell gave the highest amount of CO product, which led the authors to propose cooperative catalysis, although light-intensity variations were not reported. Infrared spectroscopy gave support for interaction between the Re complexes at higher loading levels.

MOF **x** (yellow line) shows a linear dependence of rate on [Cat] and a constant TOF, which suggests that the catalysts are not competing for redox equivalents. Roy et al.^198^ studied photochemical H_2_ production on **MIL-101(Cr)** with Fe_2_(1,2-dithiolate)(CO)_6_ as catalyst linked via amide bonds. They found that the rate of the HER was proportional to the catalyst concentration, which they proposed as evidence that the reaction was not limited by the photogeneration of reducing equivalents. This requires a high steady-state concentration of electrons and/or a slow catalyst, and one would expect the quantum yield to be rather high at high [Cat]. An alternative interpretation is instead that the reaction is light-limited. The increase in rate with [Cat] would then be because of a higher rate of electron transfer from PS^–^ to Cat, which competes more favorably with PS^–^ recombination. Light-intensity variations would be able to distinguish between these two scenarios also in this case.

For MOF **y** (green line), the rate is independent of [Cat], and different possible scenarios exist. These include the case of a MOF where the reaction is limited by the light generation of reducing equivalents, but where these equivalents are long-lived once they are formed, for example due to an efficient sacrificial donor. Electron transport to the catalysts would in this case not be limiting, and the [Cat] would therefore not be important. The TOF would then be proportional to the light intensity.

MOF **z** (purple line), in contrast, shows a decrease in rate with increasing [Cat], and a strong decrease in TOF. This is typical for a case where photogeneration of reducing equivalents is slow and the catalysts are competing for electrons. When they do not get the second electron fast enough, it has a higher risk of recombination (or other side reactions). Therefore, a higher [Cat] (beyond a certain optimum) has a negative effect on the overall reaction rate. This situation has been shown in homogeneous systems.^199,200^ If the catalyst is incorporated in a MOF, its reduction kinetics may be slower because of transport limitations, but also recombination with D^+^ may be slowed down, so that further losses are avoided. This case has been suggested when comparing a homogeneous Fe_2_(μ-Cl_2_bdt)(CO)_6_ (μ-Cl_2_bdt = 1,4-dichloro-2,3-benzenedithiolato) catalyst with a hydrogenase enzyme.^201^ Case **z** in Figure 15 would definitely show light-dependent rates, as the rate of electron generation is increased, and the dependence may even be stronger than a linear one.

The above scenarios are by no means exhaustive, and other conditions may give rise to a similar behavior as the examples given. Yet, they illustrate what insights a variation of parameters may give, here emphasizing the effects of varying the catalyst concentration and the light intensity. Variations of [PS] or [substrate] and kinetic modeling will provide additional information and insights. Because of the complexity of a complete photochemical reaction scheme, a detailed kinetic model may not be useful. Nevertheless, important insights may be gained by a simplified treatment and identification of the limiting step(s) of photocatalysis.

## 3 MOF–Catalyst Interactions

MOFs can exert a variety of effects on the electronic properties, reactivities, and catalytic performances of molecular catalysts that they host. For example, the MOF matrix can lead to a structural stabilization of the catalyst, thereby leading to higher TONs as compared to the same catalyst in homogeneous phase. Even though this is a global effect that does not necessarily require specific interactions between the catalyst and the surrounding medium at the atomic level, it is of great technological importance. It also mimics the situation in many enzymes in which active sites are often buried in the interior of the protein matrix, to protect them from bimolecular encounters which could lead to dimerization/oligomerization, competing reactions or even decomposition. Following a concise review of different means of structural stabilization by the MOF matrix in section 3.1, we will subsequently discuss examples in which the MOF plays more specific roles. Working our way from third coordination sphere effects toward the active sites, cases are presented in which the MOF has been engineered to facilitate transport from the crystal surface to the catalyst. Specific pathways for substrate or products are reminiscent of the situation in many enzymes, where substrate access is highly regulated and can even determine the reaction outcome. Finally, sophisticated cooperative effects that are imposed on the catalyst by the surrounding MOF are being discussed. Parallels to second coordination sphere effects as defined in enzymes will be drawn. The chapter also contains examples on how the size/thickness of MOF crystals/thin films may give rise to transport limitations, as well as a system perspective on how MOFs with molecular catalysts are being interfaced with the surrounding media. While these are not effects within the catalyst-containing MOFs, both phenomena will have huge implications to their overall activity. Contacting is also an important topic in enzymes, some of which, for example, are embedded or associated with membranes or possess specific binding sites, for example, for ferredoxin, which connects the enzymes’ electron transport chain to the surrounding.^37^

### 3.1 Stabilization of Molecular Integrity

**Figure 16:**
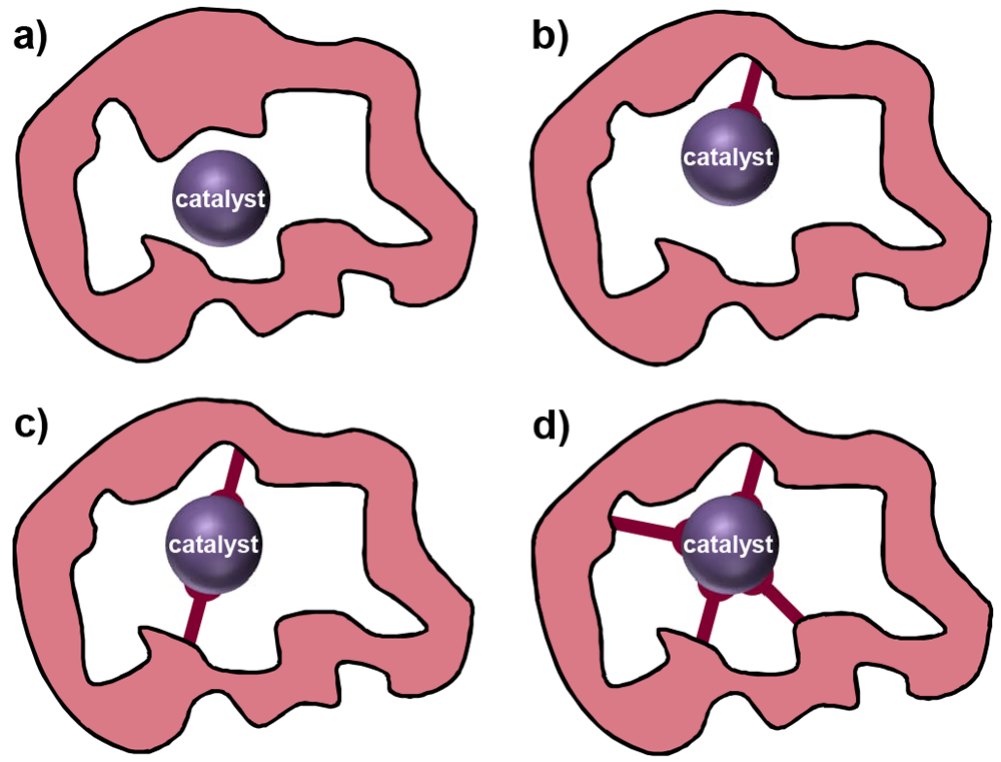
Schematic illustration of a catalyst moiety (purple sphere) inside a matrix (enzyme or MOF) with increasing degree of matrix/catalyst interaction from a) to d). Catalyst a) entrapped inside the matrix, b) coordinated to one dangling group, c) anchored by two groups, and d) fully fixed into the matrix.

The crystallinity and topological stability of MOFs can contribute to the structural stabilization of incorporated catalysts in various ways. Taking the degree of bonding interactions between the catalyst and the surrounding matrix, may it be a MOF or a peptide, as a basis for classification, catalyst incorporation can be divided into four themes (Figure 16). The first class contains examples in which the active site is merely physically encapsulated by the matrix without any specific atomic interaction (Figure 16a). This strategy is often referred to as the ship-in-a-bottle approach, expressing the fact that the catalyst needs to be bigger than the pore window of the MOF to prevent catalyst leakage. In the second class, matrix and catalyst interact through one specific interaction, for example through a linker that contains a dangling group that interacts with the catalyst through a covalent or coordination bond (Figure 16b). Catalysts that are decorated with a Lewis basic group that coordinates to the SBU belong into this design as well. Similar patterns in biology can, for example, be found in [FeFe] H_2_ases in which a cysteine-*S* is an integral part of the H-cluster active site. This cysteine is the only amino acid that provides a ligand to the active site. The two aforementioned approaches differ from the one in Figure 16c in that the catalyst of the latter is an integral part of the MOF, with one of the catalyst ligands being at the same time a MOF linker. In analogy, multiple first coordination sphere interactions can also be found, for example, in [NiFe] hydrogenases, carbon monoxide dehydrogenases or photosystem II. A common structural feature of these enzymes is that the active site metals also contain small inorganic ligands such as CO and CN^–^, μ-S, or μ-O ligands, respectively. Finally, all permanent ligands at the active site metals may be provided by the surrounding matrix (Figure 16d). In MOFs, this represents a situation in which the SBU exhibits the catalyst function, and all permanent ligands to the active site metals are provided by the MOF linkers. The latter class bears analogy to the nonheme diiron enzymes,^202^ the entire first coordination sphere of which is composed of amino acid side chains and substrate-derived ligands.

Irrespective of the bonding interactions described in Figure 16, MOF incorporation sterically isolates the molecular catalysts and protects from bimolecular catalyst encounters that, in the case of homogeneous counterparts, are often a major pathway for catalyst deactivation. This holds for reduction catalysts where low-valent metals may engage in unwanted bond forming reactions between catalyst molecules and even more so oxidation catalysts, where the formation of metal oxides is often thermodynamically favored. Another general effect that contributes to catalyst longevity is a tendency to slow down permanent ligand dissociation. This includes also situations where ligands may temporarily decoordinate from the metal, but cannot diffuse away due to the confinement provided by the MOF, thereby enabling recoordination and catalyst reconstitution.

**Figure 17:**
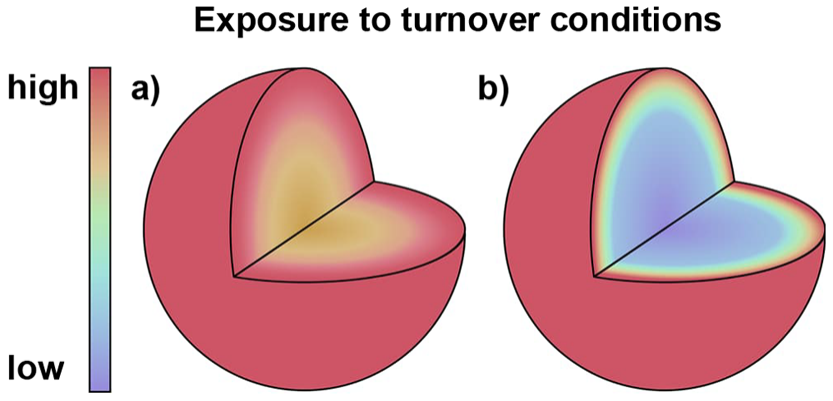
Spheres representing MOF crystals, where a) the entire crystal was relatively homogeneously exposed to turnover conditions or b) only the surface layer was sufficiently exposed. The gradient on the left illustrates the exposure to the turnover conditions from low to high.

Different techniques and types of experiments are being used in the field to describe and quantify the extent to which the MOF stabilizes the structural integrity of the incorporated catalysts. These include comparisons with homogeneous reference systems, recycling experiments that show that the catalytic material is active in repeated runs, as well as spectroscopic work, using diagnostic markers that are indicative of the structurally intact catalyst. The latter strategy gives perhaps the most information about the fate of the catalyst and can also be used to define the term “stabilization” more clearly. As MOFs are hundreds of nanometers to micrometer size objects, there is the possibility that not all catalysts are exposed to turnover conditions and that catalysis occurs only in a thin shell at the MOF/solution interphase (as schematically shown in Figure 17). This may be the case for MOFs in which transport of, for example, redox equivalents or a substrate, as well as a reductant or an oxidant, is limited, as discussed in section 2. In such cases, a certain proportion of the total catalyst population may be merely spectators and is therefore unchanged after the catalytic activity of the MOF ceases. As this scenario will result in postcatalysis materials with intact catalysts, it may first appear as structural stabilization, and additional experiments may be needed to establish the role of all catalysts within the MOF crystal. Spectator catalysts will also have implications on the apparent TONs and TOFs, as described in section 2. If these metrics are normalized to the total amount of catalysts, they may largely underestimate the actual TOFs and TONs in the environment provided by the MOF.

Prior to discussing specific examples of the four incorporation types in detail, one example of a MOF/catalyst system based on model complexes of the [FeFe] hydrogenase will be presented to illustrate the spectator catalyst topic, while another group of materials based on cobaloxime catalysts will be presented to illustrate the different incorporation designs.

#### [FeFe] Hydrogenase Active Site Models in MOFs: A Case Study

One of the most obvious biomimetic strategies to design and prepare MOFs for the HER is to take inspiration from [FeFe] H_2_ase enzymes. Reports of MOFs into which structural analogues of this enzyme active site have been incorporated have entered the scene relatively early. Already in 2013, Pullen et al.^203^ reported the incorporation of [FeFe](dcbdt)(CO)_6_ (dcbdt = 1,4-dicarboxylbenzene-2,3-dithiolato), a well-established enzyme active site model complex and proven HER catalyst, into **UiO-66**. A postsynthetic linker exchange strategy was chosen as the catalyst was not compatible with the harsh solvothermal conditions, and an incorporation yield of 14% was achieved. The resulting **UiO-66-[FeFe](dcbdt)(CO)**_**6**_ (Figure 19a) in combination with [Ru(bpy)_3_]^2+^ as a PS and ascorbate as an electron donor under illumination catalyzed the HER with overall higher TONs and even initial rates higher than those of a homogeneous reference system. IR studies after the ceased HER revealed that the vast majority of the [FeFe](dcbdt)(CO)_6_ was still structurally intact, thus suggesting that MOF integration has a stabilizing effect on the structure of the catalyst. In a follow-up study, it was shown that photocatalytic HER activity from the suspension of **UiO-66-[FeFe](dcbdt)(CO)**_**6**_ in a solution of [Ru(bpy)_3_]^2+^ and ascorbate could be recovered after 60 min of stirring without replacing any other component.^204^ This unexpected phenomenon was proposed to be due to intracrystal linker scrambling that brings structurally intact catalysts from the crystal interior to the surface, where they can then engage in catalysis. This interpretation also implies that the [FeFe](dcbdt)(CO)_6_ complexes in the inner MOF layers are not being reduced during earlier rounds of photocatalytic HER, and merely lie dormant. This hypothesis is consistent with results from another study that showed that a large proportion of [FeFe](dcbdt)(CO)_6_ complexes in **UiO-66-[FeFe](dcbdt)(CO)**_**6**_ remains in the initial oxidized state even in the presence of a large excess of cobaltocene,^198^ a chemical reductant of similar strength as [Ru(bpy)_3_]^+^ that is produced photochemically by reductive quenching of the *[Ru(bpy)_3_]^2+^ excited state in the earlier works. In contrast, the same [FeFe](dcbdt)(CO)_6_ complex can almost quantitatively be reduced when incorporated in **MIL-101(Cr)**, which is a MOF with larger pore diameters that allows more facile diffusion of the cobaltocene reductant to the [FeFe](dcbdt)(CO)_6_ complexes.^198^

**Figure 18:**
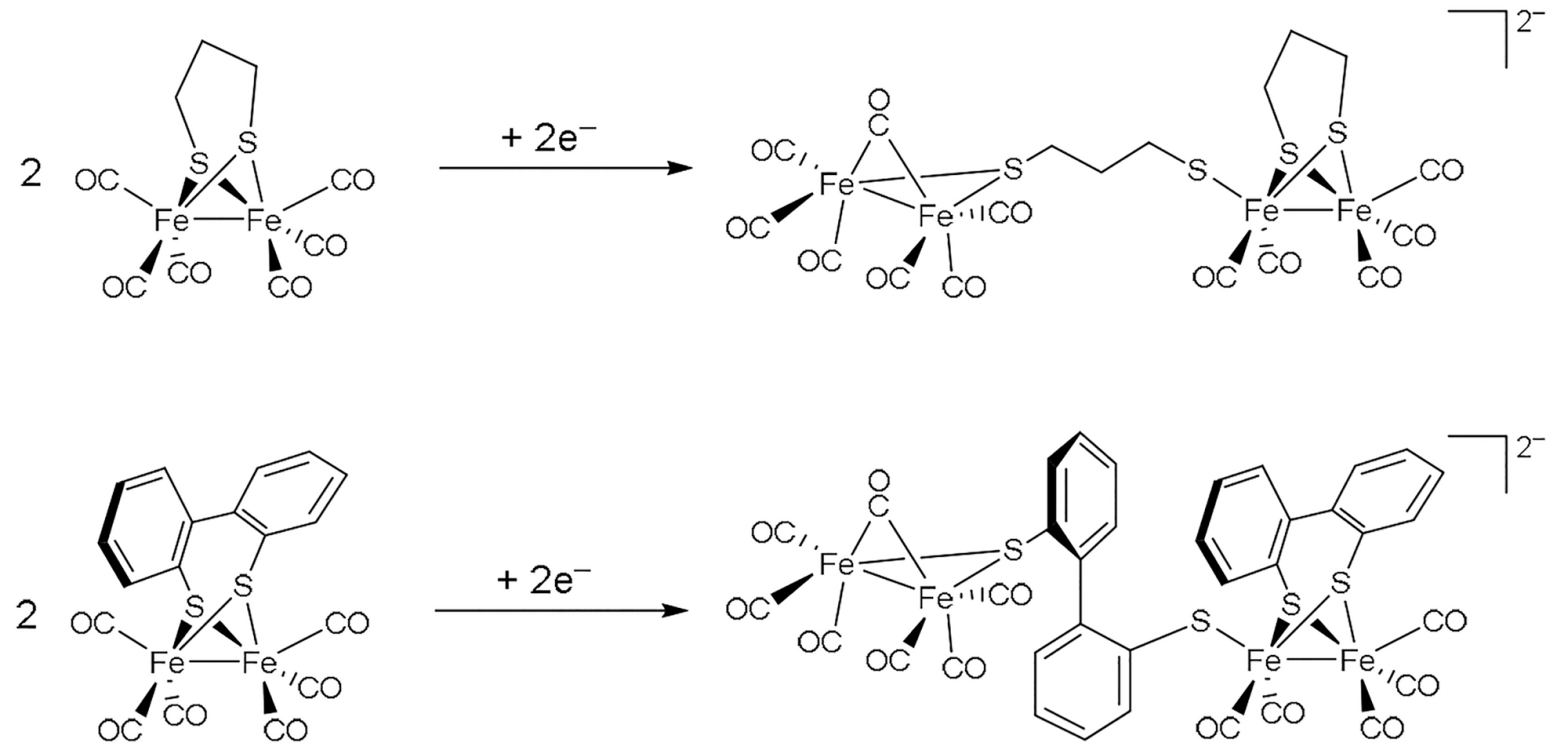
Dimerization of one-electron reduced model complexes of the [FeFe] H_2_ase active site that can be prevented owing to incorporation into MOFs.^205,206^

The accumulated results of these publications are that the mentioned TONs in the first report^203^ are most likely a significant underestimate, as only a fraction of total catalysts species is active during the initial round of irradiation. This also means that the active catalysts are greatly stabilized compared to the homogeneous reference catalyst. In homogeneous phase, it is well-established that one-electron reduced states of the catalyst are prone to engage in scission of an Fe–S bond to produce a free thiolate that can attack a second Fe_2_ complex to form tetra-nuclear species as those shown in Figure 18.^205,206^ This bimolecular decomposition pathway is most likely prevented by incorporating the catalyst in the MOF.

**Figure 19:**
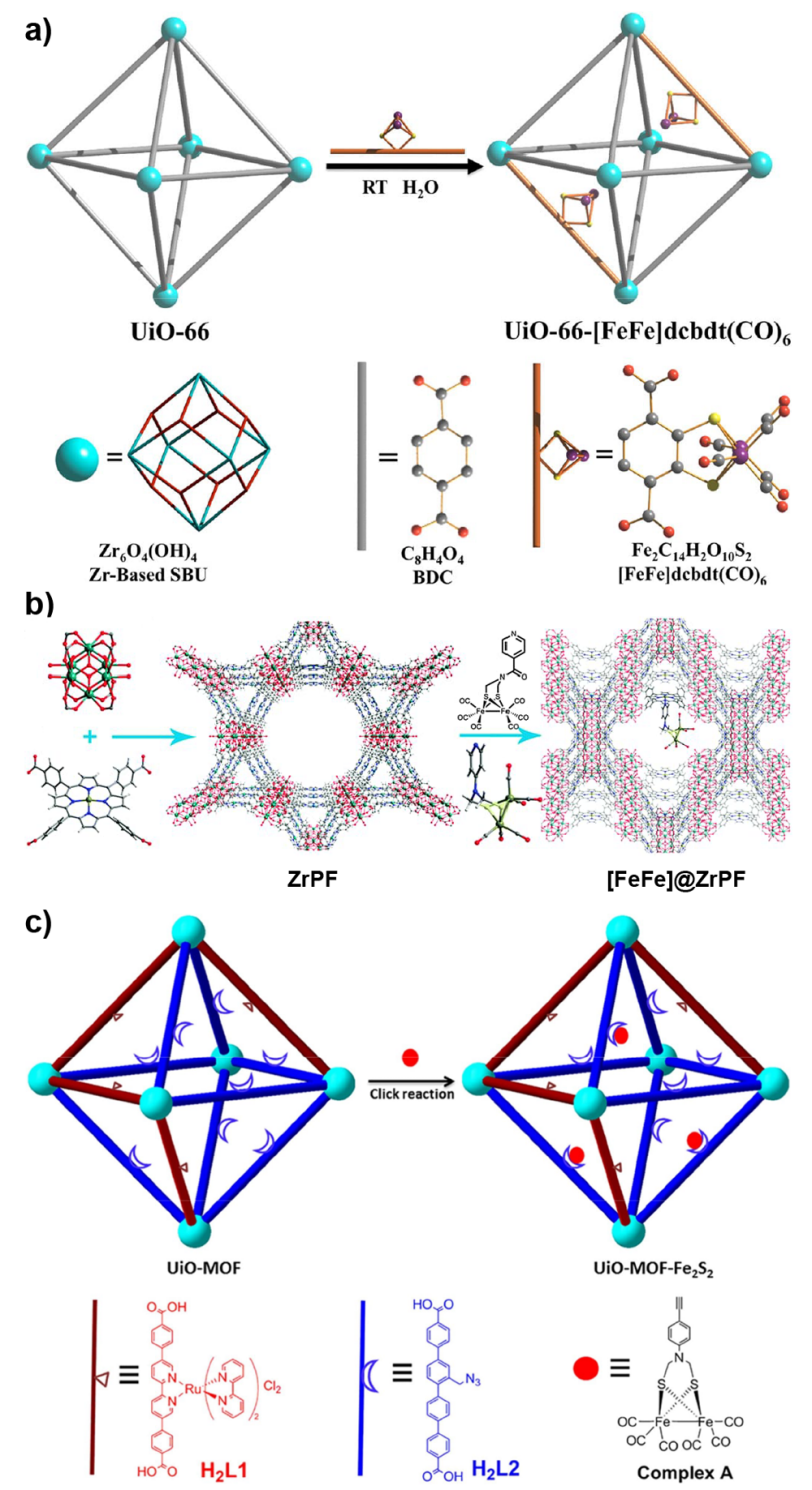
Examples of MOFs with incorporated model complexes of the [FeFe] H_2_ase active site. a) Incorporation of the H_2_ase mimicking complex [FeFe](dcbdt)(CO)_6_ into **UiO-66** by postsynthetic exchange. Reproduced with permission from reference (203). Copyright 2013 American Chemical Society. b) Postsynthetic decoration of **ZrPF** with [FeFe] H_2_ase active sites model complexes to form **[FeFe]@ZrPF**. Reproduced with permission from reference (207). Copyright 2014 Royal Society of Chemistry. c) Azide-functionality-driven click reaction to covalently attach a Fe_2_ catalyst to the linker units of a **UiO**-type MOF. Reproduced with permission from reference (208). Copyright 2019 Elsevier.

The structural stabilization becomes more apparent in MOFs into which both a PS as well as an active site model complex have been incorporated. In such systems, it is more likely that all catalysts are exposed to a photochemically produced reductant. Early work by Feng and co-workers^207^ described such a system based on a robust zirconium-porphyrin-based MOF (**ZrPF**). Herein, the [FeFe] H_2_ase active site model complex [((SCH_2_)_2_NC(O)C_5_H_4_N)Fe_2_(CO)_6_] is connected to the ZnTCPP linker through axial coordination by the pyridine moiety at the Fe_2_ complex (Figure 19b). Under illumination and in the presence of ascorbate, the Fe_2_-decorated MOF **[FeFe]@ZrPF** exhibits HER activity that vastly exceeds that of the corresponding homogeneous reference system. In another example, a multifunctional **UiO-69** that contained a [Ru(bpy)_3_]^2+^-derived metallo-linker and an azide-functionalized tetraphenyl-dicarboxylate was prepared. The latter engages in a click reaction with an acetylene-containing Fe_2_ complex to form a covalent attachment between the MOF linker and the Fe_2_ catalyst (**UiO-MOF-Fe**_**2**_**S**_**2**_ in Figure 19c).^208^ Under illumination and in the presence of ascorbate, this system resulted in a photochemical HER with higher yields than that of the corresponding homogeneous reference system under otherwise identical conditions. IR studies of both MOF systems, the **[FeFe]@ZrPF** and **UiO-MOF-Fe**_**2**_**S**_**2**_, after ceased photocatalysis showed complete disappearance of the IR frequencies that are attributed to the Fe_2_ catalyst. As decomposition is a result of catalyst reduction, it can be seen as an indicator that all catalysts have been exposed to turnover conditions. This being the case, both of the systems show higher TONs than the homogeneous references, illustrating the effect of the MOF matrix for catalyst stabilization.

From a biomimetic perspective perhaps the most complete mimic of [FeFe] H_2_ases was reported in 2021.^209^ In this paper, not only a model of the active site was incorporated but also redox-active linkers that model the [4Fe4S] electron transport chain of the enzyme. The system was realized by taking advantage of earlier work on **PCN-700**,^210^ which possesses two missing linker defect sites of specific size and positions in the crystal lattice. These sites were postsynthetically filled by the [FeFe](dcbdt)(CO)_6_ active site mimic and a naphthalene diimide-based redox linkers. Rigorous studies on the dual-functionalized MOF by cyclic voltammetry reveal similarities to the natural system but also important limitations in the MOF–enzyme analogy. Most importantly, restrictions apply to the total concentration charge-balancing counter cations that can be accommodated within the MOF. With the capacity of countercation uptake in the MOF being limited, not all linkers can be reduced electrochemically, even if the applied potential is well beyond the standard reduction potential of the isolated linker.^209^

#### Cobaloximes in MOFs: A Case Study

Over the years, the same catalyst motif has sometimes been incorporated into MOFs by multiple strategies of the ones outlined in Figure 16. This is nicely illustrated for a series of MOFs with incorporated cobaloximes, one of the most well-studied molecular HER catalysts.^211^ Cobaloximes contain two bidentate 1,2-diglyoxime ligands that occupy the equatorial coordination sphere of the central Co cation. Axial monodentate ligands such as pyridine, halides, or solvent molecules typically complete the primary coordination sphere of cobaloximes. As proton reduction involves the reduction of the Co center, usually to a Co^I^ level, these axial ligands are prone to decoordination; in fact, it is at these positions that hydride formation occurs. Structural decomposition pathways of the catalyst include reduction and hydrogenation of the glyoxime ligands followed by nanoparticle formation,^30,212−214^ as well as decoordination of the diglyoxime ligand, as evidenced by increased TONs in photocatalysis experiments (Eosin Y as PS and triethanolamine (TEOA) as donor) to which exogenous diglyoxime ligand was added.^215^ This effect was rationalized by excess diglyoxime pushing the decoordination equilibrium to the complex side. Incorporation of cobaloximes into heterogeneous matrices such as MOFs as an alternative means to stabilize their molecular structure has been in the center of attention in numerous studies. From an incorporation point of view, most conceivable ways to incorporate cobaloximes have been explored, most of which show the desired effect that the molecular integrity of the catalyst is increased.

**Figure 20:**
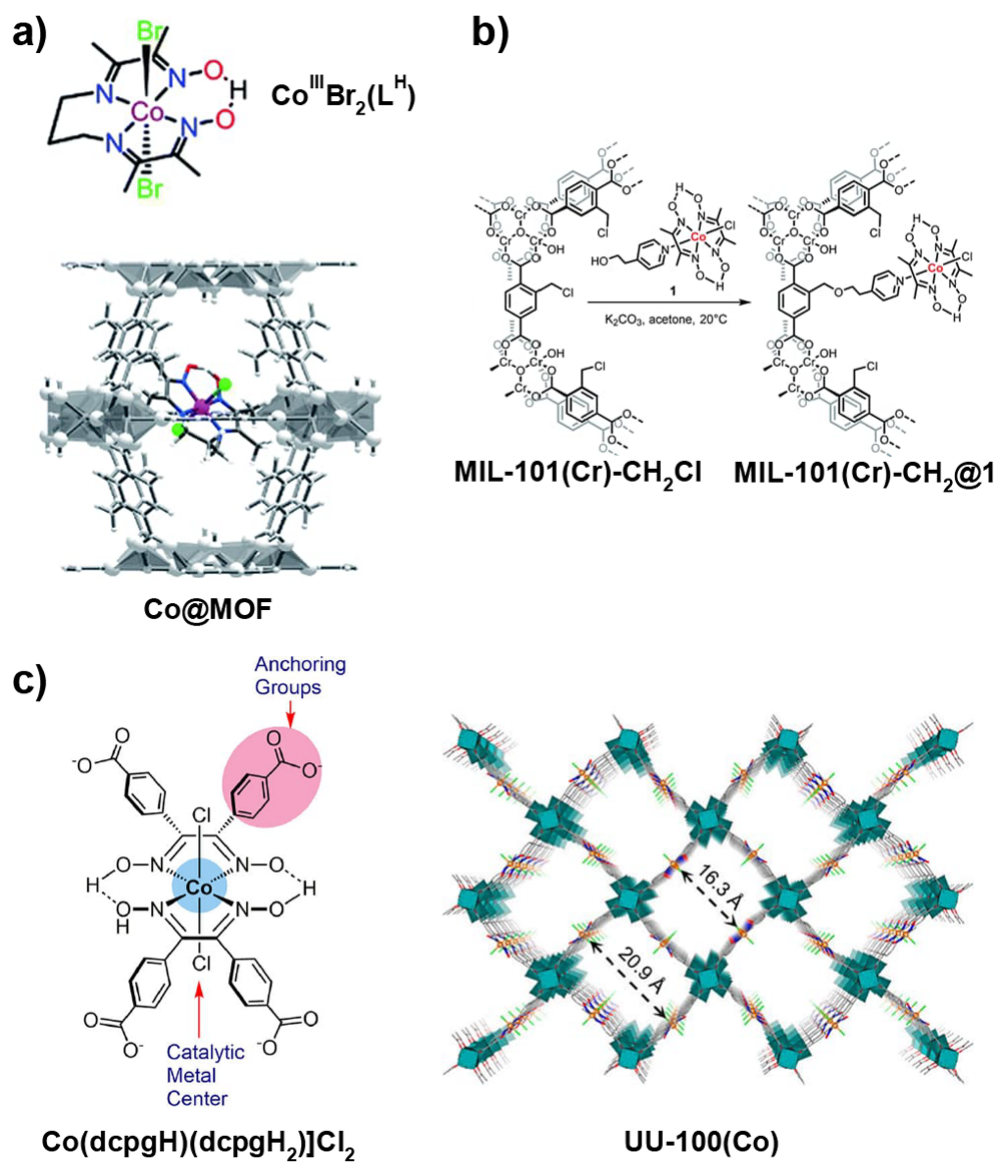
Examples of cobaloxime analogues incorporated in MOFs: a) Ship-in-a-bottle approach, based on the incorporation of Co^III^Br_2_(L^H^) into **NH**_**2**_**-MIL-125(Ti)** to form **Co@MOF**. Adapted with permission from reference (216). Copyright 2015 Royal Society of Chemistry. b) Cobaloxime complex covalently linked to the MOF matrix, via postsynthetic functionalization on the aromatic rings of **MIL-101(Cr)** after chlorination and immobilization of the catalyst. Adapted with permission from reference (218). Copyright 2018 Royal Society of Chemistry. c) Cobaloxime analogues can serve as structural linkers, when comprising carboxylate anchors as shown in Co(dcpgH)(dcpgH_2_)Cl_2_. Reaction with preassembled Zr-oxo clusters led to the formation of **UU-100(Co)**. Adapted with permission from reference (219). Copyright 2015 American Chemical Society.

For example, a Co-dioxime-diimine has been assembled in the pores of a photoactive **NH**_**2**_**-MIL-125(Ti)** following a ship-in-a-bottle strategy (Figure 20a).^216^ The resulting **Co@NH**_**2**_**-MIL-125(Ti)** could be recycled a couple of cycles over the time scale of days and functioned as a light-driven HER catalyst in the presence of triethylamine (TEA). In related work, the parent cobaloxime was assembled from the diglyoxime ligands and the Co salt in the presence of **NH**_**2**_**-MIL-125(Ti)** to give a material that photocatalytically produced H_2_. In these experiments, TEOA was used as a sacrificial donor, and H_2_ production rates could be increased by additional Eosin Y as a cophotosensitizer.^217^

Going beyond the ship-in-a-bottle approach, cobaloximes were incorporated by a coordination linkage in the interior of appropriately functionalized **MIL-101(Cr)** (Figure 20b).^218^ The resulting material was shown to be an efficient photocatalyst with a high rate for H_2_ evolution in the presence of TEOA as an electron donor and Eosin Y as a PS. The obtained results are consistent with the hypothesis that cobaloxime species, [Co^I^(dmgH)_2_] and [Co^III^(dmgH)_2_(H)] (dmgH = dimethylglyoxime), that are detached from the pyridine groups during the photocatalytic cycle remain in close proximity of the pyridine anchors inside the MOF-pores. Restoration of the Co–pyridine coordination bond during catalysis is therefore more likely to occur inside the MOF-pores compared to the situation in the homogeneous system. Consequently, the heterogeneous MOF–cobaloxime hybrid displayed enhanced HER activity due to the presence of the axial *N*-ligand. Eventual deactivation of the system likely occurs through diffusion of detached [Co(dmgH)_2_] units out of the MOF-cages, followed by loss of the glyoxime ligands from cobalt.

Finally, cobaloximes have also been incorporated as structural linkers in a MOF (Figure 20c).^219^ For this purpose, tetraphenyl-cobaloxime was decorated with four carboxyl-groups for anchorage to Zr_6_-cluster-based SBUs. The resulting material, **UU-100**, showed electrocatalytic hydrogen evolution over more than 20 h when grown on glassy carbon substrates. This result illustrates the power of MOF incorporation, as electrochemical reference experiments on the homogeneous linker lead to complete catalyst degradation after a couple of turnovers. In contrast to the homogeneous case where ligand decoordination from the Co center is not restricted, the diglyoxime ligands in **UU-100(Co)** are held in place by their anchorage to four difference SBUs. Even if transiently decoordinating from the catalytic Co center, the ligand stays in the vicinity, offering the possibility for recoordination.

#### 3.1.1 Catalysts Entrapped within MOF Pores

In the absence of a ligand at the molecular catalyst that could synthetically be modified to allow for MOF incorporation, a ship-in-a-bottle strategy, as illustrated in Figure 16a, can be employed. This strategy is suitable for catalysts that are smaller than the pores of the chosen MOF, but larger than its pore windows. In that way, the catalyst is physically trapped inside the MOF crystal without directly interacting with the framework in a specific, atomically defined manner. In the absence of covalent or coordination bonds, this section also includes examples in which the molecular catalyst is imbedded in the MOF pores by electrostatic or van der Waals interactions. Catalysts can be trapped during the solvothermal synthesis of the MOF, as well as that they can be assembled in the MOF pores in a postsynthetic process.

##### 3.1.1.1 Polyoxometallates (POMs)

Polyoxometalates (POMs) as catalysts for energy-relevant transformations have received significant attention in recent years due to their high catalytic activity, but they have also been criticized for their instability, sometimes being merely precursor species for poorly defined, heterogeneous, yet very active metal oxides.^220,221^ For example, certain Co_4_POMs have been found to transform under certain catalytic conditions into CoO_*x*_ oxide, which itself is a competent WOC.^222^ Such transformations appear to occur via initial leaching of Co^II^ from the Co_4_POM into the bulk solution, followed by formation of the CoO_*x*_ oxide.

POMs have been incorporated into MOFs by the encapsulation approach, wherein the POM is physically trapped inside a MOF’s pores. In 2018, Das and co-workers^223^ encapsulated both a molecular cobalt complex and a Keggin K_6_[CoW_12_O_40_] POM inside the pores of **ZIF-8**. The POM@MOF species proved to be a competent WOC at a neutral pH, while the homogeneous POM was not observed to evolve O_2_. The authors argue that for successful stabilization, the guest must be bigger than the MOF pore windows and yet fit within the cavities. Without meeting these size-match criteria, leaching can occur, with resultant possible formation of heterogeneous metal oxides or hydroxides.

The handful of examples demonstrating water oxidation by POMs encapsulated inside MOFs suggest that POM stability is enhanced compared to that of their homogeneous analogues, however, as discussed above, the probable decomposition pathway to CoO_*x*_ begins with leaching of Co^II^ from the POM clusters. It is not yet clear why MOF encapsulation guards against this pathway. As recently as 2020, the enhanced stability of POMs in MOFs was purely attributed to preventing leaching of the POM units.^224^ For a more complete review of stabilization of POMs in MOFs for diverse catalytic applications largely not related to solar fuels, see the recent summary of Dolbecq and co-workers.^225^

**Figure 21:**
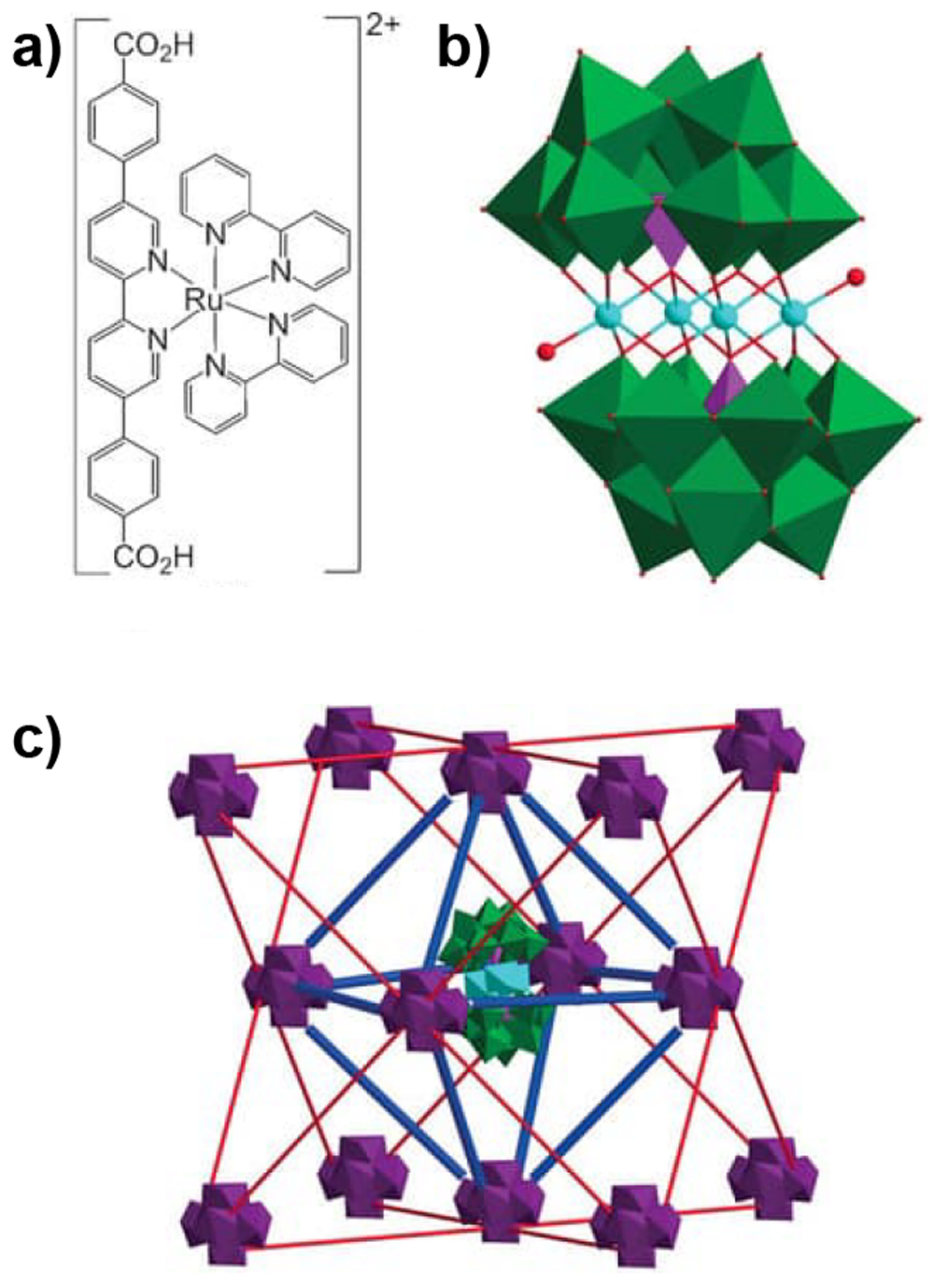
Example of catalyst and PS coimmobilization within a MOF by a ship-in-a-bottle strategy for light-driven HER. a) structure of a Ru-based PS linker. b) Polyhedral view of the structure of [Ni_4_(H_2_O)_2_(PW_9_O_34_)_2_]^10–^ (Ni_4_P_2_). c) Structural model showing unoccupied tetrahedral cavities and the central Ni_4_P_2_-loaded octahedral cavity. Adapted with permission from reference (227). Copyright 2016 WILEY-VCH Verlag GmbH & Co.

POMs have also been trapped inside **UiO**-type MOFs to obtain stabilized HER catalysts (Figure 21).^226,227^ In these materials, the encapsulating MOF also hosted a [Ru(bpy)_3_]^2+^ PS as an integral part of the constituting MOF linkers, resulting in a system that was well set up for light-induced electron transfer reactions to the POM-based HER catalyst. Photophysical and electrochemical studies established the oxidative quenching of the excited PS by the POM as the initiating step of HER.

MOF-encapsulated POMs with high negative charges, when entrapped in MOFs, can also attract additional positively charged guests into the MOF matrix. This strategy has been reported for a **MIL-101(Cr)** framework into which anionic Wells–Dawson-type POMs had been incorporated during solvothermal MOF synthesis.^228^ Owing to the high negative charge of the POM, the POM@MOF composite allows for the adsorption of cationic rutheniumtris(bipyridyl) ([Ru^II^(bpy)_3_]^2+^) PS, resulting in a POM@PSs@MOF. In the presence of TEOA, this hybrid system catalyzes the light-driven HER with much higher activity than that of the corresponding homogeneous reference system.

##### 3.1.1.2 Polypyridyl Complexes

Polypyridines are popular ligands to transition metals in catalysis of energy relevance. 2,2′-Bipyridine (bpy) and 2,2′:6′,2″-terpyridine (terpy), as well as ligand motifs in which the pyridines are separated by saturated units, in principle, allow for synthetic modifications to introduce anchoring points for SBU coordination and thus MOF production. On the other hand, polypyridine-based catalysts are typically rather large, which qualifies them also for a ship-in-a-bottle incorporation into suitable MOF scaffolds.

**Figure 22:**
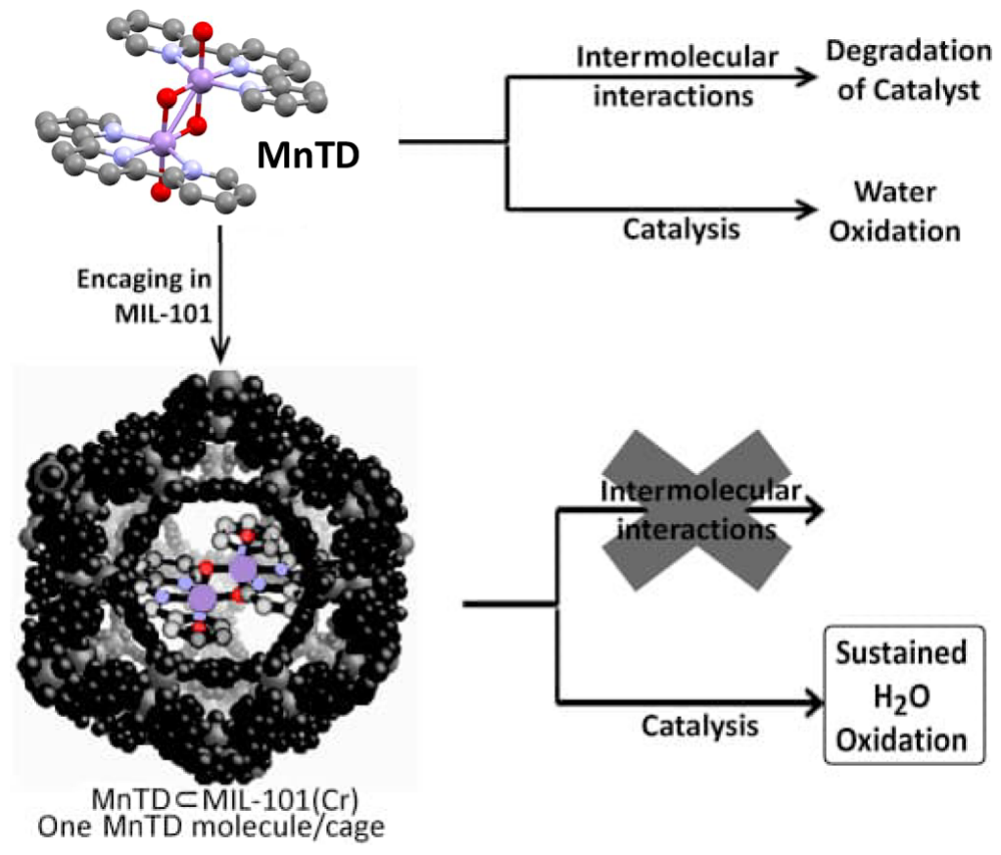
Structure of the **MnTD** water oxidation catalyst and its catalytic activity as either a homogeneous solution or encapsulated inside **MIL-101(Cr)**. Atom labeling: C, gray; O, red; N, blue; Mn, purple. H atoms are omitted for clarity. Adapted with permission from reference (229). Copyright 2013 WILEY-VCH Verlag GmbH & Co.

One such example is a dinuclear manganese-based WOC, **MnTD** ([(terpy)Mn(μO)_2_Mn](terpy)]^3+^), that has received considerable attention both for its high activity and similarity to the oxygen-evolving complex of photosystem II. Kinetic analysis of **MnTD** has revealed that catalysis is first order in **MnTD**, but the major degradation products require multiple **MnTD** equivalents.^229^ Significant efforts had focused on bulking up the ligand substituents to improve resistance to bimolecular decomposition until Nepal and Das^229^ reported the postsynthetic assembly of **MnTD** inside the pores of **MIL-101(Cr)** via a stepwise process. The pristine **MIL-101(Cr)** was first soaked in a solution of terpy ligand for 18 h followed by addition of Mn(OAc)_2_ and K-oxone to form the **MnTD** catalyst in the pores of **MIL-101(Cr)**,^229^ as evidenced by FTIR and EPR spectroscopic studies. Chemical oxidation experiments established that while homogeneous **MnTD** had a higher initial catalytic rate, the **MnTD@MIL-101(Cr)** material (Figure 22) maintained catalytic activity for a longer time period as long as fresh oxidant was supplied. To permit comparison between homogeneous and MOF-encapsulated catalysis, the same initial quantity of **MnTD** was used in both scenarios. The authors were careful to note that this “analytical quantification techniques would not distinguish the catalytically active and inactive forms of Mn” within the **MIL-101(Cr)** MOF and that therefore the catalytic enhancement as a result of MOF incorporation could be higher than reported.^229^ Intriguingly, the authors commented that Hatton and co-workers^230^ found no difference in catalytic rate for Baeyer condensation reactions using an analogous ship-in-a-bottle approach with **MIL-101(Cr)** for crystallite sizes ranging from ca. 400 nm up to 10 μm, pointing toward catalysis occurring throughout the **MIL-101(Cr)** crystals. Nepal and Das^229^ argued that based on these literature results, water oxidation for their system is likely also not transport limited, though no kinetic comparison of different particle sizes was examined. Hansen and Das^231^ demonstrated that the previously described **MnTD@MIL-101(Cr)** material was catalytically active for up to seven continuous days of operation. A gradual decrease of catalytic activity was rationalized as possible buildup of insoluble Ce^III^ species originating from the employed sacrificial oxidant CAN, emphasizing the need to avoid sacrificial reagents and instead rely on electrode constructs. An analogous MOF host with pores large enough to host two molecules of **MnTD** had noticeably decreased catalytic lifetime (7 h versus 7 days), supporting the notion that catalyst site isolation leads to enhanced stability.^231^ Since their initial report, the group of Das has reported similar success for water oxidation using an encapsulated Co complex as a WOC in a Co framework, **Co-WOC-1**.^232^

In 2017, Park et al.^233^ reported the functionalization of the anionic zinc-adeninate framework **bMOF-100** via postsynthetic cation exchange with [Ru(bpy)_3_]^2+^. Thereby, the mesopores of **bMOF-100** were spacious enough to host on average 2.43 [Ru(bpy)_3_]^2+^ cations. **[Ru(bpy)**_**3**_**]**^**2+**^**@bMOF-100** was further immobilized on the surface of a glassy carbon electrode (GCE) to investigate its electrocatalytic and electrochemiluminescence properties. In an aqueous electrolyte solution, the cyclic voltammograms of **Ru@bMOF-100** showed an irreversible Ru^III/II^ couple that the authors interpreted as an electrocatalytic OER.

Following the theme of light-driven proton reduction based on a photoresponsive MOF and a molecular HER catalyst, a molecular Ni^II^ catalyst, [Ni(dmobpy)(2-mpy)_2_] (dmobpy = 4,4′-dimethoxy-2,2′-bipyridine, 2-mpy = 2-mercapto-pyridyl) was incorporated in a photoactive MOF, **NH**_**2**_**-MIL-125(Ti)**. Synthetically, the catalyst was assembled in the pores of the MOF by simple soaking of the two ligands and a Ni^II^ precursor. ^1^H NMR spectroscopic evaluation of digested **Ni@NH**_**2**_**-MIL-125(Ti)** as well as other spectroscopic studies support the formation of the [Ni(dmobpy)(2-mpy)_2_] catalyst within the composite. In the presence of TEA as electron donor, **Ni@NH**_**2**_**-MIL-125(Ti)** catalyzed the light-driven HER at appreciable rates for up to 50 h, substantially longer than corresponding reference experiments.^234^ The same **NH**_**2**_**-MIL-125(Ti)** MOF was also used to host a Co^II^-based molecular HER catalyst, [Co^II^(TPA)CI][CI] (TPA = tris(2-pyridylmethyl)-amine).^235^ Similar to the previous example, the Co complex was assembled inside the MOF cages by successive exposure of the **NH**_**2**_**-MIL-125(Ti)** MOF to the ligand, followed by the Co^II^ salt. The molecular integrity of the catalyst was proven by NMR spectroscopy and mass spectrometry of digested samples. In the presence of TEOA as an electron donor, the **Co@NH**_**2**_**-MIL-125(Ti)** hybrid catalyzed the light-driven HER at a rate that greatly exceeded that of a physical mixture of **NH**_**2**_**-MIL-125(Ti)** and the same Co^II^ complex in solution, illustrating the necessity for close proximity between the light-responsive MOF and the encapsulated catalyst for appreciable electron transfer.

In terms of biomimicry, also model complexes of the [NiFe] H_2_ase active site have been incorporated into MOFs. **NiFe@PCN-777** was synthesized by simple encapsulation of [NiFe] ([L^N2S2^Ni^II^Fe^II^Cp(CO)]BF_4_; L^N2S2^ = 2,2′-(2,2′-bipyridine-6,6′-diyl)bis(1,1′-diphenylethanethiolate, Cp = cyclopentadienyl) into the Zr-based MOF **PCN-777** through soaking. The interactions between the **PCN-777** matrix and **NiFe** are nonspecific, but involve electrostatic interactions of the Zr-cluster surface with the [L^N2S2^Ni^II^Fe^II^Cp(CO)]^+^ cation and/or the BF_4_^–^ anion. Electrochemical reductions associated with the molecular [NiFe] complex could be observed in cyclic voltammograms of **NiFe@PCN-777** that had been immobilized on fluorine-doped tin oxide (FTO) by electrophoretic deposition. In the presence of acid, cyclic voltammograms of **NiFe@PCN-777** show a current enhancement that is consistent with proton reduction, the magnitude of which is however largely similar to the currents observed for analogous films of the parent **PCN-777**. The large background current of the latter on FTO makes it difficult to determine the extent to which the molecular catalyst in **NiFe@PCN-777** is involved in catalysis, and prevents a more rigorous kinetic treatment of the system.^236^

Yan et al.^237^ reported the construction of [Ni^II^(bpet)(H_2_O)_2_] (bpet = 1,2-bis((pyridin-2-ylmethyl)thio)ethane) in the pores of the photosensitizing **Ru-UiO-67** ([Zr_6_(μ_3_-O)_4_(μ_3_–OH)_4_(bpdc)_5.7_(Ru(bpydc)(bpy)_2_)_0.3_]·(OAc)_0.6_) (bpdc = biphenyl-4,4′-dicarboxylate) framework. The photocatalytically active composite **Ni@Ru-UiO-67** was produced in a typical ship-in-the-bottle fashion by entrapment of bpet in **Ru-UiO-67**, followed by its metalation with Ni(ClO_4_)_2_·6H_2_O. By varying the amount and ratio of bpet and the Ni^II^ precursor, three different composites **Ni1@Ru-UiO-67**, **Ni2@Ru-UiO-67**, and **Ni3@Ru-UiO-67** were prepared, which differed in their Ni^II^ loadings of 0.15, 0.23, and 0.31 wt %, respectively. All **Ni@Ru-UiO-67** composites displayed photocatalytic activity for the reduction of CO_2_, with **Ni3@Ru-UiO-67** showing 99% selectivity for CO with an appreciable TON.

#### 3.1.2 Catalysts Anchored in MOF Pores

While the previous section reviewed MOF–catalyst composites in which the catalyst was trapped either physically or by nonspecific electrostatic interactions in the MOF pores, examples in the present section rely on more specific catalyst–MOF interactions. This design generally allows more control as to the spatial positions of the constituting components, also resembling the situation in enzymes more closely. The molecular catalysts are either covalently bound to a MOF linker, or encapsulated through defined noncovalent interactions, including hydrogen bonds. The noncovalent interactions may also be between Lewis basic groups that are “dangling” off the MOF linkers and that coordinate to the transition-metal catalyst, or between the Lewis acidic SBUs and catalysts that are equipped with a Lewis base.

##### 3.1.2.1 Catalysts Anchored to Linkers

Zhang et al.^238^ postsynthetically added terpyridine ligands via covalent bonds to the internal pores of **MIL-101(Cr)**, followed by complexation of manganese to yield a di-μ-oxo dimanganese unit. This material was found to evolve oxygen upon addition of a chemical oxidant and, notably, no leaching of Mn-species was found in the bulk solution after catalysis. The authors also recreated the **MnTD@MIL-101(Cr)** material of Nepal and Das^229^ (see section 3.1.1.2) and found that leaching of Mn did occur under similar catalytic conditions. Based on this comparison, it was argued that covalent attachment gives rise to more stable MOF/catalyst hybrids than the ship-in-a-bottle approach.

Using the same parent **MIL-101(Cr)** MOF, Ott and co-workers^239^ described the postsynthetic incorporation of the molecular WOC [Ru(bda)(L)_2_] (bda = 2,2′-bipyridine-6,6′-dicarboxylate; L = pyridine-based axial ligands) into two different **MIL-101(Cr)**-derived frameworks. The WOCs were incorporated through one of the axial pyridine ligands that in turn were covalently bound to functionalized linkers in the **MIL-101(Cr)**. The benefits of stabilizing the structural integrity of the WOC within the **MIL-101(Cr)** matrix became evident during water oxidation experiments over a longer time period and in the presence of a higher concentration of the oxidant CAN. After 1 h, one of the **MIL-101(Cr)@Ru(bda)** materials displayed TONs of 1500, around 10 times higher than that of the homogeneous control experiment. The study also contained attempts to elucidate whether catalysis is surface-confined or a bulk phenomenon, as well as whether catalysis proceeds by a nucleophilic attack or radical coupling mechanism. In a follow-up study, the same group presented a quantitative kinetic model that allowed to distinguish between surface and bulk reactivity in a catalytic MOF (see also discussion around Figure 9 for details).^98^

Liang et al.^240^ demonstrated the integration of Ru-based molecular WOCs into the **MIL-101(Cr)** framework through an amide bond connection to the linkers of the scaffold. The authors described the homogeneous control catalysts [Ru(terpy)(pic)_3_](PF_6_)_2_ (pic = 4-picoline) and [Ru(terpy-Ac)(pic)_3_](PF_6_)_2_ (terpy-Ac = [2,2′:6′,2″-terpyridine]-4′-carboxylic acid), as well as the three novel heterogeneous systems with covalently anchored Ru-based catalysts **MIL-101(Cr)-[Ru(terpy-Ac)(pic)**_**2**_**Cl](PF**_**6**_**)**, **MIL-101(Cr)-[Ru(terpy)(isc)Cl**_**2**_**]** (isc = isonicotinic acid), and **MIL-101(Cr)-[Ru(terpy)(isc)(pic)**_**2**_**](PF**_**6**_**)**_**2**_. The Ru-based catalysts were built into the MOF structure through amide bond at the terpy-Ac ligand in case of **MIL-101(Cr)-[Ru(terpy-Ac)(pic)**_**2**_**Cl](PF**_**6**_**)**, or the isc ligand in case of the other two materials. The heterogeneous hybrid materials were found to be robust catalysts for the OER, with the CAN-driven OER efficiency of **MIL-101(Cr)-[Ru(terpy-Ac)(pic)**_**2**_**Cl](PF**_**6**_**)** being 120 times higher compared to that of the nonimmobilized Ru-catalyst. The experimental results revealed that the Ru-aqua species, [Ru(terpy)(pic)_2_(OH_2_)]^2+^, is the actual active species in the OER. The formation of the aqua complex proceeds preferentially by replacement of chloride ligands. In the absence of chloride ligands at the Ru complex as in **MIL-101(Cr)-[Ru(terpy)(isc)(pic)**_**2**_**](PF**_**6**_**)**_**2**_, isc that constitutes the anchor to the MOF matrix is replaced by water, explaining the experimentally found poor stability of this construct during water oxidation experiments.

**Figure 23:**
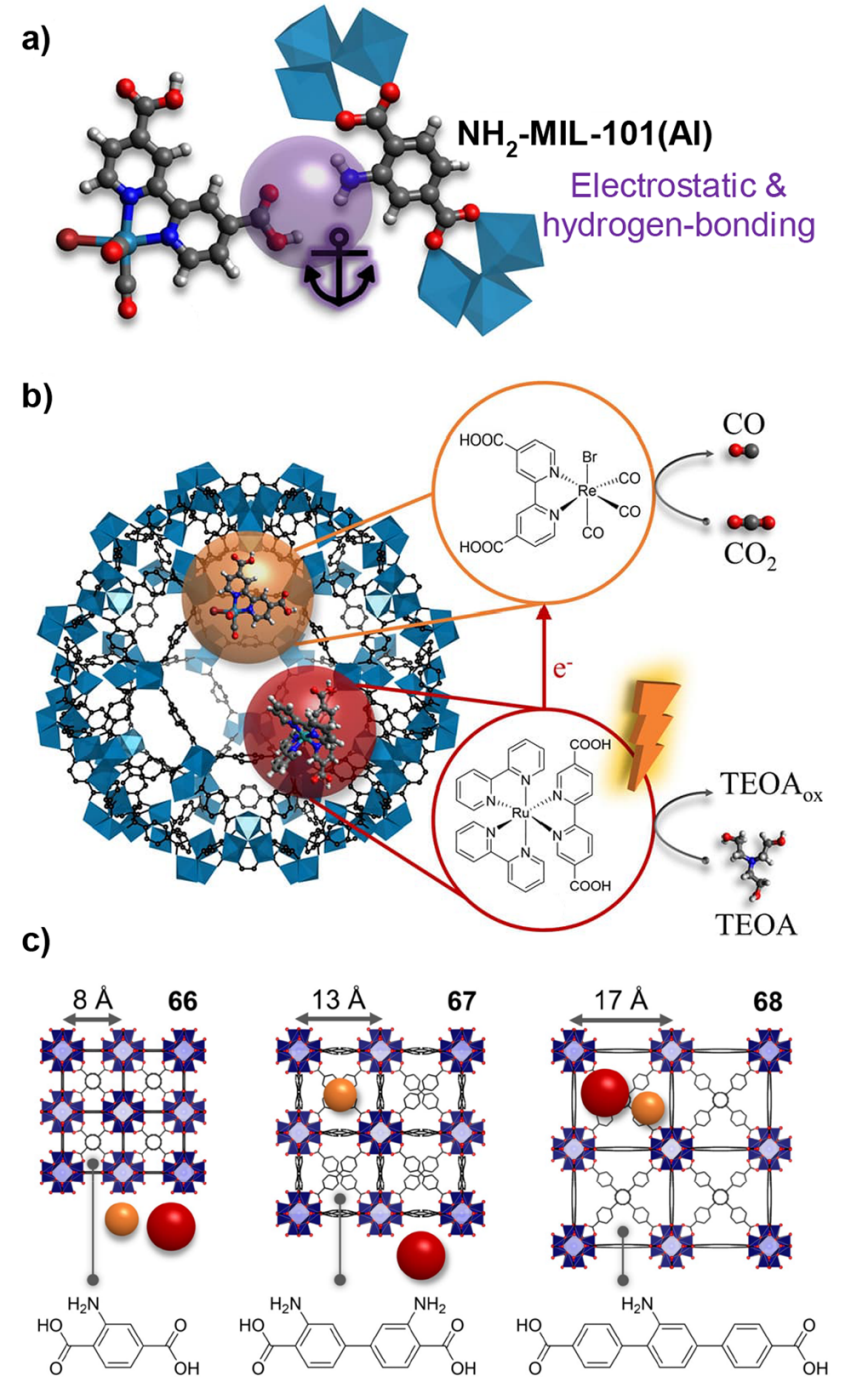
a) Noncovalent anchoring of the carboxylic acids of the encapsulated catalyst to the amine functionalities of **NH**_**2**_**-MIL-101(Al)**. b) Photoinduced electron cascade from triethanolamine (TEOA) over the [Ru(bpy)_2_(bpydc)]Cl_2_ PS (red) to the ReBr(CO)_3_(bpy-4,4′-dc) catalyst (orange) for the CO_2_ reduction reaction in a host–guest environment. Adapted with permission from reference (242). Copyright 2021 American Chemical Society. c) Catalyst (orange) and PS (red) location in the isoreticular **UiO** (**66**, **67**, **68**) host series. Adapted with permission from reference (243). Copyright 2021 WILEY-VCH Verlag GmbH & Co.

MOFs can also serve as host matrices for the concomitant incorporation of molecular catalysts and PSs (different arrangements presented in Figure 14). In 2018, Wang et al.^241^ established such a system by entrapping the molecular catalyst [Cp*Rh(bpy-4,4′-dc)]^2+^ (Cp* = Cp* = η^5^-C_5_Me_5_) and the PS [Ru(bpy)_2_(bpy-4,4′-dc)]^2+^ within **NH**_**2**_**-MIL-101(Al)**. The host framework acts as a protective cage for the captured components which are held in place by H-bonding interactions between the carboxylate-functionalized complexes and the NH_2_-functionalized MOF matrix. Owing to these interactions, leaching out during photocatalysis is inhibited. Utilization of the resulting **Rh–Ru@NH**_**2**_**-MIL-101(Al)** in the CO_2_RR displayed a change in selectivity toward the exclusive production of formate and suppression of H_2_ production, compared to the homogeneous counterpart. In 2021, Stanley et al.^242^ followed with a similar demonstration to coimmobilize *fac*-ReBr(CO)_3_(bpy-4,4′-dc) and a Ru-based PS within the pores of a **NH**_**2**_**-MIL-101(Al)** MOF (Figure 23a and b). This system showed selective CO evolution under photocatalytic CO_2_ reduction conditions from 1.5 to 40 h, indicating improved stability compared to the homogeneous reference. Furthermore, in contrast to typical homogeneous systems, not catalyst but PS degradation was identified as the major performance-limiting factor, corroborating the high potential of MOF platforms to stabilize catalytically active sites.

In another study, Stanley et al.^243^ reported the coimmobilization of the molecular CO_2_ reduction catalyst [ReBr(CO)_3_(bpy-4,4′-dc)] and the PS [Ru(bpy)_2_(bpydc)]Cl_2_ within the isoreticular series of amino-decorated **UiO-66**, **-67**, and **-68** frameworks (Figure 23c). The series displays a range of maximum pore diameters of 8.0, 13.1, and 17.2 Å, for **UiO-66**, **-67**, and **-68** respectively, and will thus host the molecular Recatalyst (12 Å) or/and the Ru-PS (15 Å) either on the MOF surface, as in **UiO-66**, or within the pores within the pores in case of the more spacious analogous. Immobilization of the molecular species is guaranteed through interactions of the carboxy groups at the molecular components with the SBUs and the amine moieties of the linkers. When illuminated and in the presence of TEOA, the **UiO-66** assembly with surface confined catalyst and PS showed limited TONs and deactivation after 1.5 h due to the instability of the molecular catalyst on the MOF surface. In the **UiO-67** assembly, the smaller catalyst was able to enter the pores, while the larger PS remained on the outside, thereby partially blocking the pores. Consequently, **ReRu-67** showed very low activity, most likely due to disabled electron transfer between the surface anchored PS and the entrapped catalyst. In case of **UiO-68**, the catalyst as well as the PS were able to be coimmobilized within the framework. **ReRu-68** constructs with a slight excess of PS displayed TONs of about 10, which is comparable to those of the homogeneous reference system, but higher stability over a longer period of time. Greatly improved TONs were obtained by replacing TEOA with another sacrificial electron donor, namely, BIH (1,3-dimethyl-2-phenyl-2,3-dihydro-*1H*-benzo[*d*]imidazole), leading to increased molecular stability and activity. **ReRu-66** in combination with BIH reached final TONs of 419 ± 31 until its deactivation within 5 h. While **ReRu-67** still showed limited reactivity, **ReRu-68** displayed total TONs of 506 ± 29 after two 24 h cycles.

In 2019, Liu et al.^244^ anchored catalytically active Co^II^ centers postsynthetically into a robust Zr-based MOF (**Zr-DMBD**), functionalized with dangling thiol groups. The well-defined Co–thiolate units in the resulting **Zr-DMBD–Co** MOF were capable to convert CO_2_ into CO in a photocatalytic assay consisting of a Ru-based PS and TEOA as sacrificial reductant. A series of **Zr-DMBD–Co** with different Co^II^ contents, designated as **Zr-DMBD–Co-*x*** (*x* = wt % of Co^II^ ions), were synthesized to investigate the influence of the Co^II^ loading on the catalytic activity. Of the **Zr-DMBD–Co** materials that were studied, the one with the lowest Co loading, gave the highest formal TON and TOF and a CO selectivity of 98% over 10 h.

##### 3.1.2.2 Catalysts Anchored to SBUs

**Figure 24:**
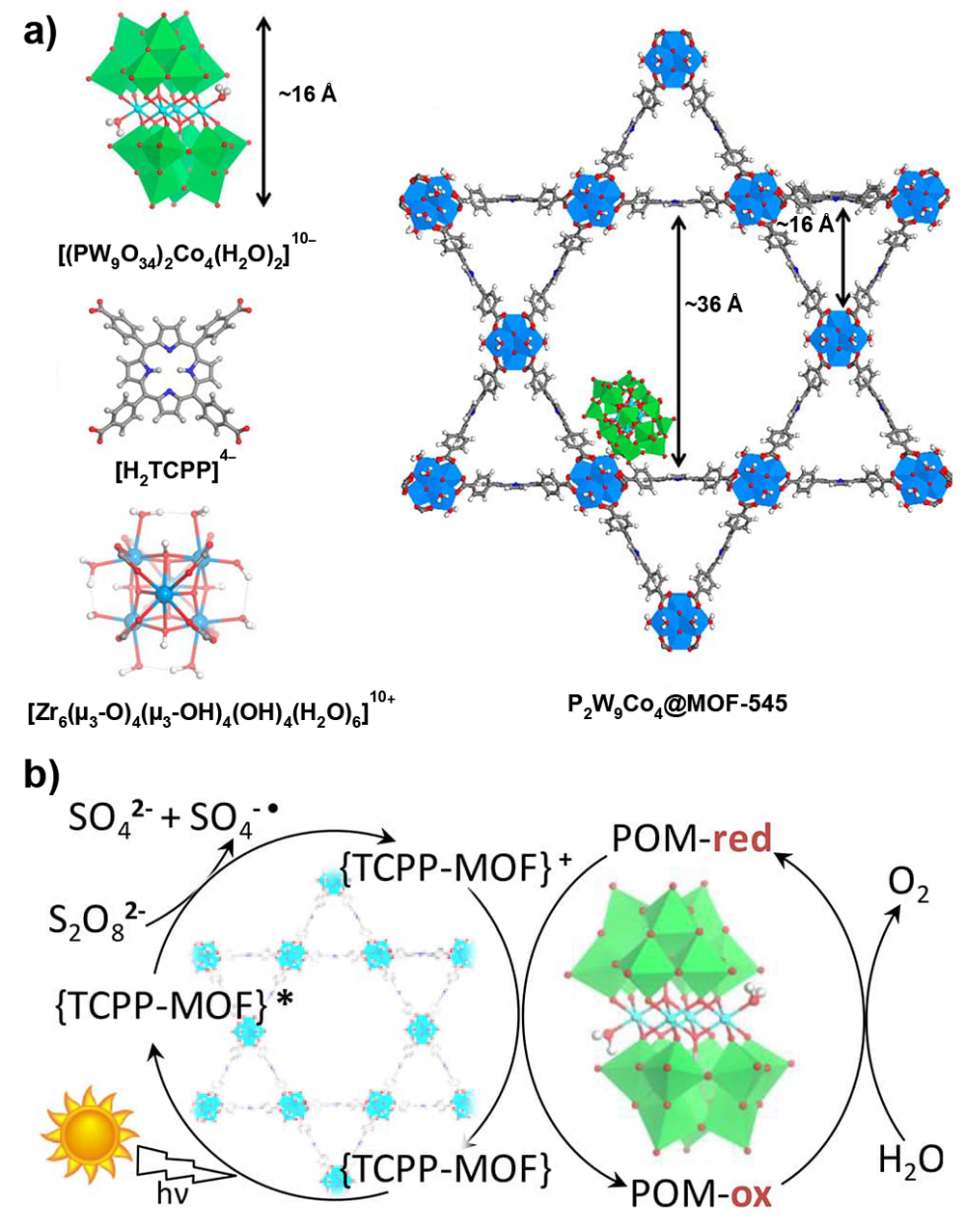
a) Structure of **MOF-545** with encapsulated P_2_W_18_Co_4_ POM water oxidation catalyst and b) proposed mechanism of water oxidation wherein initial photoexcitation of the MOF porphyrin linker is quenched by a sacrificial electron acceptor, followed by oxidation of the encapsulated POM. Reproduced with permission from reference (245). Copyright 2018 American Chemical Society.

In 2018, Paille et al.^245^ encapsulated the sandwich-type POM [(PW_9_O_34_)_2_Co_4_(H_2_O)_2_]^10–^ (P_2_W_18_Co_4_) inside the pores of **MOF-545** (also known as **PCN-222**) as shown in Figure 24a. Encapsulation was performed by soaking the pristine MOF in an aqueous solution of the P_2_W_18_Co_4_ POM, and relied on strong H-bond interactions between the POM and the Zr_6_ cluster SBU, as suggested by density functional theory (DFT) calculations. Photocatalysis experiments in the presence of the sacrificial electron acceptor Na_2_S_2_O_8_ demonstrated that the porphyrin linkers of **MOF-545** were able to act as PSs for POM oxidation (Figure 24b). Recycling experiments revealed similar initial water oxidation rates and only slightly decreased TONs using recovered POM@MOF. Post mortem X-ray diffraction (XRD), thermogravimetric analysis, energy-dispersive X-ray spectroscopy, and X-ray absorption near edge spectroscopy studies showed no noticeable difference between the material before and after catalysis, indicating that this strategy effectively stabilizes the POM against decomposition.

Subsequent work by the same group demonstrated that this POM@MOF material could be prepared as thin films on ITO electrodes, permitting electro- and photocatalysis experiments.^246^ The method of preparing the thin films had a significant impact on the experimental TONs, as electrophoretic deposition gave thicker films than simple drop casting. The authors stated that lower TONs in thicker films may be due to incomplete illumination of the deposited material, while transport limitation were not considered in the work. Analogous work by Das and co-workers^247^ showed greatly improved electrocatalytic stability of the K_6_[CoW_12_O_40_] Keggin POM toward water oxidation via encapsulation in the pores of **ZIF-8**.

In 2019, Hod and co-workers^248^ anchored the porphyrin-based ORR catalyst Hemin to the SBUs of **UiO-66** through solvent-assisted ligand incorporation. This process occurs at missing-linker defect sites at the SBUs that are filled by coordination of the carboxylate-containing Hemin. Accordingly, it is shown that the catalyst incorporation yield is increased upon increased number of such defect sites in the as-prepared **UiO-66**. The distribution of the Hemin catalysts is however not homogeneous throughout the crystal. Five different **UiO-66@Hemin** materials that differ in catalyst amount and spatial distribution have been prepared, and are shown to be good catalysts for the ORR. As shown by the same group in 2020, the ORR activity of the system can further be improved by axial coordination of 2-methylimidazole to the central Fe atom of the Hemin moiety.^249^

FeX (X = Br^–^, Cl^–^, AcO^–^, and BF_4_^–^) could be supported on a hexanuclear Zr_6_O_4_(OH)_4_ cluster that constituted the SBU of a mixed-linker MOF, containing nitro-quaterphenyl dicarboxylate and *p*-phenanthroline dibenzoate (PT).^250^ The PT-sites were used to assemble a [Cu(PT)(dppe)]^+^-type PS (dppe = 1,2-bis(diphenylphosphino)ethane) by a series of postsynthetic modifications. The resulting **FeX@Zr**_**6**_**-Cu** promotes the light-driven HER with TONs of up to 33 700 and TOFs of up to 880 h^–1^. Photocatalytic H_2_ evolution activities of **FeX@Zr**_**6**_**-Cu** were found to correlate with the lability of the counteranions X, consistent with the generation of open coordination sites at the single atom Fe centers by decoordination of labile X groups to facilitate the formation of Fe-hydride intermediates.

Zr_6_-cluster SBUs also served as the anchoring site for the attachment of transition metals for the HER. In one such report, diverse metal ions (Ni^2+^, Co^2+^, Cu^2+^, Ru^3+^) were bound to Zr_6_-oxo clusters of different MOFs in a microwave-assisted method. The introduction of the single metal sites was followed by hydroxylation, sulfidation, or further oxidations. Among the prepared materials, a Ni-loaded **UiO-66-NH**_**2**_, was shown to catalyze the light-driven HER in the presence of TEA.^251^

**Figure 25:**
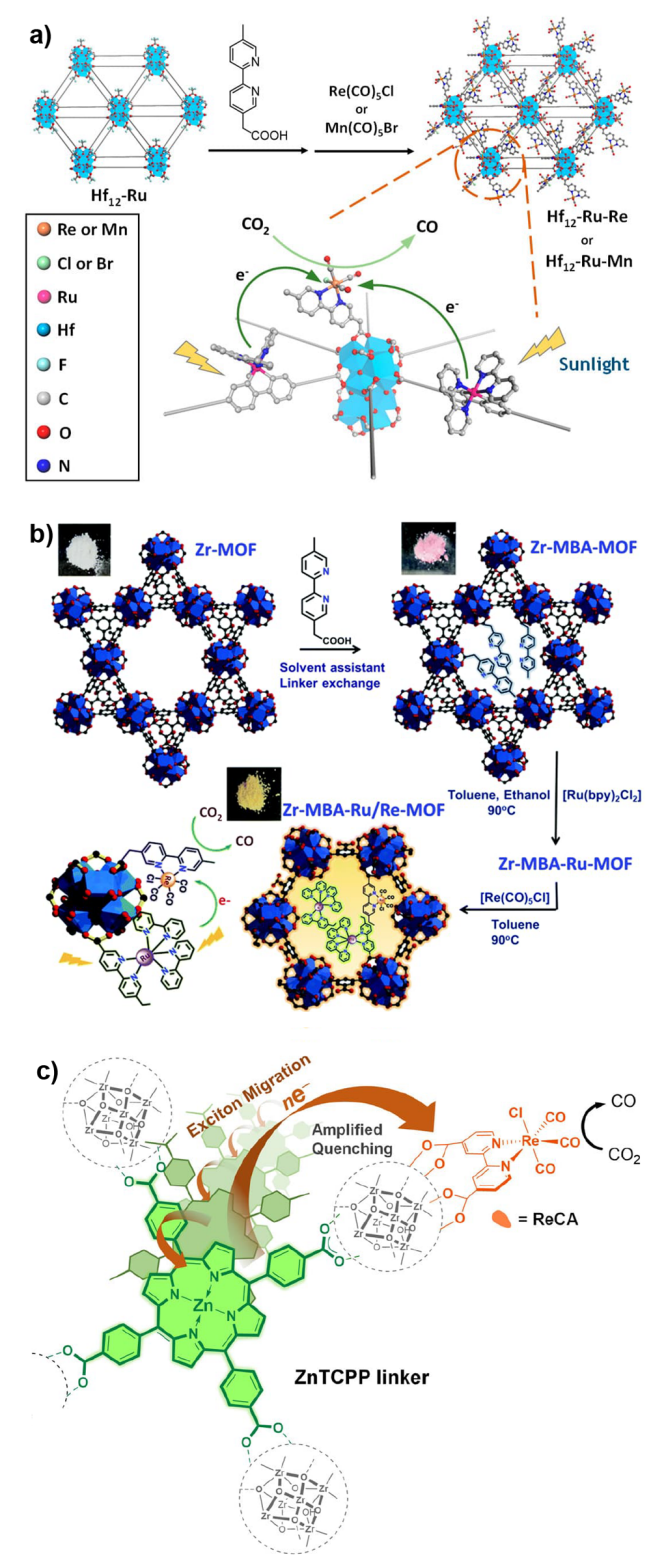
a) Postsynthetic monocarboxylic acid exchange of **Hf**_**12**_**-Ru** toward the synthesis of **Hf**_**12**_**-Ru-M** (M = Re, Mn) employed in the sunlight-driven CO_2_ reduction to CO. Reproduced with permission from reference (252). Copyright 2018 American Chemical Society. b) Synthetic pathway toward **Zr-MBA-Ru/Re-MOF** via postsynthetic exchange of formates with MBA ligands at the SBUs and the consecutive metalation of their *N*,*N*′-chelating sites with [Ru(bpy)_2_Cl_2_] and [Re(CO)_5_Cl], to afford a hybrid system with a Ru-based PS and a molecular Re catalyst for visible light-driven CO_2_ reduction. Reproduced with permission from reference (191). Copyright 2021 Royal Society of Chemistry. c) Depiction of the **PCN-222(Zn)/Re** system, enabling electron transport through the MOF to the surface-attached Re active sites for the photocatalytic reduction of CO_2_. Adapted with permission from reference (173). Copyright 2021 American Chemical Society.

Lin and co-workers^252^ described a novel photosensitizing MOL, **Hf**_**12**_**-Ru**, consisting of Hf_12_ SBUs and [Ru(bpy)_3_]^2+^-derived dicarboxylate linkers. **Hf**_**12**_**-Ru** was postsynthetically modified by replacing weakly coordinating trifluoroacetic acid units on the SBUs with MBA (2-(5′-methyl-[2,2′-bipyridin]-5-yl)acetic acid). The resulting **Hf**_**12**_**-Ru-MBA** was then metalated with [Re(CO)_5_Cl] or [Mn(CO)_5_Br] to afford the hybrid systems **Hf**_**12**_**-Ru-Re** and **Hf**_**12**_**-Ru-Mn**, respectively, bearing Ru-based PSs and catalysts for the light-driven CO_2_RR in the presence of electron donors (Figure 25a). From the systems studied, **Hf**_**12**_**-Ru-Re** exhibited the highest TON of 8613 under artificial visible light irradiation, and of 670 under ambient sunlight. The activity is facilitated by electron transfers from the photogenerated, reduced PS to the catalytic centers that are in close proximity of each other on the MOL skeleton.

Following a similar postsynthetic sequence, Karmakar et al.^191^ immobilized not only a CO_2_ reduction catalyst at the SBU but also a Ru-based PS. The chosen **Zr-MOF** (=**MOF-808**) was first postsynthetically modified through a solvent-assistant exchange of formates with MBA ligands at the SBUs. By consecutive postsynthetic metalation with [Ru(bpy)_2_Cl_2_] and [Re(CO)_5_Cl], both functional units were installed at the *N*,*N*′-chelating sites of the MBA ligands, to afford **Zr-MBA-Ru/Re-MOF** (Figure 25b). The photocatalytic CO_2_RR with the hybrid system was investigated and CO formation was established with a selectivity of >99%. Control experiments with **Zr-MBA-Re-MOF** and **Zr-MBA-Ru-MOF** separately employed as a photocatalyst showed only trace amounts of a CO_2_ reduction product, supporting the hypothesis that the close proximity between the PS and the catalyst within the MOF pores shortens the transport distances of the charge carriers. Interestingly, the photocatalysis experiments were conducted without an exogenous electron donor, implying that water may be the terminal electron donor.

Hu et al.^253^ described in 2019 the efficient photoreduction of CO_2_ to HCOOH and CO by 2D light-harvesting MOLs. The parent MOL (**Zr-TCBPE-MOL**; TCBPE = tetrabenzoatetraphenylethylene) displays 12 available sites on the Zr_6_O_4_(OH)_4_ SBUs whereof only four are connected to TCBPE linkers, leaving the others accessible for other carboxylate ligands. By postsynthetic modification, the H_2_bpydc-anchored (H_2_bpydc = 2,2′-bipyridine-5,5′-dicarboxylic acid) catalytic complexes H_2_bpydc-Re(CO)_3_Cl and [H_2_bpydc-IrCp*OH]NO_3_ can be introduced by coordinating their dicarboxylate moieties to the MOL’s SBUs. The Ir or Re compounds can either bridge two SBUs within one MOL or interconnect two MOLs. Employing **Zr-TCBPE-MOL-Ir** as a photocatalyst, CO_2_ was reduced to both formic acid and formaldehyde. In comparison, the homogeneous counterpart under the same reaction conditions generated significantly less product.

Recently, Choi et al.^173^ described the porphyrinic MOF **PCN-22(Zn)** onto which the prototypic CO_2_RR catalyst (bpy-4,4′-dc)Re^I^(CO)_3_Cl was installed via carboxylate anchoring groups at SBUs close to the crystal surface. Upon illumination, the synthesized **MOF-Re(I)** hybrid was able to produce CO over 59 h with BIH (1,3-dimethyl-2-phenyl-1,3-dihydrobenzimidazole) as electron donor, without significant loss in catalytic activity. The observed enhanced catalytic performance of the **MOF-Re(I)** was ascribed to efficient exciton migration between excited Zn-porphyrins and electron transfer to the Re-based active centers (Figure 25c).

#### 3.1.3 Catalysts as Linkers

Molecular catalysts can be used as integral linkers in MOFs if they contain ligands that can be synthetically modified to introduce multiple anchoring groups. The location of each catalyst in such materials is known more precisely than in the previous section in which the catalysts were hosted in the MOF pores attached to the MOF matrix through some sort of tether. The molecular catalysts in this section can also be seen as metallo-linkers and can either be included in the solvothermal syntheses or introduced into the MOF by postsynthetic strategies. In the former cases, the catalytically active metallo-linker may be the sole linker that the MOF is composed of, or complemented by a second, perhaps sterically less demanding colinker to produce mixed-linker MOFs. The most frequent postsynthetic methods include metalation of MOF-linkers with suitable binding pockets, or postsynthetic linker exchange in which inert linkers from the pristine MOF are exchanged by catalytically active metallo-linkers.

##### 3.1.3.1 2,2′-Bipyridine (bpy)-Related Work

A significant amount of work in this section is based on **UiO-67**, as its constituting biphenyldicarboxylate linker can be exchanged by bipyridine dicarboxylate that is identical in length but can host a variety of metal fragments. This strategy is particularly advantageous in context of the OER, as decomposition of, for example, ruthenium-based WOCs has long been attributed to intermolecular pathways.^31^ Consequently, several groups have targeted the site isolation of ruthenium moieties as metallo-linkers inside MOFs.^254−256^ Resonance Raman analysis found that while signals for dimeric ruthenium species were observed after chemical oxidation of a homogeneous ruthenium catalysts, no such signals were observed under the same chemically driven catalysis conditions with the analogous MOF-immobilized catalyst.^255^ Other bpy linker-based WOCs incorporated into MOFs with improved stability include **UiO-67** decorated with iridium bipyridine units^257^ and a **UiO**-type MOF with expanded linkers using iridium catalytic centers.^114^ Taken together, these reports demonstrate promising catalytic stability and recyclability of the MOF-based hybrid materials.

Chamber et al.^258^ realized the first photosensitized rhodium-based MOF for CO_2_ reduction with a high selectivity for formate formation. In this report, a catalytically active Rh-based half-sandwich complex Cp*Rh(bpydc)Cl_2_ was introduced into **UiO-67** via postsynthetic linker exchange to form Cp*Rh@UiO-67 that was employed in the CO_2_RR together with homogeneous Ru(bpy)_3_Cl_2_ as PS. Even though catalytic activities of the homogeneous and heterogeneous systems were found comparable, the MOF-based system offered the advantage of higher stability and selectivity for formate production, and could also be recycled without loss of activity. Similarly, Liao et al.^259^ described a MOF platform with incorporated Ru- and Rh-based half sandwich units for CO_2_ reduction and H_2_ evolution. The presented MOFs **RuCl@UiO**, **RuOH**_**2**_**@UiO**, **RhCl@UiO**, and **RhOH**_**2**_**@UiO** were synthesized via postsynthetic linker exchange of the **UiO-67** parent system with the complexes [Ru(Cy*)(bpydc)Cl]Cl·H_2_O (H_2_RuCl, Cy* = *p*-cymene), [Ru(Cy*)(bpydc)(OH_2_)](NO_3_)_2_ (H_2_RuOH_2_), [Rh(Cp*)(bpydc)Cl]Cl_2_·H_2_O (H_2_RhCl), and [Rh(Cp*)(bpydc)(OH_2_)](NO_3_)_2_·1.5H_2_O (H_2_RhOH_2_), respectively. In combination with Ru(bpy)_3_Cl_2_ and the sacrificial electron donors TEOA or *N*,*N*-dimethylaniline (DMA), all of the modified MOFs possess the ability to produce H_2_ and reduce CO_2_ into CO and HCOO^–^. Of the systems investigated, **RhOH**_**2**_**@UiO** displayed the best photocatalytic HER activity while the Ru-based MOF catalysts showed better performance in terms of CO_2_ reduction selectivity against H_2_ evolution. A long-term hydrogen evolution lasting for 174 h without significant decrease in efficiency was achieved in the **RhOH**_**2**_**@UiO-DMA** system, illustrating the power of the MOF matrix to stabilize the structural integrity of molecular catalysts for extended periods of time under turnover conditions. Overall, accommodating the molecular catalysts in the MOF platform showed superior and long-term photocatalytic stability as well as higher TONs than corresponding homogeneous catalysts for both the HER and the CO_2_RR.

In 2020, Benseghir et al.^260^ demonstrated the coimmobilization of the Keggin-type POM PW_12_O_40_^3–^ and the catalytically active complex Cp*Rh(bpydc)Cl_2_ in **UiO-67**. The POM was encapsulated inside the pores of the MOF scaffold in a ship-in-the-bottle approach, while the Rh-based catalytic complex was introduced by subsequent postsynthetic linker exchange to derive the composite **(PW**_**12**_,Cp*Rh)@UiO-67. The material was evaluated for photocatalytic CO_2_ reduction toward formate and hydrogen. In comparison with the POM-free Cp*Rh@UiO-67 the POM-assisted composite showed a doubled formate production yield. Prepared as drop-casted thin films on ITO plates, **(PW**_**12**_,Cp*Rh)@UiO-67 was investigated under photocatalytic conditions to elaborate its recyclability. During the first and second run of irradiation, only slight activity decreases of 8% and 9% were observed, respectively. Compared to analogous experiments of **(PW**_**12**_,Cp*Rh)@UiO-67 as suspension, the ITO immobilized system showed higher TONs of 175 versus 14.6 over 3 h, which was attributed to more efficient illumination of the crystallites in the thin film.

**Figure 26:**
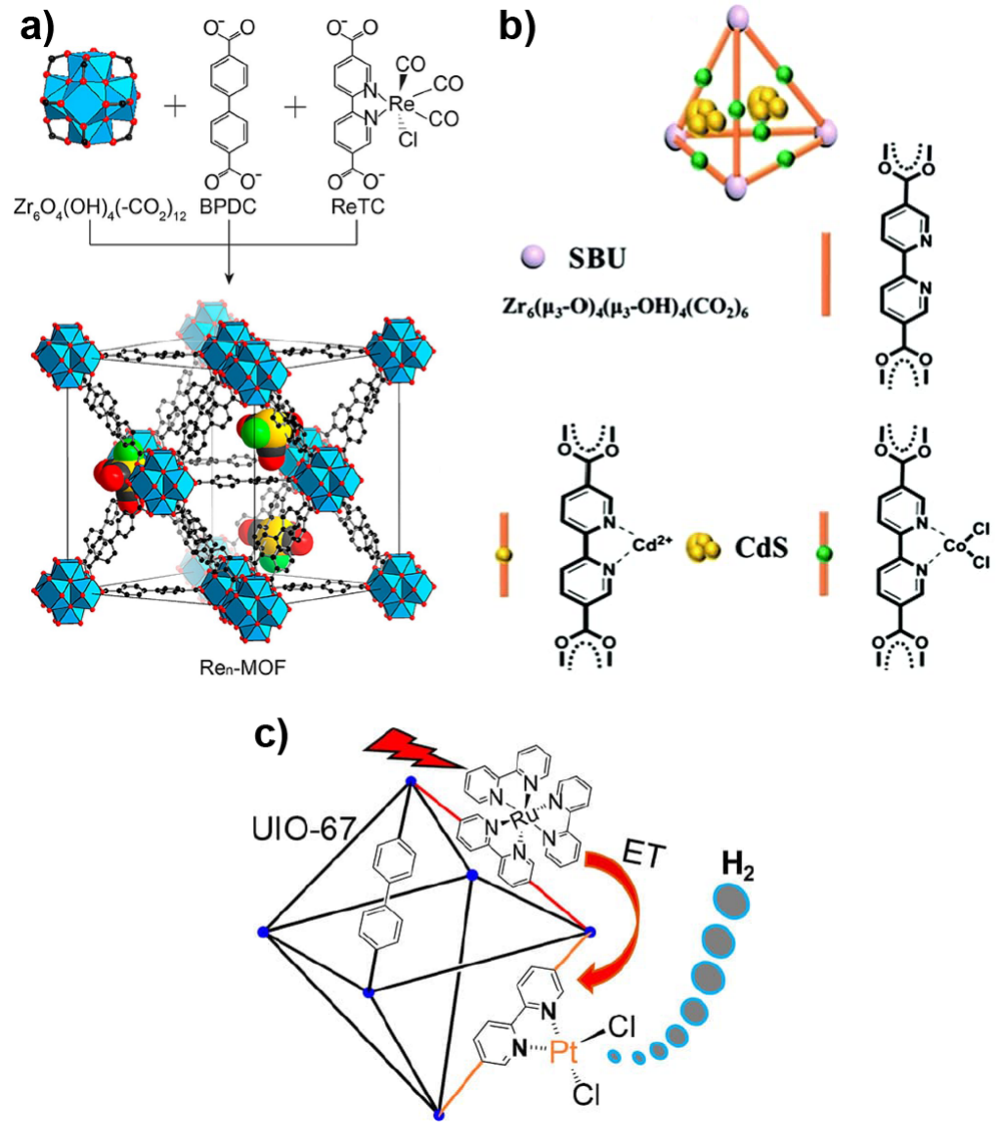
a) Combination of Zr_6_O_4_(OH)_4_(−CO_2_)_12_ SBUs with bpdc and ReTC linkers to form **Re**_**n**_**-MOF**. Depicted is the structure of **Re**_**3**_**-MOF** identified from single-crystal X-ray diffraction. Atom labeling: C, black; O, red; Zr, blue polyhedra; Re, yellow; Cl, green. H atoms are omitted for clarity. Adapted with permission from reference (197). Copyright 2017 American Chemical Society. b) Depiction of the ternary **CdS/UiO-bpy/Co** composite, obtained through a stepwise modification of a **UiO-bpy** framework with Cd(CH_3_COO)_2_·2H_2_O in DMSO to form **CdS/UiO-bpy**, followed by the metalation of the free bpy sites with CoCl_2_ in THF. Adapted with permission from reference (261). Copyright 2018 Royal Society of Chemistry. c) Example of catalyst and PS coimmobilization within a **UiO-67**-based framework as metallo-linker for the light-driven HER. Reproduced with permission from reference (263). Copyright 2018 American Chemical Society.

In 2017, Yaghi and co-workers^197^ described the plasmon-enhanced photocatalytic CO_2_-to-CO conversion by a **Re**_**n**_**-MOF**-coated Ag nanocube hybrid system. The Re^I^(CO)_3_(bpydc)Cl acts as the photocatalyst and was incorporated into a **UiO-67** framework to form a series of **Re**_***n***_**-MOF** with *n* = 0 to 24 complexes per unit cell (Figure 26a). Of the materials prepared, **Re**_**3**_**-MOF** showed the highest photocatalytic activity in the presence of TEA as an electron donor. Further, **Re**_**3**_**-MOF** was coated onto plasmonic Ag nanoparticles to afford **Ag⊂Re**_**3**_**-MOF**, which showed a 7-fold improved photocatalytic CO_2_-to-CO conversion activity compared to **Re**_**3**_**-MOF**. This activity enhancement was explained by electric fields at the surface of the Ag nanocubes and its influence on the spatially confined catalytically active Re centers.

In 2018, Chen et al.^261^ reported on a MOF composite, **CdS/UiO-bpy/Co**, with an inorganic CdS semiconductor quantum dot and the redox catalyst Co(bpydc)Cl_2_. The composite was synthesized by the stepwise postsynthetic modification of **UiO-bpy** to introduce the functional components (Figure 26b). The ternary system was described to feature good CO_2_ adsorption properties and facilitate efficient charge separation and diffusion. It was also shown to promote the light-driven reduction of CO_2_ to CO in the presence of TEOA as an electron donor.

The bpydc linker in **UiO-67(bpy)** can not only be used as an integral part of a molecular catalyst but also as a ligand of archetypical [Ru(bpy)_3_]^2+^-type PSs. Such a strategy places the photosensitizing and catalytic units in close proximity and may promote fast light-induced electron transfer. In this context, we encourage the reader to revisit the section in chapter 2 that also discusses potential drawbacks of such spatial arrangements that can, for example, promote back electron transfers. Examples of systems in which PS and catalysts are incorporated as metallo-linkers include **Ru-Pt@UiO-67** (Figure 26c), which contains both a PS and a Pt-based HER catalyst.^262^ The system was later studied by time-resolved techniques to deduce light-induced charge transfer kinetics and the detection of the catalytically relevant Pt^I^ species.^263^ A related **Co-Ru-UiO-67(bpy)** system follows the same design principles with the noble metal-based catalyst having been replaced by a Co complex.^264^ Another bipyridine-embedded **UiO**-type MOF, namely, **Pt-n_Ir_BUiO** with an incorporated iridium-based PS was shown to be active in the light-driven HER for weeks.^265^ The longevity was ascribed to bpydc linkers in close vicinity to the HER catalyst and the PS which are available to recapture metal fragments that may have temporarily decoordinated from their original bpydc linker. As a result, the system has a certain self-healing capacity, and colloid formation that was observed in homogeneous reference experiments was not observed in the **Pt-n_Ir_BUiO** MOF.

Besides the aforementioned transition-metal complexes comprising Co,^264,266^ Ru,^254−256,259,267^ Rh,^258,259^ Ir,^114,257^ and Pt^262,263,265^ active sites, multiple MOF systems with bpy-containing CO_2_RR catalysts of the general formula M(L)(CO)_3_X, where M is a transition metal, L a bidentate bipyridine-based linker, and X a halide, have been reported. As one of the most well-documented deactivation pathways for this type of catalysts is the bimolecular dimerization,^268,269^ site isolation of the catalysts as integral linkers in MOFs is highly motivated. Among other materials, Lin and co-workers^257^ were first to report a **UiO-67** system with an integrated Re^I^(CO)_3_(bpydc)Cl catalyst. The resulting hybrid MOF catalyzed the light-driven reduction of CO_2_ to CO in the presence of TEA with a TON of 10.9, three times higher than that of the homogeneous complex. Similarly, Fei et al.^270^ reported the synthesis of a mixed linker **UiO-67-bpydc** MOF with an equimolar ratio of H_2_bpydc and H_2_bpdc (biphenyl-4,4′-dicarboxylic acid). Via postsynthetic metalation, the earth-abundant, but thermally unstable, molecular catalyst Mn(bpydc)(CO)_3_Br was incorporated into the robust MOF platform, to achieve site-isolated catalytically active moieties. Furthermore, the **UiO-67** matrix enhanced the stability of the Mn active sites, enabling their reuse for up to three cycles. In combination with a ruthenium-based PS in solution and 1-benzyl-1,4-dihydronicotinamide as a sacrificial reductant, **UiO-67-bpy-Mn(bpy)(CO)**_**3**_**Br** represents an efficient catalytic system for the selective reduction of CO_2_ to formate under visible light irradiation, reaching a TON for formate production of 50 over 4 h and a selectivity of 96%. Also the corresponding **UiO-67-Re-30%** system with an incorporated Re(CO)_3_(bpydc)Cl catalyst was reported. In this MOF, the Re complex acts as both a PS and a catalyst for the reduction of CO_2_ to CO in the presence of an electron donor.

In 2018, Deng et al.^271^ presented the postsynthetic metalation of **MOF-253** (Al(OH)(bpydc)) with [Re(CO)_5_Cl] to produce a photocatalytically active **MOF-253-Re(CO)**_**3**_**Cl** system. In addition to superior catalytic performance over the homogeneous counterpart, the synthesized MOF was found to selectively reduce CO_2_ to formate instead of CO. Trying to enhance the light absorption of the formed heterogeneous system, further treatment with [Ru(bpy)_2_Cl_2_] afforded **Ru-MOF-253-Re**. Due to the augmented visible light absorption, **Ru-MOF-253-Re** showed enhance photocatalytic CO_2_RR performance. While the TON of the MOF is comparable to Ru–Re supramolecular structures based on covalent bonds, the assembly of a hybrid Ru–Re system within a MOF is synthetically easier and generally more modular.

Going beyond Re and Mn-based bpydc catalyst systems, Sun et al.^267^ presented the incorporation of a Ru-based catalyst into the bipyridine linkers of **MOF-253** to afford **MOF-253-Ru(CO)**_**2**_**Cl**_**2**_. Even though **MOF-253-Ru(CO)**_**2**_**Cl**_**2**_ itself is capable to reduce CO_2_ when illuminated, its performance can be enhanced by the coimmobilization of a Ru(bpy)_2_(bpydc)^2+^ PS, introduced into **MOF-253-Ru(CO)**_**2**_**Cl**_**2**_ by a second postsynthetic metalation strategy, to improve light absorption. The optimum activity was observed when the molar ratio of PS:catalyst was 1:1. By doing so, the amount of formate production was increased by a factor of 12, while the amount of produced CO was unchanged. The TON for formate formation was increased from 0.3 for the homogeneous catalyst Ru(bpydc)(CO)_2_Cl_2_, over 2.9 for the nonsensitized MOF, to 35.8 for the sensitized **MOF-253-Ru(CO)**_**2**_**Cl**_**2**_. In 2019, Liu et al.^266^ described photocatalytic syngas production by the **(Co/Ru)n-UiO-67(bpydc)** framework, assembled in a two-step postsynthetic metalation procedure. The thereby obtained MOF platform enabled fast electron transfer from the PS to the molecular cobalt catalyst, and exhibited a 29.2-fold increase in syngas production yield compared to the homogeneous reference system.

**Figure 27:**
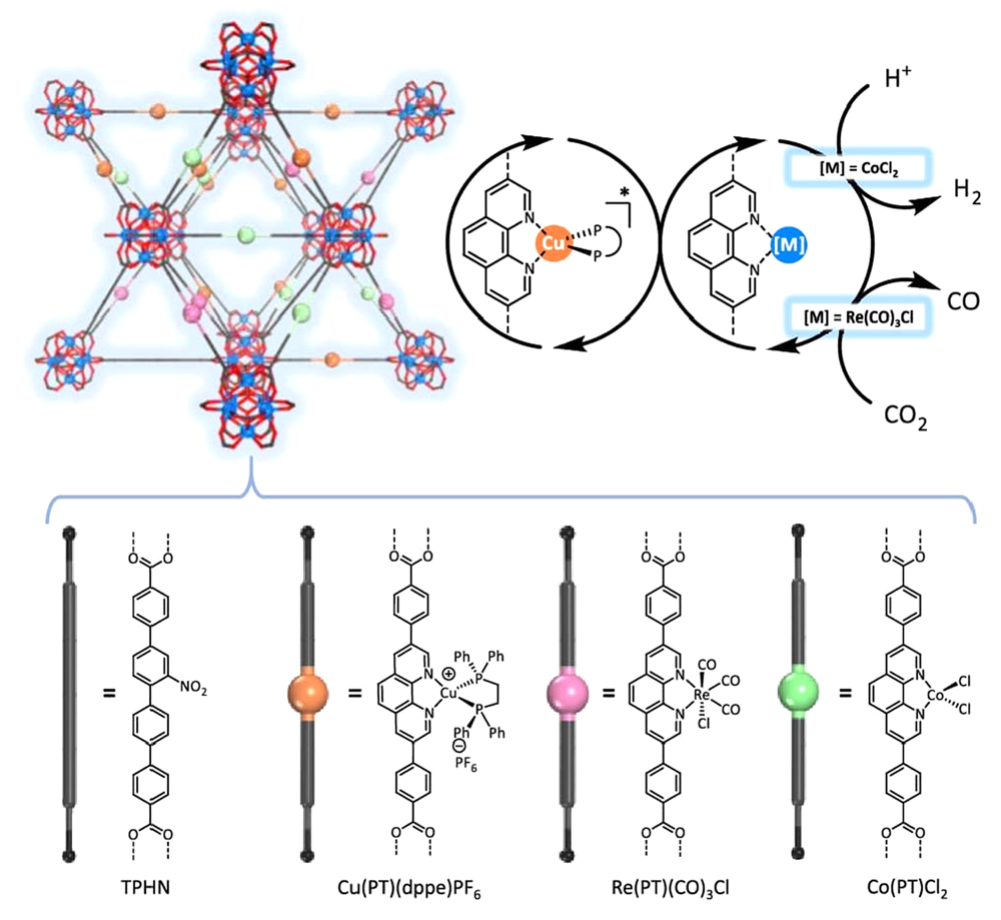
Schematic representation of the framework composition of **mPT-Cu/Co** and **mPT-Cu/Re**, utilized in the HER or CO_2_RR, respectively. Adapted with permission from reference (273). Copyright 2020 American Chemical Society.

Even though MOF systems with bpydc as linkers are versatile and suitable to anchor a variety of molecular catalysts, the pores of the MOFs are relatively small, hindering diffusion and high concentrations of sterically demanding active sites. To prevent such performance limitations, elongated linkers can be employed to build isoreticular systems with enlarged pores. In 2016, Lin and co-workers^272^ described a **UiO**-type MOF consisting of the elongated metallo-linker Re(CO)_3_Cl(bpydb) (bpydb = 4,4′-(2,2′-bipyridine-5,5′-diyl)dibenzoic acid). In contrast to the mixed linker systems described by the same group in 2011,^257^ the elongated linker allowed the synthesis of a MOF that is exclusively formed from the catalytically active metallo-linker. After 6 h of photocatalytic CO_2_ reduction in acetonitrile, the **Re-MOF** showed a TON for CO of 6.44, roughly six times higher than that of the corresponding homogeneous complex. Employing phenanthroline instead of bpydb, Lin and co-workers^273^ further reported the synthesis of two mixed-linker phenanthroline-based (mPT) frameworks, **mPT-Cu/Co** and **mPT-Cu/Re**, including cuprous PSs and molecular Co or Re catalysts, suitable for photocatalytic HER and CO_2_RR, respectively. The catalytically active systems were derived through postsynthetic metalation of the parent **mPT-MOF** platform, comprising Zr-based SBUs and H_2_TPHN (2″-nitro-[1,1′:4′,1″:4″,1‴-quaterphenyl]-4,4‴-dicarboxylic acid) and H_2_PT (4,4′-(1,10-phenanthroline-3,8-diyl)dibenzoic acid) linkers, with Cu(CH_3_CN)_4_PF_6_, dppe (1,2-bis(diphenylphosphino)ethane), and either CoCl_2_ or Re(CO)_5_Cl (Figure 27). Simultaneous arrangement of Cu PSs and the molecular Co or Re catalysts led to a multifunctional MOF platform with high photocatalytic activity. The **mPT-Cu/Co** MOF catalyzed the HER with a TON of 18 700, while **mPT-Cu/Re** catalyzed the CO_2_RR with a TON of 1328, nearly 2 orders of magnitude higher than the TONs of their homogeneous counterparts.

##### 3.1.3.2 Porphyrin/Macrocycle-Related Works

MOFs with porphyrin-derived linkers, most prominently H_2_TCPP (4,4′,4″,4‴-(porphyrin-5,10,15,20-tetrayl)tetrabenzoate)), have been prepared relatively early, and considering the known catalytic activity of homogeneous porphyrins to catalyze reactions of energy relevance, TCPP-containing MOFs have early moved to the center of attention. **Al-TCPP**, that is, (AlOH)_2_H_2_TCPP, in which infinite Al(OH)O_4_ chains are interconnected by porphyrin linkers into a 3D microporous framework, was platinated at the porphyrin sites.^153^ The resulting **Al-TCPP-Pt** is an efficient catalyst for the light-driven HER, with the Pt^II^-porphyrin linkers acting as both PS and HER catalyst in the presence of a reductive quencher. The activity of **Al-TCPP-Pt** is superior to that of a reference system consisting of Pt nanoparticles that are stabilized by the same sensitizing **Al-TCPP**. The authors argue that the difference in activity stems from the site isolation of the Pt centers and associated maximized atom utilization for catalysis as compared to the nanoparticle system. Another example of a Pt^II^TCPP HER catalyst that was used as linkers in conjunction with Ti-oxo clusters as metal nodes was reported as the HER catalyst under illumination in the presence of ascorbic acid.^274^

In 2017, Fateeva and co-workers^275^ investigated the electrocatalytic ORR performance of **Co-Al-PMOF**, consisting of Co-porphyrin linkers interconnected by aluminum oxide SBUs. The authors suggested that an increasing porosity to promote oxygen and product transport inside micropores does not always imply more efficient reduction chemistry. The authors stated that the distance between the catalytic centers is more pivotal, and influences whether the reaction proceeds via a 2- or a 4-electron pathway. In related work, **PCN-224(Co)**, a MOF consisting of Zr_6_ clusters and Co-TCPP linkers, was deposited on carbon substrates as an ink together with surface-activated multiwalled CNTs.^276^ The composite material was shown to be an active electrocatalyst for both the ORR and OER.

**Figure 28:**
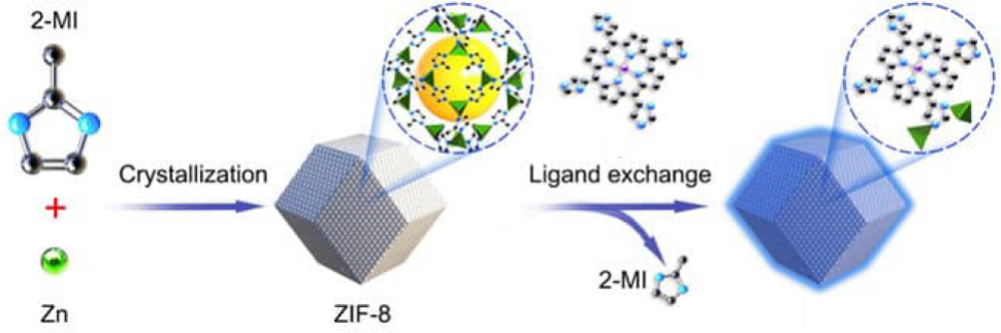
Grafting of Co tetra(imidazolyl)porphyrin onto the Zn-based **ZIF-8** framework via a ligand exchange. Adapted with permission from reference (277). Copyright 2021 WILEY-VCH Verlag GmbH & Co.

In 2021, Liang et al.^277^ successfully grafted cobalt porphyrins as the active sites on a MOF surface through ligand exchange. The generated hybrid materials showed improved ORR activity with substantial anodic shift (>70 mV) of the half-wave potential compared to the molecular electrocatalysts without covalent immobilization. The authors described both, **ZIF-8**, a framework mainly selective for the 2e^–^ ORR toward hydrogen peroxide, and **ZIF-67**, its analogue, showing higher selectivity for the 4e^–^ ORR reducing H_2_O_2_ further to H_2_O (Figure 28). Control experiments with cobalt-free porphyrins grafted on **ZIF-8** revealed that the Co porphyrin linker is the catalytically active site. To demonstrate the practical potential of the prepared porphyrin@MOF hybrids, they were employed as electrode catalysts in Zn–air batteries, exhibiting performances comparable to that of Pt/C.

In 2013, Liu et al.^278^ investigated the CO_2_ adsorption and activation properties of the unmetalated (S_P_) and Cu^2+^-metalated (S_Cu_) version of a porphyrin-based MOF. Compared to S_P_, S_Cu_ showed both enhanced performance in CO_2_ capture and photocatalytic conversion of CO_2_ to methanol. Thereby the product evolution rate catalyzed by S_Cu_ was about 7 times higher than that of S_P_. FTIR measurements revealed that the CO_2_ adsorption and its activation over Cu^2+^ played a crucial role in the conversion of CO_2_. Similarly, Zhang et al.^279^ demonstrated the incorporation of Co^II^ and Zn^II^ cations into the stable scaffold of **MOF-525**. The authors claimed that the presence of single atoms within the TCPP ligands of **MOF-525-Co** and **MOF-525-Zn** enhanced their photoinduced electron–hole pair separation efficiency significantly, achieving long-lived electrons able to be transferred to the catalytically active metal centers for the CO_2_RR to CO and CH_4_.

In 2019, Huang et al.^280^ presented the synthesis of **MCF-55**, an analogue of **MOF-525**, both of which are composed of Zr_6_O_4_(OH)_4_ SBUs but differ in their constituting linkers. While the latter is based on TPCC linkers, the former contains H_4_tactmb (1,4,7,10-tetrazazcyclododecane-*N*,*N*′,*N*″,*N*‴-tetra-*p*-methylbenzoic acid) azamacrocycle linkers. Both MOFs were postsynthetically metalated to **MCF-55-M** and **MOF-525-M** (M = Co, Ni), and the resulting materials investigated for their catalytic activity for the light-driven CO_2_RR in the presence of [Ru(phen)_3_]Cl_2_ (phen = phenanthroline) and TEOA. An interesting feature of the H_4_tactmb-M (M = Co, Ni) linker is that its methylene groups create a hydrophobic microenvironment around the metal active sites, resulting in repulsive forces to H_2_O and enhanced CO_2_ to CO activity. **MCF-55-Co** displayed a near-quantitative selectivity for CO and a TOF significantly larger compared to that of the TCPP-based **MOF-525-Co**(279) that lacks the hydrophobic surroundings. Owing to the stronger CO_2_ affinity of Ni compared to Co, **MCF-55-Ni** showed an even higher TOF that was furthermore constant for five consecutive runs of 10 h each.

Hod et al.^281^ described the postsynthetic metalation of electrophoretically deposited **MOF-525** (Zr_6_O_4_(OH)_4_(H_2_TCPP)_3_) thin films on conductive FTO surfaces to generate the catalytically active redox-conductive **Fe_MOF-525** (Zr_6_O_4_(OH)_4_(FeTCPP)_3_). This approach afforded a high surface coverage of heterogenized catalyst for the reduction of CO_2_ to CO, where the metalloporphyrins act as catalysts and redox-hopping channels to distribute charge carrier to active sites, not directly linked to the electrode. Throughout the catalysis, a mixture of CO and H_2_ in about equal amounts was generated with a Faradaic efficiency (FE) of ∼100%.

Sadeghi et al.^282^ investigated the electrocatalytic performance of the porphyrin-based framework **Zn/PMOF**, synthesized from Zn(NO_3_)_2_·6H_2_O and H_2_TCPP, to afford the metalated ZnTCPP linkers within the framework. **Zn/PMOF** was employed in the light-driven CO_2_RR in the presence of H_2_O vapor as a sacrificial electron donor under UV−vis light. During 4 h of illumination, 10.43 μmol CH_4_ was produced with 0.3 g of **Zn/PMOF**, outperforming bare ZnO by 80.6% under the same conditions but still giving much less than one CH_4_ per pore unit.

In 2020, Zhou et al.^283^ reported the synthesis of a Cu-based porphyrinic MOF for efficient and selective CO_2_ reduction toward hydrocarbons. The material was prepared from CuTCPP interconnected by Cu paddle wheel dimers as SBUs. The Cu-based MOF displayed a FE of 73.6% and a current density of 7.5 mA cm^–2^ at −1.4 V vs RHE (reversible hydrogen electrode) for the production of methane and ethylene. These products are exclusively formed as a function of MOF incorporation of the catalyst, as control experiments with the homogeneous Cu-porphyrin catalyst generated mainly CO as the major product. In the MOF, CO was found to be an intermediate for the generation of hydrocarbons. The porous MOF architecture with abundant copper-based active sites enables not only a high CO_2_ absorption but also confines the CO intermediates spatially for the selective reduction into hydrocarbons, revealed by finite-element simulations.

In related work on multiple Cu environments, Gu and co-workers^284^ investigated the electrocatalytic CO_2_RR with Cu_2_(COO)_4_ paddle wheel-based Cu-porphyrinic MOF nanosheets (**Cu**_**2**_**(CuTCPP)**) and compared their performance to CuO, Cu_2_O, Cu, CuTCPP, and the CuO/CuTCPP composite. **Cu**_**2**_**(CuTCPP)** was exposed to potentials between −1.40 V and −1.65 V vs Ag/Ag^+^, and formate and acetate were found to be the predominant products, accompanied by small amounts of CO and CH_4_, as well as H_2_. At an applied potential of −1.40 V, formate and acetate were detected with an FE of 28.1% and 11.6%, respectively, which increased to 61.5% and 12.3%, respectively, at −1.55 V. It was found that the catalyst’s cathodic current density decreased rapidly during the first hour and stabilized thereafter at 4.5 mA cm^–2^, indicating cathodized restructuring of the catalyst. The structural changes at −1.55 V were observed by XRD, scanning electron microscopy, transmission electron microscopy, X-ray photoelectron spectroscopy (XPS) and FTIR experiments, revealing that the Cu-paddle wheel nodes probably changed to CuO, Cu_2_O, and Cu_4_O_3_ under cathodization conditions. Despite of the restructuring, the material shows a vastly different product selectivity compared to Cu, CuTCPP, CuO, and Cu_2_O, which all showed predominantly HER activity.

Qin et al.^285^ described the ionic liquid-induced formation of the 2D Mn-MOF **[BMI]**_**2**_**(Mn[Mn(H**_**2**_**O)**_**2**_**-TCPP](H**_**2**_**O)**_**2**_**)** (BMI = 1-butyl-3-methylimidazolium), bearing high loading of Mn^II^-metalated porphyrin linkers interconnected by Mn nodes. **[BMI]**_**2**_**(Mn[Mn(H**_**2**_**O)**_**2**_**-TCPP](H**_**2**_**O)**_**2**_**)** exhibited a high MnTCPP loading of 67 wt % and was synthesized from the metal-free H_2_TCPP ligand, which was metalated with Mn^II^ during the MOF formation in the ionic liquid [BMI]Br. **[BMI]**_**2**_**(Mn[Mn(H**_**2**_**O)**_**2**_**-TCPP](H**_**2**_**O)**_**2**_**)** was shown to be a photocatalyst for the conversion of CO_2_ and water vapor to CH_4_ and CO in the gas–solid interface.

Huang et al.^286^ demonstrated the fabrication of a core–shell MOF@MOF structure in a one-pot synthesis by taking advantage of differing nucleation kinetics of the two MOFs **PCN-222-Ni** and **UiO-67-NH**_**2**_. Their difference in nucleation kinetics is due to the fact that linkers with high connectivity show faster nucleation rates than those with low connectivity. Consequently, **PCN-222-Ni** which consists of tetra-topic linkers nucleated first, while **UiO-67-NH**_**2**_ with ditopic linkers nucleated later, forming a shell around the already formed **PCN-222-Ni** to afford the **P@U** composite. The combination of the two MOFs in one composite material resulted in the formation of a Z-scheme heterojunction, where the electrons in **UiO-67-NH**_**2**_ can be excited and then transferred to **PCN-222-Ni**, which is catalytic for the reduction of CO_2_ to HCOOH. The **P@U** composite combines the crystallinity, robustness, and high porosity of the parent MOFs with an optimized band alignment, an enhanced photoresponse and an increased charge separation. Consequently, the **P@U** composite catalyzed CO_2_ photoreduction with a rate for formic acid formation that is higher than that of the parent MOFs **PCN-222-Ni** and **UiO-67-NH**_**2**_. In these experiments, no exogenous electron donor was added, implying that water was the terminal reductant.

In contrast to the aforementioned applications of metalloporphyrins as catalytically active sites, Yaghi and co-workers^287^ synthesized the catecholate framework **MOF-1992**, by connecting tetratopic cobalt phthalocyanin-2,3,9,10,16,17,23,24-octaol ligands with Fe_3_(−C_2_O_2_−)_6_(OH_2_)_2_ clusters. Owing to sterically accessible Co active sites and good charge transfer properties, **MOF-1992**-covered carbon black cathodes reached per-site TONs of 5800 over 6 h for the electrocatalytic reduction of CO_2_ to CO in water with a FE of 80%.

**Figure 29:**
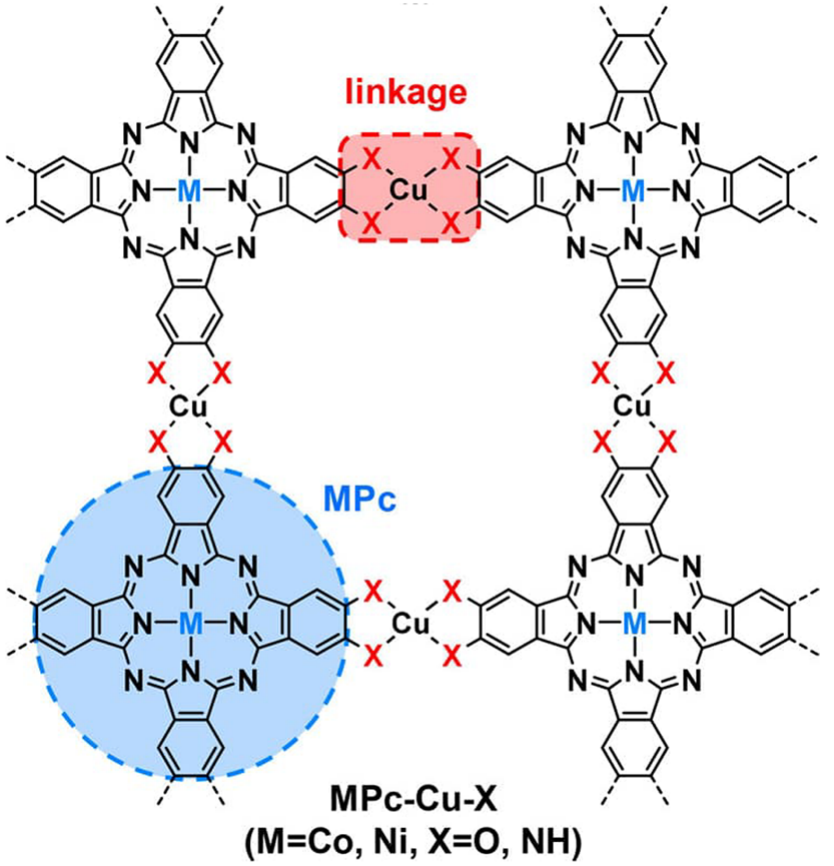
Series of **MPc–Cu–X** (M = Co, Ni, X = O, NH) MOFs containing CoPc and NiPc units connected by Cu bis(diimine) and Cu bis(dioxolene) linkages. Adapted with permission from reference (288). Copyright 2020 American Chemical Society.

A series of four structural analogues of conductive 2D MOFs **MPc–Cu–X** (M = Co, Ni and X = O, NH) comprising metallophthalocyanine (MPc) linkers and Cu nodes was prepared by Meng et al.^288^ (Figure 29). The authors investigated the influence of the metal center within the MPc and the heteroatom connectivity to the SBU on the electrochemical CO_2_RR activity. At an applied reductive potential, **CoPc–Cu–O** exhibited the highest selectivity toward CO formation with a FE of 85% when employed as a composite with carbon black (1:1 m/m) and a FE of 79% without any conductive additive. DFT studies revealed that CoPc-based and O-interconnected MOFs display lower activation energies to form carboxyl intermediates, which accounts for the higher activity and selectivity compared to the NiPc-based and NH-linked analogues.

In 2021, Qiu et al.^289^ investigated the 2D-MOF **PcCu-Cu-O** (Pc = phthalocyanine) with two different Cu environments for the electrochemical CO_2_RR. The MOF was synthesized from square-planar CuO_4_ nodes and PcCu-(OH)_8_ ((2,3,9,10,16,17,23,24-octahydroxyphthalo-cyaninato)copper) ligands. Applied as a powder on a GCE with Nafion binder, **PcCu-Cu-O** displayed a current density of 7.3 mA cm^–2^ at a potential of −1.2 V vs RHE in 0.1 M KHCO_3_, and a FE of 50% for C_2_H_4_ production. The FE for C_2_H_4_ production was twice of that of the homogeneous CuPc reference, and was attributed to the synergistic effect between the CuO_4_ and the CuPc units. According to the authors, CO is produced at the CuO_4_ SBU and then migrates to the C_2_H_4_-producing (CuPc) site, thereby lowering the energy barrier for the C–C dimerization. This is based on the facts that CuO_4_ has a high activity for the reduction of CO_2_ to CO and a lower *CO adsorption energy (16 kJ mol^–1^) compared to that of the CuPc (48 kJ mol^–1^), thereby being able to serve as CO source.

##### 3.1.3.3 Miscellaneous

The use of metal-free, purely organic molecular linkers as active sites for the OER inside MOFs has been claimed. Lu and co-workers^290^ prepared a lanthanide-based MOF using the linker triphenylene-2,6,10-tricarboxylic acid (H_3_TTCA) and demonstrated that this MOF was not only stable from pH 1–13 but could photoelectrochemically catalyze water oxidation. A catalytic cycle for the observed OER activity was, however, not reported. The homogeneous parent ligand H_3_TTCA does not evolve oxygen under similar conditions, which the authors suggest to be due to the instability of such organic catalysts when not site-isolated.

A 3D dithiolene-based MOF, **Cu[Ni(pdt)**_**2**_**]** (pdt = 2,3-pyrazinedithiolate), was evaluated as an electrocatalyst for the HER.^291^**Cu[Ni(pdt)**_**2**_**]** is constructed by nickel bis(pyrazine-2,3-dithiolate) complexes that are linked by 4-coordinated square planar Cu centers through the N atoms of the pyrazine moiety. **Cu[Ni(pdt)**_**2**_**]** forms a 3D structure with 1D square channels along the c direction. **Cu[Ni(pdt)**_**2**_**]** catalyzes the electrochemical HER from aqueous electrolyte solution at pH 1.3 with good current density after an initial activation step which involved the cleavage of Cu–N bonds upon applied negative potential in the acidic reaction medium. Computational results suggested that the copper centers were not involved in the HER, whereas the high H affinity of the pyrazine linker suggested a ligand-based HER mechanism.

Dou et al.^292^ presented the strategy of ligand doping to enhance the activity of MOFs for the electrocatalytic reduction of CO_2_. In this report, as-synthesized **ZIF-8** was first activated to generate open Zn sites which were subsequently coordinated by electron-donating 1–10-phenanthroline units to form **ZIF-8-LD** (LD = ligand doped). The electron-donating properties of the phenanthroline ligand positively impacts the catalytically active sp^2^ carbon atoms in the imidazole ligands, facilitating the activation of CO_2_ and its conversion to CO. Compared to the parent **ZIF-8** scaffold, **ZIF-8-LD** displayed enhanced electrocatalytic activity and a high selectivity for the production of CO with a FE_CO_ of 90.6%.

Molecular Ru-based water oxidation catalysts of the general type (tda)Ru(py)_2_ (tda = 2,2′:6′,2″-terpyridine-6,6′′-dicarboxylic acid) have been decorated with carboxylates at the axial pyridine groups, and used as linkers in Zr_6_O_4_(OH)_4_ SBU-based MOFs. Liseev at al.^293^ postsynthetically introduced a ditopic linker of this type (with 4-carboxylpyridine ligands) into a UiO-type MOF with ethynyldibenzoic acid linkers. The resulting mixed linker MOF was, however, a rather poor OER catalyst. In subsequent work, the same group reported a mixed linker analogue of NU-1000, prepared from the native H_4_TBAPy (H_4_TBAPy = 4,4′,4″,4‴-(pyrene-1,3,6,8-tetrayl)tetrabenzoic acid) linker and a tetratopic analogue of the Ru metallolinker with pyridine-3,5-dibenzoic acid ligands.^294^ In analogy to the homogeneous reference systems,^295^ the Ru complexes in the mixed linker MOF could be activated and engaged in electrocatalytic OER, albeit only with a FE of 37%. Interestingly, it was suggested that hole transport to Ru catalysts in the interior of the MOF crystals may be mediated by the TBAPy colinker, which, however, also engaged in irreversible oxidation processes, consistent with the low FE.

#### 3.1.4 Catalysts as SBUs

This section summarizes a class of catalytic materials in which the SBU is the catalytically active site. The SBU, and thus catalyst assembly, is enabled and stabilized by the coordinating linkers, and the overall stability of the MOF. In a way, this scenario resembles the situation in many metalloenzymes, where the active sites are held in place by amino acid side chains that are positioned in space at a defined distance by the surrounding peptide environment. The section is structured by the nature of the metals that the SBU is composed of.

##### 3.1.4.1 Titanium/Zirconium/Iron

Most examples in this section deal with MOFs that are built from high-valent Zr^IV^ or Ti^IV^ based SBU, and that engage in light induced electron transfer processes. Frequently, the linker is excited and oxidatively quenched by electron transfer to produce a reduced metal state at the SBU, which then drives the reductive chemistry. For a thorough discussion of possible photophysical processes in such systems, please see section 2.3 of this review. The organic linker that is oxidized in the process needs to be rereduced to enter another absorption/charge-separation cycle. In some examples, this is accomplished by the use of an organic electron donor, but in others, also water oxidation is proposed for this process. The latter is often implied in passing, and mechanistic details as to the water oxidation process are vague.

The oldest example in this section stems from 2012, when Li and co-workers^296^ described the light-driven reduction of CO_2_ to formate catalyzed by the Ti-containing MOF, **NH**_**2**_**-MIL-125(Ti)**. This MOF was synthesized by employing 2-aminoterephthalic acid (H_2_ATA) instead of benzene-1,4-dicarboxylic acid (H_2_BDC) that was used for the parent framework, **MIL-125(Ti)**. The 2-aminoterephthalate (ATA) linker is important for catalytic function as it shifts the optical absorption of the MOF into the visible region of the electromagnetic spectrum. Upon photoexcitation, Ti^4+^ is reduced to Ti^3+^ by the transfer of an electron from the excited ATA linker to the SBU, creating a charge-separated state that is able to reduce CO_2_. The electrons for this process are provided by a sacrificial electron donor, in the present case TEOA, that reduces that transiently oxidized ATA back to its ground state. The strategy of light-induced charge separation that involve ATA and derivatives thereof has been utilized in numerous subsequent studies, and the thereby generated charge-separated states employed for different transformations.

**NH**_**2**_**-MIL-125(Ti)** was also investigated for photocatalytic CO_2_ conversion as an emulsion with CO_2_/water under high pressures. In the presence of TEOA as electron donor, Luo et al.^297^ reported an increased efficiency of the CO_2_/H_2_O/**NH**_**2**_**-MIL-125(Ti)** emulsion for the conversion of CO_2_ to HCOO^–^, as compared to the same MOF system in conventional organic solvents. The authors identified the large interfacial area and outstanding mass transport under the high-pressure conditions as contributing factors for the improved activity.

**Figure 30:**
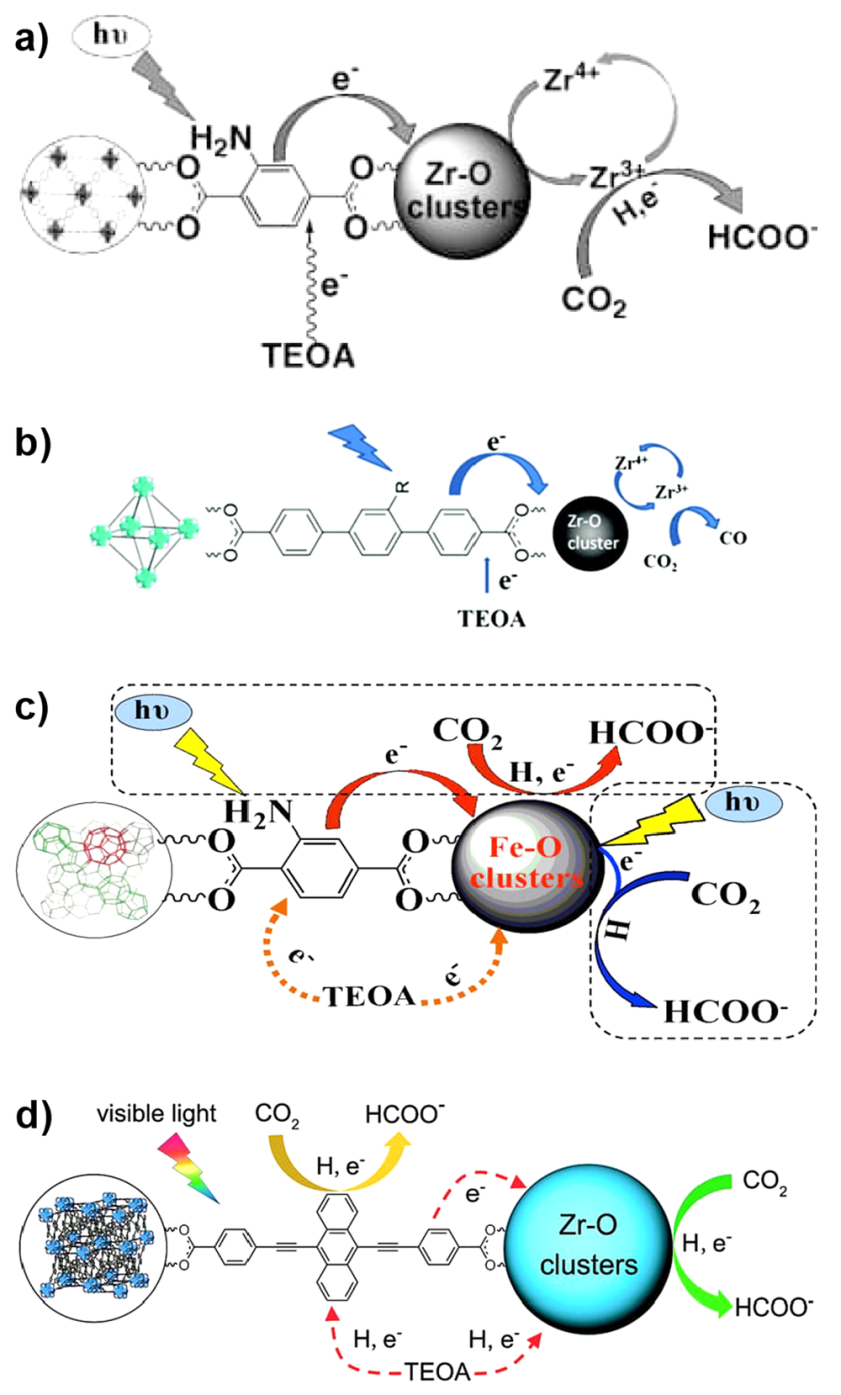
Suggested mechanisms for the photocatalytic reduction of CO_2_ by a) **NH**_**2**_**-UiO-66(Zr)**,^298^ b) **UiO-68-R** (R = F, CH_3_, OCH_3_),^301^ c) Fe-featured **NH**_**2**_**-MIL** frameworks,^302^ and d) **NNU-28**.^303^ Adapted with permission from the respective references. Copyright 2013 WILEY-VCH Verlag GmbH & Co, 2019 Royal Society of Chemistry, 2014 American Chemical Society, and 2016 Royal Society of Chemistry, respectively.

Further, Li and co-workers^298^ conducted photoluminescence studies on **NH**_**2**_**-UiO-66(Zr)** and suggested a photoinduced electron transfer from the excited ATA linker to the Zr-oxo clusters to form Zr^3+^ species. The latter species was proposed to drive the reduction of CO_2_ to formate (Figure 30a). Light absorption of the amino-linkers could be pushed further into the visible spectrum by partial substitution of ATA to DTA (2,5-diaminoterephthalate) linkers, resulting into a mixed **NH**_**2**_**-UiO-66(Zr)** framework with improved photocatalytic activity. Two years later, the same group prepared the mixed metal Ti-substituted **NH**_**2**_**-UiO-66(Zr/Ti)** framework by postsynthetic exchange. This material displayed improved photocatalytic performance for CO_2_RR and HER.^299^ DFT and electron spin resonance studies proposed that the introduced Ti acts as an electron mediator between the ATA light absorbing linker and the SBU-based Zr^IV^ centers. The interested reader realizes that this thermodynamic ordering is opposite to that of bulk metal oxide nanoparticles where the conduction band of ZrO_2_ is considerably higher than that of TiO_2_.^300^

In a related work, Gao et al.^188^ described a Ti/Zr porphyrinic MOF, **Ti/Zr-MOF-525**, which was able to perform gas-phase CO_2_ reduction into CH_4_ and CO under visible light irradiation. No exogenous organic electron donor was added, and the CO_2_RR was accompanied by water oxidation. The **Ti/Zr-MOF-525** was afforded from **Zr-MOF-525** in a metal metathesis procedure that replaced Zr with Ti to afford a material with a Ti/Zr atomic ratio of 9:1. The **Ti/Zr-MOF-525** yielded CH_4_ and CO as CO_2_ reduction produces in yields that were roughly 18 times higher than those of **Zr-MOF-525**. Overall, catalysis proceeded with less than one turnover per SBU.

In 2019, Wei et al.^301^ reported the postsynthetic modification of **UiO-68-NH**_**2**_ by a Schiff-base condensation with 3-fluorobenzaldehyde, 3-methylbenzaldehyde, and 3-methoxybenzaldehyde to afford three novel MOFs, **UiO-68-F**, **UiO-68-CH**_**3**_, and **UiO-68-OCH**_**3**_, respectively (Figure 30b). The MOF with the most electron-rich linker of the series, **UiO-68-OCH**_**3**_, performs best in the light-driven CO_2_-to-CO reduction in the presence of TEOA. After 6 h, the TONs of **UiO-68-F**, **UiO-68-CH**_**3**_, and **UiO-68-OCH**_**3**_ per SBU were determined to be 0.44, 2.88, and 4.08, respectively. Recycling experiments for the different frameworks revealed that the CO production does not level off for three consecutive runs over a total of 18 h.

Li and co-workers^302^ described a set of Fe-MOFs (**MIL-101(Fe)**, **MIL-53(Fe)**, **MIL-88B(Fe)**). The constituting Fe-oxo cluster SBUs could directly be photoexcited, and after reductive quenching produced Fe^II^ species that catalyze the reduction of CO_2_ to formate. Owing to unsaturated Fe sites, **MIL-101(Fe)** showed the highest activity among the examined MOFs. The amine-functionalized Fe-MOFs, **NH**_**2**_**-MIL-101(Fe)**, **NH**_**2**_**-MIL-53(Fe)**, and **NH**_**2**_**-MIL-88B(Fe)**, assembled with H_2_ATA instead of H_2_BDC, displayed improved photocatalytic performance compared to their unfunctionalized controls (Figure 30c). Photocatalytic CO_2_-to-formate conversion experiments over the series of Fe-MOFs and their amino-functionalized derivatives showed that **NH**_**2**_**-MIL-101(Fe)** performed the best. The authors suggest that both ATA excitation and subsequent electron transfer to the SBUs, as well as the direct excitation of the Fe–O clusters contribute to catalytic turnover under irradiation and in the presence of an electron donor.

In contrast to the dual excitation route described above, Chen et al.^303^ described a dual catalytic mechanism in the light-driven reduction of CO_2_ to formate, catalyzed by the Zr-MOF **NNU-28** ([Zr_6_O_4_(OH)_4_(L)_6_]·6DMF, H_2_L = 4,4′-(anthracene-9,10-diylbis(ethyne-2,1-diyl))dibenzoic acid; DMF = dimethylformamide) with an anthracene-based linker (Figure 30d). Photocatalytic experiments and EPR studies revealed that CO_2_ reduction can occur directly at the photosensitizing anthracene-based organic linker or at the Zr_6_ oxo clusters after ligand-to-metal charge transfer. Taking advantage of the same principle, Sun et al.^304^ reported the use of an amino-functionalized photosensitizing MOF **Zr-SDCA-NH**_**2**_ (H_2_SDCA-NH_2_ = 2,2′-diamino-4,4′-stilbenedicarboxylic acid), for CO_2_-to-formate reduction.

Based on the work on **NNU-28**, SK et al.^190^ reported an anthracene-based Zr-MOF, of which the activated form was employed as a photoactive catalyst for the CO_2_RR, accompanied by water oxidation. The Zr-MOF was afforded in a solvothermal synthesis from ZrOCl_2_·8H_2_O and the anthracene-based linker. The latter was postulated to be the photoresponsive unit that is oxidatively quenched by the Zr-cluster SBU that catalyzes the CO_2_RR. As water is proposed as the terminal donor in the process, this implies that the anthracene linker catalyzes water oxidation, a process that is unprecedented in the homogeneous phase. Either way, the system evolved formic acid as the only CO_2_ reduction product with a TOF of less than one per hour.

Logan et al.^305^ reported the synthesis of a series of isoreticular **NHR-MIL-125** (R = methyl, ethyl, isopropyl, *n*-butyl, *n*-heptyl, cyclopentyl, and cyclohexyl) frameworks and connected the experimentally observed reduction of the optical bandgap to an increase in the photocatalytic CO_2_RR activity. **NHCyp-MIL-125** (Cyp = cyclopentyl) displayed a smaller bandgap (*E*_g_ = 2.30 eV), a longer-lived excited-state (τ = 68.8 ns) and a larger apparent quantum yield (Φ_app_ = 1.80%) compared to those of the parent **NH**_**2**_**-MIL-125** (*E*_g_ = 2.56 eV, Φ_app_ = 0.31%, τ = 12.8 ns) and was the most active catalyst for formate production under blue light illumination over 120 h. We note that these MOFs are, however, not expected to be semiconductors (see section 2.3.3), so instead of “bandgap” one should discuss the lowest excited state energy as for a general molecular PS. A general finding of the report was that linkers with secondary amines displayed larger apparent quantum yields and longer excited-state lifetimes compared to their primary amine analogues.

Recently, Chen et al.^306^ presented a combination of dual excitation and dual catalytic function in a mixed linker MOF, **D-TiMOF**. This MOF is based on the widely used light-responsive **NH**_**2**_**-MIL-125(Ti)** (**TiMOF**) and was developed to further increase its photocatalytic activity. For this reason, photosensitizing Zn-porphyrins, ZnTCPP, were installed at the Ti-oxo SBUs as a second light-harvester in addition to the already present ATA linkers. Photocatalytic CO_2_ reduction experiments were performed for TiO_2_, ZnTCPP, **TiMOF**, **D-TiMOF**, and the physical mixture of **TiMOF** and ZnTCPP. **TiMOF** exhibited higher catalytic activity than TiO_2_ which was attributed to the CO_2_ sorption ability of MOFs. The presence of ZnTCPP in **D-TiMOF** led to an 11-fold enhancement in CO_2_-to-CO conversion efficiency as compared to that of the parent **TiMOF**. The significantly enhanced activity is accredited to the synergistic effect between the Ti-oxo clusters and ZnTCPP, not only providing photoelectrons for the Ti-sites but also offering a second reactive site.

In contrast to the benzene-based linkers, the following frameworks consist of either larger π-systems or porphyrins in combination with Zr-oxo clusters. Considering the benefits of incorporating large π-conjugated units into MOFs, Qin et al.^307^ reported the synthesis of the Zr-based MOF **PCN-136**, Zr_6_(μ_3_-O)_4_(μ_3_-OH)_4_(OH)_6_(H_2_O)_6_(HCHC) (HCHC = hexakis(4-carboxyphenyl)hexabenzocoronene). Owing to the low solubility and the unfavorable conformation of the HCHC ligand, **PCN-136** could only be obtained from aromatization-driven postsynthetic annulation of the hexaphenylbenzene unit from the parent **pbz-MOF-1** (pbz = polybenzene) scaffold. In comparison with **pbz-MOF-1**, the combination of isolated photosensitizing hexabenzocoronene moieties and catalytically active Zr-oxo nodes in **PCN-136** led to an enhanced photocatalytic activity for the reduction of CO_2_ to formate.

Based on porphyrinic TCPP linkers, Qiu et al.^308^ reported the synthesis of two mixed-linker MOFs, [(CH_3_)_2_NH_2_]_4_[Zn_4_O]_4_[ZnTCPP]_5_[BTB]_8/3_ (**PCN-137**) and [Zr_6_(μ_3_-O)_4_(μ_3_-OH)_4_][TCPP][TBTB]_8/3_ (**PCN-138**) (BTB = 1,3,5-benzene(tris)benzoate and TBTB = 4,4′,4″-(2,4,6-trimethylbenzene-1,3,5-triyl)tribenzoate). Employed as photocatalysts for the CO_2_RR in the presence of triisopropanolamine as an electron donor, **PCN-138** displayed high selectivity for formate production over 12 h in an aqueous medium. The authors postulate that this performance was due to simultaneous immobilization of porphyrinic PS units and active Zr-oxo nodes within the MOF platform, resulting in a material that features a broad absorption in the visible region and a high CO_2_ sorption performance.

Xu et al.^151^ demonstrated the selective conversion of CO_2_ to formate with TEOA as an electron donor and a **PCN-222** framework as a catalyst, consisting of H_2_TCPP light-harvesting porphyrinic linkers and Zr_6_ clusters as SBUs. Mechanistic investigations revealed that the porphyrinic linkers act as PSs that upon illumination transfer electrons to the Zr-oxo clusters, where CO_2_ is reduced to formate. Under similar conditions, H_2_TCPP was also investigated as a homogeneous photocatalyst for the CO_2_RR, but produced noticeable smaller amounts of product compared to the MOF.

Aziz et al.^309^ described the change in electronic structure in a series of Fe-doped ZnTCPP-based MOFs (**Al-PMOFs**) employed for the reduction of CO_2_ to CH_3_OH and CH_4_, by incorporating Fe at the SBUs as well as into the TCPP linkers. While the incorporation of Fe at the porphyrinic sites led to an increase of the highest occupied molecular orbital (HOMO) level, the Fe-doping at the metal nodes lowered the conduction band significantly. Hence, the most promising results were achieved by partially introducing Fe into the Al SBUs (1:1), while keeping the Zn porphyrinic centers unchanged, leading to a nearly ideal bandgap of 1.9 eV and band edge positions suitable for water splitting and CO_2_ reduction at pH 7. We again note that these MOFs are unlikely to be semiconductors (per section 2.3.3), so one should discuss the lowest excited state energy rather than the “bandgap”.

Zhang and co-workers^310^ reported a series of Zr-based MOFs with large π-conjugated organic linkers for a near-infrared light-driven CO_2_RR. The photoinduced electron transfer pathway from the excited π-conjugated linkers to the Zr cluster SBU was investigated by ultrafast spectroscopic studies, XPS, and in situ EPR experiments. In the presence of TEOA, the tetrakis(4-carboxybiphenyl)naphthoporphyrin)-based MOF (**TNP-MOF**) with the largest π-conjugated linker displayed the highest CO_2_ reduction rate under NIR light irradiation (λ ≥ 730 nm), as well as the highest apparent quantum efficiencies of up to 2.03 and 1.11% at 760 and 808 nm, respectively.

##### 3.1.4.2 Cobalt

**Figure 31:**
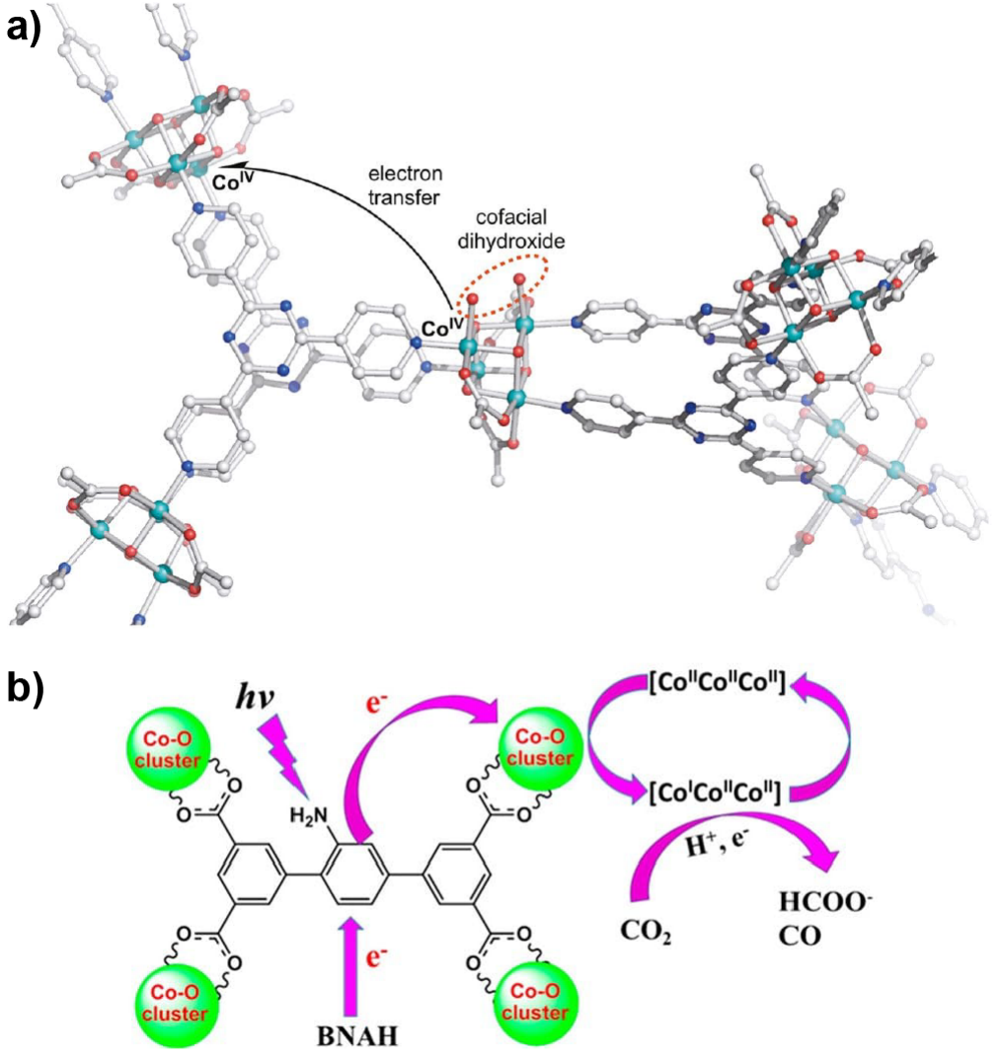
a) Proposed redox-hopping mechanism to achieve a [Co^III^_2_Co^IV^_2_] or [Co^III^_3_Co^V^] intermediate by redox disproportionation of two [Co^III^_3_Co^IV^] clusters in **Co**_**4**_**-TPT**. Reprinted with permission from reference.^311^ Copyright 2019 National Academy of Science. b) Suggested mechanism for the photocatalytic reduction of CO_2_ by **Co-MOF**. Reprinted with permission from reference.^318^ Copyright 2018 American Chemical Society.

The Co_4_O_4_(OAc)_4_(py)_4_ cubane is a competent WOC partly due to labile carboxylate ligands, though it is claimed that this comes at the cost of long-term stability. As a homogeneous compound, loss of the carboxylate ligands is proposed to result in cluster aggregation and deactivation.^311^ Tilley and co-workers^311^ constructed a MOF containing isolated cubane sites (Figure 31a) and demonstrated that this material remained stable up to pH 14. As noted by Wang and Meyer,^312^ this advance did result in notable stability improvement, though a significant proportion of the cubane units remained inaccessible due to the insulating nature of the parent MOF. The comparison between the **cubane@MOF** material and photosystem II was specifically drawn, noting that while photosystem II has an elegant electron transport chain in place, **cubane@MOF** lacked such a system.^312^ This points toward the possible future rational design of biomimetic MOFs wherein both catalytic sites and electron transport chains are incorporated.^209^

The Co centers in **ZIF-67**, a MOF in which Co-centers are coordinated by four imidazolate linkers, have been utilized for light-driven HER. In order to do so, Ru^2+^-based PSs, RuN_3_, were coordinated to **ZIF-67** crystals to afford a **RuN**_**3**_**/ZIF-67** hybrid system. Following photon absorption, the excited RuN_3_ dye engages in energy transfer with the **ZIF-67**, which upon addition of water and a sacrificial electron donor engages in the HER. Catalysis ceases after 10 h due to RuN_3_ decomposition and can be restored by addition of a fresh PS.^313^

Qin et al.^314^ presented the coprecipitation of Co(NO_3_)_2_·6H_2_O and 2-methylimidazole to assemble nanosized **ZIF-67** crystals. The latter function as a catalyst for the light-driven HER and CO_2_-to-CO reduction in cooperation with Ru(bpy)_3_Cl_2_ as a PS and TEOA as an electron donor. By varying the excitation wavelength, it was shown that the combined yields of the reaction products correlated with the absorption spectrum of the PS, showing that excitation of the PS is the initial step of the photocatalytic cycle.

The catalytic activity of Co^II^ was utilized in a cobalt-based MOF, **Co–MB**, prepared from Co(NO_3_)_2_, 3,3′-methylenediphthalic acid (H_4_mda) and 1,4-bis(imidazol-1-ylmethyl)-benzene (bib) in an aqueous sodium hydroxide solution under hydrothermal conditions.^315^ In **Co–MB**, two crystallographically independent Co^II^ centers exhibit distorted tetrahedral coordination geometries with two carboxylate oxygen atoms from two different mda^4–^ anions and two nitrogen atoms from two different bib ligands creating the primary coordination sphere. **Co–MB** catalyzes the light-driven HER in conjunction with various fluorescein-based PSs and TEA as electron donor. The material could be recycled three times without compromising its activity as catalyst for the light-driven HER, illustrating the power of the MOF matrix to stabilize molecularly defined active sites.

In 2014, Wang et al.^316^ described a robust cobalt imidazolate framework **Co-ZIF-9** that serves as a CO_2_ reduction catalyst in conjunction with a Ru-based PS and TEOA as an electron donor. Even though a homogeneous mixture of the ligand and Co^2+^ was proven to be able to catalyze CO and H_2_ evolution, the combined CO and H_2_ production performances as well as the TON were superior for the MOF system. Furthermore, stability experiments with recovered **Co-ZIF-9**, redispersed in fresh dye solution, showed no noticeable alteration in the activity of the reused catalyst over 2.5 h. Similarly, Zhao et al.^317^ presented a pillared layer **Co**_**6**_**-MOF** that catalyzed the CO_2_-to-CO reduction, when combined with a Ru-based PS. Compared to the homogeneous control, the MOF system exhibited higher stability, giving rise to higher TONs.

In 2018, Liao et al.^318^ reported the solvothermal one-pot reaction of Co(NO_3_)_2_·6H_2_O and H_4_L (2′-amino-[1,1′:4′,1″-terphenyl]-3,3″,5,5″-tetracarboxylic acid), to form **Co-MOF** ([Co_3_(HL)_2_·4DMF·4H_2_O]) for both photocatalytic hydrogen evolution and CO_2_ reduction. While the HER was driven by a Ru-based PS under illumination, the internal light absorbing organic linker in combination with the catalytically active Co–O clusters was sufficient to drive the photocatalytic CO_2_ reduction process. Under visible light irradiation and in the presence of BNAH (1-benzyl-1,4-dihydronicotinamide) as a sacrificial electron donor, excited HL^3–^ transfers electrons to the SBUs, where the trinuclear [Co^II^Co^II^Co^II^] cluster is reduced to a [Co^I^Co^II^Co^II^] oxidation state that reduced CO_2_ to CO or formate (Figure 31b).

##### 3.1.4.3 Copper

An unusual active site has been reported in a mixed linker-mixed metal **Na/Cu-MOF**.^319^ The dinuclear Cu_2_ site is asymmetric, with one of the Cu^II^ ions displaying a five-coordinate square-pyramidal geometry while the other adopts a six-coordinate octahedral geometry. In close proximity is an octahedrally coordinated Na^I^ cation. In comparison to a number of reference systems, including a system that lacks the Na^I^ cation, it is shown that **Na/Cu-MOF** is a superior catalyst for the light-driven HER in the presence of TEA and fluorescein as a PS.

Missing linker defect engineering has been used to turn a catalytically incompetent metal-triazolate (**MET**) MOF into a defective, but catalytically active material.^320^ In a defect-free state, each copper center is coordinated by six nitrogen atoms from different 1H-1,2,3-triazole linkers in an octahedral geometry. The coordination sphere of the metal centers is permanently saturated, and as a result, the **MET-Cu** does not catalyze the HER. However, by decreasing the amount of triazole linkers relative to the Cu salt, as well as variation of other reaction parameters, defective **MET-Cu** (**MET-Cu-D**) in which some of the Cu centers lack permanent ligands was prepared. Using Eosin Y as a PS and TEA as a sacrificial electron donor, **MET-Cu-D** was found to be a competent catalyst for the light-driven HER. Postsynthetic “repair” of **MET-Cu-D** by addition of a triazole linker decreased the HER activity, providing indirect proof that the partially under-coordinated Cu centers are indeed responsible for the observed HER activity.

In 2012, Mao et al.^321^ demonstrated the first application of a MOF as an ORR electrocatalyst. The work was based on **Cu-BTC** (BTC = 1,3,5-benzenetricarboxylate) that, however, revealed its structural instability under ORR conditions in aqueous media. Therefore, the water-stable **Cu-bpy-BTC** was synthesized and used as an electrocatalyst in form of MOF-modified GCEs. In a phosphate buffer at pH 6.0, cyclic voltammograms of **Cu-bpy-BTC** showed well-defined reduction peaks and a current enhancement at −0.20 V in the presence of O_2_, consistent with catalytic turnover. Rotating ring-disk electrode voltammetry of the Cu^II^-based MOF showed an almost 4e^–^ reduction pathway, consistent with reduction of oxygen to water without generation of H_2_O_2_ as side product.

In 2012, the electrochemical synthesis of the bulk MOF **Cu**_**3**_**(BTC)**_**2**_, also known as **HKUST-1**, was described, followed by its application for the electrocatalytic reduction of CO_2_ to oxalic acid as thin films on GCEs.^322^ Within the framework, Cu^I^, which is formed at a potential of −0.62 V vs Ag/Ag^+^ is the catalyst for the CO_2_RR. The authors demonstrated that the synthesized **Cu**_**3**_**(BTC)**_**2**_ framework is able to form the oxalate anion upon 2-electron reduction and dimerization of CO_2_.

In 2019, Zhang et al.^323^ described the zeolite-related boron imidazolate framework **BIF-29**, which features mononuclear copper in a square-planar coordination mode. In this geometry, the Cu sites are highly active and are able to catalyze the light-driven reduction of CO_2_ to CO in combination with a Ru-based PS and TEOA as a sacrificial electron donor. Theoretical studies revealed the contribution of the Cu sites with weakly bound water molecules (Cu–O: 2.538 Å) for the absorption and activation of CO_2_ and the further stabilization of the *COOH intermediate. Furthermore, steady-state and time-resolved fluorescence experiments indicated that the Cu sites engaged in charge separation. Solar-driven catalytic CO_2_ reduction employing **BIF-29** led to CO evolution with a selectivity of 82.6%.

In 2021, Liu et al.^324^ reported a series of tricycloquinazoline-based 2D MOFs as electrocatalysts for the reduction of CO_2_ toward methanol. The electron-deficient but nitrogen-rich catechol linker HHTQ (2,3,7,8,12,13-hexahydroxytricycloquinazoline) was employed to coordinate to transition metals (Cu^2+^ or Ni^2+^) to afford 2D graphene-like sheets of **M**_**3**_**(HHTQ)**_**2**_ (M = Cu or Ni). **Cu**_**3**_**(HHTQ)**_**2**_ as an electrocatalyst for the CO_2_RR displayed high selectivity toward CH_3_OH with a FE of up to 53.6% at −0.4 V vs RHE, exhibiting larger CO_2_ adsorption energies and higher activities than both, the isostructural **Ni**_**3**_**(HHTQ)**_**2**_ and the archetypical **Cu**_**3**_**(HHTP)**_**2**_ (HHTP = 2,3,6,7,10,11-hexahydroxytriphenylene).

##### 3.1.4.4 Nickel

Mani et al.^325^ reported **Ni-BTB-BPE** ([Ni_3_(BPE)_4_(BTB)_2_(H_2_O)_2_]·2DMF·2H_2_O (BTB = 1,3,5-tris(4-carboxyphenyl)benzene and BPE = 1,2-bis(4-pyridyl) ethane), in which the BTB linkers interconnect the Ni ions to form 2D Kagome-type layers, which are then connected through the BPE linker to form the 3D construct. **Ni-BTB-BPE** showed both ORR and OER activity but only at the surface Ni sites due to the nonporous structure of **Ni-BTB-BPE**. The catalytic activity of **Ni-BTB-BPE** for oxygen reduction and evolution was investigated when adsorbed together with Ketjenblack on a conducting substrate. **Ni-BTB-BPE/C** showed a high selectivity for the four-electron pathway with OH^–^ as the major product.

The 2D layered MOF **[Ni**_**2**_**(PymS)**_**4**_**]**_**n**_ (PymSH = pyrimidine-2-thiol) with thiolate-bridged binuclear Ni^II^ nodes was reported to catalyze the light-driven HER in the presence of a variety of PSs and sacrificial donors, with fluorescein and TEA being the optimal combination.^326^ The MOF was formed by the reaction of Ni(OAc)_2_ and 2-mercapto-pyrimidine in the presence of KOH and was a superior catalyst compared to only the Ni^II^ salt of the ligand. The **[Ni**_**2**_**(PymS)**_**4**_**]**_**n**_ catalyst possessed a 2D lamellar structure built from binuclear nodes [Ni_2_(PymS)_4_], in which the Ni^II^ ions are in turn connected by PymS^–^ ligands through S and N atoms.

Another interesting SBU active site and its activity for the HER was recently reported by Budnikova and co-workers.^327^ Using 4,4′-bipyridine as cross-linking units, Ni and Co redox-active coordination polymers with ferrocene-containing diphosphinate ligands were tethered together to form 3D MOFs. The nickel or cobalt atoms were coordinated to four phosphinic oxygen atoms in the equatorial plane and two nitrogen atoms from the 4,4′-bpy ligand in the axial positions. It is shown that the 4,4′-bpy linkers reduced the overvoltage for HER, resulting in lower Tafel slopes, consistent with more favorable HER kinetics.

Ultrathin 2D nanosheets, produced by exfoliation of a MOF and consisting of bis(4′-carboxy-2,2′:6′,2″-terpyridine) ruthenium linkers and 3d transition-metal (Mn, Co, Ni, and Zn) SBUs, were reported as good photocatalytic HER materials.^328^ Combined experimental and theoretical insights revealed a reductive quenched pathway of the *Ru^2+^ excited state by the ascorbate electron donor, followed by electron transfer to the transition-metal nodes. The nodes at the edge of the nanosheets were proposed as the active sites. DFT calculations attributed the different catalytic activities of the nanosheets to different hydrogen atom adsorption free energies at the varying transition-metal nodes. Of the systems investigated, the Ni–Ru nanosheets exhibited the highest HER rates.

In 2020, Fang et al.^150^ described the synthesis of the framework **PCN-601**, combining pyrazolyl porphyrinic light harvesters and Ni-based SBU active sites. **PCN-601** with a Ni center in the porphyrin linker catalyzed the gas phase reduction of CO_2_-to-CH_4_. The reduction was accompanied by the oxidation of H_2_O vapor to H_2_O_2_.

Very recently, Iqbal et al.^329^ explored the synergistic effect of the incorporation of Ni and Fe in a 2D MOF for the electrochemical CO_2_RR. Surrounded by four nitrogen atoms of dipyrazino quinoxaline-2,3,6,7,10,11-hexamine, the metal centers are located in a porphyrin-like environment. DFT calculations showed that the simultaneous incorporation of Ni and Fe leads to a system with a high FE of 98.2% for CO evolution and a stability of up to 30 h under an applied potential of −0.5 V vs RHE.

##### 3.1.4.5 Other Metals: Ruthenium, Cerium, Europium, Bismuth, Aluminum, and Zinc

**Figure 32:**
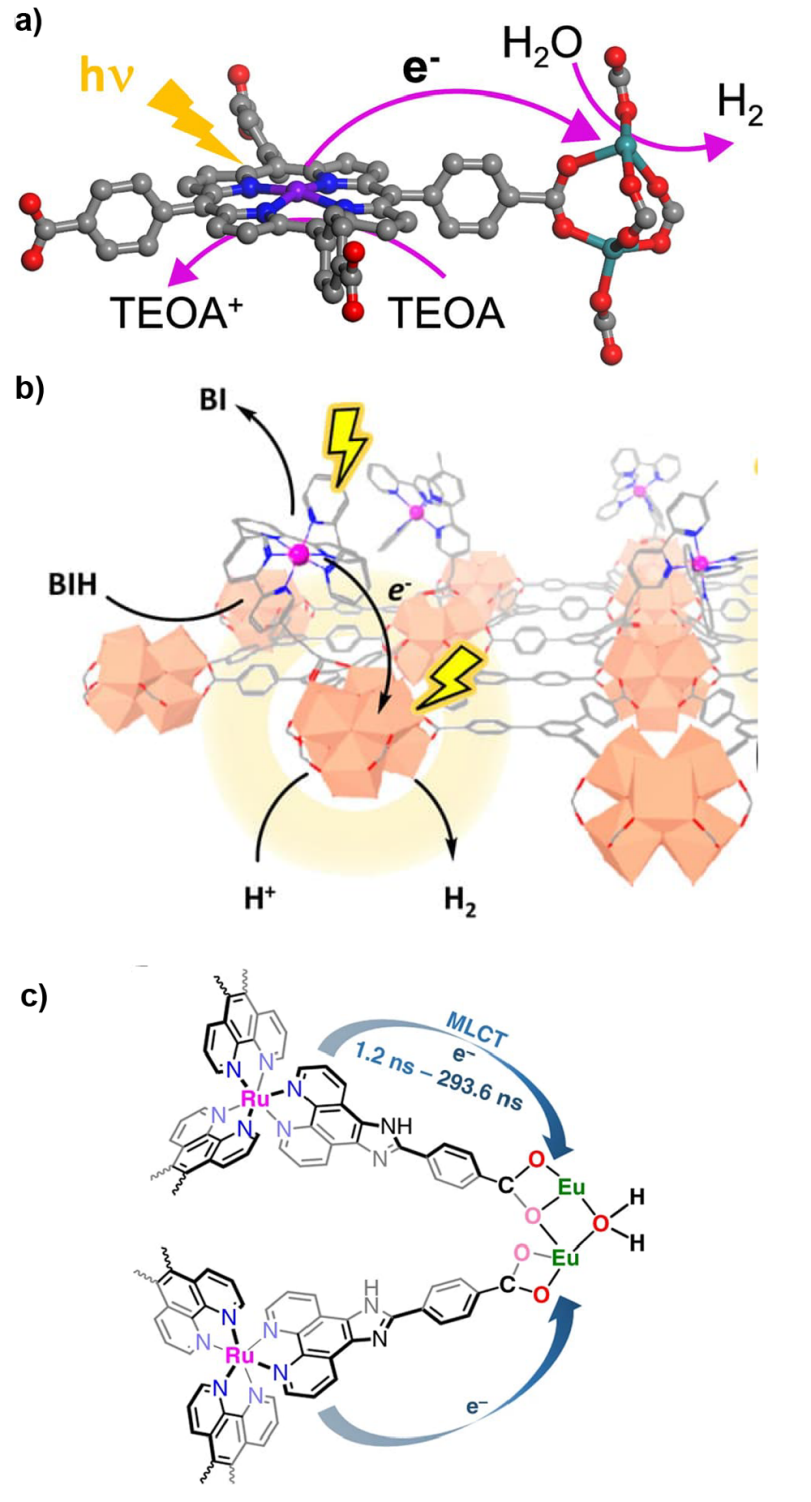
a) An example of a molecular catalyst stabilized as secondary building unit in form of a Ru_2_ paddle wheel in **Ru-TBP-Zn**.^331^ b) A molecular catalyst stabilized as secondary building unit, demonstrated with a hexanuclear Ce_6_ cluster in **Ce**_**6**_**-BTB**.^332^ c) Suggested mechanism for the photocatalytic reduction of CO_2_ by **Eu-Ru(phen)**_**3**_**-MOF**.^333^ Adapted with permission from the respective references. Copyright 2018 American Chemical Society, 2020 American Chemical Society, and 2018 Springer Nature, respectively.

A series of diruthenium-based MOFs **[Ru**_**2**_**(BDC)**_**2**_**X]**_**n**_ (X = Cl, Br, and BF_4_) have been shown to catalyze the light-driven HER in the presence of [Ru(bpy)_3_]^2+^, methyl viologen, and EDTA for longer than 4 h.^330^ While in the early report, the PS was in the solution phase, subsequent work has focused on immobilizing light absorbing PSs as linker units in conjunction with catalysts that are stabilized or supported as SBUs. Ru_2_ paddle wheel SBUs (Figure 32a) have been combined with TCPP linkers to afford two novel MOFs, **Ru-TBP** and **Ru-TBP-Zn**. In these systems, it is shown that the excited porphyrin linkers are oxidatively quenched by the Ru_2_ SBUs as the initial step of the HER process.^331^

Moving away from scarce metal catalysts, a hexanuclear cerium cluster as catalytic site could be stabilized in a **Ce**_**6**_**–BTB** MOL consisting of the Ce_6_ clusters as SBUs and BTB linkers (Figure 32b). When postsynthetically modified with molecular Ir- or Ru-based PSs, both MOLs engaged in the photocatalytic HER under visible light irradiation.^332^

In 2018, Yan et al.^333^ described a MOF with large pore dimensions, consisting of dinuclear Eu_2_(μ_2_-H_2_O) SBUs with photosensitizing Ru(phen)_3_-derived metalloligands (phen = phenanthroline). Time-resolved photoluminescence studies in combination with femtosecond transient optical absorption spectroscopy were consistent with a charge transfer from excited Ru-PS to the Eu–O nodes on a time scale of 1–300 ns. The reduced [Eu^II^–H_2_O–Eu^II^] oxidation state of the SBU is catalytically competent for the selective reduction of CO_2_ to formate (Figure 32c). The formate production rate of for **Eu-Ru(phen)**_**3**_**-MOF** was notably higher than the reported values of **NH**_**2**_**-MIL-125(Ti)**,^296^**NH**_**2**_**-UiO-66(Zr)**,^298^ and **PCN-222**(151) under similar reaction conditions.

In 2020, Li et al.^334^ reported a 2D bismuth framework (**Bi-MOF**), consisting of Bi–O rods connected with tritopic BTC linkers. This MOF showed permanently accessible pore channels for the efficient electrochemical CO_2_RR to HCOOH. **Bi-MOF** displayed a remarkable FE for formic acid formation over a broad potential window, reaching its maximum of 92.2% at −0.9 V vs RHE with a durability of over 30 h. The partial current density for HCOOH around 41.0 mA mg_Bi_^–1^ was 4 times higher compared to that of commercial Bi_2_O_3_ and Bi sheets. Possible explanations are the finding that the Bi^3+^ state was able to be preserved within the **Bi-MOF** system and that the channels with accessible Bi active sites favor the formation of *HCOO and suppress the unwanted side-reaction to hydrogen, as suggested by theoretical calculations.

Toma and co-workers^335^ presented the power of confinement of metal centers based on investigations on the aluminum containing MOF, **MIL-53(Al)**. Aluminum as a foil is not active for the CO_2_RR but instead shows high selectivity for the HER. Confined in **MIL-53(Al)**, aluminum restrains the HER and enhances the carbon dioxide reduction up to a total FE of 40% for CO and formic acid at a potential of −1.1 V vs RHE.

Bie et al.^336^ reported the synthesis of **Spiro-Zn-MOF**, a 3D framework consisting of the organic PS SATC (5,5′-(10*H*,10′*H*-9,9′-spirobi[acridine]-10,10′-diyl)diisophthalic acid) and Zn^2+^-based SBUs. The linker is a donor/acceptor structure that displayed an excited state lifetime up to a microsecond, which was preserved also in the **Spiro-Zn-MOF**. The long-lived excited state and charge transfer to the Zn nodes allowed for the photocatalytic CO_2_RR toward CO. Proof for the electron transfer to the Zn^2+^-based SBU was obtained by EPR analysis of illuminated samples of **Spiro-Zn-MOF**, which showed a signal that was attributed to Zn^+^. The authors pointed out that no organic donor was required for catalysis, implying that electrons are provided from water oxidation, which is however not commented on further. Control experiments with pure SATC, zinc nitrate, and a homogeneous mixture of SATC and zinc nitrate did not afford any CO.

##### 3.1.4.6 Bimetallic SBUs

**Figure 33:**
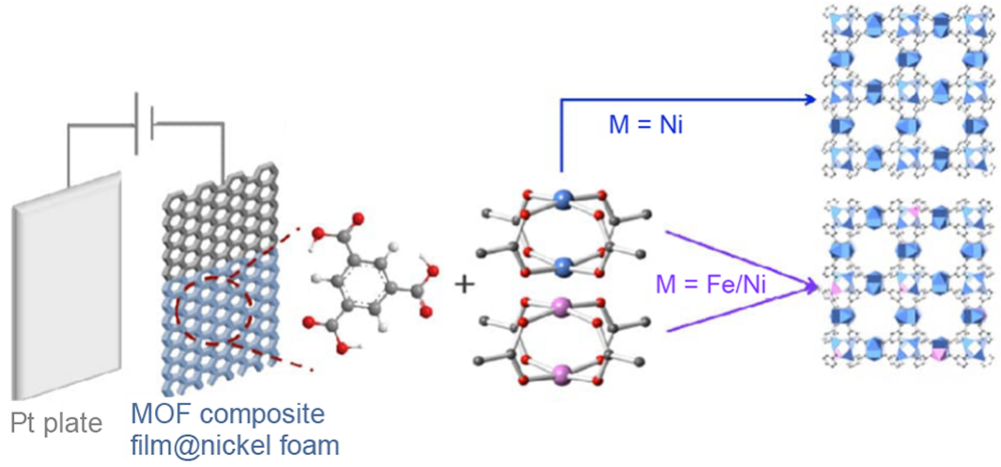
Electrochemical synthesis of **Ni-BTC** or mixed metal **Fe/Ni-BTC** MOFs on nickel foam. Adapted with permission from reference.^337^ Copyright 2016 American Chemical Society.

The electrochemical potential that needs to be applied at a catalytic metal site to engage in catalysis can be modulated by including a second metal center, often resulting in a structurally more complex SBU. Examples of such MOFs with tuned redox potentials have been reported. For example, MOFs which have been doped with a second metal cation have been shown to have redox potentials more suitable for fuel-forming reactions. In 2016, Wang and co-workers^337^ used cathodic electrochemical deposition on nickel foam to prepare a series of such bimetallic MOFs based on the BTC linker with either Ni, Fe, or both Ni and Fe as metals in the SBU (Figure 33). The mixed metal **Fe/Ni-BTC** MOFs, where the Fe:Ni ratio was 1:12, yielded the lowest overpotential for electrochemical OER catalysis. Based on literature precedent, the authors suggested that the nickel sites in the SBU were the active catalytic sites for the OER. Intriguingly, the Fe was suggested to play a dual role in improving catalysis, by increasing framework conductivity and by shifting the Ni^2+/3+^ redox potential to milder values. Since then, similar data has been published supporting this cooperative tuning of the electronics of the active metal site by incorporation of a second metal.^338−341^

Yao et al.^342^ recently reported the synthesis of **N-NiFe-MOF** that was obtained through a postsynthetic nitridation process from **NiFe-MOF**, a BDC-based MOF that was obtained from a NiFe layered double hydroxide. During the nitridation, NH_3_ coordinates to the open metal sites in **NiFe-MOF** to form **N-NiFe-MOF**, which is a precatalyst for the OER. Based on DFT calculations and experimental results, the authors suggested that the amino ligands at the open metal sites are oxidized to nitric oxides during the OER, thereby affording **NiFe(NO)-MOF**, which is a potent OER catalyst in a 1.0 M KOH solution. The transformation from **N-NiFe-MOF** to **NiFe(NO)-MOF** introduces an electron deficiency on the Fe atoms and promotes the OER through the stabilization of the intermediate *O species, thereby decreasing the required overpotential for the conversion to *OOH.

Yue et al.^343^ reported the synthesis of 2D nanosheets of the trimetallic framework **NiCoFe–NDA** (NDA = 2,6-naphthalenedicarboxylic acid) on nickel foam (**NiCoFe–NDA/NF**) as a highly active electrocatalyst for the OER. The **NiCoFe–NDA/NF** electrode displayed a much higher OER activity and stability when compared to the monometallic versions. Thereby, the **NiCoFe–NDA** suffered topological transformation and underwent an in situ phase transformation, inducing surface-rich metal oxyhydroxides with low-coordination environments, providing a multimetal coupling effect which is beneficial for the water oxidation.

Yoon et al.^344^ synthesized bimetallic conductive 2D MOFs, **Co**_*x*_**Ni**_*y*_**-CAT**, consisting of HHTP linkers and varied ratios of Co^2+^ to Ni^2+^ metal ions, and employed them as catalysts in the ORR. The authors found that compared to monometallic Co^2+^ or Ni^2+^ MOFs (**Co-CAT** or **Ni-CAT**), the bimetallic MOFs displayed higher ORR activity, possibly due to the combination of abundant Co–O moieties serving as catalytically active sites and the high conductivity within **Ni-CAT**. The optimized **Co**_**0.27**_**Ni**_**0.73**_**-CAT** material had a higher electron transfer number of 3.95 (lower H_2_O_2_ production) and better durability (87% after 10 000 s) than its monometallic congeners.

Shinde et al.^345^ reported the synthesis of hexaiminobenzene (HIB)-based MOFs (**Mn/Fe-HIB-MOF**) as bifunctional ORR and OER electrocatalysts. The **Mn/Fe-HIB-MOF** was afforded from HAB in ammoniacal solutions of Mn^II^ and Fe^II^ salts, followed by a thermal treatment. **Mn/Fe-HIB-MOF** exhibited precisely controlled M^2+^ species in quintet-shelled hollow spheres, fast electron and mass transfer pathways, and catalytically effective M^II^–N_4_ moieties. Being a bidirectional electrocatalyst, **Mn/Fe-HIB-MOF** was shown to function well as electrode material in rechargeable liquid zinc air batteries. Zinc air batteries made with **Mn/Fe-HIB-MOF** displayed a remarkable cycling stability for 600 h (3600 cycles@25 mA cm^−2^) and excellent mechanical robustness.

##### 3.1.4.7 POM

Huang et al.^346^ presented two structurally analogous POM-based metalloporphyrin coordination frameworks (POMCFs), **NNU-13** ([PMoV_8_Mo^VI^_4_O_35_(OH)_5_Zn_4_]_2_[ZnTCPP][2H_2_O]·*x*Guest) and **NNU-14** ([PMoV_8_Mo^VI^_4_O_35_(OH)_5_Zn_4_]_2_[ZnTCPP][2H_2_O]·*y*Guest). The systems were composed of catalytically active Zn-ε-Keggin nodes and photosensitizing ZnTCPP linkers, and catalyzed CO_2_-to-CH_4_ conversion in >96% selectivity in an aqueous solution in the presence of TEOA as a sacrificial agent and in the absence of any additional PS. CO that was formed during the reaction was shown to be a reaction intermediate that was further reduced to CH_4_. Owing to the slight structural deformation, **NNU-14** displayed slightly lower CH_4_ selectivity of 96.2% compared to the 96.6% for **NNU-13**. Under illumination, the electrons are easily transferred from photoexcited ZnTCPP units to the POM clusters, where coordinated CO_2_ gets reduced.

In 2019, Li et al.^347^ revealed the novel POM-based framework TBA_5_[P_2_Mo_16_^V^Mo_8_^VI^O_71_(OH)_9_Zn_8_(L)_4_] (**NNU-29**; L^2–^ = 4,4′-((((perfluoropropane-2,2-diyl)bis(4,1-phenylene))bis(oxy))bis(methylene))dibenzoate and TBA^+^ = tetrabutylammonium). This system displayed catalytically active molybdenum POM clusters that , in combination with [Ru(bpy)_3_]^2+^ as a PS and TEOA as an electron donor, engaged in CO_2_-to-formate reduction in an aqueous solution with a selectivity of 97.9% after 16 h. The authors suggested that the competing HER is suppressed by the hydrophobic linkers that shield the catalytically active sites from water. Control experiments with a physical mixture of Zn_4_-ε-Keggin and H_2_L revealed a lower selectivity of 78.5% and yield for HCCOH production, demonstrating the benefits of heterogenizing CO_2_RR catalysts in MOFs.

##### 3.1.4.8 Transition Metal–Heteroatom SBUs

**Figure 34:**
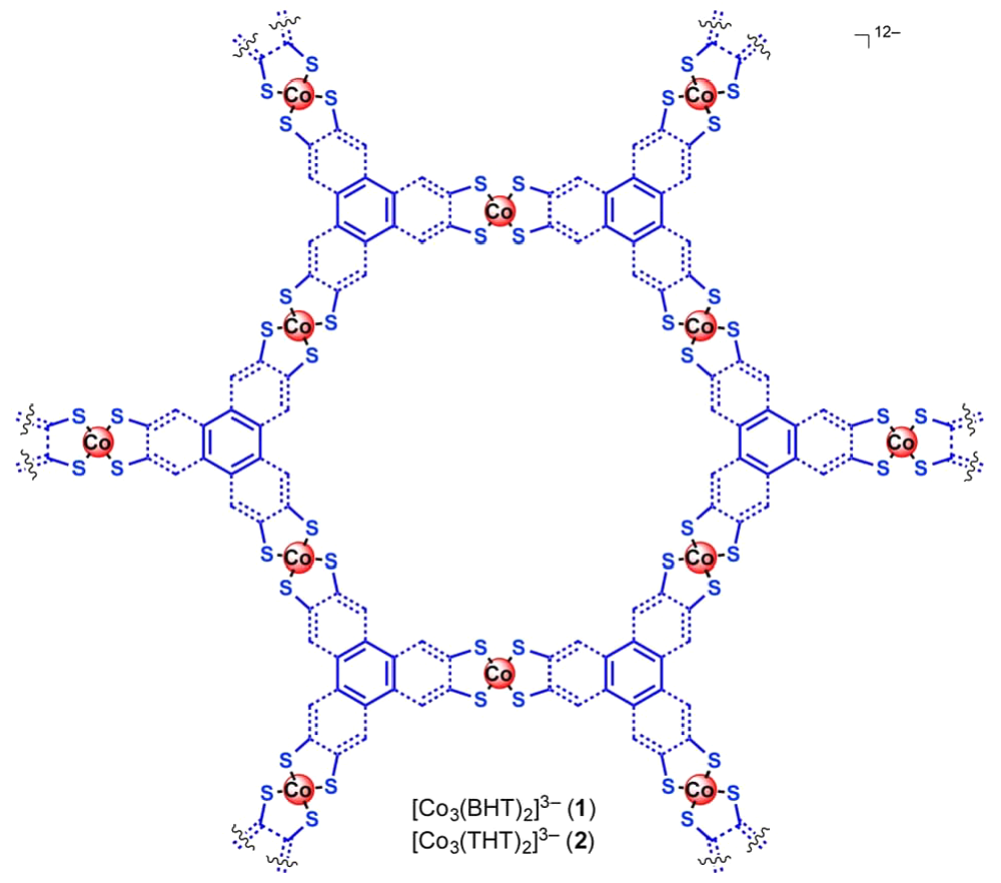
Synthesis of the cobalt dithiolene surfaces, through a liquid–liquid interfacial process. Adapted with permission from reference (348). Copyright 2015 American Chemical Society.

Cobalt dithiolene HER catalysts can be structurally stabilized by incorporation into 2D metal–organic surfaces.^348^ For this purpose, cobalt dithiolene catalytic sites were assembled in extended architectures, using a tritopic benzenehexathiol linker through a liquid–liquid interfacial process (Figure 34). In this process, an acetonitrile/ethyl acetate solution of [Co(MeCN)_6_][BF_4_]_2_ was layered on top of an aqueous solution of sodium benzenehexathiolate (C_6_S_6_Na_6_). It was demonstrated that integration of cobalt dithiolene catalysts into a metal–organic surface generated highly active electrocatalytic cathode materials for the HER from water. These surfaces display high catalyst loadings and remarkable stability even in acidic aqueous solutions.

In 2017, Liu et al.^349^ designed well-defined M–O_6_ coordination sites within two 2D porous metal-catecholates (**M–CATs**, M = Ni or Co), consisting of HHTP interconnected with either of the bivalent metal ions, Ni^II^ or Co^II^. The catalytic activity was found to come exclusively from the M–O_6_ sites and the ORR was predominantly displayed as a four-electron pathway.

A related Ni and Co-based 2D MOF with mixed THT (2,3,6,7,10,11-triphenylenehexa-thiol) and THA (2,3,6,7,10,11-triphenylenehexamine) linkers has also been reported.^350^ The mixed linker **THTA-M** 2D MOF (M = Ni or Co) with a metal dithiolene-diamine MS_2_N_2_ SBU was evaluated for the electrocatalytic HER, and compared to the activity of the **THT-M** and **THA-M** analogues with metal bis(dithiolene) (MS_4_), and metal bis(diamine) (MN_4_) sites, respectively. It was found that the electrocatalytic HER activity followed the order of MS_2_N_2_ > MN_4_ > MS_4_, and that protonation occurred preferentially on the metal atoms. Heterolytic H_2_ elimination was favored on the M–N units in the MS_2_N_2_ active sites.

Recently, Zhou et al.^351^ systematically investigated a series of 30 **MN**_*x*_**O**_**4–*x***_**–HTP** (M = Fe, Co, Ni, Ru, Rh, and Pd; *x* = 0–4; HTP = hexatriphenylene) 2D conductive MOFs with the help of DFT calculations as catalysts for the electrocatalytic HER, OER, and ORR. The active centers of these materials are the transition-metal–heteroatom linkages. The strong interaction between the metal and the N_*x*_O_4–*x*_ moiety guaranteed the stability of the MOF structure. Tuning the transition metal and the local coordination number of N/O in the catalysts modulated the catalytic activity. Among the investigated materials, **RhN**_**3**_**O**_**1**_**–HTP** was found to be an efficient bifunctional catalyst for both the HER and OER, with an OER overpotential of 0.33 V. Besides, **RhN**_**1**_**O**_**3**_**–HTP** is predicted to be highly promising for the OER and ORR with calculated overpotentials of 0.28 and 0.27 V, respectively.

### 3.2 Facilitated Transport

Substrate and charge transport within porous materials such as MOFs are important phenomena that can easily limit overall catalytic efficacy. Consequently, very good reviews on the topic have recently appeared.^60,61^ In this section, articles are reviewed that specifically address transport in context of catalysis. As outlined in section 2, hopping electron/hole transport that is accompanied by diffusion–migration of charge-balancing counterions, mass transport of reactants/products, or in case of photochemical schemes, the diffusion of sacrificial electron donors/acceptors may all limit catalysis. In some instances, structural modifications to the MOF may not selectively alter only one of these potential bottlenecks, and improved catalytic performance, as for example manifested in increased current densities in electrochemical experiments, may in fact be due to several factors.

In general, two approaches have been taken in the literature to overcome transport limitations. In the first set of examples (section 3.2.1), transport limitations are kept to a minimum at a system level by working with ultrathin films or few-nanometer-sized crystallites.^81,352^ In section 3.2.2, parent MOFs are structurally altered to facilitate one or more of the above-mentioned transport phenomena. From a biomimetic perspective, this strategy bears resemblance to enzymes in which substrate transport is often actively controlled and aided by specific amino acids that form channels to/from the active site.

#### 3.2.1 Minimizing Transport Limitations by Size Reduction

A number of papers have systematically addressed the interplay between catalytic efficacy and the size of MOF particles in case of solution experiments, or thicknesses in case of MOF films on electrodes. It should be pointed out that the examples herein do not have a biomimetic aspect, but address transport limitations at a system level.

Morris and co-workers^255^ reported a study on **Ru-UiO-67**, that is, **UiO-67** with Ru-based WOCs metallo-linkers. Variation of the MOF particle size between 200 and 1200 nm gave no significant differences in oxygen yield when normalized for total number of active sites, suggesting that catalysis was not limited by charge transport hopping kinetics. Additional work by the group of Morris demonstrated that charge transport can, however, indeed be limited to a surface-confined regime when the Ru-to-Ru distance exceeds a certain percolation threshold, that is, if the distance between the redox active hopping sites is too large, thereby slowing down electron transport significantly. Increasing the amount of redox-active Ru catalysts will move the system into a regime where charge transport into the MOF interior is no longer limiting.^256^

In another report, Chen et al.^353^ prepared a series of **(Co)PCN222** frameworks with varied particle sizes spanning from 200 to 1000 nm via a coordination control synthesis technique. The ORR activity of these particles has been found to be proportional to their size. The smallest particle sized MOF had the highest mass activity, while the largest one displayed the highest surface area normalized activity of the three sizes tested. In this example, the correlation between catalytic activity and crystal size was suggested to be due to a noticeable decrease in the electrolyte ions’ diffusion distance and an increase in the active material’s specific surface area.

**Figure 35:**
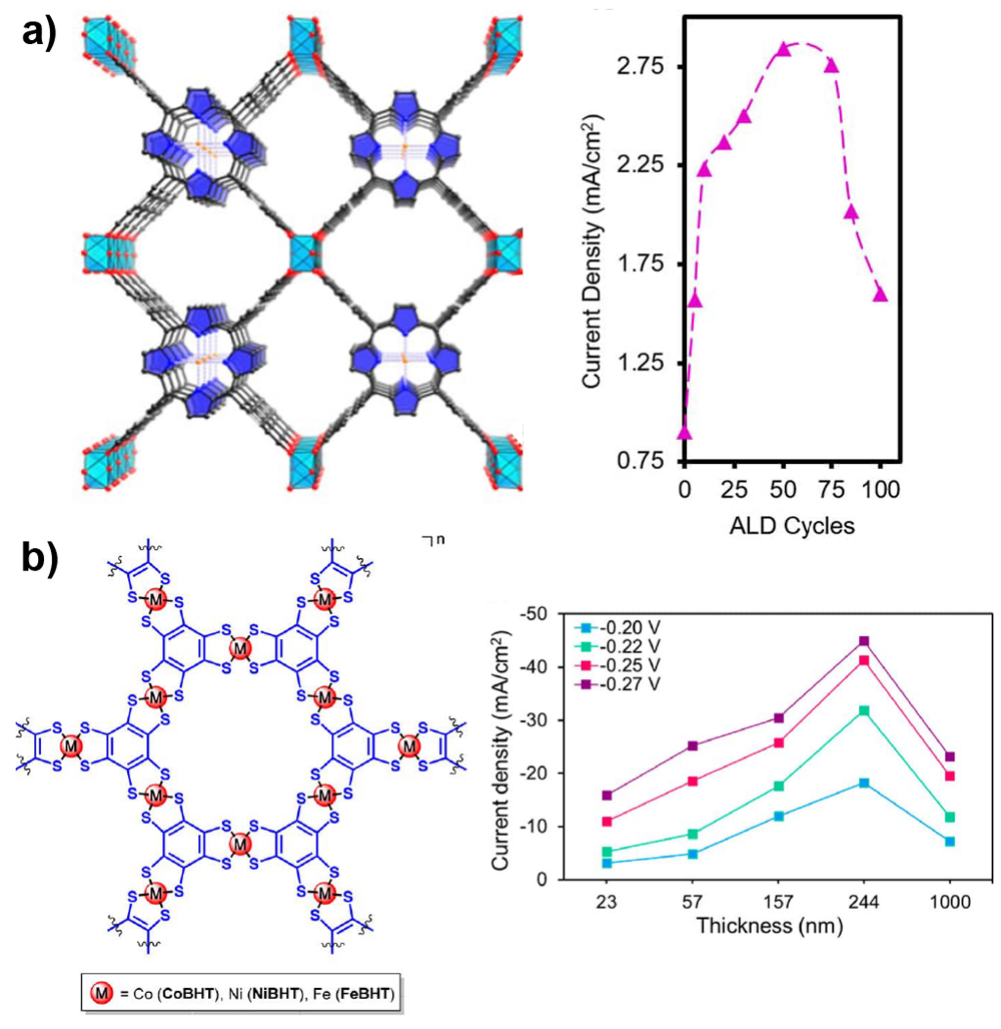
a) **Al**_**2**_**(OH)**_**2**_**CoTCPP** MOF and current densities obtained from the electrocatalytic reduction of CO_2_ from thin films. Maximum activity is obtained from films produced from 50 ALD cycles of the Al_2_O_3_ precursor.^354^ b) Structure of **CoBHT** and comparison of the current density (mA cm^–2^) versus thickness (nm) at −0.20 V vs RHE (blue), −0.22 V vs RHE (green), −0.25 V vs RHE (pink), and −0.27 V vs RHE (purple).^355^ Adapted with permission from the respective references. Copyright 2015 and 2018 American Chemical Society, respectively.

Two systematic reports on the correlation between MOF film thicknesses and catalytic current densities for thin MOF films have been reported. In 2015, Kornienko et al.^354^ reported the cobalt-porphyrin MOF **Al**_**2**_**(OH)**_**2**_**CoTCPP**, grown on a conducting substrate as a selective electrocatalyst for the reduction of CO_2_ to CO in aqueous media. The **Al**_**2**_**(OH)**_**2**_**CoTCPP** were made by the initial deposition of a known amount of Al_2_O_3_ on the electrode surface by ALD, followed by exposure to the H_2_TCPP linker. By controlling the amount of ALD cycles, and thus the amount of available Al^3+^ cations, films of different film thicknesses could be prepared. It was found that ultrathin films exhibit relatively small current densities, which increase with increasing film thickness to a maximum value after which current densities start to drop again (Figure 35a). This behavior was interpreted in that the catalytic performance is maximized at a starting layer thickness of 50 ALD cycles, which offers a balance of charge transport, mass transport, and active-site density.

The importance of film thickness on the efficacy of MOF-based catalysts has also been demonstrated on a series of **CoBHT**-modified GCEs. **CoBHT** is a 2D-MOF consisting of benzenehexathiolate linkers (BHT) that are interconnected by Co^II^-cations. These sheets stack to form films, the thickness of which was systematically varied in a study by Downes et al.^355^ It was found that the initial increase in film thickness from 23 to 244 nm decreased the overpotential to achieve 10 mA cm^–2^ from 246 to 185 mV (Figure 35b). This finding is consistent with an increase of the number of catalytically active sites, as expected from an increase in film thickness. However, increasing the film thickness further to 1000 nm did not lead to a further increase in the number of electrochemically accessible active sites, and a reduced HER activity was observed, quantified by an increased overpotential of 213 mV. In this regime, charge transport and proton permeation through the thick films limit high electrocatalytic activity. The thickness-dependent HER activity as observed for the **CoBHT** films is an experimental manifestation of how transport limitations can become dominant in electrodes for electrocatalytic HER.

In addition to the reports above, further example that benefit from decreased dimensions to lessen transport limitations have been reported. Taken advantage of this strategy, ultrathin nanosheet **NiFe-MOF** arrays, consisting of metal–oxygen-layers (MO_6_ units; M = Ni or Fe, in a Fe/Ni ratio of 23%) interconnected by 2,6-naphthalenedicarboxylate linkers, have been fabricated on different substrates through a dissolution–crystallization mechanism.^356^ The metal ions (Ni, Fe) are octahedrally coordinated, and each metal ion is coordinated to two trans monodentate carboxylates and four equatorial water molecules, while each naphthalene dicarboxylate bridges two metal atoms. These materials exhibit intriguing properties for electrocatalysis including highly exposed active molecular metal sites owning to ultrasmall thickness of nanosheets, improved electrical conductivity and a combination of hierarchical porosity. The **NiFe-MOF** array demonstrated electrocatalytic HER performance with a small overpotential of 134 mV at 10 mA cm^–2^.^356^ The same system was also found to be highly active for electrochemical oxygen evolution from a 0.1 M aqueous KOH solution.

Another example of a nanometer sized catalyst layer is a conductive **Cu-MOF** with HHTP linkers that was prepared as a shell around presynthesized Cu_2_O nanocube cores. Subsequent removal of the Cu_2_O core by a redox-etching approach in the presence of Fe^III^ produced a thin Fe(OH)_*x*_ shell underneath the surface Cu-MOF layer. The resulting **Fe(OH)**_*x*_**@Cu-MOF** nanoboxes catalyzed the electrochemical HER with low overpotentials, and X-ray absorption spectroscopy and DFT calculations indicated coordinatively unsaturated Cu–O centers as highly active catalytic sites that accelerate the formation of key *H intermediates toward fast HER kinetics.^357^

In 2020, Kang et al.^358^ reported the growth of the copper paddle wheel MOF **Cu**_**2**_**(L)** (H_4_L = 4,4′,4″,4‴-(1,4-phenylenebis(pyridine-4,2,6-triyl))tetrabenzoic acid) on a Cu-foam electrode by electrosynthesis in the presence of the ionic liquid EmimOAc (1-ethyl-3-methylimidazolium acetate). Owing to the electrosynthesis conditions, **Cu**_**2**_**(L)-e/Cu** featured structural defects throughout the MOF film, affording undercoordinated Cu^II^ centers which show strong interactions with CO_2_. **Cu**_**2**_**(L)-e/Cu** was able to reduce carbon dioxide to formic acid with a current density of 65.8 mA cm^–2^ and a FE of 90.5% for formic acid after 2 h. In comparison, **Cu**_**2**_**(L)-t** that was synthesized in a conventional solvothermal way and loaded onto carbon paper (CP) to form **Cu**_**2**_**(L)-t/CP** exhibited a considerably lower current density. Electrochemical impedance spectroscopy measurements revealed that **Cu**_**2**_**(L)-e/Cu** showed a significantly lower charge-transfer resistance compared to the **Cu**_**2**_**(L)-t/CP**, reflecting the microscopic morphology of **Cu**_**2**_**(L)-e/Cu**, that form a compact and thin coating of the Cu-foam electrode, instead of the **Cu**_**2**_**(L)-t**, which was prepared as larger crystals. In a follow-up paper, the same group further explored the technology to prepare catalytically active MOF materials by electrosynthesis. In that paper, a **MFM-300(In)-e/In** composite material was prepared by the electrosynthetic growth of **MFM-300(In)** on an indium foil in the presence of a biphenyl-3,3′,5,5′-tetracarboxylic acid linker.^359^ Owing to a templating effect by the ionic liquid EmimOAc that was used during electrosynthesis, the **MFM-300(In)** contained highly active In^3+^ defect sites. **MFM-300(In)-e/In** was found to be a good catalyst for the electrochemical CO_2_RR to formic acid, with a current density of 46.1 mA cm^–2^ at −2.15 V vs Ag/Ag^+^ and a FE of 99.1%. The high activity of **MFM-300(In)-e/In** is supported by a low interfacial charge-transfer resistance of 9.5 Ω cm^–2^.

Ye et al.^360^ reported the incorporation of a [Re(bpydc)(CO)_3_Cl] CO_2_ reduction catalyst into surface-grafted MOF (SURMOF) thin films on conductive FTO electrodes by a liquid phase epitaxy method. The thereby obtained catalytically active thin films were found to be highly oriented, grown exclusively along the [001] direction. Charge transport by a redox hopping mechanism along the [001] direction is particularly efficient, and consequently, the **Re-SURMOF** performed well as an electrocatalyst for the reduction of CO_2_ to CO.

In 2016, Zhao et al.^361^ developed ultrathin MOF nanosheets (UMOFNs), comprising Ni^2+^, Co^2+^, and BDC, for the electrocatalytic OER under alkaline conditions. The NiCo-UMOFNs showed high electrocatalytic activity as well as long-term stability. While installed on GCEs, the NiCo bimetallic UMOFNs required an overpotential of 250 mV to reach a current density of 10 mA cm^–2^. Loading the catalyst on copper foam enables decreasing the overpotential to 189 mV. At a constant overpotential of 250 mV, a highly stable current density was obtained for at least 200 h, with a high associated FE of 99.3%. Supported by, for example, X-ray spectroscopy and DFT calculations, the authors proposed that the surface atoms in the MOF nanosheets are coordinatively unsaturated and the active catalytic centers. Further was the electronic interplay between the Ni and Co atoms suggested to be crucial for the electrocatalytic activity. To substantiate this, the single-metal-binding motifs, **Co-UMOFNs** and **Ni-UMOFNs**, were prepared as well and showed lower activity.

In 2018, Zhu et al.^362^ employed exfoliated nanosheets from the conductive 2D-MOF **Ni**_**3**_**(HITP)**_**2**_ (HITP = 2,3,6,7,10,11-hexaaminotriphenylene) as an efficient CO_2_RR catalyst to produce CO in high selectivity of 97%. The reaction was light-driven and performed with [Ru(bpy)_3_]^2+^ as a PS and TEOA as an electron donor. The dimension of the **Ni**_**3**_**(HITP)**_**2**_ nanosheets provided high conductivity and highly accessible Ni–N_4_ active sites, giving rise to an efficient catalytic performance of **Ni**_**3**_**(HITP)**_**2**_. In 2015, Zhang et al.^363^ described the self-assembly of the flower-like bifunctional **Ru-MOF**, [Cd_2_(Ru(bpy-4,4′-dc)_3_)·12H_2_O]_*n*_, from nanosheets of the same material. The nanoflower **Ru-MOF** catalyzed the light-driven reduction of CO_2_ to formate in ca. 150% higher yields than solid-micro crystalline versions of the same **Ru-MOF**. Moreover, the nanostructure enhanced the stability of the agglomerated nanosheets, which gives rise to higher photostability and recyclability of the photocatalyst.

#### 3.2.2 Engineering Transport Pathways

In the case when the reaction of energy relevance is driven by a chemical reductant or oxidant, these species have to physically diffuse into the MOF matrix to engage all catalysts in the reaction at hand. Similarly, electron donors or acceptors need to diffuse into the MOF in light-driven transformations that are initiated by PSs inside the MOF crystal, or photoactive MOFs. The transport of these sometimes rather large entities will more often than not result in severe transport limitations. Finally, also electrons and holes in electrocatalytic systems have to be transported to the active sites, and strategies to accelerate this process are in high demand. In enzymes, mass and charge transport often occur in well-defined channels^364^ and between redox-active cofactors,^365^ respectively. In the case of hydrogenases, the redox potentials of the [4Fe4S] clusters are adjusted to give the enzyme an inherent redox gradient that contributes to bias the enzyme toward either the anodic or cathodic reaction. Such a level of sophistication has not been realized in MOFs in which gradients may, however, develop dynamically under operation.^60^ The fundamental principles behind transport phenomena in MOFs are outlined in section 2.2 of this review.

In the case an oxidant or a reductant has to physically diffuse through the MOF crystal, ion pairing can lead to pore clogging. This phenomenon was shown on a **NH-MIL-101(Cr)** into which an [FeFe] hydrogenase active site mimic was incorporated. Electron transfer between the neutral [FeFe] site and neutral cobaltocene afforded the reduced [FeFe]^−^ species and oxidized cobaltocenium ion, which formed ion pairs that were stable on the time scale of hours, as shown by altering IR spectra over these time scales. This effect was shown to be lessened in **NH-MIL-101(Cr)-[FeFe]** with lower [FeFe] loadings.^198^

The role of mass transport as a phenomenon potentially limiting the light-assisted HER has been addressed in a **Ru-NH**_**2**_**-MIL-125(Ti)** series of MOFs.^366^ Even though the exact molecular structure of the catalyst in these systems is not entirely clear, an interesting transport phenomenon was observed. In order to prepare these materials, pristine **NH**_**2**_**-MIL-125(Ti)** powder was treated with RuCl_3_·*x*H_2_O in a supercritical CO_2_/methanol solution. This treatment did not only introduce the Ru catalyst into the MOF but also led to a certain degree of hollowness of the **Ru-NH**_**2**_**-MIL-125(Ti)**, as observed by TEM. The hollowness is attributed to the formation of mesopores in the MOF, a process that is only observed under supercritical fluid conditions in the presence of RuCl_3_·*x*H_2_O. The effect becomes increasingly pronounced as the processing time is increased. The presence of mesopores reduces the Brunauer–Emmett–Teller surface areas of the materials but increases their pore volumes significantly. From a biomimetic perspective, such pore enlargements could be compared to the presence of channels in enzymes. In comparison to **NH**_**2**_**-MIL-125(Ti)**, into which a Ru nanoparticle had been loaded by conventional methods, the **Ru-NH**_**2**_**-MIL-125(Ti)** catalyst that was prepared under supercritical fluid conditions exhibited a higher light-driven HER activity in the presence of TEOA. While the incorporated Ru centers contribute to the electronic structure of the MOF, the highly mesoporous structure of **Ru-NH**_**2**_**-MIL-125(Ti)** also contributes to accelerating catalytic reaction by enhancing both the exposure of catalytic active sites and the facilitated transport of the TEOA donor.

In 2019, Zhong et al.^367^ synthesized a Cu phthalocyanine-based 2D conjugated MOF, **PcCu-O**_**8**_**-Co**, displaying a layer-stacked structure that featured Co-bis(dihydroxy) complexes (Co–O_4_) as linkages. When contacted on an electrode with carbon nanotubes (CNTs), **PcCu-O**_**8**_**-Co** is a decent catalyst for the ORR in alkaline solutions. A possible reason for the excellent performance was the presence of dimensionally controlled micropores (1.5 nm) in combination with mesopores (2–10 nm) in **PcCu-O**_**8**_**-Co**, both of which are favorable for substrate and electrolyte transport during the ORR.

Identifying factors that govern the diffusional charge hopping transport, as well as identifying means to accelerate this process has been subject of intense research,^117^ and reports in context of catalysis are reviewed hereafter. First, various reports address the dependency of material crystallinity and crystal orientation relative to the electrode surface as factors that govern diffusional electron hopping charge transport. Also here, parallels can be drawn between the presence and directionality of channels in enzymes, and diffusion pathways in MOFs that are disrupted when the material is poorly crystalline. It has also been shown that partial pore collapse at the crystal surface can lead to surface barriers that impede mass and ion ingress into the MOF,^61^ thereby slowing down catalysis. In 2020, Park et al.^368^ presented 2D MOF systems, consisting of M-N_4_ moieties (M = Ni, Cu) and hexaaminobenzene (HAB) linkers. The authors conducted studies to find correlations between crystallinity and conductivity, and the effect on the materials activities as ORR catalysts. Highly crystalline **Ni-HAB-H** exhibited high activity, enhanced stability, and better electron conduction compared to its low crystalline analogue (**Ni-HAB-L**), most likely due to amorphous features of **Ni-HAB-L** resulting in less exposed active sites and slow mass transport. Additionally, theoretical modeling suggested that along with metal cations as active center, the in-plane linker site can also act as catalytically active site for the ORR.

Meng et al.^369^ reported a porous Co framework ([Co_1.5_(tib)(dcpna)]·6H_2_O), based on 1,3,5-tris(1-imidazolyl)-benzene (tib) and 5-(3′,5′-dicarboxylphenyl)nicotinic acid (H_3_dcpna), as an OER electrocatalyst. A high OER activity was ascribed to 1D open channels along the *b* axis of the MOF, which exposed more active sites and facilitates electrolyte penetration. A comparison of the material with a speculative interpenetrated analogue could support this hypothesis.

The fact that charge hopping transport is coupled to diffusion–migration of charge-balancing electrolyte counterions has been found to also affect electrochemical catalysis. Such findings are indicative that charge transport is limiting in such systems. The first report that addressed this topic was as early as in 2011 by Nohra et al.^370^ The report is based on three MOFs that were prepared from POMs and tridentate 1,3,5-benzene tricarboxylate linkers, capped by Zn^2+^ ions. The channels of the POM-based MOFs (POMOFs) are occupied with tetrabutylammonium (TBA) counterions. It turned out that carbon paste electrodes of one of the POMOFs showed a remarkably high electrocatalytic activity in 1 M lithium chloride at pH = 1 (HCl). Lithium chloride as a supporting electrolyte was important for high current densities, as a change to sodium and potassium led to a decrease in activity by a factor of 2.8 and 2.5, respectively. The effect was ascribed to the sizes of the hydrated alkali ions as well as their propensity to carry water molecules into the POMOF. The relative hydrated ionic radii for alkali ions are in the order Li^+^ (340 pm) > Na^+^ (276 pm) > K^+^ (232 pm). Compared to the radius of the unhydrated TBA (494 pm) in the as-prepared POMOF, hydrated Li^+^ appeared as the cation most likely to ensure POMOF stability. In addition, the large and firmly hydrated Li^+^ ions will carry the largest number of water molecules into the structures, a circumstance that is favorable for HER electrocatalysis.

Another example of introducing electroactive units into a catalytic MOF to increase charge transport was reported by Mukhopadhyay et al.^371^ In this work, **ZIF-8** functions as a host, Fe-salen as the active OER catalyst, and the Keggin SiW_12_ POM was incorporated to facilitate charge transport, as shown by lower overall electrical resistance of the resulting composite system.

**Figure 36:**
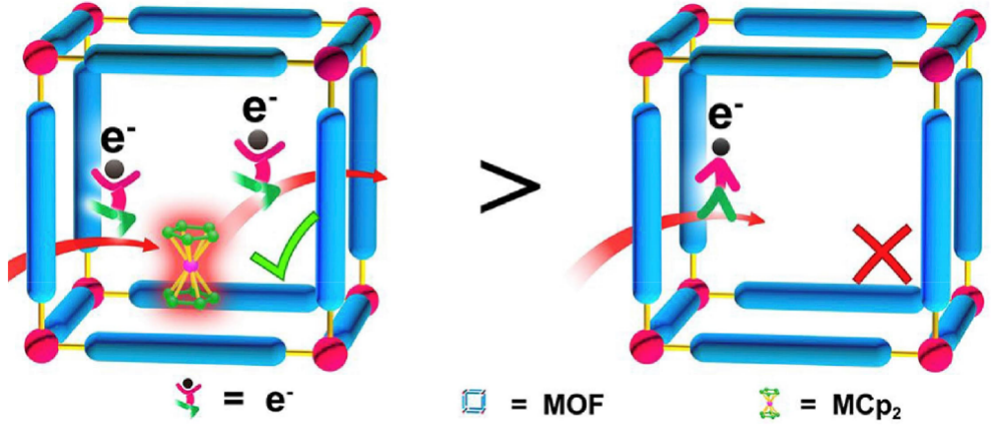
Enhanced electron transfer in **MCp**_**2**_**@MOF** through the introduction of electron-rich metallocenes, promoting the electrocatalytic CO_2_ reduction. Adapted with permission from reference (372). Copyright 2020 Elsevier.

Following a different strategy, Xin et al.^372^ reported the introduction of electron-rich metallocenes into the pores of MOFs by chemical vapor deposition. Following this strategy, a series of **MCp**_**2**_**@MOF-545-Co** (**MOF-545-Co** = Zr_6_O_8_(H_2_O)_8_(CoTCPP)_2_, M = Fe, Co, Ni) frameworks were prepared and were shown to be effective CO_2_RR electrocatalysts. The MCp_2_ moieties serve as additional electron hopping transport sites, creating electron transport channels within the MOF toward the active metalloporphyrin sites (Figure 36). In addition, the presence of the metallocenes in close proximity to the MOF-linkers leads to a reduced CO_2_ adsorption energy, as revealed by DFT calculations, and thereby contributes to an enhanced electrocatalytic performance.

While the above papers relied on charge transport by a diffusion-like charge hopping mechanism between discrete redox active units, reminiscent of charge transport between redox active cofactors in enzymes, the following papers rely on charge transport by different mechanisms. In 2018, Dincǎ and co-workers^373^ reported 2D hexagonal MOFs, Ni_3_(HITP)_2_, and Cu_3_(HHTP)_2_ along with Co_3_(HHTP)_2_ and Ni_3_(HHTP)_2_ as trigonal MOFs with high intrinsic band-like conductivity. Such semiconductor-like charge transport is unprecedented in nature, but conceivable in MOFs due to extended conjugation paths. The dependence (nonzero order) of ORR overpotential on [H^+^] during reduction with the hexagonal MOFs suggested that electron transfer during ORR is proton-coupled while trigonal MOFs lack the electron delocalization in the ab plane and hence disruption of the π-stacking in the c direction was observed.

In 2021, Zhao et al.^374^ reported a π–d conjugated **truxone–Cu MOF** that also showed band-like conduction with semiconductor characteristics. The 2D MOF, consisting of 2,3,7,8,12,13-hexahydroxyl truxone linkers interconnected by copper ions, showed typical characteristics of a semiconductor with an intrinsic conductivity of 4.0 mS cm^–1^ at 30 °C and a small energy gap of 0.24 eV as determined by DFT calculations. Owing to its good electrical conductivity and redox reversibility of both the truxone and the metal center, **truxone–Cu MOF**-modified electrode was successfully applied to catalyze the ORR.

In 2020, Wang et al.^375^ reported coordinatively unsaturated **Co-BTC-IMI** MOFs with high intrinsic activity for both the ORR and OER. The material consisted of BTC linkers that interconnected the Co^II^ cations, aided by imidazoles that are integral to the structure, mainly incorporated by π–π interactions with the benzene rings of the BTC linker. The high activity of **Co-BTC-IMI** for the electrocatalytic ORR and OER was ascribed to accessible coordination sites at the catalytic Co centers as well as good electric conductivity that is promoted by the through-space π–π interactions.

In 2018, Wang et al.^376^ presented a series of POM-porphyrinic MOFs, so-called **PMOFs**, synthesized by a hydrothermal method that links redox active Zn-ε-Keggin clusters (ε-PMo^V^_8_Mo^VI^_4_O_40_Zn_4_) as SBUs with metalloporphyrin linkers. The combination of these two structural units in this **PMOF** leads to outstanding electron transport properties. Of the metalloporphyrin linkers tested, the Co-porphyrin **Co-PMOF** exhibited the most impressive CO_2_RR performance with a TOF of 1656 h^–1^ and a faradaic efficiency for CO of >94%. Remarkably, **Co-PMOF** catalyzed the electrochemical CO_2_RR to CO at a constant current density of 17 mA cm^–2^ for 36 h without any significant drop in stability.

In 2020, Das and co-workers^371^ described the in situ coencapsulation of the WOC catalyst Fe-salen (i.e., Fe(salen)Cl) and the Keggin POM SiW_12_ (i.e., H_4_[SiW_12_O_40_]) into the pores of the zeolitic network **ZIF-8** to form **FSWZ-8** ([Fe(salen)(OH)]+H_4_[SiW_12_O_40_]·HCl)@ZIF-8), an efficient and robust electrocatalyst for the OER at a neutral pH. Incorporation of SiW_12_ resulted in accelerated formation of **FSWZ-8**, higher catalyst loadings, and in the lowering of the required overpotential for the electrochemical OER by more than 150 mV. Furthermore, the POMs lower the electrical resistance and hence ease the charge transport. The composite system exhibited high TOFs of around 5 s^–1^ and FE of overall 94% during electrocatalytic water oxidation.

Catalysis of energy relevance inevitably involves protons. In order to not become limiting, their facile transport through the MOF crystal is of vital importance. In fact, MOFs as proton conductors is a field of research in itself, with some excellent reviews having emerged recently.^377,378^ One interesting example is that of **UiO-66-NH**_**2**_ that was decorated with sulfonate groups.^379^ Two materials were prepared that differed by the length of the carbon chain between the −SO_3_H group and the amino group of the BDC-NH_2_ linker. It was found that the material with the shorter C_2_H_4_ linker exhibited a record proton conductivity of 1.64 × 10^–1^ S cm^–1^ at 80 °C, while that of the C_3_H_6_ analogue was 4.6 × 10^–3^ S cm^–1^. Even though this report does not directly address catalysis, it teaches an important lesson in that modifications that may seem rather insignificant (ethyl versus propyl tether) can hugely impact performance. In the present case, if catalysis had been limited by proton diffusion, choosing the right alkyl sulfonate tether would have increased the material’s TOF.

The effect of proton sources on the electrocatalytic ORR was also investigated by Morris and co-workers in 2017.^380^ Using **PCN-223-Fe**, a MOF consisting of Zr_6_-oxo clusters and Fe^III^-porphyrin linkers, which was synthesized on a conductive FTO substrate, it could be shown that the ORR activity increased with increasing strength of the acid that was added to the electrolyte solution. Interestingly, the use of the stronger trifluoroacetic acid gave a lower product selectivity than that promoted by the weaker acetic acid. For the latter, the amount of H_2_O_2_ was less than 6%, while the former acid gave rise to 34%. The results show the importance to not only supply protons to the catalytic centers at appreciable rates, but also at appropriate activity to warrant good product selectivity.

One example exists wherein two isostructural MOFs that differ in the constituting metal of the SBU (zinc or cobalt) were prepared and directly compared for the OER.^381^ Notably, the **FJU-82-Co** had a ca. 300 mV lower overpotential relative to the **FJU-82-Zn** analogue, yet had only a slightly higher electronic conductivity (1.96 × 10^–12^ vs 1.67 × 10^–12^ S cm^–1^, respectively). Subsequent measurement of the two MOFs by alternative current impedance spectroscopy revealed that **FJU-82-Co** had a proton conductivity 1 order of magnitude larger than that of the Zn analogue. While Zn has a lower intrinsic OER activity than Co, the authors ventured that since the OER mechanism involved release of a proton, the rate of the OER could be limited by proton transport out of the MOF. **FJU-82-Co** had a larger unit cell, suggesting that solvated protons would diffuse more quickly than within **FJU-82-Zn**. While the authors did not measure the diffusion rate of water (the OER substrate) in their two MOFs, it is possible that the water diffusion rate was also larger for **FJU-82-Co** in addition to having a superior proton conductivity.

**Figure 37:**
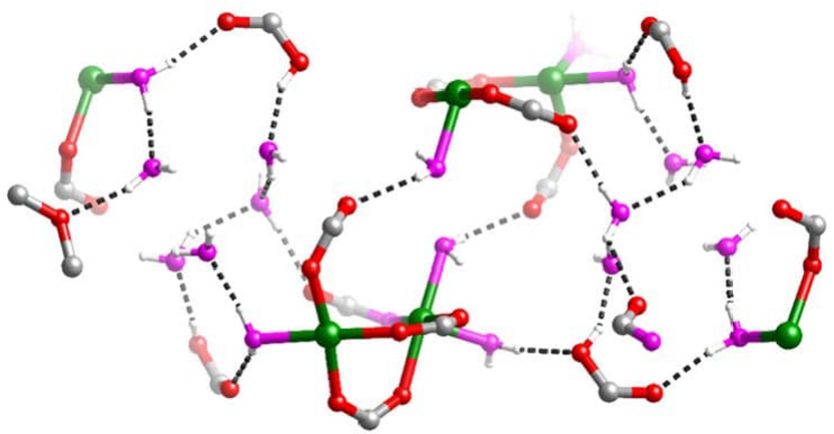
Crystallographic structure of the hydrogen bonding network of the coordination polymer **Co-HL**. Atom labeling: Co, green; C, gray; H, white; O (ligand), red; O (water), pink. Adapted with permission from reference (382). Copyright 2020 American Chemical Society.

Furthermore, hydrogen bonding within MOFs for OER has been observed and postulated. The single crystal structure of the cobalt coordination polymer **Co-HL** ([(Co_2_(HL)(H_2_O)_5_)3H_2_O]*_n_*), synthesized from H_5_L (= 5,5′-[(5-carboxy-1,3-phenylene)bis(oxy)]diisophthalic acid), exhibited a clearly visible hydrogen bonding network (Figure 37).^382^ Comparison of the linear sweep voltammograms of this coordination polymer with its deuterated analogue **Co-DL** revealed a lower overpotential requirement for **Co-HL**, strongly suggesting that the hydrogen bond network near the presumed active metal sites was directly involved in OER, thereby being reminiscent to enzymatic chemistry. Related work has found further evidence of proton shuttling and proton binding in MOFs for the OER.^383−385^

A metal–organic cage (MOC) [Pd_6_(RuL_3_)_8_]^28+^ (**MOC-16**; L = 2-(pyridin-3-yl)-1*H*-imidazo[4,5-*f*][1,10]-phenanthroline) consisting of six Pd vertices and eight Ru^2+^-based photosensitizing units that cover one face of the cage each has been incorporated into the Zn^2+^/methylimidazole (MeIm)-based **ZIF-8**.^386^ The **ZIF-8** is constructed around the MOC through coordination-assisted secondary assembly, where **MOC-16@ZIF-8** precursors are initially grown from a mixture of **MOC-16**, Zn^2+^, and 2-MeIm, which then crystallize to the **MOC@MOF**. Subsequent partial substitution of MeIm in the **ZIF-8** by carbonate ions gives rise to CZIF with molecularly intact **MOC-16** in the CZIF scaffold. The transformation of the **ZIF-8** to the CZIF is of crucial importance for the photocatalytic HER activity of the MOC. While the **ZIF-8** matrix is highly hydrophobic and does not support light-induced HER in the presence of electron donors, the hydrophilicity that is introduces into the CZIF matrix by the constituting CO_3_^2–^ ions guarantees sufficient proton transport to the catalytic sites. In other words, the hydrophilic character of the carbonate ions in the CZIF structure is integral to a H-bonded water network that assists in proton transport to catalytic sites.

### 3.3 Cooperative Effects

In this section, original reports are reviewed in which the active site interacts with the surrounding MOF matrix in a way that alters the catalytic mechanism as compared to the situation in homogeneous solution phase. Specific catalyst–matrix interactions that, for example, stabilize transition states in the catalytic cycle, offer alternative mechanistic routes, or promote proton-coupled electron transfers pathways are being discussed. Such cooperative effects go beyond “simple” stabilization of the molecular integrity of the catalyst and intermediates as discussed in section 3.1, and are one of the strongholds of enzymatic chemistry. The latter is famous for intricately designed active site pockets, within which amino acid side chains may stabilize transition states or temporarily host substrates like protons. In the context of MOFs, careful design can enable hydrogen bond formation and noncovalent interactions through changes in the microenvironment within the pores to activate substrates, stabilize intermediates, and thereby enhance catalytic efficiencies. The section also includes reports of SBU-based catalysts, the thermodynamics of which are altered by the surrounding MOF matrix, as manifested, for example, by linkers that enforce a coordination geometry on the SBU that is different to the relaxed structure in solution. Finally, this section also contains reports in which the MOF matrix is shown to impart a more global effect on the molecular catalyst, for example by controlling solvation or concentrating substrate, which are effects that resemble outer coordination sphere effects in enzymes.

#### 3.3.1 Microenvironment-Induced Tuning of Catalyst Performance

Historically, MOFs have first been investigated for their gas sorption activities. It is thus not surprising that this effect has also been explored in the context of CO_2_RR activity. Kajiwara et al.^387^ demonstrated the synergistic effect between gas adsorption properties and catalytically active sites in a **UiO-67**-based MOF, modified by the CO_2_ reduction catalyst [Ru^II^(H_2_bpydc)(terpy)(CO)]^2+^ (H_2_RuCO). The catalytic activity of the obtained **Zr-bpdc/RuCO** composite was not only comparable to the corresponding homogeneous system, but was in contrast maintained even under low CO_2_ pressure of a 5% CO_2_/Ar gas mixture, presenting an important opportunity for the use of low-concentration CO_2_ streams as C1 sources.

In 2020, Zhang et al.^388^ described the postsynthetic modification of the 2D MOF nanosheets **Zr-BTB** by anchoring the cobalt porphyrin-derived catalysts CoTCPP to the Zr_6_ cluster SBUs. The resulting material, **CoTCPP/Zr-BTB**, was a good catalyst for the electrochemical reduction of CO_2_ to CO, significantly outperforming the molecular reference system. The microenvironment around the CoTCPP catalysts was further tuned by postsynthetic coordination of a selection of carboxylate-containing ligands to the SBUs. Of the modified 2D MOFs that were tested, the ones with *p*-sulfamidobenzoicacid (PSABA) ligands, **CoTCPP/Zr-BTB-PSABA**, showed the best performance with FE_CO_ that increased by about 10% compared to the system that lacked the additional carboxylate ligand. This increase in FE_CO_ is accompanied by a decrease of the material’s HER activity, suggesting that HER pathways at the SBUs are shut down by the postsynthetic ligation, thereby improving the utilization of electrons for the CO_2_RR.

Huang et al.^338^ reported the synthesis of a series of 3D pillared-layered monometallic (**SyA-Co** and **SyA-Fe**) and bimetallic CoFe MOFs with syringic acid (SyA) linkers. It was found that the optimal Co:Fe ratio for the performance of the materials as electrochemical OER catalyst was 2:1 (**SyA-Co**_**2**_**Fe**). Besides the series of pristine MOFs, an N,S codoped version of the CoFe-MOF was prepared by adding thiourea into the solvothermal mixture. These N,S codoped materials, (**SyA-Co**_**2**_**Fe-ST**), showed a remarkable increase in the OER performance compared to the pristine MOF **SyA-Co**_**2**_**Fe**, with low overpotentials and a long-term stability for at least 16 h. The improved electrocatalytic activity of **SyA-Co**_**2**_**Fe-ST** was mainly attributed to in situ surface modifications that alter the pore structures, tune the electronic and surface structures of the catalysts, while also promoting the formation of new active species and facilitating charge and mass transport for the OER process.

Zhong et al.^389^ demonstrated a synergetic effect in a series of layer-stacked 2D **PcM-O**_**8**_**-M** (Pc = phthalocyanine; M = Cu or Zn) frameworks that can be exploited for the electrocatalytic CO_2_RR to CO. These 2D MOFs consist of zinc/copper phthalocyaninato linkers and zinc/copper-bis(dihydroxy) complex (ZnO_4_) SBUs. Spectroscopic studies combined with DFT calculation revealed that the ZnO_4_ complexes of **PcCu-O**_**8**_**-Zn** exhibit high catalytic activity for CO_2_-to-CO conversion, while CuN_4_ complexes in the Pc macrocycles act to promote proton and electron transfer during the reaction process. **PcCu-O**_**8**_**-Zn** in combination with carbon nanotubes (**PcCu-O**_**8**_**-Zn/CNT**) exhibited highly selective catalytic activity for CO_2_-to-CO conversion (88%), high TOFs, and excellent stability. By varying the metal centers (Cu and Zn) of ligand/linkage as well as applied potentials, the H_2_/CO ratio could be tuned from 1:7 to 4:1. Interestingly, the authors found that the CuN_4_ linker in **PcCu-O**_**8**_**-Zn** shows the lowest energy barriers for HER, while also promoting the lowest free energy for the generation of rate-determining *COOH intermediate at the ZnO_4_ SBU. It is this synergistic effect that is suggested to result in the good CO_2_RR activity of **PcCu-O**_**8**_**-Zn/CNT**.

#### 3.3.2 Lattice Effects

Lattice effects have been observed in the photochemical and photoelectrochemical OER using MOFs. In one example, the HOMO–LUMO gap of the triphenylene-2,6,10-tricarboxylic acid (H_3_TTCA) organic linker decreased upon MOF incorporation (LUMO = lowest unoccupied molecular orbital).^290^ On its own, H_3_TTCA is not catalytic for oxygen evolution, but once inside a MOF it could be photoexcited and drive photochemical OER. In a related example, the HOMO of the porphyrin linker within a **MOF-545** hybrid material increased in potential relative to that of its homogeneous analogue; the resulting increased HOMO–LUMO gap provided more driving force for the OER.^245^

**Figure 38:**
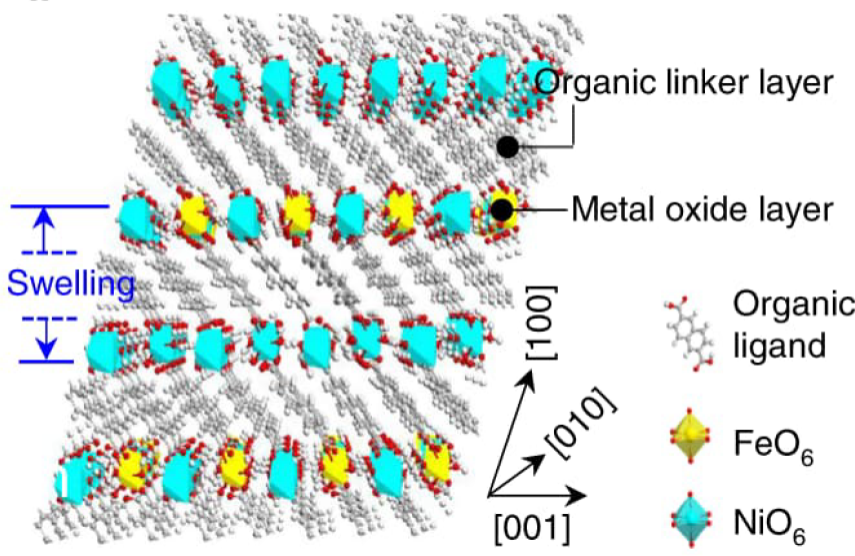
Structure of lattice-strained **NiFe MOF**. Adapted with permission from reference (390). Copyright 2019 Springer Nature.

Lattice strain has also been utilized to tune the electronics for MOF-based OER and ORR. In 2019, Cheng et al.^390^ compared the OER and ORR activity of a **NiFe MOF** to its lattice-strained congeners (Figure 38). Increasing duration of ultraviolet irradiation yielded more strained **NiFe MOFs**. Analysis of the resulting materials suggested that increased strain upshifted the oxygen 2p level, resulting in a stronger covalent interaction between Ni and oxygen. Comparison of their linear sweep voltammograms revealed that increases in strain directly decreased OER overpotentials, and the most strained **NiFe MOF** had better durability than the noble metal RuO_2_ during long-term bulk electrolysis. The lattice-strained **NiFe-MOF** facilitated fast and efficient 4-electron pathways for both the ORR and OER compared to the low-efficiency 2-electron catalytic kinetics for pristine MOFs. This use of the MOF lattice to tune the catalytic site electronics is an excellent example of how the actual MOF structure can be leveraged to improve catalysis, much as how the protein environment around an enzyme’s active site is tuned to support catalysis.

In 2020, Yang et al.^391^ demonstrated the influence of lattice strain on the electrocatalytic CO_2_RR activity of a Zn-porphyrinic MOF. The strain was introduced by a vaper-phase infiltration (VPI) method in the gas phase. First, the pristine porphyrinic framework **MOF-p** was synthesized starting from a carbon fiber electrode onto which a thin layer of Al_2_O_3_ had been deposited by ALD, followed by its treatment with H_2_TCPP. Subsequently, Zn was introduced into the porphyrinic linkers of pristine **MOF-p** by VPI with diethyl zinc and H_2_O (10 cycles) to afford **MOF-Zn-inf-10c**. To induce lattice strain, additional VPI cycles (*x* = 20, 40, 60, or 80 cycles) resulted in the formation of additional ZnO clusters within the MOF pores (**MOF-Zn-inf-*x*c**). XRD measurements revealed that increasing number of VPI cycles (0–60 cycles) correlated with shifts of the characteristic (201) peak, offering the possibility to fine-tune the internal strain of the resulting MOF materials. The effect of the lattice strain on the intrinsic catalytic activity of the ZnTCPP sites was investigated for the electrochemical CO_2_RR toward CO in a CO_2_-saturated solution (DMF/H_2_O, *v*/*v* = 9:1). Out of the afforded infiltration samples, **MOF-Zn-inf-60c** outperformed the other VPI samples with an overpotential positively shifted by 200 mV and a maximum FE close to 100% at −1.8 V vs SHE (standard hydrogen electrode). Increasing the VPI cycles from 20 to 60 increased selectivity and was argued to be directly linked to the generation of internal strain through gradually filling up of the pores with ZnO clusters.

#### 3.3.3 Discrete Cooperative Effects between Catalyst Linkers and Surrounding MOF Matrix

A discrete cooperative effect between two platinum centers during the HER was reported for **Pt-MOF-253**.^392^ The structure of the MOF is such that its Al-based SBUs position bpydc linkers on top of each other. Upon metalation with PtCl_2_, the Pt(bpydc) metallo-linkers in the resulting **Pt-MOF-253** act both as a PS as well as a HER catalyst. Interestingly, the **Pt-MOF-253** catalyzed the light-driven HER in the presence of TEOA as electron donor circa five times faster than the corresponding reference system with Pt(bpydc)Cl_2_ in homogeneous solution phase. While different factors may be at play that explain the difference in activity, one contributing factor was suggested to be a cooperative effect between two Pt centers at adjacent bpydc linkers. It was proposed that upon excitation and reductive quenching of one metallo-linker, a Pt^III^-hydride intermediate is formed by an oxidative addition pathway. This species interacts with an adjacent Pt^II^ metallo-linker to form a mixed-valence hydride-bridged diplatinum(II,III) intermediate. This hypothesis is supported by low temperature luminescence, studies and extended X-ray absorption fine structure data that suggest a short Pt···Pt distance in **Pt-MOF-253**. The interpretation of the diplatinum intermediate is further supported by the observation that the initial H_2_ evolution rate exhibits a first order dependence on the catalyst concentration at low catalyst loading, while it deviates from a linear relationship upon higher Pt concentrations. The authors, however, also point out that this effect may be due to inefficient reductive quenching that becomes more severe under high Pt conditions.^392^

Another example of catalyst linker interations was reported by Cichocka et al.^393^ in 2020. A new kind of porphyrinic MOF, denoted as **PCN-226**, was prepared from CoTCPP linkers and Zr-oxide chain structures. The latter give rise to good chemical and redox stability of the MOF, and, unlike other Zr-based PCN MOFs, generate a compact packing structure of the porphyrin linkers. As a result, **PCN-226(Co)** had a superior ORR performance compared to previously published cluster-based MOFs. According to theoretical calculations, the spatial arrangement in **PCN-226(Co)** brings porphyrin molecules within a distance of 7 Å, which is favorable for the adsorption of *O, *OH, and *OOH intermediates. The chain structure not only improves the overall stability of **PCN-226(Co)** but also allows the redox-active centers to engage in high reaction kinetics.

**Figure 39:**
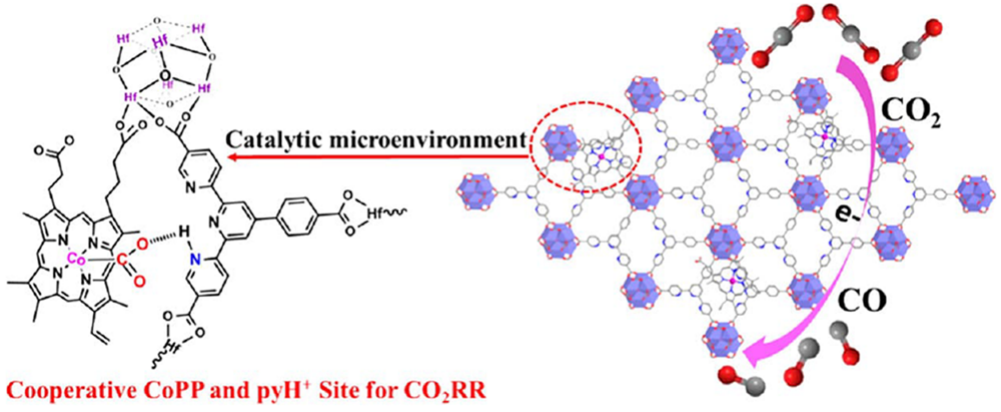
Near proximity between the pyridinium moiety and the CoPP active site leads to a CO_2_-activating microenvironment within **TPY-MOL-CoPP**. Reproduced with permission from reference (108). Copyright 2019 American Chemical Society.

Another cooperative effect was reported by Lin and co-workers^108^ who described the postsynthetic SBU decoration of 2D **TPY-MOL** ([Hf_6_(μ_3_-O)_4_(μ_3_-OH)_4_(HCO_2_)_6_(terpy)_2_] with cobalt-protoporphyrin (CoPP) units via carboxylate exchange. The thus obtained **TPY-MOL-CoPP** features basic pyridine sites as part of the terpyridine-based linkers and the CoPP catalyst anchored to the SBU, but otherwise pointing into the MOF pore. In this spatial arrangement, CO_2_ binding to the CoPP is aided by protonated pyridine moieties (pyH^+^) to coactivate CO_2_ by the formation of the [pyH^+^-^–^O_2_C-CoPP] adduct (Figure 39). This interaction facilitates the CO_2_RR over the HER, hence leading to a CO/H_2_ selectivity of more than 80%. The control experiment with **BTB-MOL-CoPP** that lacks the pyridine moieties, and thus the hydrogen bond interactions, resulted in a lower CO/H_2_ selectivity of 2.7:1, illustrating the effect of the catalytic pocket.

**Figure 40:**
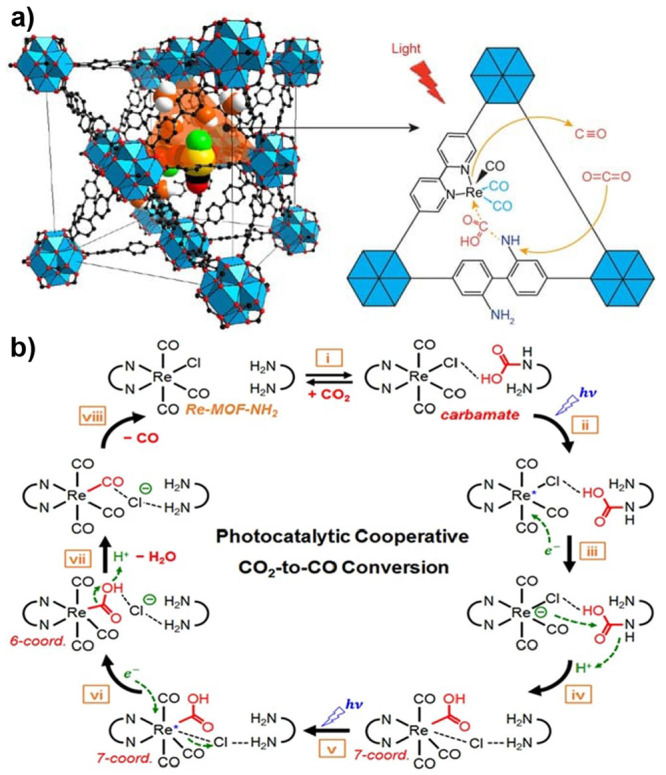
a) Structure and cooperative interaction between an amino-functionality of H_2_bpdc-(NH_2_)_2_ and H_2_ReTC within **Re-MOF-(NH**_**2**_**) (X%)**, employed in the photocatalytic CO_2_-to-CO conversion. Atom labeling: C, black; O, red; Zr, blue polyhedra; Re, yellow; Cl, green. H atoms are omitted for clarity. b) Proposed mechanism for the amine-assisted conversion of CO_2_ to CO based on DFT calculations. Adapted with permission from reference (394). Copyright 2017 Springer Nature.

In 2017, Ryu et al.^394^ reported a series of amino-functionalized Re-containing frameworks, comprising the Re^I^(CO)_3_(bpydc)(Cl) metallo-linker and the amino-containing bpdc-(NH_2_)_2_ linker within a **UiO-67** scaffold. The system was investigated for the light-driven conversion of CO_2_ to CO, using TEA as an electron donor. Of the systems prepared, the one in which about 30% of the linkers contained the amino substituents, **Re-MOF-NH**_**2**_**(33%)**, showed the highest activity for photocatalytic CO_2_RR, with a 3-fold increase in comparison with **Re-MOF** without any amino groups. Supported by DFT calculations, the authors proposed a number of cooperative effects between the Re-based metallo-linker and adjacent amino groups to explain this improved performance (Figure 40a). At the start of the catalytic cycle (Figure 40b), the amino-group can reversibly react with CO_2_ to form a carbamate, where the OH of the carboxylic group forms a weak hydrogen bond with the chloride of the Re complex. Following light absorption and reductive quenching by the TEA donor, the Re center attacks the electrophilic carbonyl carbon to form the metallo-carboxylate along with the regeneration of the amino group. It is notable that the Re center at this stage is seven-coordinate with the chloride being H-bound to the amino-group. Upon a second light absorption/reductive quenching cycle, the metal-bound COOH is transformed to CO and water as a byproduct.

Mao et al.^395^ reported the synthesis of a **UiO-66**-derived electrocatalyst into which FeTCPPCl ((5,10,15,20-tetrakis(4-carboxyphenyl)porphyrinato)-Fe^III^ chloride) linkers had been incorporated. The resulting **FeTCPP⊂UiO-66** consists of Zr-oxo clusters interconnected by BDC and FeTCPPCl and was demonstrated to catalyze the electrochemical CO_2_RR in an aqueous solution. The **UiO-66** framework provides protons at the Zr-oxo clusters that act to counterbalance negative charge that emerges at the porphyrin-bound carboxylates during electrochemical CO_2_ reduction, thereby enhancing the CO_2_ reduction activity of the iron porphyrin linkers. The authors considered a concerted proton-coupled electron transfer mechanism, which contributed to the high FE_CO_ of ∼100% at a low overpotential.

**Figure 41:**
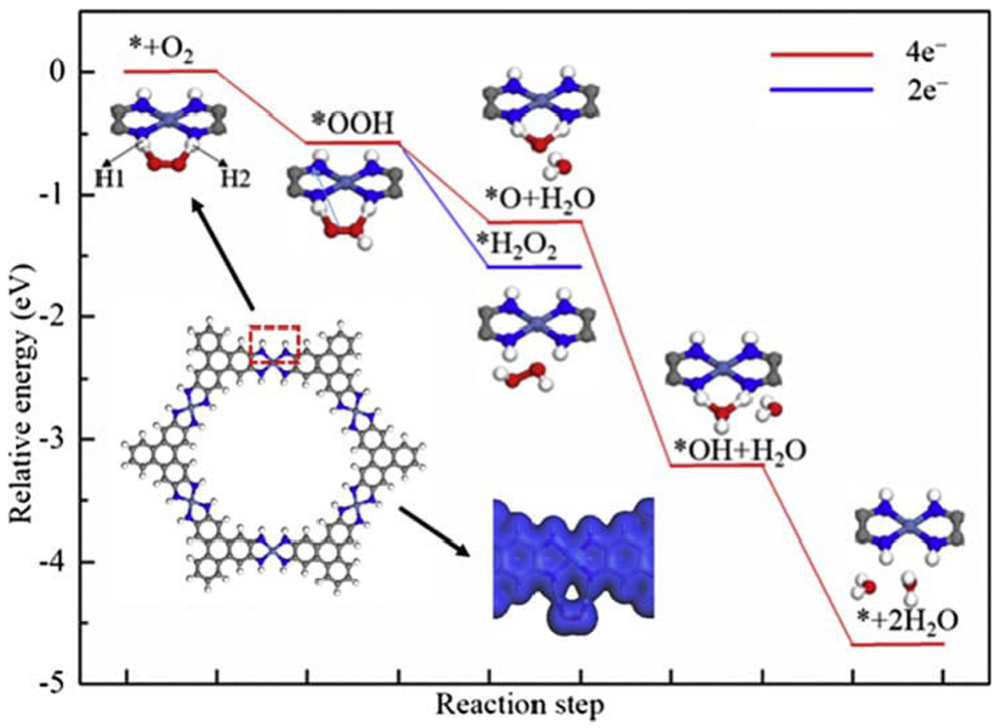
Relative energy diagram for the ORR pathway where oxygen is adsorbed in a bridged configuration between the two protons at the nitrogen centers of HITP. Reprinted with permission from reference (396). Copyright 2017 Elsevier.

In 2016, Dincǎ and co-workers^118^ reported a 2D MOF, namely, **Ni**_**3**_**(HITP)**_**2**_, with a high intrinsic conductivity of 40 S cm^–1^. Thin films of **Ni**_**3**_**(HITP)**_**2**_ on glassy carbon rotating disk electrodes were found to catalyze the electrochemical ORR in a 0.1 M KOH solution with decent overpotential, affording predominantly H_2_O_2_ (87.5%) in a two-electron pathway. Further systematic theoretical investigations by Sun and Chen^396^ dealt with the elucidation of the reaction mechanism of **Ni**_**3**_**(HITP)**_**2**_ and the origin of the high electrocatalytic ORR activity. In addition to the expected Ni–N catalytic site, it was found that the hydrogens at the nitrogen heteroatoms of HITP also serve as an active site for the ORR (Figure 41). This site showed a higher adsorption energy for O_2_ compared to the Ni–N site, while Mulliken population analysis revealed that the oxidation state of the HITP ligand can be altered during the ORR.

In 2019, Li et al.^397^ presented the solvothermal synthesis of two Co-based MOFs, [Co_2_(HAD)_2_(AD)_2_(BA)]·DMF·2H_2_O (**AD-MOF-1**; HAD = adenine and BA = butanedioic acid) and [Co_2_(HAD)_2_(AD)_2_(IA)_2_]·DMF (**AD-MOF-2**; IA = isobutyric acid) and investigated their activities for the light-driven reduction of CO_2_ to formic acid. Under illumination and in the presence of triisopropanolamine as electron donor, **AD-MOF-2** displayed a HCOOH production rate in aqueous media more than twice as high as that of **AD-MOF-1** in acetonitrile. Experimental and theoretical studies revealed that light-absorption is localized at the adenine, and following reductive quenching, also CO_2_RR catalysis occurs at this site, rather than at the Co centers. During the catalytic cycle, CO_2_ binds to the aromatic nitrogen atom of the adenine moieties, assisted by the amino group in *ortho*-position to the nucleophilic adenine-*N*.

#### 3.3.4 Discrete Cooperative Effects between SBU-Based Catalysts and Surrounding MOF Matrix

An interesting report describing how MOFs can not only stabilize unusual active sites but also alter their reactivity in the light-driven HER was reported for mixed-node **MOF-74(Fe,Co)**.^398^ The material consisted of Co/Fe mixed-metal SBUs that are interconnected with 2,5-dihydroxyterephthalic acid linkers. Using in situ X-ray absorption spectroscopy, it was possible to elucidate details of the catalytic mechanism, and the chemistry around the Co and Fe centers in **MOF-74(Fe,Co)**. Under standard catalytic conditions in the presence of [Ru(bpy)_3_]Cl_2_ and TEOA, it is shown that both Co and Fe centers in their reduced oxidation states are catalytically active sites. Interestingly, it is shown that the accumulation of these states is responsible for the induction period that is observed before continuous hydrogen evolution. Under steady-state HER conditions, the SBU features a Co center with an elongated Co–O bond distance, while the Fe center adopts a more distorted octahedral geometry compared to the situation in the as-synthesized geometry. According to the authors, it is this cooperative effect of the two metal centers that leads to these structural changes upon reduction, and that is responsible for the high HER activity of the mixed-metal **MOF-74(Fe,Co)**.

**Figure 42:**
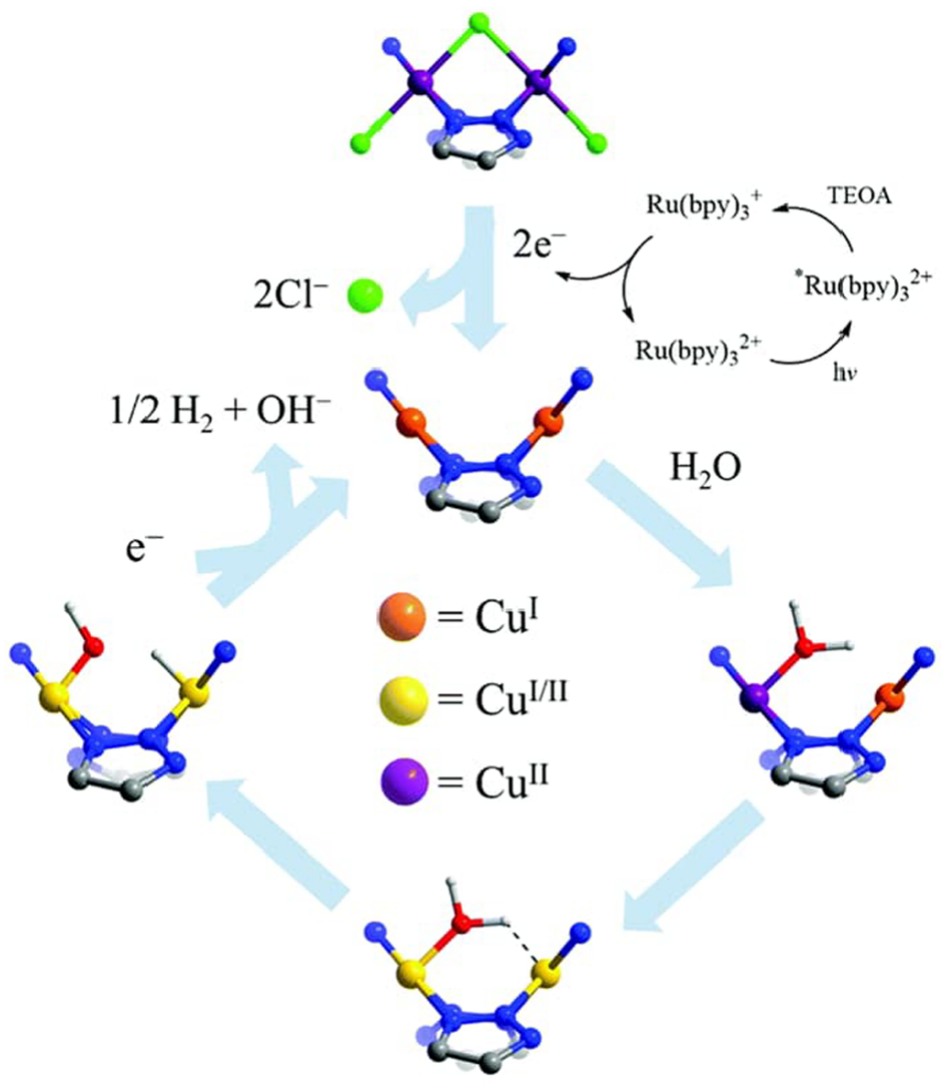
Cooperative effect between two Cu^I^ centers to active water under the formation of a Cu–H state. Reprinted with permission from reference (399). Copyright 2020 Royal Society of Chemistry.

Light-driven HER that is catalyzed by the cooperative action of two low-valent Cu ions that represent the SBU of a MOF has also been reported.^399^ Starting from the as-prepared [Cu_2_(μ-Cl)_2_(bbta)] MOF (**Cu**_**2**_**–Cl**_**2**_**-bbta** or **MAF-X29**, H_2_bbta = 1*H*,5*H*-benzo(1,2-*d*:4,5-*d*′)bistriazole) in which the Cu^II^ centers are bridged by charge balancing μ-chloride ions, light-induced reduction leads to decoordination of the chloride ligands, and the formation of the catalytically active dinuclear Cu^I^–Cu^I^ site to form **Cu**_**2**_**-bbta** (Figure 42). The ligand exchange that is triggered by Cu-reduction was followed spectroscopically, and **Cu**_**2**_**-bbta** is found to be a competent catalyst for the light-driven HER, using [Ru(bpy)_3_]Cl_2_ as a PS and TEOA as an electron donor. In accordance with DFT calculation on the catalytic mechanism, the high activity of **Cu**_**2**_**-bbta** is attributed to a cooperative effect in which the two Cu ions jointly activate water in a pathway with a comparably low-energy potential energy surface, ultimately leading to the Cu hydride intermediate. Further experimental proof for the cooperative effect is obtained from the reference material **Cu-btdd** (H_2_btdd = bis(1*H*-1,2,3-triazolo-[4,5-*b*],[4′,5′-*i*])dibenzo-[1,4]-dioxin) with a single Cu catalytic center that exhibits a significantly lower HER activity.

Another example of a MOF with SBUs consisting of a dinuclear Cu-site was reported as a photocatalytic HER material. In this report, **Cu-I-bpy**, a MOF consisting of discrete Cu_2_I_2_ clusters that are interconnected with 4,4′-bipyridine linkers acts as both a light absorber and a catalyst.^400^ Following light absorption, reductive quenching by the TEA electron donor led to the reduction of the Cu^I^ centers. The thereby produced Cu^0^ state reacts with protons to afford Cu^I^ hydride species that are crucial intermediates for HER. Interestingly, the distance between two Cu^I^ centers of the Cu_2_I_2_ cluster is appropriate for the interaction between two Cu^I^ hydrides, offering a pathway for facile hydrogen generation.

Sargent and co-workers^401^ demonstrated in 2018 that the product selectivity of **HKUST-1** as a catalyst material for the CO_2_RR can be modulated by controlling the coordination number and geometry of the constituting paddle-wheel Cu dimer SBU. The latter was distorted to an asymmetric motif by separating adjacent benzene tricarboxylate moieties using a thermal treatment, thereby altering the local atomic structure, oxidation state, and bonding strain of the Cu dimers. Using EPR and in situ X-ray absorption spectroscopy, Cu clusters with low coordination numbers were observed, giving rise to an unusually high FE of 45% for ethylene production.

**Figure 43:**
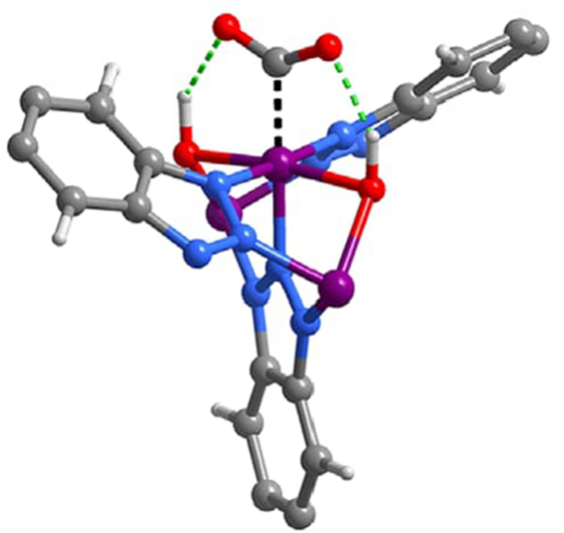
Perturbation DFT-derived CO_2_ binding structure for the reduced forms of **MAF-X27-OH**, showing the cooperative environment of the μ-OH^–^ ligands, stabilizing the Co–CO_2_ adducts and promoting the C–O bond dissociation. Adapted with permission from reference (402). Copyright 2018 American Chemical Society.

A cooperative effect at Co-based SBUs was identified by Wang et al.^402^ when investigating a series of cobalt-based MOFs (**MAF-X27-Cl**, **MAF-X27***l***-Cl**, **MAF-X27-OH**, **MAF-X27***l***-OH**, **MOF-74-Co**, and **Co-ZIF-9**) for their activity in the photocatalytic CO_2_RR in conjunction with [Ru(bpy)_3_]Cl_2_ and TEOA. The MOFs have in common that they contain a catalytically active Co site but differ in the surrounding coordination environment, most importantly in the presence or absence of μ-OH^–^ ligands at the *cis*-positions neighboring the Co centers. Under 1 atm CO_2_ and identical photocatalysis conditions, the MOFs with μ-OH^–^ ligands showed the best CO selectivity and TOF. In these MOFs, the TOFs are only marginally reduced when the CO_2_ partial pressure was decreased to 0.1 atm, clearly pointing to a cooperative role of μ-OH^–^ ligands. Indeed, computational results suggested that the μ-OH^–^ ligands acted as hydrogen-bonding donors to stabilize the Co–CO_2_ adducts and served as proton sources to promote the C–O bond dissociation (Figure 43).

In 2019, Lian et al.^403^ reported a study on **M**_**3**_**HITP**_**2**_ (M = Co or/and Ni) conductive coordination polymers with different Co/Ni ratios. The nonpreferred interaction of the HITP linker with Co^2+^, the reduced crystallinity, and the π–π layering of **Co**_**3**_**HITP**_**2**_ predicted a deformed quadrilateral configuration and a lack of coplanarity due to the unpaired electron in the 3dz^2^ orbital. This distortion limits charge transport through space, resulting in a significant reduction in electric conductivity. Despite the lower conductivity, **Co**_**3**_**HITP**_**2**_ displayed outstanding electrocatalytic ORR and OER activity, which can be attributed to the more active metal center with unpaired 3d electrons. Importantly, the ORR mechanism was observed to undergo a transition from the two-electron pathway on **Ni**_**3**_**HITP**_**2**_ to the four-electron pathway on **Co**_**3**_**HITP**_**2**_.

### 3.4 Contacting

While examples in the preceding sections have concentrated on effects within the MOF crystals, this section clearly takes a system perspective on catalytic efficacy and focuses on the importance of the MOF/electrode interface. Reports are reviewed in which this interface has been addressed specifically, and in many cases optimized for improved performance. The section bears certain analogies to protein film electrochemistry,^404,405^ and the use of enzymes for potential bioelectrocatalysis applications.^406^ In the latter contexts, it has been shown that the enzyme/substrate interfaces are of vital importance.^66^ For example, in case of 2D electrode substrates, it is important for electrocatalytic function that the enzyme is immobilized in a way that enables interfacial electron transfer at sufficiently high rates. This entails that enzymes need to reside on the electrode in a way that either the active site itself, or the most outer unit of an electron transport chain is in close spatial proximity to the electrode surface. Consequently, multiple strategies to “wire” enzymes to electrode surfaces have been developed.^407^ In particular, increasing the physical contact area between enzyme and electrode by nanostructured substrates have the potential to give rise to enhanced current densities.^408^ Further efforts to enlarge electrode surface areas, often based on carbon nanotubes, have been reported to increase enzyme loadings and current densities.^409^

Considering the above, it is not surprising that the performance of MOF-based electrocatalytic systems also exhibits a dependence on the immobilization and contacting methods. This was illustrated in a comparative study on a **UiO-66** sample into which model complexes of the [FeFe] H_2_ase active site had been incorporated. Due to the low abundance of the [FeFe] metallo-linker, the resulting **UiO-66-[FeFe]** was poorly conducting, and only close-to-surface [FeFe] sites could electrochemically be addressed.^410^ It was shown that 3D electrodes based on multiwalled CNTs inks best engulfed the MOF particles, giving rise to the highest current responses of the investigated substrate anchorage strategies.

#### 3.4.1 MOFs in 3D Electrodes

Carbon nanotubes (CNTs) have been used extensively together with catalytic MOFs as conducting components to produce inks that are then deposited on electrode surfaces for electrocatalysis experiments. In this context, Huang et al.^411^ reported a systematic study on the optimal ratio between catalytic MOF materials and contacting CNTs. In this contribution, an iron triazolate framework **FeTa**_**2**_ and its performance as composite with varying amounts of conductive Ketjenblack (KB) carbon for the ORR in alkaline electrolyte was investigated. Maximum current densities were observed in **FeTa**_**2**_**-KB** composites that contained 50 wt % of the **FeTa**_**2**_ catalyst. The authors hypothesized that this composition is the “sweet spot”, balancing maximum catalyst loading and conductivity, provided by **FeTa**_**2**_ and KB, respectively.

A similar observation was reported by Dong et al.^412^ in a study on the Fe porphyrin-based framework **PCN-222(Fe)**. Mixed in different ratios with carbon black and deposited onto carbon paper through a dip-coating method, the resulting **PCN-222(Fe)/C** composites were investigated for electrocatalytic CO_2_-to-CO reduction activity. The composites showed decreased activity the higher the catalyst content was (**PCN-222(Fe)**:**C** > 1:1), consistent with poor MOF crystallite contacting with low amounts of carbon black. Interestingly, also product selectivity depended on **PCN-222(Fe)/C** composition, with the materials with better **PCN-222(Fe)** contacting (**PCN-222(Fe)**:**C** < 1:1) showing less HER activity.

MOFs are, however, not only coimmobilized with CNTs but also grown directly onto them. In 2016, Fang et al.^413^ reported such a hybrid material when growing a cobalt-containing zeolitic imidazolate MOF on CNTs to produce **Co-MOF@CNTs**. The authors showed an efficient “wiring” of the **Co-MOF** through the CNTs, and that the addition of CNTs can improve the conductivity of the composite. Consequently, the **Co-MOF@CNTs** showed OER and ORR catalytic activity comparable to that of commercial RuO_2_ and 20 wt % Pt/C catalysts, respectively, while also exhibiting good stability.

Lin and co-workers^414^ described a **Hf**_**12**_**-CoDBP/CNT** composite by growing a MOF that consisted of a Co-porphyrin-type linker (DBP = 5,15-di(*p*-benzoato)-porphyrin) and a Hf_12_-based SBU on CNTs. It was shown that the covalent attachment of the MOF nanoplates to conductive CNTs improved interfacial electron transfer from the electrode to the Co-porphyrin active sites, which in turn are involved in diffusional electron hopping charge transport. The **Hf**_**12**_**-CoDBP/CNT** assembly electrocatalyzed the HER with a TON of 32 000 in 30 min, corresponding to an apparent TOF of 17.7 s^–1^.

Going beyond the “wiring” function, Ma et al.^415^ recently illustrated that different conducting substrates can alter product selectivity in the ORR reaction. For this purpose, **Co-MOF** comprising Al octahedral nodes connected by CoTCPP linkers were grown on CNTs and reduced graphene oxide (rGO). The two different carbon supports promote film growth of **Co-MOF** stacks with different surface orientations, which in turn caused differences in conductivity normal to the substrate surface. The **Co-MOF** nanoplates on rGO exhibit a lower overpotential for the ORR, but more importantly also a different product selectivity. By changing from the CNTs to the rGO support, the electrocatalytic mechanism of the ORR is also altered from the two-electron reduction to the four-electron reduction pathway.

#### 3.4.2 3D Electrodes from 2D Layered Architectures

For long-term durability of future devices, the structural stability of the electrode/MOF interface is of high importance. Already in 2014, Jiang et al.^416^ conducted a study on this topic by investigating different MOF composites consisting of catalytically active **NPC-4** (Cu_2_(TMBDI)(H_2_O)_2_) (TMBDI = 2,3,5,6-tetramethyl-benzene-1,4-di-isophthalate) MOF layers for the electrochemical ORR. When immobilized directly on GCEs, the application of **NPC-4** was limited due to its detachment from the electrode surface. To remedy this shortcoming, rGO was immobilized first onto the GCE surface prior to the MOF-immobilization. The extra rGO layer served as a binder and an electron transfer mediator, providing a large specific surface area and more contact points for the adhesion of the MOF than the bare GCE.

**Figure 44:**
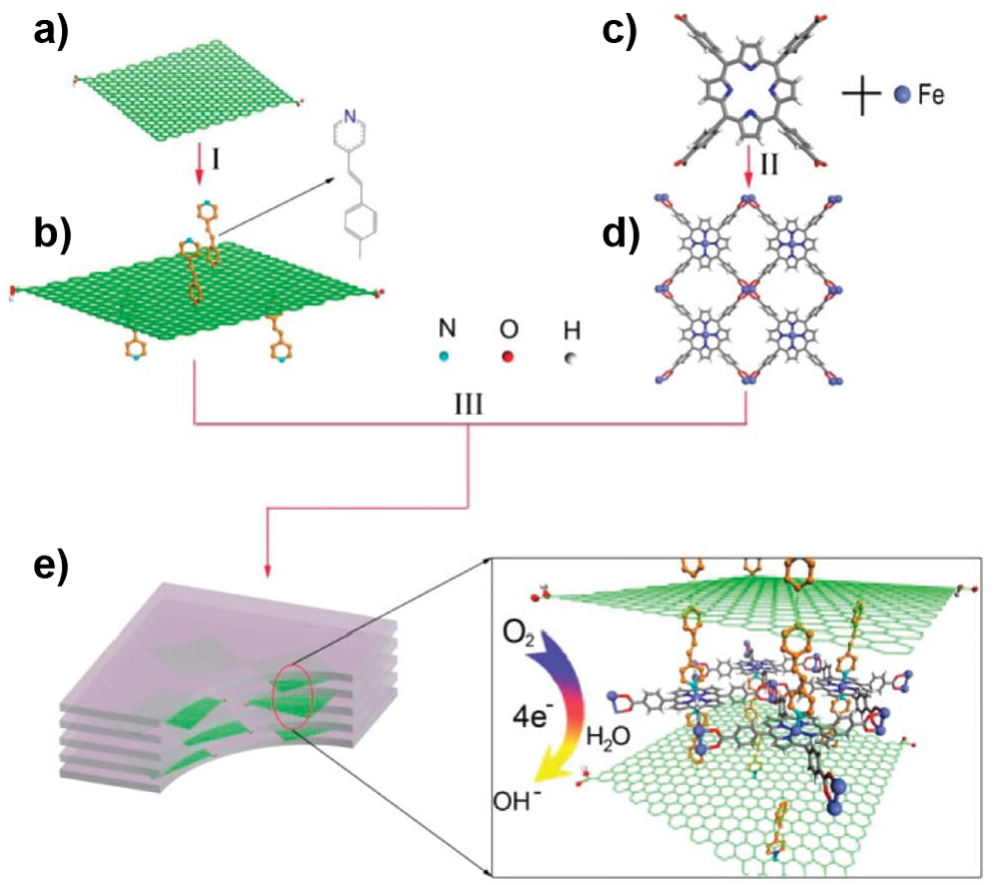
Schematic representation of a) reduced graphene oxide (rGO), b) G-dye, c) TCPP, d) **(Fe–P)**_***n***_ MOF, e) **(G-dye-FeP)**_***n***_ MOF, and f) magnified view of layers inside the framework of **(G-dye-FeP)**_***n***_ MOF showing how graphene sheets intercalated between porphyrin networks. Reprinted with permission from reference (417). Copyright 2012 American Chemical Society.

An alternative approach for efficient “MOF wiring” is the construction of 3D electrodes, which was already demonstrated in 2012 by Loh and co-workers.^417^ The authors reported the composite material **(G-dye-FeP)**_***n***_, consisting of pyridine-functionalized rGO nanosheets that were incorporated as a building block in varying ratios into an FeTCPP-based MOF during solvothermal synthesis (Figure 44). Incorporation of the pyridine-functionalized graphene into the MOF produced a composite graphene/MOF material with enhanced catalytic ORR activity. The structure and electrochemical property of the hybrid MOF were investigated as a function of the weight percentage of the functionalized graphene added to the iron porphyrin framework. The results showed that the addition of pyridine-functionalized graphene changes the crystallization process of the iron porphyrin MOF increases its porosity and enhances charge transport, resulting in facile four-electron ORR.

The following year, the same group reported a composite material consisting of graphene oxide (GO) and a copper-based MOF for the electrocatalytic HER, the OER, as well as the ORR. The MOF, abbreviated as **Cu-MOF**, consisted of paddle wheel Cu_2_(COO)_4_(ted)_2_ (ted = triethylenediamine) SBUs that are linked by BDC to form a 2D net parallel to the xy plane that is then further connected by ted to form the 3D **Cu-MOF**. GO sheets decorated with −OH and epoxy groups on either side of the sheets are analogous to the BDC pillar connectors and used in the solvothermal MOF preparation in amounts ranging from 2 to 10 wt %. The resulting GO-incorporated **Cu-MOF** composites exhibited smaller overpotentials and higher currents for all three electrocatalytic reactions and showed better stability in acidic media compared to that of the pure MOF. It was postulated that the enhanced electrocatalytic properties and stability in acid of the GO-MOF composite is due to the unique porous scaffold structure, improved charge transport, and synergistic interactions between the GO and the MOF.^418^

In 2016, Sohrabi et al.^419^ described the synthesis of the composite **PCN-222-G-py**, a combination of an iron porphyrin MOF and pyridine-functionalized graphene (G-py). The pyridine groups at the graphene substrates act as axial ligands to the Fe porphyrins the MOF is composed of, altering the electronic properties of the latter. **PCN-222-G-py** deposited on a GCE catalyzed the ORR in acidic media at a potential that was anodically shifted by 100 mV compared to that of pristine **PCN-222**. The pyridine functional groups connected the graphene sheets of the MOF facilitated interfacial electron transport and thus the electrocatalytic ORR.

#### 3.4.3 Unusual Electrolytes and Compartmentalization

Apart from the electrode/MOF interface, also the MOF/electrolyte interface can be designed for improved catalytic activity and device durability. One such paper addressed the role of ionic liquids (ILs) as replacements of traditional electrolyte solutions in polar organic solvents or water. ILs possess a wide electrochemical window and exhibit good conductivity. In addition, they are able to interact with CO_2_ by physical adsorption, thereby promoting the CO_2_ activation. This effect was utilized by Kang et al.^420^ who investigated the electrochemical reduction of CO_2_ to CH_4_ with a **Zn-BTC** MOFs that had electrophoretically been deposited on carbon paper (CP). Electrocatalysis was performed in different Bmim-based (Bmim = 1-butyl-3-methylimidazolium) ILs. Out of the investigated ILs (BmimBF_4_, BmimOTF, BmimPF_6_, and BmimClO_4_), the fluorine-containing ILs displayed the highest total current densities, owing to the interaction between fluorine with CO_2_. The combination of ILs and the **Zn-MOF/CP** cathode gave rise to the CO_2_-to-CH_4_ electroreduction with a FE exceeding 80% at a current density higher than 3 mA cm^–2^ and an overpotential of 0.25 V. Control experiment with 0.1 M BmimBF_4_ in acetonitrile showed much lower selectivity for CH_4_, demonstrating that ILs are responsible for the high selectivity of the CO_2_RR toward CH_4_.

**Figure 45:**
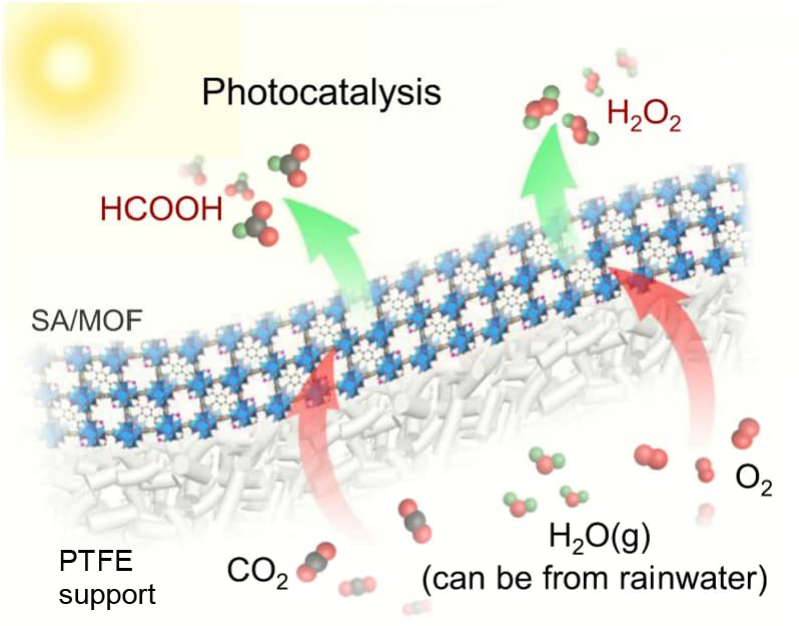
Humidified gases (e.g., CO_2_, O_2_) can be fed through the gas-permeable MOF/PTFE membranes and photocatalytically reduced to value-added chemicals (e.g., HCOOH and H_2_O_2_) under visible light irradiation and ambient conditions. Adapted with permission from reference (421). Copyright 2021 Springer Nature.

MOFs that catalyze the CO_2_RR and ORR in a light-driven process have also been organized as gas-permeable MOF membrane systems.^421^ The MOFs that were used for this study were based on highly defective **NH**_**2**_**-UiO-66** (**A-aUiO**) crystals into which Ir and Pd single-atom (SA) active sites had been coordinated to the Zr_6_O_4_(OH)_4_ cluster SBUs. These **SA/A-aUiO** (SA = Ir or Pd) crystallites were used to fabricate flexible and gas-permeable membranes via layer-by-layer deposition onto porous polytetrafluoroethylene (PTFE) films (Figure 45). The membranes can be run in gas-membrane-gas (GMG) mode in which humidified CO_2_ gas is fed through the membrane together with isopropanol vapor. The water and isopropanol in the CO_2_ stream were used as the proton source and sacrificial agent, respectively. Running the **Ir/A-aUiO** membrane in the GMG mode under illumination, CO_2_ is converted to HCOOH with a near-unity selectivity and an impressive apparent quantum efficiency of 15.76% at 420 nm. Control experiments with a more traditional gas–liquid–solid reaction mode resulted in significantly lower quantum efficiencies, proving the favorable effect of the GMG operational mode that promotes gas diffusion. Analogous observations have been made for the photocatalytic O_2_-to-H_2_O_2_ reduction, using membranes consisting of **Ir/A-aUiO** MOFs.

**Figure 46:**
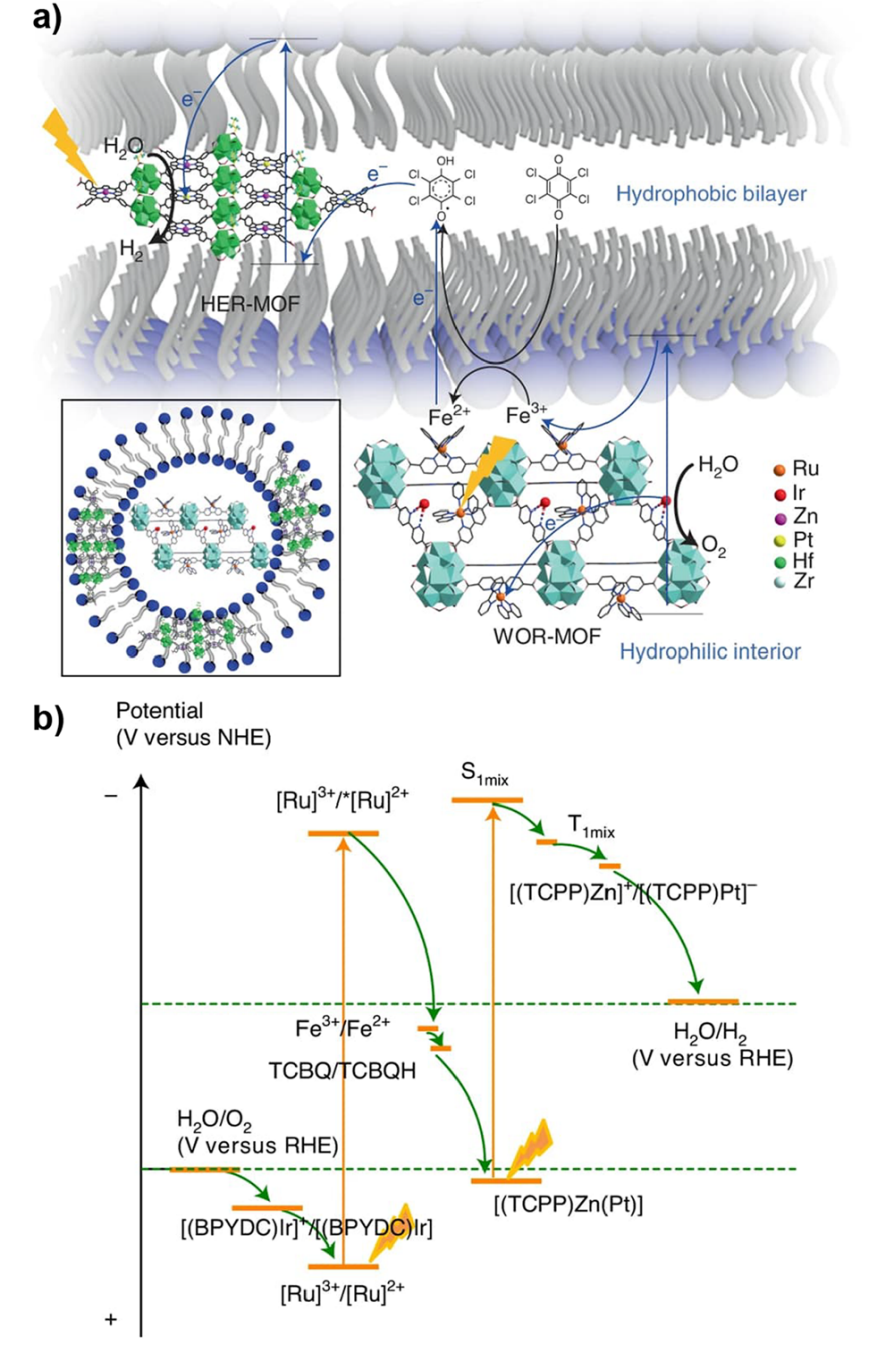
MOFs for photocatalytic HER and OER embedded in lipid bilayer for overall photocatalytic water splitting. a) Systematic representation of the constituting materials and b) energy level diagram of the reaction. Adapted with permission from reference (189). Copyright 2021 Springer Nature.

The final contribution in this section is possibly the most advanced organization of catalytic MOFs as components for a complete light-driven water splitting system.^189^ In fact, the compartmentalization of the half reactions and their communication promoted by proton and redox shuttles bears strong resemblance to the Z-scheme in natural photosynthesis.^422^ In this contribution, MOFs for the light-driven HER and OER have been embedded in the lipid bilayer and interior of a liposome, respectively, and were shown to promote water splitting into its constituting elements with an apparent quantum yield of 1.5 ± 1% without the need of any sacrificial agents (Figure 46a). The MOF nanosheets that were used for the light-driven HER half reaction consisted of light-harvesting Zn-porphyrin (ZnTCPP) and catalytic Pt-porphyrin (PtTCPP) linkers, and hexanuclear Hf-based SBUs. For facile incorporation into the hydrophobic lipid bilayer of the liposome, the constituting components of the HER-MOF were functionalized with hydrophobic groups. The MOF for OER consisted of [Ru(bpy)_3_]^2+^-based PSs and Ir-bpy catalyst linkers, and Zr-based SBUs, localized in the hydrophilic interior of the liposome. The two half reactions are electrically coupled by means of Fe^3+/2+^ tetrachlorobenzoquinone-based redox and proton mediators. Careful analyses of the MOF–liposome assembly by electrochemical and transient absorption techniques revealed the sequence of events that led to the overall water splitting process (Figure 46b). Photo-oxidation of the [Ru]^2+^ linker in the OER-MOF by Fe^3+^ followed by hole transfer to the catalytic Ir linker, generates high-valent Ir species that oxidize water to O_2_ with concomitant release of protons. The produced Fe^2+^ is reoxidized by TCBQ (tetrachlorobenzoquinone) at the lipid/water interface to afford TCBQH (tetrachlorobenzohydrosemiquinone) that is the reducing agent for the HER half reaction. On the cathodic side, photon absorption by the [ZnTCPP]/[PtTCPP] leads to a triplet state, which undergoes charge separation to generate [PtTCPP]^−^ and [ZnTCPP]^+^. The [ZnTCPP]^+^ oxidizes the TCBQH back to TCBQ and releases the proton; the [PtTCPP]^−^ is protonated to [H–PtTCPP], which accepts another electron and proton to release H_2_ and to complete the overall water splitting cycle. The use of two relays and their different hydrophobicity/hydrophilicity allows for more favorable concentration gradients compared to those in a homogeneous system, and is believed to be a key to the performance of this system. The authors identified potential for improvements by better balancing the intrinsic rates of the two photosystems, where currently the HER reaction was slower and allowed reduced forms of the relay to accumulate, which in turn increased recombination with the OER MOF components. This study is a beautiful example of organization on a microscopic and macroscopic level. The MOFs stabilized the light-harvesting and catalytic functional components as linkers in three dimensions, while the OER- and HER-MOF nanosheets themselves are designed to localize in the lipid bilayer or the interior of the liposome, respectively.

## 4 Conclusion

A number of distinct factors differentiate MOFs from other solid supports into which molecular catalysts may be incorporated. The modular nature of MOFs allows for a large variety of SBUs and linkers to be combined in unique topologies. Coordination compounds or metal clusters that may be unstable in a homogeneous solution phase may be constructed as discrete molecular entities as SBUs in MOFs. If such SBUs are catalytically active, as shown for many examples in section 3.1.2, unprecedented catalyst motifs can be designed, synthesized, and investigated. In addition, known catalysts can be incorporated into MOFs by a variety of strategies (sections 3.1.1–3.1.3), thereby boosting structural integrity under turnover conditions.

Furthermore, the arsenal of postsynthetic modification methods that can be employed on existing frameworks is unrivalled by any other incorporation matrix. It is this feature that triggers the imagination of chemists and provokes drawing parallels between MOFs and enzymes. If a MOF pore is regarded as a reaction vessel with linkers and SBUs (more or less) fixed in space, additional functionality can be introduced at specific sites by organic chemistry methods to tailor pore interiors. If these decorations interact favorably with a simultaneously present catalyst, the situation truly resembles second and third coordination sphere effects in enzymes. In the latter, it is a globally (more or less) rigid protein framework that provides defined points in space to which amino acid side chains are attached that interact with catalytic active sites.

The major difference between enzymatic and MOF-borne catalysis lies in the fact that enzymes contain *one* active site and a protein environment that is optimized to support its function. Under biological conditions, every enzyme, and thus every active site is supplied with electrons/holes and substrate from the surrounding environment to enable its fast and energy efficient operation. Reflecting the importance of transport in biological systems, nature has optimized the third (and fourth) coordination sphere to facilitate charge and mass transport to/from the active sites through carefully designed channels.

It is at this stage that the MOF/enzyme analogy does not hold any longer. In contrast to single entity enzymes with one active site, MOF crystals or thin films exhibit significantly higher concentrations of active sites, sometimes periodically reoccurring in three dimensions within distances as short as a nanometer. Catalyst concentrations in MOFs can approach 1 M, and the importance of transport phenomena cannot be overestimated in such a situation. Consequently, principles concerning charge and mass transport in MOFs are outlined in section 2.1 and 2.2. In an ideal case, high active site concentrations can give rise to significant current densities in electrocatalytic experiments, but only if every catalyst in the MOF is supplied with a sufficient flux of redox equivalents and substrates. In porous materials such as MOFs, this requirement may often not be met, and catalysts in the interior of the bulk MOF may lie dormant. Making things worse, the presence of transport limitations may often remain unnoticed, and transport as a phenomenon that limits efficiency is still underappreciated in the field. As outlined in section 2, it is highly advisable to vary as many parameters as possible (including light intensity, catalyst concentration and particle size), and to match these data to physical models as a basis for understanding kinetic bottlenecks in MOF/molecular catalyst systems.

**Figure 47:**
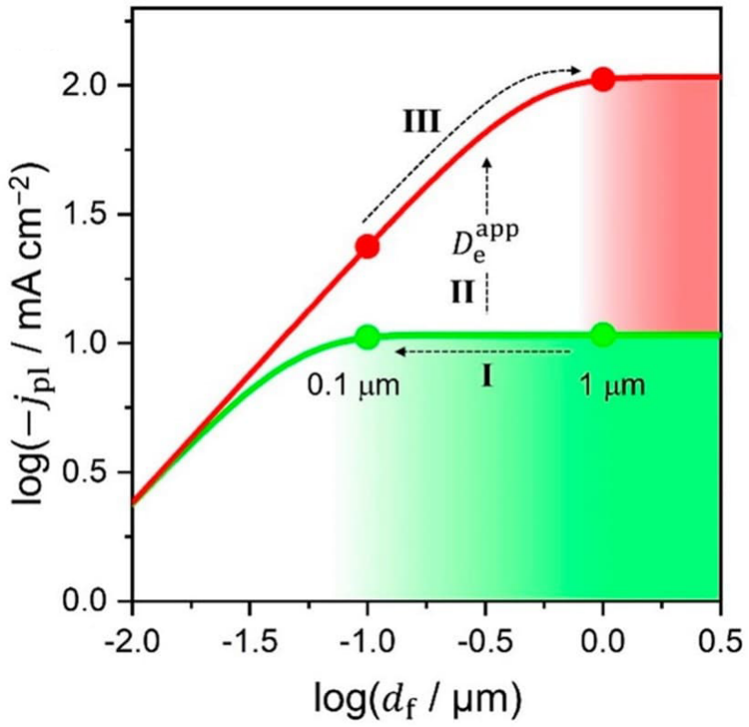
Options for optimizing MOF-based molecular catalysis either by adjustment of the film thickness or by increasing a diffusion coefficients. Plotted are current density versus the film thickness (*d*_f_), under the assumption of facile substrate transport and a reasonable intrinsic catalyst TOF. The shaded region below each curve signifies film thicknesses where the efficiency is less than unity and the amount of active catalyst is less than the amount of total catalyst. The curves are for *D*_e_^app^ = 5 × 10^–10^ cm^2^ s^–1^ (green curve) and 5 × 10^–8^ cm^2^ s^–1^ (red curve). Adapted with permission from reference (60). Copyright 2020 American Chemical Society.

That such scrutinous studies can be highly rewarding and yield significant enhancements in catalytic efficiency and current density was recently illustrated in a thought experiment on molecular electrocatalysis in MOFs by Johnson et al.^60^ (Figure 47). Under the assumption of facile substrate transport and a reasonable intrinsic catalyst turnover frequency, an apparent electron diffusion coefficient (*D*_e_^app^) of 5 × 10^–10^ cm^2^ s^–1^ was shown to sustain a maximum (plateau) current density of 10 mA cm^–2^ in a 0.1 μm thick MOF film. Utilizing the unique properties of MOFs to tailor specific properties, one could imagine that of *D*_e_^app^, may be increased to 5 × 10^–8^ cm^2^ s^–1^. As a result of the faster charge transport, the catalytic MOF film can be made thicker (1 μm) without compromising efficiency, reaching a maximum current density of 100 mA cm^–2^.

Transport limitations in photochemical schemes in which the light absorber and the catalyst are coimmobilized in close proximity and engage in a fast light-induced electron transfer process may at first glance be less of an issue. This view is, however, highly deceiving, as sacrificial agents that are the final oxidant/reductant need to diffuse into the MOF to provide the holes/electrons for the reaction at hand. As described in section 2.3, this process needs to be fast in order to compete with nonproductive pathways such as charge recombination. Even in a scenario when the oxidant/reductant is too large to diffuse into the MOF and provides holes/electrons to the MOF at the MOF/solution interface, charge transport between discrete redox active sites may limit the overall process. The vast majority of MOFs discussed in this review are not semiconducting, as is often assumed, but instead insulating materials. Therefore, charge transport proceeds through a hopping mechanism which is diffusional in nature. Moreover, it is coupled to the diffusion/migration of charge balancing counterions.

The present review clearly illustrates that the field has realized the effect of MOFs to stabilize structural integrity of a catalyst, which is very important. The inclusion of higher coordination sphere effects, and their successful experimental manifestation is, however, still in its infancy. Papers in which transport, which is third coordination sphere effects, is specifically investigated as discussed in section 3.2 are scarce in general and in context of catalysis even more so. An alternative strategy to lessen the impact of transport limitations is the use of nanometer-sized platelets and nanosheets, which however, comes at the cost of low current densities. With transport not being limiting, it is in such systems that synthetic modifications that lead to accelerated intrinsic catalyst rates are easiest demonstrated. Examples of such strategies and their experimental manifestation as described in section 3.3 are still scarce in the field. On the other hand, all original papers that were discussed herein originate from the past decade, and with the knowledge that has been accumulated, it is certain that MOFs as materials to host molecular catalysts will continue to evolve. For example, the manifestation of potential gradients for long-range electric fields is such an open quest. Such a system would be particularly important for full artificial photosynthesis, where both catalytic reduction and oxidation occurs by light-induced charge separation. Another interesting challenge would be to utilize and even control the structural dynamics of the MOFs,^423,424^ and couple such processes to transport and catalysis. Extrapolating the current knowledge increase in MOF-based catalysis into the future, our mechanistic understanding will continue to grow, and its translation into exciting new catalysis systems can be expected. One such example on the rational design of MOF-based artificial enzymes was reported very recently by Lin and co-workers.^425^ The study was based on the integration of active sites for the CO_2_RR and OER as well as proximal amino acids and other cofactors into tunable MOF monolayers. The MOF layers were first separately optimized for the CO_2_RR and OER by a diversification, selection and optimization strategy, and then coupled into a complete artificial photosynthesis scheme by the addition of a Co(bpy)_3_^2+^ redox mediator. The optimized CO_2_RR MOF featured pendant urea groups in the second coordination sphere of the Hemin active site, resulting in highly active and selective photocatalytic CO_2_ reduction with a 27-fold increase in activity over the homogeneous control. The most active photocatalytic OER system contained pendant (*p*-chloro-phenyl)amide groups in the second coordination sphere of an Ir-based molecular catalyst. The coupling of the two optimized MOFs with the redox mediator resulted in a complete artificial photosynthesis system that catalyzed light-driven CO_2_RR and OER, according to (1 + *n*)CO_2_ + 2H_2_O → CH_4_ + *n*CO + (2 + *n*/2)O_2_. The turnover frequency of the process was close to 100 h^–1^, thus over 1 order of magnitude faster than previously reported photocatalysts. This paper is a glimpse into what the future in MOF-based catalysis with orchestrated higher coordination sphere effects may hold.

## Abbreviations

The following list of abbreviations contains commonly known abbreviations as well as abbreviations often used throughout this review article. Further abbreviations were used in certain sections, which are then directly introduced and used throughout smaller sections and are therefore not listed here.

For naming conventions of MOFs and certain linkers, we tried to make it as consistent as possible throughout this review. This includes that we changed some “set” names from certain publications to fit into this consistency. For example, the adjustment whenever 2,2′-bipyridine-5,5′-dicarboxylate was mentioned to bpydc, even though the original publication wrote dcbpy or a deviation from it.

1D	one-dimensional
2D	two-dimensional
Adt	three-dimensional
ALD	azadithiolate
ATA	2-aminoterephthalate
H_2_ATA	2-aminoterephthalic acid
BDC	benzene-1,4-dicarboxylate
H_2_BDC	benzene-1,4-dicarboxylic acid
bpdc	biphenyl-4,4′-dicarboxylate
H_2_bpdc	biphenyl-4,4′-dicarboxylic acid
bpy	2,2′-bipyridine
bpydc	2,2′-bipyridine-5,5′-dicarboxylate
H_2_bpydc	2,2′-bipyridine-5,5′-dicarboxylic acid
BTB	1,3,5-benzene(tris)benzoate
H_3_BTB	1,3,5-benzene(tris)benzoic acid
BTC	1,3,5-benzenetricarboxylate
H_3_BTC	1,3,5-benzenetricarboxylic acid
CAN	carbon nanotubes
Cat	catalyst (in relation with figures or equations)
CNTs	carbon nanotubes
CO_2_RR	carbon dioxide reduction reaction
Cp	cyclopentadienyl
Cp*	η^5^-C_5_Me_5_/1,2,3,4,5-pentamethylcyclopentadiene
D	electron donor (in relation with figures or equations)
dcbdt	1,4-dicarboxylbenzene-2,3-dithiolato
DFT	density functional theory
DMF	dimethylformamide
EPR	electron paramagnetic resonance
FE	Faradaic efficiency
FTO	fluorine-doped tin oxide
GCE	glassy carbon electrode
GO	graphene oxide
H_2_ase	hydrogenase
H_2_TCPP	4,4′,4″,4‴-(porphyrin-5,10,15,20-tetrayl)tetrabenzoate
HER	hydrogen evolution reaction
HHTP	2,3,6,7,10,11-hexahydroxytriphenylene
HITP	2,3,6,7,10,11-hexaaminotriphenylene
MIL	Matériaux de l′Institut Lavoisier
MOF	metal–organic framework
MOL	metal–organic layer
OER	oxygen evolution reaction
ORR	oxygen reduction reaction
PCN	porous coordination network
POM	polyoxometalate
PS	photosensitizer
rGO	reduced graphene oxide
RHE	reversible hydrogen electrode
SBU	secondary building unit
TEA	triethylamine
TEOA	triethanolamine
terpy	2,2′:6′,2″-terpyridine
TOF	turnover frequency
TON	turnover number
UiO	Universitetet i Oslo
WOC	water oxidation catalyst
XPS	X-ray photoelectron spectroscopy
XRD	X-ray diffraction
ZIF	zeolitic imidazolate framework

### List of Mathematical Symbols in Section 2

The following list summarizes all mathematical symbols used throughout the formulas in section 2 and their respective meaning.

ϕ	Thiele modulus
Da	Damköhler number
*L*	Damköhler number
*k*_cat_	first-order rate constant of the catalytic reaction
	(in s^–1^)
*D*_*s*_	intraparticle diffusion coefficient of the substrate
*v*	observed reaction rate (in mol s^–1^)
η	internal effectiveness factor
TOF^app^	apparent TOF; rate normalized to the total
	amount of catalyst present
TOF^true^	only active catalyst considered in catalytic particle
*n*_total_	total moles of catalyst within the MOF particle
*n*_active_	active moles of catalyst within the MOF particle
*V*	volume of the particle
*C*_cat_	concentration of the catalyst
*r*	radial distance in spherical coordinates
*R*	radius of the particle
*D*_*ox*_	diffusivity of the sacrificial oxidant
*C*_*ox*_^0^	bulk oxidant concentration
*C*_*cat*_^0^	total catalyst concentration
*C*_S_^0^	substrate concentration
*D*	electron hopping coefficient OR diffusion
	coefficient of the substrate
*d*_*f*_	film thickness
*d*_*f*_^*opt*^	optimal film thickness
Φ_*H*_2__	HER quantum yield
*n*_*H*_2__	number of moles of H_2_ produced
*n*_*photon*_	number of moles of photons absorbed
*Rate*_*hv*_	rate of photon absorption

## References

[ref1] Montoya J. H.; Seitz L. C.; Chakthranont P.; Vojvodic A.; Jaramillo T. F.; Norskov J. K. Materials for Solar Fuels and Chemicals. Nat. Mater. 2017, 16, 70–81. 10.1038/nmat4778. 27994241

[ref2] Appel A. M.; Bercaw J. E.; Bocarsly A. B.; Dobbek H.; DuBois D. L.; Dupuis M.; Ferry J. G.; Fujita E.; Hille R.; Kenis P. J.; et al. Frontiers, Opportunities, and Challenges in Biochemical and Chemical Catalysis of CO_2_ Fixation. Chem. Rev. 2013, 113, 6621–6658. 10.1021/cr300463y. 23767781 PMC3895110

[ref3] Lewis N. S.; Nocera D. G. Powering the Planet: Chemical Challenges in Solar Energy Utilization. Proc. Natl. Acad. Sci. U. S. A. 2006, 103, 15729–15735. 10.1073/pnas.0603395103. 17043226 PMC1635072

[ref4] Benson E. E.; Kubiak C. P.; Sathrum A. J.; Smieja J. M. Electrocatalytic and Homogeneous Approaches to Conversion of CO_2_ to Liquid Fuels. Chem. Soc. Rev. 2009, 38, 89–99. 10.1039/B804323J. 19088968

[ref5] Li X.; Yu J.; Jaroniec M.; Chen X. Cocatalysts for Selective Photoreduction of CO_2_ into Solar Fuels. Chem. Rev. 2019, 119, 3962–4179. 10.1021/acs.chemrev.8b00400. 30763077

[ref6] Jones A. K.; Sillery E.; Albracht S. P.; Armstrong F. A. Direct Comparison of the Electrocatalytic Oxidation of Hydrogen by an Enzyme and a Platinum Catalyst. Chem. Commun. 2002, 866–867. 10.1039/b201337a. 12123018

[ref7] Sun L.; Reddu V.; Fisher A. C.; Wang X. Electrocatalytic Reduction of Carbon Dioxide: Opportunities with Heterogeneous Molecular Catalysts. Energy Environ. Sci. 2020, 13, 374–403. 10.1039/C9EE03660A.

[ref8] Fang Y.; Powell J. A.; Li E.; Wang Q.; Perry Z.; Kirchon A.; Yang X.; Xiao Z.; Zhu C.; Zhang L.; et al. Catalytic Reactions within the Cavity of Coordination Cages. Chem. Soc. Rev. 2019, 48, 4707–4730. 10.1039/C9CS00091G. 31339148

[ref9] Tan C.; Chu D.; Tang X.; Liu Y.; Xuan W.; Cui Y. Supramolecular Coordination Cages for Asymmetric Catalysis. Chem. Eur. J. 2019, 25, 662–672. 10.1002/chem.201802817. 30076749

[ref10] Corma A.; Garcia H. Supramolecular Host-Guest Systems in Zeolites Prepared by Ship-in-a-Bottle Synthesis. Eur. J. Inorg. Chem. 2004, 2004, 1143–1164. 10.1002/ejic.200300831.

[ref11] Kosinov N.; Liu C.; Hensen E. J. M.; Pidko E. A. Engineering of Transition Metal Catalysts Confined in Zeolites. Chem. Mater. 2018, 30, 3177–3198. 10.1021/acs.chemmater.8b01311. 29861546 PMC5973782

[ref12] Dai C.; Zhang A.; Song C.; Guo X. Advances in the Synthesis and Catalysis of Solid and Hollow Zeolite-Encapsulated Metal Catalysts. Adv. Catal. 2018, 63, 75–115. 10.1016/bs.acat.2018.10.002.

[ref13] Murray R. W. Introduction to Molecularly Designed Electrode Surfaces. In Molecular Design of Electrode Surfaces, 1st ed.; John Wiley & Sons, Inc.: New York, 1992; pp 1–48.

[ref14] Gascon J.; Corma A.; Kapteijn F.; Llabrés i Xamena F. X. Metal Organic Framework Catalysis: Quo Vadis?. ACS Catal. 2014, 4, 361–378. 10.1021/cs400959k.

[ref15] Xie L. S.; Skorupskii G.; Dincǎ M. Electrically Conductive Metal-Organic Frameworks. Chem. Rev. 2020, 120, 8536–8580. 10.1021/acs.chemrev.9b00766. 32275412 PMC7453401

[ref16] Kalaj M.; Cohen S. M. Postsynthetic Modification: An Enabling Technology for the Advancement of Metal-Organic Frameworks. ACS Cent. Sci. 2020, 6, 1046–1057. 10.1021/acscentsci.0c00690. 32724840 PMC7379093

[ref17] Rayder T. M.; Bensalah A. T.; Li B.; Byers J. A.; Tsung C. K. Engineering Second Sphere Interactions in a Host-Guest Multicomponent Catalyst System for the Hydrogenation of Carbon Dioxide to Methanol. J. Am. Chem. Soc. 2021, 143, 1630–1640. 10.1021/jacs.0c08957. 33464883

[ref18] Rakowski DuBois M.; DuBois D. L. Development of Molecular Electrocatalysts for CO_2_ Reduction and H_2_ Production/Oxidation. Acc. Chem. Res. 2009, 42, 1974–1982. 10.1021/ar900110c. 19645445

[ref19] Ball P. Water as an Active Constituent in Cell Biology. Chem. Rev. 2008, 108, 74–108. 10.1021/cr068037a. 18095715

[ref20] Rebilly J. N.; Colasson B.; Bistri O.; Over D.; Reinaud O. Biomimetic Cavity-Based Metal Complexes. Chem. Soc. Rev. 2015, 44, 467–489. 10.1039/C4CS00211C. 25319612

[ref21] Hussein R.; Ibrahim M.; Bhowmick A.; Simon P. S.; Chatterjee R.; Lassalle L.; Doyle M.; Bogacz I.; Kim I. S.; Cheah M. H.; et al. Structural Dynamics in the Water and Proton Channels of Photosystem II During the S_2_ to S_3_ Transition. Nat. Commun. 2021, 12, 6531 10.1038/s41467-021-26781-z. 34764256 PMC8585918

[ref22] Jeuken L. J.; Jones A. K.; Chapman S. K.; Cecchini G.; Armstrong F. A. Electron-Transfer Mechanisms through Biological Redox Chains in Multicenter Enzymes. J. Am. Chem. Soc. 2002, 124, 5702–5713. 10.1021/ja012638w. 12010043

[ref23] Siegbahn P. E.; Tye J. W.; Hall M. B. Computational Studies of [NiFe] and [FeFe] Hydrogenases. Chem. Rev. 2007, 107, 4414–4435. 10.1021/cr050185y. 17927160

[ref24] Silakov A.; Wenk B.; Reijerse E.; Lubitz W. ^14^NHYSCORE Investigation of the H-Cluster of [FeFe] Hydrogenase: Evidence for a Nitrogen in the Dithiol Bridge. Phys. Chem. Chem. Phys. 2009, 11, 6592–6599. 10.1039/b905841a. 19639134

[ref25] Erdem Ö. F.; Schwartz L.; Stein M.; Silakov A.; Kaur-Ghumaan S.; Huang P.; Ott S.; Reijerse E. J.; Lubitz W. A Model of the [FeFe] Hydrogenase Active Site with a Biologically Relevant Azadithiolate Bridge: A Spectroscopic and Theoretical Investigation. Angew. Chem., Int. Ed. 2011, 50, 1439–1443. 10.1002/anie.201006244. 21290530

[ref26] Berggren G.; Adamska A.; Lambertz C.; Simmons T. R.; Esselborn J.; Atta M.; Gambarelli S.; Mouesca J. M.; Reijerse E.; Lubitz W.; et al. Biomimetic Assembly and Activation of [FeFe]-Hydrogenases. Nature 2013, 499, 66–69. 10.1038/nature12239. 23803769 PMC3793303

[ref27] Lorenzi M.; Berggren G. [FeFe] Hydrogenases and Their Functional Models. In Comprehensive Coordination Chemistry III, 3rd ed.; Elsevier Ltd.: Amsterdam, 2021; pp 731–756.

[ref28] Ho M.-H.; Rousseau R.; Roberts J. A. S.; Wiedner E. S.; Dupuis M.; DuBois D. L.; Bullock R. M.; Raugei S. Ab Initio-Based Kinetic Modeling for the Design of Molecular Catalysts: The Case of H_2_ Production Electrocatalysts. ACS Catal. 2015, 5, 5436–5452. 10.1021/acscatal.5b01152.

[ref29] Helm M. L.; Stewart M. P.; Bullock R. M.; DuBois M. R.; DuBois D. L. A Synthetic Nickel Electrocatalyst with a Turnover Frequency above 100,000 S^–1^ for H_2_ Production. Science 2011, 333, 863–866. 10.1126/science.1205864. 21836012

[ref30] Anxolabéhère-Mallart E.; Costentin C.; Fournier M.; Nowak S.; Robert M.; Savéant J. M. Boron-Capped Tris(Glyoximato) Cobalt Clathrochelate as a Precursor for the Electrodeposition of Nanoparticles Catalyzing H_2_ Evolution in Water. J. Am. Chem. Soc. 2012, 134, 6104–6107. 10.1021/ja301134e. 22458714

[ref31] Nagoshi K.; Yamashita S.; Yagi M.; Kaneko M. Catalytic Activity of [(bpy)_2_(H_2_O)Ru-O-Ru(H_2_O)(bpy)_2_]^4+^ for Four-Electron Water Oxidation. J. Mol. Catal. A: Chem. 1999, 144, 71–76. 10.1016/S1381-1169(98)00334-3.

[ref32] Lampret O.; Duan J.; Hofmann E.; Winkler M.; Armstrong F. A.; Happe T. The Roles of Long-Range Proton-Coupled Electron Transfer in the Directionality and Efficiency of [FeFe]-Hydrogenases. Proc. Natl. Acad. Sci. U. S. A. 2020, 117, 20520–20529. 10.1073/pnas.2007090117. 32796105 PMC7456106

[ref33] Duan J.; Mebs S.; Laun K.; Wittkamp F.; Heberle J.; Happe T.; Hofmann E.; Apfel U.-P.; Winkler M.; Senger M.; et al. Geometry of the Catalytic Active Site in [FeFe]-Hydrogenase Is Determined by Hydrogen Bonding and Proton Transfer. ACS Catal. 2019, 9, 9140–9149. 10.1021/acscatal.9b02203.

[ref34] Finkelmann A. R.; Stiebritz M. T.; Reiher M. Inaccessibility of the Μ-Hydride Species in [FeFe] Hydrogenases. Chem. Sci. 2014, 5, 215–221. 10.1039/C3SC51700D.

[ref35] Li H.; Rauchfuss T. B. Iron Carbonyl Sulfides, Formaldehyde, and Amines Condense to Give the Proposed Azadithiolate Cofactor of the Fe-Only Hydrogenases. J. Am. Chem. Soc. 2002, 124, 726–727. 10.1021/ja016964n. 11817928

[ref36] Esselborn J.; Lambertz C.; Adamska-Venkatesh A.; Simmons T.; Berggren G.; Noth J.; Siebel J.; Hemschemeier A.; Artero V.; Reijerse E.; et al. Spontaneous Activation of [FeFe]-Hydrogenases by an Inorganic [2Fe] Active Site Mimic. Nat. Chem. Biol. 2013, 9, 607–609. 10.1038/nchembio.1311. 23934246 PMC3795299

[ref37] Lubitz W.; Ogata H.; Rudiger O.; Reijerse E. Hydrogenases. Chem. Rev. 2014, 114, 4081–4148. 10.1021/cr4005814. 24655035

[ref38] Artz J. H.; Mulder D. W.; Ratzloff M. W.; Lubner C. E.; Zadvornyy O. A.; LeVan A. X.; Williams S. G.; Adams M. W. W.; Jones A. K.; King P. W.; et al. Reduction Potentials of [FeFe]-Hydrogenase Accessory Iron-Sulfur Clusters Provide Insights into the Energetics of Proton Reduction Catalysis. J. Am. Chem. Soc. 2017, 139, 9544–9550. 10.1021/jacs.7b02099. 28635269

[ref39] Raleiras P.; Khanna N.; Miranda H.; Mészáros L. S.; Krassen H.; Ho F.; Battchikova N.; Aro E.-M.; Magnuson A.; Lindblad P.; et al. Turning around the Electron Flow in an Uptake Hydrogenase. EPR Spectroscopy and in Vivo Activity of a Designed Mutant in HupSL from Nostoc Punctiforme. Energy Environ. Sci. 2016, 9, 581–594. 10.1039/C5EE02694F.

[ref40] O’Keeffe M.; Yaghi O. M. Deconstructing the Crystal Structures of Metal-Organic Frameworks and Related Materials into Their Underlying Nets. Chem. Rev. 2012, 112, 675–702. 10.1021/cr200205j. 21916513

[ref41] Batten S. R.; Champness N. R.; Chen X.-M.; Garcia-Martinez J.; Kitagawa S.; Öhrström L.; O’Keeffe M.; Paik Suh M.; Reedijk J. Terminology of Metal-Organic Frameworks and Coordination Polymers (IUPAC Recommendations 2013). Pure Appl. Chem. 2013, 85, 1715–1724. 10.1351/PAC-REC-12-11-20.

[ref42] Betard A.; Fischer R. A. Metal-Organic Framework Thin Films: From Fundamentals to Applications. Chem. Rev. 2012, 112, 1055–1083. 10.1021/cr200167v. 21928861

[ref43] Xu C.; Fang R.; Luque R.; Chen L.; Li Y. Functional Metal-Organic Frameworks for Catalytic Applications. Coord. Chem. Rev. 2019, 388, 268–292. 10.1016/j.ccr.2019.03.005.

[ref44] Majewski M. B.; Peters A. W.; Wasielewski M. R.; Hupp J. T.; Farha O. K. Metal-Organic Frameworks as Platform Materials for Solar Fuels Catalysis. ACS Energy Lett. 2018, 3, 598–611. 10.1021/acsenergylett.8b00010.

[ref45] Qin J. S.; Yuan S.; Lollar C.; Pang J.; Alsalme A.; Zhou H. C. Stable Metal-Organic Frameworks as a Host Platform for Catalysis and Biomimetics. Chem. Commun. 2018, 54, 4231–4249. 10.1039/C7CC09173G. 29637210

[ref46] Honicke I. M.; Senkovska I.; Bon V.; Baburin I. A.; Bonisch N.; Raschke S.; Evans J. D.; Kaskel S. Balancing Mechanical Stability and Ultrahigh Porosity in Crystalline Framework Materials. Angew. Chem., Int. Ed. 2018, 57, 13780–13783. 10.1002/anie.201808240. 30160076

[ref47] Li H.; Wang K.; Sun Y.; Lollar C. T.; Li J.; Zhou H.-C. Recent Advances in Gas Storage and Separation Using Metal-Organic Frameworks. Mater. Today 2018, 21, 108–121. 10.1016/j.mattod.2017.07.006.

[ref48] Getman R. B.; Bae Y. S.; Wilmer C. E.; Snurr R. Q. Review and Analysis of Molecular Simulations of Methane, Hydrogen, and Acetylene Storage in Metal-Organic Frameworks. Chem. Rev. 2012, 112, 703–723. 10.1021/cr200217c. 22188435

[ref49] Suh M. P.; Park H. J.; Prasad T. K.; Lim D. W. Hydrogen Storage in Metal-Organic Frameworks. Chem. Rev. 2012, 112, 782–835. 10.1021/cr200274s. 22191516

[ref50] Kreno L. E.; Leong K.; Farha O. K.; Allendorf M.; Van Duyne R. P.; Hupp J. T. Metal-Organic Framework Materials as Chemical Sensors. Chem. Rev. 2012, 112, 1105–1125. 10.1021/cr200324t. 22070233

[ref51] Burtch N. C.; Jasuja H.; Walton K. S. Water Stability and Adsorption in Metal-Organic Frameworks. Chem. Rev. 2014, 114, 10575–10612. 10.1021/cr5002589. 25264821

[ref52] McCarthy B. D.; Beiler A. M.; Johnson B. A.; Liseev T.; Castner A. T.; Ott S. Analysis of Electrocatalytic Metal-Organic Frameworks. Coord. Chem. Rev. 2020, 406, 213137 10.1016/j.ccr.2019.213137. 32499663 PMC7272229

[ref53] Rungtaweevoranit B.; Diercks C. S.; Kalmutzki M. J.; Yaghi O. M. Spiers Memorial Lecture:. Progress and Prospects of Reticular Chemistry. Faraday Discuss. 2017, 201, 9–45. 10.1039/C7FD00160F. 28820210

[ref54] Wang X.; Lan P. C.; Ma S. Metal-Organic Frameworks for Enzyme Immobilization: Beyond Host Matrix Materials. ACS Cent. Sci. 2020, 6, 1497–1506. 10.1021/acscentsci.0c00687. 32999925 PMC7517118

[ref55] Cohen S. M. The Postsynthetic Renaissance in Porous Solids. J. Am. Chem. Soc. 2017, 139, 2855–2863. 10.1021/jacs.6b11259. 28118009

[ref56] Cohen S. M. Postsynthetic Methods for the Functionalization of Metal-Organic Frameworks. Chem. Rev. 2012, 112, 970–1000. 10.1021/cr200179u. 21916418

[ref57] Deria P.; Mondloch J. E.; Karagiaridi O.; Bury W.; Hupp J. T.; Farha O. K. Beyond Post-Synthesis Modification: Evolution of Metal-Organic Frameworks via Building Block Replacement. Chem. Soc. Rev. 2014, 43, 5896–912. 10.1039/C4CS00067F. 24723093

[ref58] Mondloch J. E.; Katz M. J.; Isley W. C. III; Ghosh P.; Liao P.; Bury W.; Wagner G. W.; Hall M. G.; DeCoste J. B.; Peterson G. W.; et al. Destruction of Chemical Warfare Agents Using Metal-Organic Frameworks. Nat. Mater. 2015, 14, 512–516. 10.1038/nmat4238. 25774952

[ref59] Yadav A.; Kanoo P. Metal-Organic Frameworks as Platform for Lewis-Acid-Catalyzed Organic Transformations. Chem. Asian J. 2019, 14, 3531–3551. 10.1002/asia.201900876. 31509343

[ref60] Johnson B. A.; Beiler A. M.; McCarthy B. D.; Ott S. Transport Phenomena: Challenges and Opportunities for Molecular Catalysis in Metal-Organic Frameworks. J. Am. Chem. Soc. 2020, 142, 11941–11956. 10.1021/jacs.0c02899. 32516534 PMC7366383

[ref61] Sharp C. H.; Bukowski B. C.; Li H.; Johnson E. M.; Ilic S.; Morris A. J.; Gersappe D.; Snurr R. Q.; Morris J. R. Nanoconfinement and Mass Transport in Metal-Organic Frameworks. Chem. Soc. Rev. 2021, 50, 11530–11558. 10.1039/D1CS00558H. 34661217

[ref62] Zhao M.; Huang Y.; Peng Y.; Huang Z.; Ma Q.; Zhang H. Two-Dimensional Metal-Organic Framework Nanosheets: Synthesis and Applications. Chem. Soc. Rev. 2018, 47, 6267–6295. 10.1039/C8CS00268A. 29971309

[ref63] Huang Y. B.; Liang J.; Wang X. S.; Cao R. Multifunctional Metal-Organic Framework Catalysts: Synergistic Catalysis and Tandem Reactions. Chem. Soc. Rev. 2017, 46, 126–157. 10.1039/C6CS00250A. 27841411

[ref64] Leger C.; Bertrand P. Direct Electrochemistry of Redox Enzymes as a Tool for Mechanistic Studies. Chem. Rev. 2008, 108, 2379–438. 10.1021/cr0680742. 18620368

[ref65] Noll T.; Noll G. Strategies for ″Wiring″ Redox-Active Proteins to Electrodes and Applications in Biosensors, Biofuel Cells, and Nanotechnology. Chem. Soc. Rev. 2011, 40, 3564–3576. 10.1039/c1cs15030h. 21509355

[ref66] Xiao Y.; Patolsky F.; Katz E.; Hainfeld J. F.; Willner I. ″es″: Nanowiring of Redox Enzymes by a Gold Nanoparticle. Science 2003, 299, 1877–1881. 10.1126/science.1080664. 12649477

[ref67] Wang W.; Xu X.; Zhou W.; Shao Z. Recent Progress in Metal-Organic Frameworks for Applications in Electrocatalytic and Photocatalytic Water Splitting. Adv. Sci. 2017, 4, 1600371 10.1002/advs.201600371. PMC5396165 28435777

[ref68] Wang Q.; Astruc D. State of the Art and Prospects in Metal-Organic Framework (MOF)-Based and MOF-Derived Nanocatalysis. Chem. Rev. 2020, 120, 1438–1511. 10.1021/acs.chemrev.9b00223. 31246430

[ref69] Fang Y.; Ma Y.; Zheng M.; Yang P.; Asiri A. M.; Wang X. Metal-Organic Frameworks for Solar Energy Conversion by Photoredox Catalysis. Coord. Chem. Rev. 2018, 373, 83–115. 10.1016/j.ccr.2017.09.013.

[ref70] Banerjee S.; Anayah R. I.; Gerke C. S.; Thoi V. S. From Molecules to Porous Materials: Integrating Discrete Electrocatalytic Active Sites into Extended Frameworks. ACS Cent. Sci. 2020, 6, 1671–1684. 10.1021/acscentsci.0c01088. 33145407 PMC7596858

[ref71] Tu T. N.; Nguyen M. V.; Nguyen H. L.; Yuliarto B.; Cordova K. E.; Demir S. Designing bipyridine-Functionalized Zirconium Metal-Organic Frameworks as a Platform for Clean Energy and Other Emerging Applications. Coord. Chem. Rev. 2018, 364, 33–50. 10.1016/j.ccr.2018.03.014.

[ref72] Dhakshinamoorthy A.; Li Z.; Garcia H. Catalysis and Photocatalysis by Metal Organic Frameworks. Chem. Soc. Rev. 2018, 47, 8134–8172. 10.1039/C8CS00256H. 30003212

[ref73] Cohen S. M.; Zhang Z.; Boissonnault J. A. Toward ″metalloMOFzymes″: Metal-Organic Frameworks with Single-Site Metal Catalysts for Small-Molecule Transformations. Inorg. Chem. 2016, 55, 7281–7290. 10.1021/acs.inorgchem.6b00828. 27231968

[ref74] Chen K.; Wu C.-D. Designed Fabrication of Biomimetic Metal-Organic Frameworks for Catalytic Applications. Coord. Chem. Rev. 2019, 378, 445–465. 10.1016/j.ccr.2018.01.016.

[ref75] Nath I.; Chakraborty J.; Verpoort F. Metal Organic Frameworks Mimicking Natural Enzymes: A Structural and Functional Analogy. Chem. Soc. Rev. 2016, 45, 4127–4170. 10.1039/C6CS00047A. 27251115

[ref76] Ma L.; Jiang F.; Fan X.; Wang L.; He C.; Zhou M.; Li S.; Luo H.; Cheng C.; Qiu L. Metal-Organic-Framework-Engineered Enzyme-Mimetic Catalysts. Adv. Mater. 2020, 32, e2003065 10.1002/adma.202003065. 33124725

[ref77] Zhao M.; Ou S.; Wu C. D. Porous Metal-Organic Frameworks for Heterogeneous Biomimetic Catalysis. Acc. Chem. Res. 2014, 47, 1199–1207. 10.1021/ar400265x. 24499017

[ref78] Dhakshinamoorthy A.; Asiri A. M.; Garcia H. Metal-Organic Frameworks as Catalysts for Oxidation Reactions. Chem. Eur. J. 2016, 22, 8012–8024. 10.1002/chem.201505141. 27113486

[ref79] Mukhopadhyay S.; Basu O.; Nasani R.; Das S. K. Evolution of Metal Organic Frameworks as Electrocatalysts for Water Oxidation. Chem. Commun. 2020, 56, 11735–11748. 10.1039/D0CC03659E. 32940258

[ref80] Shao Q.; Yang J.; Huang X. The Design of Water Oxidation Electrocatalysts from Nanoscale Metal-Organic Frameworks. Chem. Eur. J. 2018, 24, 15143–15155. 10.1002/chem.201801572. 29687926

[ref81] Du J.; Li F.; Sun L. Metal-Organic Frameworks and Their Derivatives as Electrocatalysts for the Oxygen Evolution Reaction. Chem. Soc. Rev. 2021, 50, 2663–2695. 10.1039/D0CS01191F. 33400745

[ref82] Zhu B.; Zou R.; Xu Q. Metal-Organic Framework Based Catalysts for Hydrogen Evolution. Adv. Energy Mater. 2018, 8, 1801193 10.1002/aenm.201801193.

[ref83] Reddy D. A.; Kim Y.; Gopannagari M.; Kumar D. P.; Kim T. K. Recent Advances in Metal-Organic Framework-Based Photocatalysts for Hydrogen Production. Sustainable Energy Fuels 2021, 5, 1597–1618. 10.1039/C9SE00749K.

[ref84] Budnikova Y. H. Recent Advances in Metal-Organic Frameworks for Electrocatalytic Hydrogen Evolution and Overall Water Splitting Reactions. Dalton Trans. 2020, 49, 12483–12502. 10.1039/D0DT01741H. 32756705

[ref85] Alkhatib I. I.; Garlisi C.; Pagliaro M.; Al-Ali K.; Palmisano G. Metal-Organic Frameworks for Photocatalytic CO_2_ Reduction under Visible Radiation: A Review of Strategies and Applications. Catal. Today 2020, 340, 209–224. 10.1016/j.cattod.2018.09.032.

[ref86] Chen Y.; Wang D.; Deng X.; Li Z. Metal-Organic Frameworks (MOFs) for Photocatalytic CO_2_ Reduction. Catal. Sci. Technol. 2017, 7, 4893–4904. 10.1039/C7CY01653K.

[ref87] Li X.; Zhu Q.-L. MOF-Based Materials for Photo- and Electrocatalytic CO_2_ Reduction. EnergyChem. 2020, 2, 100033 10.1016/j.enchem.2020.100033.

[ref88] Lu X. F.; Xia B. Y.; Zang S. Q.; Lou X. W. D. Metal-Organic Frameworks Based Electrocatalysts for the Oxygen Reduction Reaction. Angew. Chem., Int. Ed. 2020, 59, 4634–4650. 10.1002/anie.201910309. 31529577

[ref89] Chen L.; Luque R.; Li Y. Controllable Design of Tunable Nanostructures inside Metal-Organic Frameworks. Chem. Soc. Rev. 2017, 46, 4614–4630. 10.1039/C6CS00537C. 28516998

[ref90] Ifraemov R.; Mukhopadhyay S.; Rozenberg I.; Hod I. Metal-Organic-Framework-Based Photo-Electrochemical Cells for Solar Fuel Generation. J. Phys. Chem. C 2022, 126, 5079–5091. 10.1021/acs.jpcc.2c00671.

[ref91] Du Y.; Jia X.; Zhong L.; Jiao Y.; Zhang Z.; Wang Z.; Feng Y.; Bilal M.; Cui J.; Jia S. Metal-Organic Frameworks with Different Dimensionalities: An Ideal Host Platform for Enzyme@MOF Composites. Coord. Chem. Rev. 2022, 454, 214327 10.1016/j.ccr.2021.214327.

[ref92] Wang C.; Liao K. Recent Advances in Emerging Metal- and Covalent-Organic Frameworks for Enzyme Encapsulation. ACS Appl. Mater. Interfaces 2021, 13, 56752–56776. 10.1021/acsami.1c13408. 34809426

[ref93] Dhakshinamoorthy A.; Asiri A. M.; Garcia H. Integration of Metal Organic Frameworks with Enzymes as Multifunctional Solids for Cascade Catalysis. Dalton Trans. 2020, 49, 11059–11072. 10.1039/D0DT02045A. 32808625

[ref94] Costentin C.; Savéant J.-M. Elements of Molecular and Biomolecular Electrochemistry: An Electrochemical Approach to Electron Transfer Chemistry, 2nd ed.; John Wiley & Sons, Inc.: Hoboken, 2019; pp 285–379.

[ref95] Costentin C.; Robert M.; Savéant J.-M. Molecular Catalysis of Electrochemical Reactions. Curr. Opin. Electrochem. 2017, 2, 26–31. 10.1016/j.coelec.2017.02.006.

[ref96] Costentin C.; Savéant J.-M. Homogeneous Molecular Catalysis of Electrochemical Reactions: Manipulating Intrinsic and Operational Factors for Catalyst Improvement. J. Am. Chem. Soc. 2018, 140, 16669–16675. 10.1021/jacs.8b09154. 30392356

[ref97] Costentin C. Molecular Catalysis of Electrochemical Reactions. Overpotential and Turnover Frequency: Unidirectional and Bidirectional Systems. ACS Catal. 2021, 11, 5678–5687. 10.1021/acscatal.1c00744.

[ref98] Johnson B. A.; Ott S. Diagnosing Surface Versus Bulk Reactivity for Molecular Catalysis within Metal-Organic Frameworks Using a Quantitative Kinetic Model. Chem. Sci. 2020, 11, 7468–7478. 10.1039/D0SC02601H. 33209240 PMC7116375

[ref99] Damköhler G. Einflüsse der Strömung, Diffusion und des Wärmeüberganges auf die Leistung von Reaktionsöfen.: I. Allgemeine Gesichtspunkte für die Übertragung eines Chemischen Prozesses aus dem Kleinen ins Große. Z. Elektrochem. Angew. Phys. Chem. 1936, 42, 846–862.

[ref100] Thiele E. W. Relation between Catalytic Activity and Size of Particle. Ind. Eng. Chem. 1939, 31, 916–920. 10.1021/ie50355a027.

[ref101] Wang R.; Bukowski B. C.; Duan J.; Sheridan T. R.; Atilgan A.; Zhang K.; Snurr R. Q.; Hupp J. T. Investigating the Process and Mechanism of Molecular Transport within a Representative Solvent-Filled Metal-Organic Framework. Langmuir 2020, 36, 10853–10859. 10.1021/acs.langmuir.0c01999. 32841562

[ref102] Kärger J.; Binder T.; Chmelik C.; Hibbe F.; Krautscheid H.; Krishna R.; Weitkamp J. Microimaging of Transient Guest Profiles to Monitor Mass Transfer in Nanoporous Materials. Nat. Mater. 2014, 13, 333–343. 10.1038/nmat3917. 24651427

[ref103] Titze T.; Lauerer A.; Heinke L.; Chmelik C.; Zimmermann N. E. R.; Keil F. J.; Ruthven D. M.; Kärger J. Transport in Nanoporous Materials Including MOFs: The Applicability of Fick’s Laws. Angew. Chem., Int. Ed. 2015, 54, 14580–14583. 10.1002/anie.201506954. 26448501

[ref104] Han S.; Hermans T. M.; Fuller P. E.; Wei Y.; Grzybowski B. A. Transport into Metal-Organic Frameworks from Solution Is Not Purely Diffusive. Angew. Chem., Int. Ed. 2012, 51, 2662–2666. 10.1002/anie.201108492. 22298442

[ref105] Leddy J.; Bard A. J.; Maloy J. T.; Savéant J. M. Kinetics of Film-Coated Electrodes: Effect of a Finite Mass Transfer Rate of Substrate across the Film—Solution Interface at Steady State. J. Electroanal. Chem. Interfacial Electrochem. 1985, 187, 205–227. 10.1016/0368-1874(85)85779-8.

[ref106] Heinke L.; Gu Z.; Woll C. The Surface Barrier Phenomenon at the Loading of Metal-Organic Frameworks. Nat. Commun. 2014, 5, 4562 10.1038/ncomms5562. 25078573

[ref107] Feng X.; Song Y.; Lin W. Dimensional Reduction of Lewis Acidic Metal-Organic Frameworks for Multicomponent Reactions. J. Am. Chem. Soc. 2021, 143, 8184–8192. 10.1021/jacs.1c03561. 34018731

[ref108] Guo Y.; Shi W.; Yang H.; He Q.; Zeng Z.; Ye J. Y.; He X.; Huang R.; Wang C.; Lin W. Cooperative Stabilization of the [Pyridinium-CO_2_-Co] Adduct on a Metal-Organic Layer Enhances Electrocatalytic CO_2_ Reduction. J. Am. Chem. Soc. 2019, 141, 17875–17883. 10.1021/jacs.9b09227. 31603671

[ref109] Guo Y.; Wang Y.; Shen Y.; Cai Z.; Li Z.; Liu J.; Chen J.; Xiao C.; Liu H.; Lin W.; et al. Tunable Cobalt-Polypyridyl Catalysts Supported on Metal-Organic Layers for Electrochemical CO_2_ Reduction at Low Overpotentials. J. Am. Chem. Soc. 2020, 142, 21493–21501. 10.1021/jacs.0c10719. 33319555

[ref110] Koros R. M.; Nowak E. J. A Diagnostic Test of the Kinetic Regime in a Packed Bed Reactor. Chem. Eng. Sci. 1967, 22, 470 10.1016/0009-2509(67)80134-9.

[ref111] Madon R. J.; Boudart M. Experimental Criterion for the Absence of Artifacts in the Measurement of Rates of Heterogeneous Catalytic Reactions. Ind. Eng. Chem. Fundam. 1982, 21, 438–447. 10.1021/i100008a022.

[ref112] Semrau A. L.; Stanley P. M.; Urstoeger A.; Schuster M.; Cokoja M.; Fischer R. A. Substantial Turnover Frequency Enhancement of MOF Catalysts by Crystallite Downsizing Combined with Surface Anchoring. ACS Catal. 2020, 10, 3203–3211. 10.1021/acscatal.0c00550.

[ref113] Stanley P. M.; Parkulab M.; Rieger B.; Warnan J.; Fischer R. A. Understanding Entrapped Molecular Photosystem and Metal-Organic Framework Synergy for Improved Solar Fuel Production. Faraday Discuss. 2021, 231, 281–297. 10.1039/D1FD00009H. 34240093

[ref114] Wang C.; Wang J. L.; Lin W. Elucidating Molecular Iridium Water Oxidation Catalysts Using Metal-Organic Frameworks: A Comprehensive Structural, Catalytic, Spectroscopic, and Kinetic Study. J. Am. Chem. Soc. 2012, 134, 19895–19908. 10.1021/ja310074j. 23136923

[ref115] Dou J.-H.; Sun L.; Ge Y.; Li W.; Hendon C. H.; Li J.; Gul S.; Yano J.; Stach E. A.; Dincǎ M. Signature of Metallic Behavior in the Metal-Organic Frameworks M_3_(hexaiminobenzene)_2_ (M = Ni, Cu). J. Am. Chem. Soc. 2017, 139, 13608–13611. 10.1021/jacs.7b07234. 28910095

[ref116] Clough A. J.; Orchanian N. M.; Skelton J. M.; Neer A. J.; Howard S. A.; Downes C. A.; Piper L. F. J.; Walsh A.; Melot B. C.; Marinescu S. C. Room Temperature Metallic Conductivity in a Metal-Organic Framework Induced by Oxidation. J. Am. Chem. Soc. 2019, 141, 16323–16330. 10.1021/jacs.9b06898. 31553621

[ref117] Sun L.; Campbell M. G.; Dincǎ M. Electrically Conductive Porous Metal-Organic Frameworks. Angew. Chem., Int. Ed. 2016, 55, 3566–3579. 10.1002/anie.201506219. 26749063

[ref118] Miner E. M.; Fukushima T.; Sheberla D.; Sun L.; Surendranath Y.; Dincǎ M. Electrochemical Oxygen Reduction Catalysed by Ni_3_(hexaiminotriphenylene)_2_. Nat. Commun. 2016, 7, 10942 10.1038/ncomms10942. 26952523 PMC4786780

[ref119] Stassen I.; Burtch N.; Talin A.; Falcaro P.; Allendorf M.; Ameloot R. An Updated Roadmap for the Integration of Metal-Organic Frameworks with Electronic Devices and Chemical Sensors. Chem. Soc. Rev. 2017, 46, 3185–3241. 10.1039/C7CS00122C. 28452388

[ref120] Costentin C.; Drouet S.; Robert M.; Savéant J.-M. Turnover Numbers, Turnover Frequencies, and Overpotential in Molecular Catalysis of Electrochemical Reactions. Cyclic Voltammetry and Preparative-Scale Electrolysis. J. Am. Chem. Soc. 2012, 134, 11235–11242. 10.1021/ja303560c. 22670885

[ref121] Costentin C.; Savéant J.-M. Cyclic Voltammetry of Fast Conducting Electrocatalytic Films. Phys. Chem. Chem. Phys. 2015, 17, 19350–19359. 10.1039/C5CP02825F. 26140372

[ref122] Costentin C.; Savéant J.-M. Cyclic Voltammetry of Electrocatalytic Films: Fast Catalysis Regimes. ChemElectroChem. 2015, 2, 1774–1784. 10.1002/celc.201500217.

[ref123] Costentin C.; Savéant J.-M. Cyclic Voltammetry Analysis of Electrocatalytic Films. J. Phys. Chem. C 2015, 119, 12174–12182. 10.1021/acs.jpcc.5b02376. 26140372

[ref124] Bard A. J. Inner-Sphere Heterogeneous Electrode Reactions. Electrocatalysis and Photocatalysis: The Challenge. J. Am. Chem. Soc. 2010, 132, 7559–7567. 10.1021/ja101578m. 20469860

[ref125] Limaye A. M.; Zeng J. S.; Willard A. P.; Manthiram K. Bayesian Data Analysis Reveals No Preference for Cardinal Tafel Slopes in CO_2_ Reduction Electrocatalysis. Nat. Commun. 2021, 12, 703 10.1038/s41467-021-20924-y. 33514735 PMC7846806

[ref126] Shinagawa T.; Garcia-Esparza A. T.; Takanabe K. Insight on Tafel Slopes from a Microkinetic Analysis of Aqueous Electrocatalysis for Energy Conversion. Sci. Rep. 2015, 5, 13801 10.1038/srep13801. 26348156 PMC4642571

[ref127] Hansen J. N.; Prats H.; Toudahl K. K.; Mørch Secher N.; Chan K.; Kibsgaard J.; Chorkendorff I. Is There Anything Better Than Pt for HER?. ACS Energy Lett. 2021, 6, 1175–1180. 10.1021/acsenergylett.1c00246. 34056107 PMC8155388

[ref128] Mariano R. G.; Wahab O. J.; Rabinowitz J. A.; Oppenheim J.; Chen T.; Unwin P. R.; Dincǎ M. Thousand-Fold Increase in O_2_ Electroreduction Rates with Conductive MOFs. ACS Cent. Sci. 2022, 8, 975–982. 10.1021/acscentsci.2c00509. 35912352 PMC9336150

[ref129] Gray H. B.; Winkler J. R. Electron Tunneling through Proteins. Q. Rev. Biophys. 2003, 36, 341–372. 10.1017/S0033583503003913. 15029828

[ref130] Page C. C.; Moser C. C.; Chen X.; Dutton P. L. Natural Engineering Principles of Electron Tunnelling in Biological Oxidation-Reduction. Nature 1999, 402, 47–52. 10.1038/46972. 10573417

[ref131] Brodsky C. N.; Bediako D. K.; Shi C.; Keane T. P.; Costentin C.; Billinge S. J. L.; Nocera D. G. Proton-Electron Conductivity in Thin Films of a Cobalt-Oxygen Evolving Catalyst. ACS Appl. Energy Mater. 2019, 2, 3–12. 10.1021/acsaem.8b00785.

[ref132] Costentin C.; Nocera D. G. Dual-Phase Molecular-Like Charge Transport in Nanoporous Transition Metal Oxides. J. Phys. Chem. C 2019, 123, 1966–1973. 10.1021/acs.jpcc.8b10948.

[ref133] Andrieux C. P.; Savéant J. M. Catalysis at Redox Polymer Coated Electrodes; Murray R. W., Ed.; John Wiley & Sons, Inc,: Hoboken, 1992; pp 207–270.

[ref134] Costentin C.; Savéant J.-M. Molecular Approach to Catalysis of Electrochemical Reaction in Porous Films. Curr. Opin. Electrochem. 2019, 15, 58–65. 10.1016/j.coelec.2019.03.014.

[ref135] Savéant J.-M. Molecular Catalysis of Electrochemical Reactions. Mechanistic Aspects. Chem. Rev. 2008, 108, 2348–2378. 10.1021/cr068079z. 18620367

[ref136] Kozuch S.; Martin J. M. L. Turning over” Definitions in Catalytic Cycles. ACS Catal. 2012, 2, 2787–2794. 10.1021/cs3005264.

[ref137] Tafel J. Über die Polarisation bei Kathodischer Wasserstoffentwicklung. Z. Phys. Chem. 1905, 50U, 641–712. 10.1515/zpch-1905-5043.

[ref138] Batchelor-McAuley C.; Katelhon E.; Barnes E. O.; Compton R. G.; Laborda E.; Molina A. Recent Advances in Voltammetry. ChemistryOpen 2015, 4, 224–260. 10.1002/open.201500042. 26246984 PMC4522172

[ref139] Colburn A. W.; Levey K. J.; O’Hare D.; Macpherson J. V. Lifting the Lid on the Potentiostat: A Beginner’s Guide to Understanding Electrochemical Circuitry and Practical Operation. Phys. Chem. Chem. Phys. 2021, 23, 8100–8117. 10.1039/D1CP00661D. 33875985

[ref140] Chang M. C.; Yee C. S.; Stubbe J.; Nocera D. G. Turning on Ribonucleotide Reductase by Light-Initiated Amino Acid Radical Generation. Proc. Natl. Acad. Sci. U. S. A. 2004, 101, 6882–6887. 10.1073/pnas.0401718101. 15123822 PMC406436

[ref141] Reisner E.; Powell D. J.; Cavazza C.; Fontecilla-Camps J. C.; Armstrong F. A. Visible Light-Driven H_2_ Production by Hydrogenases Attached to Dye-Sensitized TiO_2_ Nanoparticles. J. Am. Chem. Soc. 2009, 131, 18457–18466. 10.1021/ja907923r. 19928857

[ref142] Lubner C. E.; Knorzer P.; Silva P. J.; Vincent K. A.; Happe T.; Bryant D. A.; Golbeck J. H. Wiring an [FeFe]-Hydrogenase with Photosystem I for Light-Induced Hydrogen Production. Biochemistry 2010, 49, 10264–10266. 10.1021/bi1016167. 21058656

[ref143] Osterloh F. E. Photocatalysis Versus Photosynthesis: A Sensitivity Analysis of Devices for Solar Energy Conversion and Chemical Transformations. ACS Energy Lett. 2017, 2, 445–453. 10.1021/acsenergylett.6b00665.

[ref144] Neshvad G.; Hoffman M. Z. Reductive Quenching of the Luminescent Excited State of Tris(2,2’-bipyrazine)ruthenium(2+) Ion in Aqueous Solution. J. Phys. Chem. 1989, 93, 2445–2452. 10.1021/j100343a044.

[ref145] Mateo D.; Santiago-Portillo A.; Albero J.; Navalon S.; Alvaro M.; Garcia H. Long-Term Photostability in Terephthalate Metal-Organic Frameworks. Angew. Chem., Int. Ed. 2019, 58, 17843–17848. 10.1002/anie.201911600. 31584744

[ref146] Costentin C.; Camara F.; Fortage J.; Collomb M.-N. Photoinduced Catalysis of Redox Reactions. Turnover Numbers, Turnover Frequency, and Limiting Processes: Kinetic Analysis and Application to Light-Driven Hydrogen Production. ACS Catal. 2022, 6246–6254. 10.1021/acscatal.2c01289.

[ref147] Lakadamyali F.; Kato M.; Reisner E. Colloidal Metal Oxide Particles Loaded with Synthetic Catalysts for Solar H_2_ Production. Faraday Discuss. 2012, 155, 191–205. 10.1039/C1FD00077B. 22470975

[ref148] Limburg B.; Bouwman E.; Bonnet S. Rate and Stability of Photocatalytic Water Oxidation Using [Ru(bpy)_3_]^2+^ as Photosensitizer. ACS Catal. 2016, 6, 5273–5284. 10.1021/acscatal.6b00107.

[ref149] Limburg B.; Wermink J.; van Nielen S. S.; Kortlever R.; Koper M. T. M.; Bouwman E.; Bonnet S. Kinetics of Photocatalytic Water Oxidation at Liposomes: Membrane Anchoring Stabilizes the Photosensitizer. ACS Catal. 2016, 6, 5968–5977. 10.1021/acscatal.6b00151.

[ref150] Fang Z. B.; Liu T. T.; Liu J.; Jin S.; Wu X. P.; Gong X. Q.; Wang K.; Yin Q.; Liu T. F.; Cao R.; et al. Boosting Interfacial Charge-Transfer Kinetics for Efficient Overall CO_2_ Photoreduction via Rational Design of Coordination Spheres on Metal-Organic Frameworks. J. Am. Chem. Soc. 2020, 142, 12515–12523. 10.1021/jacs.0c05530. 32564596

[ref151] Xu H. Q.; Hu J.; Wang D.; Li Z.; Zhang Q.; Luo Y.; Yu S. H.; Jiang H. L. Visible-Light Photoreduction of CO_2_ in a Metal-Organic Framework: Boosting Electron-Hole Separation via Electron Trap States. J. Am. Chem. Soc. 2015, 137, 13440–13443. 10.1021/jacs.5b08773. 26434687

[ref152] Wu L. Y.; Mu Y. F.; Guo X. X.; Zhang W.; Zhang Z. M.; Zhang M.; Lu T. B. Encapsulating Perovskite Quantum Dots in Iron-Based Metal-Organic Frameworks (MOFs) for Efficient Photocatalytic CO_2_ Reduction. Angew. Chem., Int. Ed. 2019, 58, 9491–9495. 10.1002/anie.201904537. 31066965

[ref153] Fang X.; Shang Q.; Wang Y.; Jiao L.; Yao T.; Li Y.; Zhang Q.; Luo Y.; Jiang H. L. Single Pt Atoms Confined into a Metal-Organic Framework for Efficient Photocatalysis. Adv. Mater. 2018, 30, 1705112 10.1002/adma.201705112. 29315871

[ref154] Wasielewski M. R. Photoinduced Electron Transfer in Supramolecular Systems for Artificial Photosynthesis. Chem. Rev. 1992, 92, 435–461. 10.1021/cr00011a005.

[ref155] Gust D.; Moore T. A.; Moore A. L.; Lee S. J.; Bittersmann E.; Luttrull D. K.; Rehms A. A.; Degraziano J. M.; Ma X. C.; Gao F.; et al. Efficient Multistep Photoinitiated Electron Transfer in a Molecular Pentad. Science 1990, 248, 199–201. 10.1126/science.248.4952.199. 17740135

[ref156] Imahori H.; Guldi D. M.; Tamaki K.; Yoshida Y.; Luo C.; Sakata Y.; Fukuzumi S. Charge Separation in a Novel Artificial Photosynthetic Reaction Center Lives 380 ms. J. Am. Chem. Soc. 2001, 123, 6617–6628. 10.1021/ja004123v. 11439049

[ref157] Lin S.; Cairnie D. R.; Davis D.; Chakraborty A.; Cai M.; Morris A. J. Photoelectrochemical Alcohol Oxidation by Mixed-Linker Metal-Organic Frameworks. Faraday Discuss. 2021, 225, 371–383. 10.1039/D0FD00021C. 33107542

[ref158] Santiago-Portillo A.; Baldoví H. G.; Carbonell E.; Navalón S.; Álvaro M.; García H.; Ferrer B. Ruthenium(II) Tris(2,2′-bipyridyl) Complex Incorporated in UiO-67 as Photoredox Catalyst. J. Phys. Chem. C 2018, 122, 29190–29199. 10.1021/acs.jpcc.8b07204.

[ref159] Tachibana Y.; Moser J. E.; Grätzel M.; Klug D. R.; Durrant J. R. Subpicosecond Interfacial Charge Separation in Dye-Sensitized Nanocrystalline Titanium Dioxide Films. J. Phys. Chem. 1996, 100, 20056–20062. 10.1021/jp962227f.

[ref160] Reynal A.; Lakadamyali F.; Gross M. A.; Reisner E.; Durrant J. R. Parameters Affecting Electron Transfer Dynamics from Semiconductors to Molecular Catalysts for the Photochemical Reduction of Protons. Energy Environ. Sci. 2013, 6, 3291 10.1039/c3ee40961a.

[ref161] Bozal-Ginesta C.; Mesa C. A.; Eisenschmidt A.; Francas L.; Shankar R. B.; Anton-Garcia D.; Warnan J.; Willkomm J.; Reynal A.; Reisner E.; et al. Charge Accumulation Kinetics in Multi-Redox Molecular Catalysts Immobilised on TiO_2_. Chem. Sci. 2021, 12, 946–959. 10.1039/D0SC04344C. PMC8178996 34163861

[ref162] Hammarström L. Accumulative Charge Separation for Solar Fuels Production: Coupling Light-Induced Single Electron Transfer to Multielectron Catalysis. Acc. Chem. Res. 2015, 48, 840–850. 10.1021/ar500386x. 25675365

[ref163] Karlsson S.; Boixel J.; Pellegrin Y.; Blart E.; Becker H. C.; Odobel F.; Hammarström L. Accumulative Charge Separation Inspired by Photosynthesis. J. Am. Chem. Soc. 2010, 132, 17977–17979. 10.1021/ja104809x. 21138258

[ref164] Karlsson S.; Boixel J.; Pellegrin Y.; Blart E.; Becker H. C.; Odobel F.; Hammarström L. Accumulative Electron Transfer: Multiple Charge Separation in Artificial Photosynthesis. Faraday Discuss. 2012, 155, 233–52. 10.1039/C1FD00089F. 22470977

[ref165] Kuss-Petermann M.; Orazietti M.; Neuburger M.; Hamm P.; Wenger O. S. Intramolecular Light-Driven Accumulation of Reduction Equivalents by Proton-Coupled Electron Transfer. J. Am. Chem. Soc. 2017, 139, 5225–5232. 10.1021/jacs.7b01605. 28362497

[ref166] Nomrowski J.; Wenger O. S. Exploiting Potential Inversion for Photoinduced Multielectron Transfer and Accumulation of Redox Equivalents in a Molecular Heptad. J. Am. Chem. Soc. 2018, 140, 5343–5346. 10.1021/jacs.8b02443. 29652485

[ref167] Pannwitz A.; Wenger O. S. Proton-Coupled Multi-Electron Transfer and Its Relevance for Artificial Photosynthesis and Photoredox Catalysis. Chem. Commun. 2019, 55, 4004–4014. 10.1039/C9CC00821G. 30810148

[ref168] Tran T.-T.; Pino T.; Ha-Thi M.-H. Watching Intermolecular Light-Induced Charge Accumulation on Naphthalene Diimide by Tris(bipyridyl)ruthenium(II) Photosensitizer. J. Phys. Chem. C 2019, 123, 28651–28658. 10.1021/acs.jpcc.9b09556.

[ref169] Chen H. Y.; Ardo S. Direct Observation of Sequential Oxidations of a Titania-Bound Molecular Proxy Catalyst Generated through Illumination of Molecular Sensitizers. Nat. Chem. 2018, 10, 17–23. 10.1038/nchem.2892. 29256510

[ref170] Cardona T.; Sedoud A.; Cox N.; Rutherford A. W. Charge Separation in Photosystem II: A Comparative and Evolutionary Overview. Biochim. Biophys. Acta 2012, 1817, 26–43. 10.1016/j.bbabio.2011.07.012. 21835158

[ref171] Kent C. A.; Liu D.; Meyer T. J.; Lin W. Amplified Luminescence Quenching of Phosphorescent Metal-Organic Frameworks. J. Am. Chem. Soc. 2012, 134, 3991–3994. 10.1021/ja211271m. 22329430

[ref172] Son H. J.; Jin S.; Patwardhan S.; Wezenberg S. J.; Jeong N. C.; So M.; Wilmer C. E.; Sarjeant A. A.; Schatz G. C.; Snurr R. Q.; et al. Light-Harvesting and Ultrafast Energy Migration in Porphyrin-Based Metal-Organic Frameworks. J. Am. Chem. Soc. 2013, 135, 862–869. 10.1021/ja310596a. 23249338

[ref173] Choi S.; Jung W. J.; Park K.; Kim S. Y.; Baeg J. O.; Kim C. H.; Son H. J.; Pac C.; Kang S. O. Rapid Exciton Migration and Amplified Funneling Effects of Multi-Porphyrin Arrays in a Re(I)/Porphyrinic MOF Hybrid for Photocatalytic CO_2_ Reduction. ACS Appl. Mater. Interfaces 2021, 13, 2710–2722. 10.1021/acsami.0c19856. 33423462

[ref174] Markovic V.; Villamaina D.; Barabanov I.; Daku L. M.; Vauthey E. Photoinduced Symmetry-Breaking Charge Separation: The Direction of the Charge Transfer. Angew. Chem., Int. Ed. 2011, 50, 7596–7598. 10.1002/anie.201102601. 21728220

[ref175] Kaul N.; Lomoth R. The Carbene Cannibal: Photoinduced Symmetry-Breaking Charge Separation in an Fe(III) N-Heterocyclic Carbene. J. Am. Chem. Soc. 2021, 143, 10816–10821. 10.1021/jacs.1c03770. 34264638 PMC8397313

[ref176] Young R. M.; Wasielewski M. R. Mixed Electronic States in Molecular Dimers: Connecting Singlet Fission, Excimer Formation, and Symmetry-Breaking Charge Transfer. Acc. Chem. Res. 2020, 53, 1957–1968. 10.1021/acs.accounts.0c00397. 32786248

[ref177] Sebastian E.; Hariharan M. Symmetry-Breaking Charge Separation in Molecular Constructs for Efficient Light Energy Conversion. ACS Energy Lett. 2022, 7, 696–711. 10.1021/acsenergylett.1c02412.

[ref178] Baxendale J. H.; Rodgers M. A. J. Abnormal Decay Kinetics of the Excited State of Ruthenium Trisbipyridyl Ions in Surfactant Solutions. Chem. Phys. Lett. 1980, 72, 424–426. 10.1016/0009-2614(80)80322-8.

[ref179] Baxendale J. H.; Rodgers M. A. J. Fluorescence of Tris(2,2’-bipyridyl)ruthenium(II) in Sodium Dodecyl Sulfate Solutions Below the Critical Micelle Concentration. J. Phys. Chem. 1982, 86, 4906–4909. 10.1021/j100222a014.

[ref180] Ghosh P. K.; Bard A. J. Photochemistry of Tris(2,2’-bipyridyl)(ruthenium(II) in Colloidal Clay Suspensions. J. Phys. Chem. 1984, 88, 5519–5526. 10.1021/j150667a012.

[ref181] Cao W.; Tang Y.; Cui Y.; Qian G. Energy Transfer in Metal-Organic Frameworks and Its Applications. Small Struct. 2020, 1, 2000019 10.1002/sstr.202000019.

[ref182] Shaikh S. M.; Ilic S.; Gibbons B. J.; Yang X.; Jakubikova E.; Morris A. J. Role of a 3D Structure in Energy Transfer in Mixed-Ligand Metal-Organic Frameworks. J. Phys. Chem. C 2021, 125, 22998–23010. 10.1021/acs.jpcc.1c06427.

[ref183] Marcus R. A.; Sutin N. Electron Transfers in Chemistry and Biology. Biochim. Biophys. Acta, Rev. Bioenerg. 1985, 811, 265–322. 10.1016/0304-4173(85)90014-X.

[ref184] Closs G. L.; Miller J. R. Intramolecular Long-Distance Electron Transfer in Organic Molecules. Science 1988, 240, 440–447. 10.1126/science.240.4851.440. 17784065

[ref185] Weiss E. A.; Wasielewski M. R.; Ratner M. A. Molecules as Wires: Molecule-Assisted Movement of Charge and Energy. Top. Curr. Chem. 2005, 257, 103–133. 10.1007/b136068. 22179336

[ref186] An Y.; Liu Y.; An P.; Dong J.; Xu B.; Dai Y.; Qin X.; Zhang X.; Whangbo M. H.; Huang B. Ni(II) Coordination to an Al-Based Metal-Organic Framework Made from 2-Aminoterephthalate for Photocatalytic Overall Water Splitting. Angew. Chem., Int. Ed. 2017, 56, 3036–3040. 10.1002/anie.201612423. 28170148

[ref187] Dong L. Z.; Zhang L.; Liu J.; Huang Q.; Lu M.; Ji W. X.; Lan Y. Q. Stable Heterometallic Cluster-Based Organic Framework Catalysts for Artificial Photosynthesis. Angew. Chem., Int. Ed. 2020, 59, 2659–2663. 10.1002/anie.201913284. 31797510

[ref188] Gao W. Y.; Ngo H. T.; Niu Z.; Zhang W.; Pan Y.; Yang Z.; Bhethanabotla V. R.; Joseph B.; Aguila B.; Ma S. A Mixed-Metal Porphyrinic Framework Promoting Gas-Phase CO_2_ Photoreduction without Organic Sacrificial Agents. ChemSusChem 2020, 13, 6273–6277. 10.1002/cssc.202002414. 32743964

[ref189] Hu H.; Wang Z.; Cao L.; Zeng L.; Zhang C.; Lin W.; Wang C. Metal-Organic Frameworks Embedded in a Liposome Facilitate Overall Photocatalytic Water Splitting. Nat. Chem. 2021, 13, 358–366. 10.1038/s41557-020-00635-5. 33589788

[ref190] SK M.; Barman S.; Paul S.; De R.; Sreejith S. S.; Reinsch H.; Grzywa M.; Stock N.; Volkmer D.; Biswas S.; et al. An Anthracene-Based Metal-Organic Framework for Selective Photo-Reduction of Carbon Dioxide to Formic Acid Coupled with Water Oxidation. Chem. Eur. J. 2021, 27, 4098–4107. 10.1002/chem.202004596. 33226154

[ref191] Karmakar S.; Barman S.; Rahimi F. A.; Maji T. K. Covalent Grafting of Molecular Photosensitizer and Catalyst on MOF-808: Effect of Pore Confinement toward Visible Light-Driven CO_2_ Reduction in Water. Energy Environ. Sci. 2021, 14, 2429–2440. 10.1039/D0EE03643A.

[ref192] Ager J. W.; Shaner M. R.; Walczak K. A.; Sharp I. D.; Ardo S. Experimental Demonstrations of Spontaneous, Solar-Driven Photoelectrochemical Water Splitting. Energy Environ. Sci. 2015, 8, 2811–2824. 10.1039/C5EE00457H.

[ref193] Fabian D. M.; Hu S.; Singh N.; Houle F. A.; Hisatomi T.; Domen K.; Osterloh F. E.; Ardo S. Particle Suspension Reactors and Materials for Solar-Driven Water Splitting. Energy Environ. Sci. 2015, 8, 2825–2850. 10.1039/C5EE01434D.

[ref194] Cheng W.-H.; Richter M. H.; Sullivan I.; Larson D. M.; Xiang C.; Brunschwig B. S.; Atwater H. A. CO_2_ Reduction to CO with 19% Efficiency in a Solar-Driven Gas Diffusion Electrode Flow Cell under Outdoor Solar Illumination. ACS Energy Lett. 2020, 5, 470–476. 10.1021/acsenergylett.9b02576.

[ref195] Cheng W.-H.; Richter M. H.; May M. M.; Ohlmann J.; Lackner D.; Dimroth F.; Hannappel T.; Atwater H. A.; Lewerenz H.-J. Monolithic Photoelectrochemical Device for Direct Water Splitting with 19% Efficiency. ACS Energy Lett. 2018, 3, 1795–1800. 10.1021/acsenergylett.8b00920.

[ref196] Cox C. R.; Lee J. Z.; Nocera D. G.; Buonassisi T. Ten-Percent Solar-to-Fuel Conversion with Nonprecious Materials. Proc. Natl. Acad. Sci. U. S. A. 2014, 111, 14057–14061. 10.1073/pnas.1414290111. 25225379 PMC4191778

[ref197] Choi K. M.; Kim D.; Rungtaweevoranit B.; Trickett C. A.; Barmanbek J. T.; Alshammari A. S.; Yang P.; Yaghi O. M. Plasmon-Enhanced Photocatalytic CO_2_ Conversion within Metal-Organic Frameworks under Visible Light. J. Am. Chem. Soc. 2017, 139, 356–362. 10.1021/jacs.6b11027. 28004911

[ref198] Roy S.; Pascanu V.; Pullen S.; Miera G. G.; Martin-Matute B.; Ott S. Catalyst Accessibility to Chemical Reductants in Metal-Organic Frameworks. Chem. Commun. 2017, 53, 3257–3260. 10.1039/C7CC00022G. PMC5836565 28261731

[ref199] Reynal A.; Pastor E.; Gross M. A.; Selim S.; Reisner E.; Durrant J. R. Unravelling the pH-Dependence of a Molecular Photocatalytic System for Hydrogen Production. Chem. Sci. 2015, 6, 4855–4859. 10.1039/C5SC01349F. 28717491 PMC5502398

[ref200] Streich D.; Astuti Y.; Orlandi M.; Schwartz L.; Lomoth R.; Hammarström L.; Ott S. High-Turnover Photochemical Hydrogen Production Catalyzed by a Model Complex of the [FeFe]-Hydrogenase Active Site. Chem. Eur. J. 2010, 16, 60–63. 10.1002/chem.200902489. 19938018

[ref201] Streich D. Stepping into Catalysis: Kinetic and Mechanistic Investigations of Photo- and Electrocatalytic Hydrogen Production with Natural and Synthetic Molecular Catalysts. Ph.D. Thesis, University of Uppsala, Sweden, 2013; pp 56–59.

[ref202] Jasniewski A. J.; Que L. Jr. Dioxygen Activation by Nonheme Diiron Enzymes: Diverse Dioxygen Adducts, High-Valent Intermediates, and Related Model Complexes. Chem. Rev. 2018, 118, 2554–2592. 10.1021/acs.chemrev.7b00457. 29400961 PMC5920527

[ref203] Pullen S.; Fei H. H.; Orthaber A.; Cohen S. M.; Ott S. Enhanced Photochemical Hydrogen Production by a Molecular Diiron Catalyst Incorporated into a Metal-Organic Framework. J. Am. Chem. Soc. 2013, 135, 16997–17003. 10.1021/ja407176p. 24116734 PMC3829681

[ref204] Bozal-Ginesta C.; Pullen S.; Ott S.; Hammarström L. Self-Recovery of Photochemical H_2_ Evolution with a Molecular Diiron Catalyst Incorporated in a UiO-66 Metal-Organic Framework. Chemphotochem 2020, 4, 287–290. 10.1002/cptc.201900273.

[ref205] Singh P. S.; Rudbeck H. C.; Huang P.; Ezzaher S.; Eriksson L.; Stein M.; Ott S.; Lomoth R. (I,0) Mixed-Valence State of a Diiron Complex with Pertinence to the [FeFe]-Hydrogenase Active Site: An IR, EPR, and Computational Study. Inorg. Chem. 2009, 48, 10883–10885. 10.1021/ic9016454. 19888734

[ref206] Aguirre de Carcer I.; DiPasquale A.; Rheingold A. L.; Heinekey D. M. Active-Site Models for Iron Hydrogenases: Reduction Chemistry of Dinuclear Iron Complexes. Inorg. Chem. 2006, 45, 8000–8002. 10.1021/ic0610381. 16999394

[ref207] Sasan K.; Lin Q. P.; Mao C. Y.; Feng P. Y. Incorporation of Iron Hydrogenase Active Sites into a Highly Stable Metal-Organic Framework for Photocatalytic Hydrogen Generation. Chem. Commun. 2014, 50, 10390–10393. 10.1039/C4CC03946G. 25061635

[ref208] Wang W. J.; Song X. W.; Hong Z. X.; Li B. B.; Si Y. N.; Ji C. Q.; Su K. Z.; Tan Y. X.; Ju Z. F.; Huang Y. Y.; et al. Incorporation of Iron Hydrogenase Active Sites into a Stable Photosensitizing Metal-Organic Framework for Enhanced Hydrogen Production. Appl. Catal., B 2019, 258, 117979 10.1016/j.apcatb.2019.117979.

[ref209] Castner A. T.; Johnson B. A.; Cohen S. M.; Ott S. Mimicking the Electron Transport Chain and Active Site of [FeFe] Hydrogenases in One Metal-Organic Framework: Factors That Influence Charge Transport. J. Am. Chem. Soc. 2021, 143, 7991–7999. 10.1021/jacs.1c01361. 34029060 PMC8176456

[ref210] Yuan S.; Lu W.; Chen Y. P.; Zhang Q.; Liu T. F.; Feng D.; Wang X.; Qin J.; Zhou H. C. Sequential Linker Installation: Precise Placement of Functional Groups in Multivariate Metal-Organic Frameworks. J. Am. Chem. Soc. 2015, 137, 3177–3180. 10.1021/ja512762r. 25714137

[ref211] Dempsey J. L.; Brunschwig B. S.; Winkler J. R.; Gray H. B. Hydrogen Evolution Catalyzed by Cobaloximes. Acc. Chem. Res. 2009, 42, 1995–2004. 10.1021/ar900253e. 19928840

[ref212] El Ghachtouli S.; Fournier M.; Cherdo S.; Guillot R.; Charlot M.-F.; Anxolabéhère-Mallart E.; Robert M.; Aukauloo A. Monometallic Cobalt-Trisglyoximato Complexes as Precatalysts for Catalytic H_2_ Evolution in Water. J. Phys. Chem. C 2013, 117, 17073–17077. 10.1021/jp405134a.

[ref213] Anxolabéhère-Mallart E.; Costentin C.; Fournier M.; Robert M. Cobalt-Bisglyoximato Diphenyl Complex as a Precatalyst for Electrocatalytic H_2_ Evolution. J. Phys. Chem. C 2014, 118, 13377–13381. 10.1021/jp500813r.

[ref214] Kaeffer N.; Morozan A.; Fize J.; Martinez E.; Guetaz L.; Artero V. The Dark Side of Molecular Catalysis: Diimine-Dioxime Cobalt Complexes Are Not the Actual Hydrogen Evolution Electrocatalyst in Acidic Aqueous Solutions. ACS Catal. 2016, 6, 3727–3737. 10.1021/acscatal.6b00378.

[ref215] Lazarides T.; McCormick T.; Du P.; Luo G.; Lindley B.; Eisenberg R. Making Hydrogen from Water Using a Homogeneous System without Noble Metals. J. Am. Chem. Soc. 2009, 131, 9192–9194. 10.1021/ja903044n. 19566094

[ref216] Nasalevich M. A.; Becker R.; Ramos-Fernandez E. V.; Castellanos S.; Veber S. L.; Fedin M. V.; Kapteijn F.; Reek J. N. H.; van der Vlugt J. I.; Gascon J. Co@NH_2_-MIL-125(Ti): Cobaloxime-Derived Metal-Organic Framework-Based Composite for Light-Driven H_2_ Production. Energy Environ. Sci. 2015, 8, 364–375. 10.1039/C4EE02853H.

[ref217] Luo S.; Liu X. Y.; Wei X. J.; Fu M.; Lu P.; Li X.; Jia Y. M.; Ren Q.; He Y. Z. Noble-Metal-Free Cobaloxime Coupled with Metal-Organic Frameworks NH_2_-MIL-125: A Novel Bifunctional Photocatalyst for Photocatalytic NO Removal and H_2_ Evolution under Visible Light Irradiation. J. Hazard. Mater. 2020, 399, 122824 10.1016/j.jhazmat.2020.122824. 32535515

[ref218] Roy S.; Bhunia A.; Schuth N.; Haumann M.; Ott S. Light-Driven Hydrogen Evolution Catalyzed by a Cobaloxime Catalyst Incorporated in a MIL-101(Cr) Metal-Organic Framework. Sustainable Energy Fuels 2018, 2, 1148–1152. 10.1039/C8SE00072G. 30211322 PMC6130847

[ref219] Roy S.; Huang Z. H.; Bhunia A.; Castner A.; Gupta A. K.; Zou X. D.; Ott S. Electrocatalytic Hydrogen Evolution from a Cobaloxime-Based Metal-Organic Framework Thin Film. J. Am. Chem. Soc. 2019, 141, 15942–15950. 10.1021/jacs.9b07084. 31508946 PMC6803166

[ref220] Stracke J. J.; Finke R. G. Distinguishing Homogeneous from Heterogeneous Water Oxidation Catalysis When Beginning with Polyoxometalates. ACS Catal. 2014, 4, 909–933. 10.1021/cs4011716.

[ref221] Widegren J. A.; Finke R. G. A Review of the Problem of Distinguishing True Homogeneous Catalysis from Soluble or Other Metal-Particle Heterogeneous Catalysis under Reducing Conditions. J. Mol. Catal. A: Chem. 2003, 198, 317–341. 10.1016/S1381-1169(02)00728-8.

[ref222] Stracke J. J.; Finke R. G. Electrocatalytic Water Oxidation Beginning with the Cobalt Polyoxometalate [Co_4_(H_2_O)_2_(PW_9_O_34_)_2_]^10-^: Identification of Heterogeneous CoO_*x*_ as the Dominant Catalyst. J. Am. Chem. Soc. 2011, 133, 14872–5. 10.1021/ja205569j. 21894961

[ref223] Basu O.; Mukhopadhyay S.; Das S. K. Cobalt Based Functional Inorganic Materials: Electrocatalytic Water Oxidation. J. Chem. Sci. 2018, 130, 93 10.1007/s12039-018-1494-4.

[ref224] Buru C. T.; Farha O. K. Strategies for Incorporating Catalytically Active Polyoxometalates in Metal-Organic Frameworks for Organic Transformations. ACS Appl. Mater. Interfaces 2020, 12, 5345–5360. 10.1021/acsami.9b19785. 31961127

[ref225] Mialane P.; Mellot-Draznieks C.; Gairola P.; Duguet M.; Benseghir Y.; Oms O.; Dolbecq A. Heterogenisation of Polyoxometalates and Other Metal-Based Complexes in Metal-Organic Frameworks: From Synthesis to Characterisation and Applications in Catalysis. Chem. Soc. Rev. 2021, 50, 6152–6220. 10.1039/D0CS00323A. 34027956

[ref226] Zhang Z. M.; Zhang T.; Wang C.; Lin Z.; Long L. S.; Lin W. Photosensitizing Metal-Organic Framework Enabling Visible-Light-Driven Proton Reduction by a Wells-Dawson-Type Polyoxometalate. J. Am. Chem. Soc. 2015, 137, 3197–3200. 10.1021/jacs.5b00075. 25712689

[ref227] Kong X. J.; Lin Z. K.; Zhang Z. M.; Zhang T.; Lin W. B. Hierarchical Integration of Photosensitizing Metal-Organic Frameworks and Nickel-Containing Polyoxometalates for Efficient Visible-Light-Driven Hydrogen Evolution. Angew. Chem., Int. Ed. 2016, 55, 6411–6416. 10.1002/anie.201600431. 27094346

[ref228] Li H.; Yao S.; Wu H. L.; Qu J. Y.; Zhang Z. M.; Lu T. B.; Lin W. B.; Wang E. B. Charge-Regulated Sequential Adsorption of Anionic Catalysts and Cationic Photosensitizers into Metal-Organic Frameworks Enhances Photocatalytic Proton Reduction. Appl. Catal., B 2018, 224, 46–52. 10.1016/j.apcatb.2017.10.031.

[ref229] Nepal B.; Das S. Sustained Water Oxidation by a Catalyst Cage-Isolated in a Metal-Organic Framework. Angew. Chem., Int. Ed. 2013, 52, 7224–7227. 10.1002/anie.201301327. 23729244

[ref230] Bromberg L.; Diao Y.; Wu H.; Speakman S. A.; Hatton T. A. Chromium(III) Terephthalate Metal Organic Framework (MIL-101): Hf-Free Synthesis, Structure, Polyoxometalate Composites, and Catalytic Properties. Chem. Mater. 2012, 24, 1664–1675. 10.1021/cm2034382.

[ref231] Hansen R. E.; Das S. Biomimetic Di-Manganese Catalyst Cage-Isolated in a MOF: Robust Catalyst for Water Oxidation with Ce(IV), a Non-O-Donating Oxidant. Energy Environ. Sci. 2014, 7, 317–322. 10.1039/C3EE43040E.

[ref232] Manna P.; Debgupta J.; Bose S.; Das S. K. A Mononuclear Co(II) Coordination Complex Locked in a Confined Space and Acting as an Electrochemical Water-Oxidation Catalyst: A ″Ship-in-a-Bottle″ Approach. Angew. Chem., Int. Ed. 2016, 55, 2425–2430. 10.1002/anie.201509643. 26757444

[ref233] Park J.-E.; Oh H.; An J.; Shin I.-S. Post-Synthetic Modification of Mesoporous Zinc-Adeninate Framework with Tris(2,2′-bipyridine) Ruthenium(II) Complex and Its Electrochemiluminescence. Bull. Korean Chem. Soc. 2017, 38, 471–476. 10.1002/bkcs.11115.

[ref234] Meyer K.; Bashir S.; Llorca J.; Idriss H.; Ranocchiari M.; van Bokhoven J. A. Photocatalyzed Hydrogen Evolution from Water by a Composite Catalyst of NH_2_-MIL-125(Ti) and Surface Nickel(II) Species. Chem. Eur. J. 2016, 22, 13894–13899. 10.1002/chem.201601988. 27531470

[ref235] Li Z.; Xiao J. D.; Jiang H. L. Encapsulating a Co(II) Molecular Photocatalyst in Metal-Organic Framework for Visible-Light-Driven H_2_ Production: Boosting Catalytic Efficiency via Spatial Charge Separation. ACS Catal. 2016, 6, 5359–5365. 10.1021/acscatal.6b01293.

[ref236] Balestri D.; Roux Y.; Mattarozzi M.; Mucchino C.; Heux L.; Brazzolotto D.; Artero V.; Duboc C.; Pelagatti P.; Marchio L.; et al. Heterogenization of a [NiFe] Hydrogenase Mimic through Simple and Efficient Encapsulation into a Mesoporous MOF. Inorg. Chem. 2017, 56, 14801–14808. 10.1021/acs.inorgchem.7b01824. 29193978

[ref237] Yan Z.-H.; Ma B.; Li S.-R.; Liu J.; Chen R.; Du M.-H.; Jin S.; Zhuang G.-L.; Long L.-S.; Kong X.-J.; et al. Encapsulating a Ni(II) Molecular Catalyst in Photoactive Metal-Organic Framework for Highly Efficient Photoreduction of CO_2_. Sci. Bull. 2019, 64, 976–985. 10.1016/j.scib.2019.05.014. 36659809

[ref238] Zhang L.; Chen J.; Fan T.; Shen K.; Jiang M.; Li Y. A High-Valent Di-μ-Oxo Dimanganese Complex Covalently Anchored in a Metal-Organic Framework as a Highly Efficient and Recoverable Water Oxidation Catalyst. Chem. Commun. 2018, 54, 4188–4191. 10.1039/C8CC00258D. 29629466

[ref239] Bhunia A.; Johnson B. A.; Czapla-Masztafiak J.; Sa J.; Ott S. Formal Water Oxidation Turnover Frequencies from MIL-101(Cr) Anchored Ru(bda) Depend on Oxidant Concentration. Chem. Commun. 2018, 54, 7770–7773. 10.1039/C8CC02300J. PMC6040278 29926035

[ref240] Liang X.; Yang S.; Yang J.; Sun W.; Li X.; Ma B.; Huang J.; Zhang J.; Duan L.; Ding Y. Covalent Immobilization of Molecular Complexes on Metal-Organic Frameworks Towards Robust and Highly Efficient Heterogeneous Water Oxidation Catalysts. Appl. Catal., B 2021, 291, 120070 10.1016/j.apcatb.2021.120070.

[ref241] Wang X.; Wisser F. M.; Canivet J.; Fontecave M.; Mellot-Draznieks C. Immobilization of a Full Photosystem in the Large-Pore MIL-101 Metal-Organic Framework for CO_2_ Reduction. ChemSusChem 2018, 11, 3315–3322. 10.1002/cssc.201801066. 29978953

[ref242] Stanley P. M.; Thomas C.; Thyrhaug E.; Urstoeger A.; Schuster M.; Hauer J.; Rieger B.; Warnan J.; Fischer R. A. Entrapped Molecular Photocatalyst and Photosensitizer in Metal-Organic Framework Nanoreactors for Enhanced Solar CO_2_ Reduction. ACS Catal. 2021, 11, 871–882. 10.1021/acscatal.0c04673.

[ref243] Stanley P. M.; Haimerl J.; Thomas C.; Urstoeger A.; Schuster M.; Shustova N. B.; Casini A.; Rieger B.; Warnan J.; Fischer R. A. Host-Guest Interactions in a Metal-Organic Framework Isoreticular Series for Molecular Photocatalytic CO_2_ Reduction. Angew. Chem., Int. Ed. 2021, 60, 17854–17860. 10.1002/anie.202102729. PMC8453824 34014024

[ref244] Liu D. C.; Ouyang T.; Xiao R.; Liu W. J.; Zhong D. C.; Xu Z.; Lu T. B. Anchoring Co(II) Ions into a Thiol-Laced Metal-Organic Framework for Efficient Visible-Light-Driven Conversion of CO_2_ into CO. ChemSusChem 2019, 12, 2166–2170. 10.1002/cssc.201900338. 30740917

[ref245] Paille G.; Gomez-Mingot M.; Roch-Marchal C.; Lassalle-Kaiser B.; Mialane P.; Fontecave M.; Mellot-Draznieks C.; Dolbecq A. A Fully Noble Metal-Free Photosystem Based on Cobalt-Polyoxometalates Immobilized in a Porphyrinic Metal-Organic Framework for Water Oxidation. J. Am. Chem. Soc. 2018, 140, 3613–3618. 10.1021/jacs.7b11788. 29393639

[ref246] Paille G.; Gomez-Mingot M.; Roch-Marchal C.; Haouas M.; Benseghir Y.; Pino T.; Ha-Thi M. H.; Landrot G.; Mialane P.; Fontecave M.; et al. Thin Films of Fully Noble Metal-Free POM@MOF for Photocatalytic Water Oxidation. ACS Appl. Mater. Interfaces 2019, 11, 47837–47845. 10.1021/acsami.9b13121. 31773948

[ref247] Mukhopadhyay S.; Debgupta J.; Singh C.; Kar A.; Das S. K. A Keggin Polyoxometalate Shows Water Oxidation Activity at Neutral pH: POM@ZIF-8, an Efficient and Robust Electrocatalyst. Angew. Chem., Int. Ed. 2018, 57, 1918–1923. 10.1002/anie.201711920. 29240276

[ref248] Shimoni R.; He W.; Liberman I.; Hod I. Tuning of Redox Conductivity and Electrocatalytic Activity in Metal-Organic Framework Films via Control of Defect Site Density. J. Phys. Chem. C 2019, 123, 5531–5539. 10.1021/acs.jpcc.8b12392.

[ref249] Liberman I.; Shimoni R.; Ifraemov R.; Rozenberg I.; Singh C.; Hod I. Active-Site Modulation in an Fe-Porphyrin-Based Metal-Organic Framework through Ligand Axial Coordination: Accelerating Electrocatalysis and Charge-Transport Kinetics. J. Am. Chem. Soc. 2020, 142, 1933–1940. 10.1021/jacs.9b11355. 31910614 PMC7467674

[ref250] Pi Y. H.; Feng X. Y.; Song Y.; Xu Z. W.; Li Z.; Lin W. B. Metal-Organic Frameworks Integrate Cu Photosensitizers and Secondary Building Unit-Supported Fe Catalysts for Photocatalytic Hydrogen Evolution. J. Am. Chem. Soc. 2020, 142, 10302–10307. 10.1021/jacs.0c03906. 32449348

[ref251] Ma X.; Liu H.; Yang W.; Mao G.; Zheng L.; Jiang H. L. Modulating Coordination Environment of Single-Atom Catalysts and Their Proximity to Photosensitive Units for Boosting MOF Photocatalysis. J. Am. Chem. Soc. 2021, 143, 12220–12229. 10.1021/jacs.1c05032. 34324821

[ref252] Lan G.; Li Z.; Veroneau S. S.; Zhu Y. Y.; Xu Z.; Wang C.; Lin W. Photosensitizing Metal-Organic Layers for Efficient Sunlight-Driven Carbon Dioxide Reduction. J. Am. Chem. Soc. 2018, 140, 12369–12373. 10.1021/jacs.8b08357. 30220196

[ref253] Hu X.; Chen P.; Zhang C.; Wang Z.; Wang C. Energy Transfer on a Two-Dimensional Antenna Enhances the Photocatalytic Activity of CO_2_ Reduction by Metal-Organic Layers. Chem. Commun. 2019, 55, 9657–9660. 10.1039/C9CC04594E. 31342024

[ref254] Johnson B. A.; Bhunia A.; Ott S. Electrocatalytic Water Oxidation by a Molecular Catalyst Incorporated into a Metal-Organic Framework Thin Film. Dalton Trans. 2017, 46, 1382–1388. 10.1039/C6DT03718F. 27845800 PMC6095457

[ref255] Lin S.; Ravari A. K.; Zhu J.; Usov P. M.; Cai M.; Ahrenholtz S. R.; Pushkar Y.; Morris A. J. Insight into Metal-Organic Framework Reactivity: Chemical Water Oxidation Catalyzed by a [Ru(tpy)(dcbpy)(OH_2_)]^2+^-Modified UiO-67. ChemSusChem 2018, 11, 464–471. 10.1002/cssc.201701644. 29197150

[ref256] Lin S.; Pineda-Galvan Y.; Maza W. A.; Epley C. C.; Zhu J.; Kessinger M. C.; Pushkar Y.; Morris A. J. Electrochemical Water Oxidation by a Catalyst-Modified Metal-Organic Framework Thin Film. ChemSusChem 2017, 10, 514–522. 10.1002/cssc.201601181. 27976525

[ref257] Wang C.; Xie Z.; deKrafft K. E.; Lin W. Doping Metal-Organic Frameworks for Water Oxidation, Carbon Dioxide Reduction, and Organic Photocatalysis. J. Am. Chem. Soc. 2011, 133, 13445–13454. 10.1021/ja203564w. 21780787

[ref258] Chambers M. B.; Wang X.; Elgrishi N.; Hendon C. H.; Walsh A.; Bonnefoy J.; Canivet J.; Quadrelli E. A.; Farrusseng D.; Mellot-Draznieks C.; et al. Photocatalytic Carbon Dioxide Reduction with Rhodium-Based Catalysts in Solution and Heterogenized within Metal-Organic Frameworks. ChemSusChem 2015, 8, 603–608. 10.1002/cssc.201403345. 25613479

[ref259] Liao W.-M.; Zhang J.-H.; Wang Z.; Yin S.-Y.; Pan M.; Wang H.-P.; Su C.-Y. Post-Synthetic Exchange (PSE) of UiO-67 Frameworks with Ru/Rh Half-Sandwich Units for Visible-Light-Driven H_2_ Evolution and CO_2_ Reduction. J. Mater. Chem. A 2018, 6, 11337–11345. 10.1039/C8TA02962H.

[ref260] Benseghir Y.; Lemarchand A.; Duguet M.; Mialane P.; Gomez-Mingot M.; Roch-Marchal C.; Pino T.; Ha-Thi M. H.; Haouas M.; Fontecave M.; et al. Co-Immobilization of a Rh Catalyst and a Keggin Polyoxometalate in the UiO-67 Zr-Based Metal-Organic Framework: In Depth Structural Characterization and Photocatalytic Properties for CO_2_ Reduction. J. Am. Chem. Soc. 2020, 142, 9428–9438. 10.1021/jacs.0c02425. 32378888

[ref261] Chen C.; Wu T.; Wu H.; Liu H.; Qian Q.; Liu Z.; Yang G.; Han B. Highly Effective Photoreduction of CO_2_ to CO Promoted by Integration of Cds with Molecular Redox Catalysts through Metal-Organic Frameworks. Chem. Sci. 2018, 9, 8890–8894. 10.1039/C8SC02809E. 30627408 PMC6296294

[ref262] Hou C. C.; Li T. T.; Cao S.; Chen Y.; Fu W. F. Incorporation of a [Ru(dcbpy)(bpy)_2_]^2+^ Photosensitizer and a Pt(dcbpy)Cl_2_ Catalyst into Metal-Organic Frameworks for Photocatalytic Hydrogen Evolution from Aqueous Solution. J. Mater. Chem. A 2015, 3, 10386–10394. 10.1039/C5TA01135C.

[ref263] Yang S. Z.; Fan D. H.; Hu W. H.; Pattengale B.; Liu C. M.; Zhang X. Y.; Huang J. E. Elucidating Charge Separation Dynamics in a Hybrid Metal-Organic Framework Photocatalyst for Light-Driven H_2_ Evolution. J. Phys. Chem. C 2018, 122, 3305–3311. 10.1021/acs.jpcc.8b00471.

[ref264] Yang S. Z.; Pattengale B.; Lee S.; Huang J. Real-Time Visualization of Active Species in a Single-Site Metal-Organic Framework Photocatalyst. ACS Energy Lett. 2018, 3, 532–539. 10.1021/acsenergylett.8b00062.

[ref265] Kim D.; Whang D. R.; Park S. Y. Self-Healing of Molecular Catalyst and Photosensitizer on Metal-Organic Framework: Robust Molecular System for Photocatalytic H_2_ Evolution from Water. J. Am. Chem. Soc. 2016, 138, 8698–8701. 10.1021/jacs.6b04552. 27356034

[ref266] Liu M.; Mu Y.-F.; Yao S.; Guo S.; Guo X.-W.; Zhang Z.-M.; Lu T.-B. Photosensitizing Single-Site Metal-Organic Framework Enabling Visible-Light-Driven CO_2_ Reduction for Syngas Production. Appl. Catal., B 2019, 245, 496–501. 10.1016/j.apcatb.2019.01.014.

[ref267] Sun D.; Gao Y.; Fu J.; Zeng X.; Chen Z.; Li Z. Construction of a Supported Ru Complex on Bifunctional MOF-253 for Photocatalytic CO_2_ Reduction under Visible Light. Chem. Commun. 2015, 51, 2645–2648. 10.1039/C4CC09797A. 25572688

[ref268] Johnson F. P. A.; George M. W.; Hartl F.; Turner J. J. Electrocatalytic Reduction of CO_2_ Using the Complexes [Re(bpy)(CO)_3_L]^*n*^ (*n* = + 1, L = P(OEt)_3_, CH_3_CN; *n* = 0, L = Cl^–^, Otf^–^; bpy = 2,2’-Bipyridine; Otf^–^= CF_3_SO_3_) as Catalyst Precursors: Infrared Spectroelectrochemical Investigation. Organometallics 1996, 15, 3374–3387. 10.1021/om960044+.

[ref269] Benson E. E.; Kubiak C. P. Structural Investigations into the Deactivation Pathway of the CO_2_ Reduction Electrocatalyst Re(bpy)(CO)_3_Cl. Chem. Commun. 2012, 48, 7374–7376. 10.1039/c2cc32617e. 22714095

[ref270] Fei H.; Sampson M. D.; Lee Y.; Kubiak C. P.; Cohen S. M. Photocatalytic CO_2_ Reduction to Formate Using a Mn(I) Molecular Catalyst in a Robust Metal-Organic Framework. Inorg. Chem. 2015, 54, 6821–6828. 10.1021/acs.inorgchem.5b00752. 26135673

[ref271] Deng X.; Albero J.; Xu L.; Garcia H.; Li Z. Construction of a Stable Ru-Re Hybrid System Based on Multifunctional MOF-253 for Efficient Photocatalytic CO_2_ Reduction. Inorg. Chem. 2018, 57, 8276–8286. 10.1021/acs.inorgchem.8b00896. 29965734

[ref272] Huang R.; Peng Y.; Wang C.; Shi Z.; Lin W. A Rhenium-Functionalized Metal-Organic Framework as a Single-Site Catalyst for Photochemical Reduction of Carbon Dioxide. Eur. J. Inorg. Chem. 2016, 2016, 4358–4362. 10.1002/ejic.201600064.

[ref273] Feng X.; Pi Y.; Song Y.; Brzezinski C.; Xu Z.; Li Z.; Lin W. Metal-Organic Frameworks Significantly Enhance Photocatalytic Hydrogen Evolution and CO_2_ Reduction with Earth-Abundant Copper Photosensitizers. J. Am. Chem. Soc. 2020, 142, 690–695. 10.1021/jacs.9b12229. 31895984

[ref274] Feng H.; Li H.; Liu X.; Huang Y.; Pan Q.; Peng R.; Du R.; Zheng X.; Yin Z.; Li S.; et al. Porphyrin-Based Ti-MOFs Conferred with Single-Atom Pt for Enhanced Photocatalytic Hydrogen Evolution and NO Removal. Chem. Eng. J. 2022, 428, 132045 10.1016/j.cej.2021.132045.

[ref275] Lions M.; Tommasino J. B.; Chattot R.; Abeykoon B.; Guillou N.; Devic T.; Demessence A.; Cardenas L.; Maillard F.; Fateeva A. Insights into the Mechanism of Electrocatalysis of the Oxygen Reduction Reaction by a Porphyrinic Metal Organic Framework. Chem. Commun. 2017, 53, 6496–6499. 10.1039/C7CC02113E. 28569312

[ref276] Sohrabi S.; Dehghanpour S.; Ghalkhani M. A Cobalt Porphyrin-Based Metal Organic Framework/Multi-Walled Carbon Nanotube Composite Electrocatalyst for Oxygen Reduction and Evolution Reactions. J. Mater. Sci. 2018, 53, 3624–3639. 10.1007/s10853-017-1768-0.

[ref277] Liang Z.; Guo H.; Zhou G.; Guo K.; Wang B.; Lei H.; Zhang W.; Zheng H.; Apfel U.-P.; Cao R. Metal-Organic-Framework-Supported Molecular Electrocatalysis for the Oxygen Reduction Reaction. Angew. Chem., Int. Ed. 2021, 60, 8472–8476. 10.1002/anie.202016024. 33484092

[ref278] Liu Y.; Yang Y.; Sun Q.; Wang Z.; Huang B.; Dai Y.; Qin X.; Zhang X. Chemical Adsorption Enhanced CO_2_ Capture and Photoreduction over a Copper Porphyrin Based Metal Organic Framework. ACS Appl. Mater. Interfaces 2013, 5, 7654–7658. 10.1021/am4019675. 23808795

[ref279] Zhang H.; Wei J.; Dong J.; Liu G.; Shi L.; An P.; Zhao G.; Kong J.; Wang X.; Meng X.; et al. Efficient Visible-Light-Driven Carbon Dioxide Reduction by a Single-Atom Implanted Metal-Organic Framework. Angew. Chem., Int. Ed. 2016, 55, 14310–14314. 10.1002/anie.201608597. 27736031

[ref280] Huang N. Y.; Zhang X. W.; Xu Y. Z.; Liao P. Q.; Chen X. M. A Local Hydrophobic Environment in a Metal-Organic Framework for Boosting Photocatalytic CO_2_ Reduction in the Presence of Water. Chem. Commun. 2019, 55, 14781–14784. 10.1039/C9CC08094E. 31755885

[ref281] Hod I.; Sampson M. D.; Deria P.; Kubiak C. P.; Farha O. K.; Hupp J. T. Fe-Porphyrin-Based Metal-Organic Framework Films as High-Surface Concentration, Heterogeneous Catalysts for Electrochemical Reduction of CO_2_. ACS Catal. 2015, 5, 6302–6309. 10.1021/acscatal.5b01767.

[ref282] Sadeghi N.; Sharifnia S.; Sheikh Arabi M. A Porphyrin-Based Metal Organic Framework for High Rate Photoreduction of CO_2_ to CH_4_ in Gas Phase. J. CO2 Util. 2016, 16, 450–457. 10.1016/j.jcou.2016.10.006.

[ref283] Zhou Y.; Chen S.; Xi S.; Wang Z.; Deng P.; Yang F.; Han Y.; Pang Y.; Xia B. Y. Spatial Confinement in Copper-Porphyrin Frameworks Enhances Carbon Dioxide Reduction to Hydrocarbons. Cell Rep. Phys. Sci. 2020, 1, 100182 10.1016/j.xcrp.2020.100182.

[ref284] Wu J. X.; Hou S. Z.; Zhang X. D.; Xu M.; Yang H. F.; Cao P. S.; Gu Z. Y. Cathodized Copper Porphyrin Metal-Organic Framework Nanosheets for Selective Formate and Acetate Production from CO_2_ Electroreduction. Chem. Sci. 2019, 10, 2199–2205. 10.1039/C8SC04344B. 30881645 PMC6385528

[ref285] Qin J. H.; Xu P.; Huang Y. D.; Xiao L. Y.; Lu W.; Yang X. G.; Ma L. F.; Zang S. Q. High Loading of Mn(II)-Metalated Porphyrin in a MOF for Photocatalytic CO_2_ Reduction in Gas-Solid Conditions. Chem. Commun. 2021, 57, 8468–8471. 10.1039/D1CC02847B. 34346420

[ref286] Huang H. B.; Fang Z. B.; Wang R.; Li L.; Khanpour M.; Liu T. F.; Cao R. Engineering Hierarchical Architecture of Metal-Organic Frameworks for Highly Efficient Overall CO_2_ Photoreduction. Small 2022, 18, e2200407 10.1002/smll.202200407. 35266311

[ref287] Matheu R.; Gutierrez-Puebla E.; Monge M. A.; Diercks C. S.; Kang J.; Prevot M. S.; Pei X.; Hanikel N.; Zhang B.; Yang P.; et al. Three-Dimensional Phthalocyanine Metal-Catecholates for High Electrochemical Carbon Dioxide Reduction. J. Am. Chem. Soc. 2019, 141, 17081–17085. 10.1021/jacs.9b09298. 31613614

[ref288] Meng Z.; Luo J.; Li W.; Mirica K. A. Hierarchical Tuning of the Performance of Electrochemical Carbon Dioxide Reduction Using Conductive Two-Dimensional Metallophthalocyanine Based Metal-Organic Frameworks. J. Am. Chem. Soc. 2020, 142, 21656–21669. 10.1021/jacs.0c07041. 33305565

[ref289] Qiu X. F.; Zhu H. L.; Huang J. R.; Liao P. Q.; Chen X. M. Highly Selective CO_2_ Electroreduction to C_2_H_4_ Using a Metal-Organic Framework with Dual Active Sites. J. Am. Chem. Soc. 2021, 143, 7242–7246. 10.1021/jacs.1c01466. 33956435

[ref290] Gong Y. N.; Ouyang T.; He C. T.; Lu T. B. Photoinduced Water Oxidation by an Organic Ligand Incorporated into the Framework of a Stable Metal-Organic Framework. Chem. Sci. 2016, 7, 1070–1075. 10.1039/C5SC02679B. 28936323 PMC5590092

[ref291] Chen K.; Ray D.; Ziebel M. E.; Gaggioli C. A.; Gagliardi L.; Marinescu S. C. Cu[Ni(2,3-pyrazinedithiolate)_2_] Metal-Organic Framework for Electrocatalytic Hydrogen Evolution. ACS Appl. Mater. Interfaces 2021, 13, 34419–34427. 10.1021/acsami.1c08998. 34275268

[ref292] Dou S.; Song J.; Xi S.; Du Y.; Wang J.; Huang Z. F.; Xu Z. J.; Wang X. Boosting Electrochemical CO_2_ Reduction on Metal-Organic Frameworks via Ligand Doping. Angew. Chem., Int. Ed. 2019, 58, 4041–4045. 10.1002/anie.201814711. 30688394

[ref293] Liseev T.; Howe A.; Hoque M. A.; Gimbert-Surinach C.; Llobet A.; Ott S. Synthetic Strategies to Incorporate Ru-Terpyridyl Water Oxidation Catalysts into MOFs: Direct Synthesis vs. Post-Synthetic Approach. Dalton Trans. 2020, 49, 13753–13759. 10.1039/D0DT01890B. 32996947 PMC7116355

[ref294] Howe A.; Liseev T.; Gil-Sepulcre M.; Gimbert-Surinach C.; Benet-Buchholz J.; Llobet A.; Ott S. Electrocatalytic Water Oxidation from a Mixed Linker MOF Based on NU-1000 with an Integrated Ruthenium-Based Metallo-Linker. Mater. Adv. 2022, 3, 4227–4234. 10.1039/D2MA00128D. 35693428 PMC9125567

[ref295] Matheu R.; Ertem M. Z.; Benet-Buchholz J.; Coronado E.; Batista V. S.; Sala X.; Llobet A. Intramolecular Proton Transfer Boosts Water Oxidation Catalyzed by a Ru Complex. J. Am. Chem. Soc. 2015, 137, 10786–10795. 10.1021/jacs.5b06541. 26226390

[ref296] Fu Y.; Sun D.; Chen Y.; Huang R.; Ding Z.; Fu X.; Li Z. An Amine-Functionalized Titanium Metal-Organic Framework Photocatalyst with Visible-Light-Induced Activity for CO_2_ Reduction. Angew. Chem., Int. Ed. 2012, 51, 3364–3367. 10.1002/anie.201108357. 22359408

[ref297] Luo T.; Zhang J.; Li W.; He Z.; Sun X.; Shi J.; Shao D.; Zhang B.; Tan X.; Han B. Metal-Organic Framework-Stabilized CO_2_/Water Interfacial Route for Photocatalytic CO_2_ Conversion. ACS Appl. Mater. Interfaces 2017, 9, 41594–41598. 10.1021/acsami.7b13900. 29110454

[ref298] Sun D.; Fu Y.; Liu W.; Ye L.; Wang D.; Yang L.; Fu X.; Li Z. Studies on Photocatalytic CO_2_ Reduction over NH_2_-UiO-66(Zr) and Its Derivatives: Towards a Better Understanding of Photocatalysis on Metal-Organic Frameworks. Chem. Eur. J. 2013, 19, 14279–14285. 10.1002/chem.201301728. 24038375

[ref299] Sun D.; Liu W.; Qiu M.; Zhang Y.; Li Z. Introduction of a Mediator for Enhancing Photocatalytic Performance via Post-Synthetic Metal Exchange in Metal-Organic Frameworks (MOFs). Chem. Commun. 2015, 51, 2056–2059. 10.1039/C4CC09407G. 25532612

[ref300] Kay A.; Grätzel M. Dye-Sensitized Core-Shell Nanocrystals: Improved Efficiency of Mesoporous Tin Oxide Electrodes Coated with a Thin Layer of an Insulating Oxide. Chem. Mater. 2002, 14, 2930–2935. 10.1021/cm0115968.

[ref301] Wei Y. P.; Liu Y.; Guo F.; Dao X. Y.; Sun W. Y. Different Functional Group Modified Zirconium Frameworks for the Photocatalytic Reduction of Carbon Dioxide. Dalton Trans. 2019, 48, 8221–8226. 10.1039/C9DT01767D. 31090769

[ref302] Wang D.; Huang R.; Liu W.; Sun D.; Li Z. Fe-Based MOFs for Photocatalytic CO_2_ Reduction: Role of Coordination Unsaturated Sites and Dual Excitation Pathways. ACS Catal. 2014, 4, 4254–4260. 10.1021/cs501169t.

[ref303] Chen D.; Xing H.; Wang C.; Su Z. Highly Efficient Visible-Light-Driven CO_2_ Reduction to Formate by a New Anthracene-Based Zirconium MOF via Dual Catalytic Routes. J. Mater. Chem. A 2016, 4, 2657–2662. 10.1039/C6TA00429F.

[ref304] Sun M.; Yan S.; Sun Y.; Yang X.; Guo Z.; Du J.; Chen D.; Chen P.; Xing H. Enhancement of Visible-Light-Driven CO_2_ Reduction Performance Using an Amine-Functionalized Zirconium Metal-Organic Framework. Dalton Trans. 2018, 47, 909–915. 10.1039/C7DT04062H. 29257163

[ref305] Logan M. W.; Ayad S.; Adamson J. D.; Dilbeck T.; Hanson K.; Uribe-Romo F. J. Systematic Variation of the Optical Bandgap in Titanium Based Isoreticular Metal-Organic Frameworks for Photocatalytic Reduction of CO_2_ under Blue Light. J. Mater. Chem. A 2017, 5, 11854–11863. 10.1039/C7TA00437K.

[ref306] Chen S.; Yang F.; Gao H.; Wang J.; Chen X.; Zhang X.; Li J.; Li A. Construction of Dual Ligand Ti-Based MOFs with Enhanced Photocatalytic CO_2_ Reduction Performance. J. CO2 Util. 2021, 48, 101528 10.1016/j.jcou.2021.101528.

[ref307] Qin J. S.; Yuan S.; Zhang L.; Li B.; Du D. Y.; Huang N.; Guan W.; Drake H. F.; Pang J.; Lan Y. Q.; et al. Creating Well-Defined Hexabenzocoronene in Zirconium Metal-Organic Framework by Postsynthetic Annulation. J. Am. Chem. Soc. 2019, 141, 2054–2060. 10.1021/jacs.8b11042. 30621391

[ref308] Qiu Y. C.; Yuan S.; Li X. X.; Du D. Y.; Wang C.; Qin J. S.; Drake H. F.; Lan Y. Q.; Jiang L.; Zhou H. C. Face-Sharing Archimedean Solids Stacking for the Construction of Mixed-Ligand Metal-Organic Frameworks. J. Am. Chem. Soc. 2019, 141, 13841–13848. 10.1021/jacs.9b05580. 31343873

[ref309] Aziz A.; Ruiz-Salvador A. R.; Hernandez N. C.; Calero S.; Hamad S.; Grau-Crespo R. Porphyrin-Based Metal-Organic Frameworks for Solar Fuel Synthesis Photocatalysis: Band Gap Tuning via Iron Substitutions. J. Mater. Chem. A 2017, 5, 11894–11904. 10.1039/C7TA01278K.

[ref310] Zeng J. Y.; Wang X. S.; Xie B. R.; Li Q. R.; Zhang X. Z. Large Pi-Conjugated Metal-Organic Frameworks for Infrared-Light-Driven CO_2_ Reduction. J. Am. Chem. Soc. 2022, 144, 1218–1231. 10.1021/jacs.1c10110. 35029380

[ref311] Nguyen A. I.; Van Allsburg K. M.; Terban M. W.; Bajdich M.; Oktawiec J.; Amtawong J.; Ziegler M. S.; Dombrowski J. P.; Lakshmi K. V.; Drisdell W. S.; et al. Stabilization of Reactive Co_4_O_4_ Cubane Oxygen-Evolution Catalysts within Porous Frameworks. Proc. Natl. Acad. Sci. U. S. A. 2019, 116, 11630–11639. 10.1073/pnas.1815013116. 31142656 PMC6575163

[ref312] Wang D.; Meyer T. J. A Strategy for Stabilizing the Catalyst Co_4_O_4_ in a Metal-Organic Framework. Proc. Natl. Acad. Sci. U. S. A. 2019, 116, 13719–13720. 10.1073/pnas.1909543116. 31221748 PMC6628654

[ref313] Yang S. Z.; Pattengale B.; Kovrigin E. L.; Huang J. Photoactive Zeolitic Imidazolate Framework as Intrinsic Heterogeneous Catalysts for Light-Driven Hydrogen Generation. ACS Energy Lett. 2017, 2, 75–80. 10.1021/acsenergylett.6b00540.

[ref314] Qin J.; Wang S.; Wang X. Visible-Light Reduction CO_2_ with Dodecahedral Zeolitic Imidazolate Framework ZIF-67 as an Efficient Co-Catalyst. Appl. Catal., B 2017, 209, 476–482. 10.1016/j.apcatb.2017.03.018.

[ref315] Liu Y. H.; Zhang F. J.; Wu P. Y.; Deng C. F.; Yang Q. M.; Xue J. J.; Shi Y. H.; Wang J. Cobalt(II)-Based Metal-Organic Framework as Bifunctional Materials for Ag(I) Detection and Proton Reduction Catalysis for Hydrogen Production. Inorg. Chem. 2019, 58, 924–931. 10.1021/acs.inorgchem.8b03046. 30576126

[ref316] Wang S.; Yao W.; Lin J.; Ding Z.; Wang X. Cobalt Imidazolate Metal-Organic Frameworks Photosplit CO_2_ under Mild Reaction Conditions. Angew. Chem., Int. Ed. 2014, 53, 1034–1038. 10.1002/anie.201309426. 24339134

[ref317] Zhao J.; Wang Q.; Sun C.; Zheng T.; Yan L.; Li M.; Shao K.; Wang X.; Su Z. A Hexanuclear Cobalt Metal-Organic Framework for Efficient CO_2_ Reduction under Visible Light. J. Mater. Chem. A 2017, 5, 12498–12505. 10.1039/C7TA02611K.

[ref318] Liao W.-M.; Zhang J.-H.; Wang Z.; Lu Y.-L.; Yin S.-Y.; Wang H.-P.; Fan Y.-N.; Pan M.; Su C.-Y. Semiconductive Amine-Functionalized Co(II)-MOF for Visible-Light-Driven Hydrogen Evolution and CO_2_ Reduction. Inorg. Chem. 2018, 57, 11436–11442. 10.1021/acs.inorgchem.8b01265. 30152695

[ref319] Shi D. Y.; Cui C. J.; Hu M.; Ren A. H.; Song L. B.; Liu C. S.; Du M. A Microporous Mixed-Metal (Na/Cu) Mixed-Ligand (Flexible/Rigid) Metal-Organic Framework for Photocatalytic H_2_ Generation. J. Mater. Chem. C 2019, 7, 10211–10217. 10.1039/C9TC03342D.

[ref320] Wang Z. D.; Zang Y.; Liu Z. J.; Peng P.; Wang R.; Zang S. Q. Opening Catalytic Sites in the Copper-Triazoles Framework via Defect Chemistry for Switching on the Proton Reduction. Appl. Catal., B 2021, 288, 119941 10.1016/j.apcatb.2021.119941.

[ref321] Mao J.; Yang L.; Yu P.; Wei X.; Mao L. Electrocatalytic Four-Electron Reduction of Oxygen with Copper (II)-Based Metal-Organic Frameworks. Electrochem. Commun. 2012, 19, 29–31. 10.1016/j.elecom.2012.02.025.

[ref322] Senthil Kumar R.; Senthil Kumar S.; Anbu Kulandainathan M. Highly Selective Electrochemical Reduction of Carbon Dioxide Using Cu Based Metal Organic Framework as an Electrocatalyst. Electrochem. Commun. 2012, 25, 70–73. 10.1016/j.elecom.2012.09.018.

[ref323] Zhang H. X.; Hong Q. L.; Li J.; Wang F.; Huang X.; Chen S.; Tu W.; Yu D.; Xu R.; Zhou T.; et al. Isolated Square-Planar Copper Center in Boron Imidazolate Nanocages for Photocatalytic Reduction of CO_2_ to CO. Angew. Chem., Int. Ed. 2019, 58, 11752–11756. 10.1002/anie.201905869. 31232501

[ref324] Liu J.; Yang D.; Zhou Y.; Zhang G.; Xing G.; Liu Y.; Ma Y.; Terasaki O.; Yang S.; Chen L. Tricycloquinazoline-Based 2D Conductive Metal-Organic Frameworks as Promising Electrocatalysts for CO_2_ Reduction. Angew. Chem., Int. Ed. 2021, 60, 14473–14479. 10.1002/anie.202103398. 33826217

[ref325] Mani P.; Sheelam A.; Karthik P. E.; Sankar R.; Ramanujam K.; Mandal S. Nickel-Based Hybrid Material for Electrochemical Oxygen Redox Reactions in an Alkaline Medium. ACS Appl. Energy Mater. 2020, 3, 6408–6415. 10.1021/acsaem.0c00615.

[ref326] Feng Y. A.; Chen C.; Liu Z. G.; Fei B. J.; Lin P.; Li Q. P.; Sun S. G.; Du S. W. Application of a Ni Mercaptopyrimidine MOF as Highly Efficient Catalyst for Sunlight-Driven Hydrogen Generation. J. Mater. Chem. A 2015, 3, 7163–7169. 10.1039/C5TA00136F.

[ref327] Khrizanforova V.; Shekurov R.; Miluykov V.; Khrizanforov M.; Bon V.; Kaskel S.; Gubaidullin A.; Sinyashin O.; Budnikova Y. 3D Ni and Co Redox-Active Metal-Organic Frameworks Based on Ferrocenyl Diphosphinate and 4,4’-bipyridine Ligands as Efficient Electrocatalysts for the Hydrogen Evolution Reaction. Dalton Trans. 2020, 49, 2794–2802. 10.1039/C9DT04834K. 32073068

[ref328] Huo D. B.; Lin F. F.; Chen S.; Ni Y. R.; Wang R. H.; Chen H.; Duan L. L.; Ji Y. F.; Zhou A. J.; Tong L. P. Ruthenium Complex-Incorporated Two-Dimensional Metal-Organic Frameworks for Cocatalyst-Free Photocatalytic Proton Reduction from Water. Inorg. Chem. 2020, 59, 2379–2386. 10.1021/acs.inorgchem.9b03250. 32009398

[ref329] Iqbal R.; Akbar M. B.; Ahmad A.; Hussain A.; Altaf N.; Ibraheem S.; Yasin G.; Khan M. A.; Tabish M.; Kumar A.; et al. Exploring the Synergistic Effect of Novel Ni-Fe in 2D Bimetallic Metal-Organic Frameworks for Enhanced Electrochemical Reduction of CO_2_. Adv. Mater. Interfaces 2022, 9, 2101505 10.1002/admi.202101505.

[ref330] Kataoka Y.; Miyazaki Y.; Sato K.; Saito T.; Nakanishi Y.; Kiatagwa Y.; Kawakami T.; Okumura M.; Yamaguchi K.; Mori W. Modification of MOF Catalysts by Manipulation of Counter-Ions: Experimental and Theoretical Studies of Photochemical Hydrogen Production from Water over Microporous Diruthenium (II, III) Coordination Polymers. Supramol. Chem. 2011, 23, 287–296. 10.1080/10610278.2010.527976.

[ref331] Lan G. X.; Zhu Y. Y.; Veroneau S. S.; Xu Z. W.; Micheroni D.; Lin W. B. Electron Injection from Photoexcited Metal-Organic Framework Ligands to Ru_2_ Secondary Building Units for Visible-Light-Driven Hydrogen Evolution. J. Am. Chem. Soc. 2018, 140, 5326–5329. 10.1021/jacs.8b01601. 29578703

[ref332] Song Y.; Pi Y. H.; Feng X. Y.; Ni K. Y.; Xu Z. W.; Chen J. S.; Li Z.; Lin W. B. Cerium-Based Metal-Organic Layers Catalyze Hydrogen Evolution Reaction through Dual Photoexcitation. J. Am. Chem. Soc. 2020, 142, 6866–6871. 10.1021/jacs.0c00679. 32227854

[ref333] Yan Z. H.; Du M. H.; Liu J.; Jin S.; Wang C.; Zhuang G. L.; Kong X. J.; Long L. S.; Zheng L. S. Photo-Generated Dinuclear {Eu(II)}_2_ Active Sites for Selective CO_2_ Reduction in a Photosensitizing Metal-Organic Framework. Nat. Commun. 2018, 9, 3353 10.1038/s41467-018-05659-7. 30135431 PMC6105582

[ref334] Li F.; Gu G. H.; Choi C.; Kolla P.; Hong S.; Wu T.-S.; Soo Y.-L.; Masa J.; Mukerjee S.; Jung Y.; et al. Highly Stable Two-Dimensional Bismuth Metal-Organic Frameworks for Efficient Electrochemical Reduction of CO_2_. Appl. Catal., B 2020, 277, 119241 10.1016/j.apcatb.2020.119241.

[ref335] Lee M.; De Riccardis A.; Kazantsev R. V.; Cooper J. K.; Buckley A. K.; Burroughs P. W. W.; Larson D. M.; Mele G.; Toma F. M. Aluminum Metal-Organic Framework Triggers Carbon Dioxide Reduction Activity. ACS Appl. Energy Mater. 2020, 3, 1286–1291. 10.1021/acsaem.9b02210.

[ref336] Bie B.; Jiang Z.; Zhang J.; Deng H.; Yang C. Long Excited State Lifetime of Thermally Activated Delayed Fluorescent Photosensitizer Integrated into Metal-Organic Framework Enables Efficient CO_2_ Photoreduction. Chem. Eng. J. 2022, 431, 133897 10.1016/j.cej.2021.133897.

[ref337] Wang L.; Wu Y.; Cao R.; Ren L.; Chen M.; Feng X.; Zhou J.; Wang B. Fe/Ni Metal-Organic Frameworks and Their Binder-Free Thin Films for Efficient Oxygen Evolution with Low Overpotential. ACS Appl. Mater. Interfaces 2016, 8, 16736–16743. 10.1021/acsami.6b05375. 27300143

[ref338] Huang Z. Q.; Wang B.; Pan D. S.; Zhou L. L.; Guo Z. H.; Song J. L. Rational Design of a N,S Co-Doped Supermicroporous CoFe-Organic Framework Platform for Water Oxidation. ChemSusChem 2020, 13, 2564–2570. 10.1002/cssc.202000376. 32196953

[ref339] Wu C.; Zhang X.; Li H.; Xia Z.; Yu S.; Wang S.; Sun G. Iron-Based Binary Metal-Organic Framework Nanorods as an Efficient Catalyst for the Oxygen Evolution Reaction. Chin. J. Catal. 2021, 42, 637–647. 10.1016/S1872-2067(20)63686-5.

[ref340] Li F. L.; Wang P.; Huang X.; Young D. J.; Wang H. F.; Braunstein P.; Lang J. P. Large-Scale, Bottom-up Synthesis of Binary Metal-Organic Framework Nanosheets for Efficient Water Oxidation. Angew. Chem., Int. Ed. 2019, 58, 7051–7056. 10.1002/anie.201902588. 30913361

[ref341] Wang Q.; Liu F.; Wei C.; Li D.; Guo W.; Zhao Q. High Efficiency FeNi-Metal-Organic Framework Grown in-Situ on Nickel Foam for Electrocatalytic Oxygen Evolution. ChemistrySelect 2019, 4, 5988–5994. 10.1002/slct.201901709.

[ref342] Yao N.; Jia H.; Fan Z.; Bai L.; Xie W.; Cong H.; Chen S.; Luo W. Nitridation-Induced Metal-Organic Framework Nanosheet for Enhanced Water Oxidation Electrocatalysis. J. Energy Chem. 2022, 64, 531–537. 10.1016/j.jechem.2021.05.024.

[ref343] Yue K.; Liu J.; Zhu Y.; Xia C.; Wang P.; Zhang J.; Kong Y.; Wang X.; Yan Y.; Xia B. Y. In Situ Ion-Exchange Preparation and Topological Transformation of Trimetal-Organic Frameworks for Efficient Electrocatalytic Water Oxidation. Energy Environ. Sci. 2021, 14, 6546–6553. 10.1039/D1EE02606B.

[ref344] Yoon H.; Lee S.; Oh S.; Park H.; Choi S.; Oh M. Synthesis of Bimetallic Conductive 2D Metal-Organic Framework (Co_x_Ni_y_-CAT) and Its Mass Production: Enhanced Electrochemical Oxygen Reduction Activity. Small 2019, 15, e1805232 10.1002/smll.201805232. 30932335

[ref345] Shinde S. S.; Lee C. H.; Jung J.-Y.; Wagh N. K.; Kim S.-H.; Kim D.-H.; Lin C.; Lee S. U.; Lee J.-H. Unveiling Dual-Linkage 3D hexaiminobenzene Metal-Organic Frameworks Towards Long-Lasting Advanced Reversible Zn-Air Batteries. Energy Environ. Sci. 2019, 12, 727–738. 10.1039/C8EE02679C.

[ref346] Huang Q.; Liu J.; Feng L.; Wang Q.; Guan W.; Dong L.-Z.; Zhang L.; Yan L.-K.; Lan Y.-Q.; Zhou H.-C. Multielectron Transportation of Polyoxometalate-Grafted Metalloporphyrin Coordination Frameworks for Selective CO_2_-to-CH_4_ Photoconversion. Natl. Sci. Rev. 2020, 7, 53–63. 10.1093/nsr/nwz096. 34692017 PMC8288839

[ref347] Li X. X.; Liu J.; Zhang L.; Dong L. Z.; Xin Z. F.; Li S. L.; Huang-Fu X. Q.; Huang K.; Lan Y. Q. Hydrophobic Polyoxometalate-Based Metal-Organic Framework for Efficient CO_2_ Photoconversion. ACS Appl. Mater. Interfaces 2019, 11, 25790–25795. 10.1021/acsami.9b03861. 31240910

[ref348] Clough A. J.; Yoo J. W.; Mecklenburg M. H.; Marinescu S. C. Two-Dimensional Metal-Organic Surfaces for Efficient Hydrogen Evolution from Water. J. Am. Chem. Soc. 2015, 137, 118–121. 10.1021/ja5116937. 25525864

[ref349] Liu X.-H.; Hu W.-L.; Jiang W.-J.; Yang Y.-W.; Niu S.; Sun B.; Wu J.; Hu J.-S. Well-Defined Metal-O_6_ in Metal-Catecholates as a Novel Active Site for Oxygen Electroreduction. ACS Appl. Mater. Interfaces 2017, 9, 28473–28477. 10.1021/acsami.7b07410. 28792723

[ref350] Dong R. H.; Zheng Z. K.; Tranca D. C.; Zhang J.; Chandrasekhar N.; Liu S. H.; Zhuang X. D.; Seifert G.; Feng X. L. Immobilizing Molecular Metal Dithiolene-Diamine Complexes on 2D Metal-Organic Frameworks for Electrocatalytic H_2_ Production. Chem. Eur. J. 2017, 23, 2255–2260. 10.1002/chem.201605337. 27878872

[ref351] Zhou Y.; Sheng L.; Luo Q.; Zhang W.; Yang J. Improving the Activity of Electrocatalysts toward the Hydrogen Evolution Reaction, the Oxygen Evolution Reaction, and the Oxygen Reduction Reaction via Modification of Metal and Ligand of Conductive Two-Dimensional Metal-Organic Frameworks. J. Phys. Chem. Lett. 2021, 12, 11652–11658. 10.1021/acs.jpclett.1c03452. 34822246

[ref352] Zhu D.; Qiao M.; Liu J.; Tao T.; Guo C. Engineering Pristine 2D Metal-Organic Framework Nanosheets for Electrocatalysis. J. Mater. Chem. A 2020, 8, 8143–8170. 10.1039/D0TA03138K.

[ref353] Chen G.; Stevens M. B.; Liu Y.; King L. A.; Park J.; Kim T. R.; Sinclair R.; Jaramillo T. F.; Bao Z. Nanosized Zirconium Porphyrinic Metal-Organic Frameworks That Catalyze the Oxygen Reduction Reaction in Acid. Small Methods 2020, 4, 2000085 10.1002/smtd.202000085.

[ref354] Kornienko N.; Zhao Y.; Kley C. S.; Zhu C.; Kim D.; Lin S.; Chang C. J.; Yaghi O. M.; Yang P. Metal-Organic Frameworks for Electrocatalytic Reduction of Carbon Dioxide. J. Am. Chem. Soc. 2015, 137, 14129–14135. 10.1021/jacs.5b08212. 26509213

[ref355] Downes C. A.; Clough A. J.; Chen K. Y.; Yoo J. W.; Marinescu S. C. Evaluation of the H_2_ Evolving Activity of Benzenehexathiolate Coordination Frameworks and the Effect of Film Thickness on H_2_ Production. ACS Appl. Mater. Interfaces 2018, 10, 1719–1727. 10.1021/acsami.7b15969. 29251487

[ref356] Duan J.; Chen S.; Zhao C. Ultrathin Metal-Organic Framework Array for Efficient Electrocatalytic Water Splitting. Nat. Commun. 2017, 8, 15341 10.1038/ncomms15341. 28580963 PMC5465318

[ref357] Cheng W.; Zhang H.; Luan D.; Lou X. W. D. Exposing Unsaturated Cu_1_-O_2_ Sites in Nanoscale Cu-MOF for Efficient Electrocatalytic Hydrogen Evolution. Sci. Adv. 2021, 7, eabg2580 10.1126/sciadv.abg2580. 33910899 PMC8081372

[ref358] Kang X.; Li L.; Sheveleva A.; Han X.; Li J.; Liu L.; Tuna F.; McInnes E. J. L.; Han B.; Yang S.; et al. Electro-Reduction of Carbon Dioxide at Low over-Potential at a Metal-Organic Framework Decorated Cathode. Nat. Commun. 2020, 11, 5464 10.1038/s41467-020-19236-4. 33122645 PMC7596083

[ref359] Kang X.; Wang B.; Hu K.; Lyu K.; Han X.; Spencer B. F.; Frogley M. D.; Tuna F.; McInnes E. J. L.; Dryfe R. A. W.; et al. Quantitative Electro-Reduction of CO_2_ to Liquid Fuel over Electro-Synthesized Metal-Organic Frameworks. J. Am. Chem. Soc. 2020, 142, 17384–17392. 10.1021/jacs.0c05913. 32997941 PMC7586324

[ref360] Ye L.; Liu J.; Gao Y.; Gong C.; Addicoat M.; Heine T.; Wöll C.; Sun L. Highly Oriented MOF Thin Film-Based Electrocatalytic Device for the Reduction of CO_2_ to CO Exhibiting High Faradaic Efficiency. J. Mater. Chem. A 2016, 4, 15320–15326. 10.1039/C6TA04801C.

[ref361] Zhao S.; Wang Y.; Dong J.; He C.-T.; Yin H.; An P.; Zhao K.; Zhang X.; Gao C.; Zhang L.; et al. Ultrathin Metal-Organic Framework Nanosheets for Electrocatalytic Oxygen Evolution. Nat. Energy 2016, 1, 16184 10.1038/nenergy.2016.184.

[ref362] Zhu W.; Zhang C.; Li Q.; Xiong L.; Chen R.; Wan X.; Wang Z.; Chen W.; Deng Z.; Peng Y. Selective Reduction of CO_2_ by Conductive MOF Nanosheets as an Efficient Co-Catalyst under Visible Light Illumination. Appl. Catal., B 2018, 238, 339–345. 10.1016/j.apcatb.2018.07.024.

[ref363] Zhang S.; Li L.; Zhao S.; Sun Z.; Hong M.; Luo J. Hierarchical Metal-Organic Framework Nanoflowers for Effective CO_2_ Transformation Driven by Visible Light. J. Mater. Chem. A 2015, 3, 15764–15768. 10.1039/C5TA03322E.

[ref364] Huang X.; Holden H. M.; Raushel F. M. Channeling of Substrates and Intermediates in Enzyme-Catalyzed Reactions. Annu. Rev. Biochem. 2001, 70, 149–180. 10.1146/annurev.biochem.70.1.149. 11395405

[ref365] Munro A. W.; McLean K. J. Electron Transfer Cofactors. In Encyclopedia of Biophysics; Roberts G. C. K., Ed.; Springer: Heidelberg, 2013; pp 601–606.

[ref366] Zhang F. Y.; Zhang B. X.; Feng J. Q.; Tan X. N.; Liu L.; Liu L. F.; Han B. X.; Zheng L. R.; Zhang J.; Tai J.; et al. Highly Mesoporous Ru-MIL-125-NH_2_ Produced by Supercritical Fluid for Efficient Photocatalytic Hydrogen Production. ACS Appl. Energy Mater. 2019, 2, 4964–4970. 10.1021/acsaem.9b00649.

[ref367] Zhong H.; Ly K. H.; Wang M.; Krupskaya Y.; Han X.; Zhang J.; Zhang J.; Kataev V.; Büchner B.; Weidinger I. M.; et al. A Phthalocyanine-Based Layered Two-Dimensional Conjugated Metal-Organic Framework as a Highly Efficient Electrocatalyst for the Oxygen Reduction Reaction. Angew. Chem., Int. Ed. 2019, 58, 10677–10682. 10.1002/anie.201907002. 31169942

[ref368] Park J.; Chen Z.; Flores R. A.; Wallnerström G.; Kulkarni A.; Nørskov J. K.; Jaramillo T. F.; Bao Z. Two-Dimensional Conductive Ni-Hab as a Catalyst for the Electrochemical Oxygen Reduction Reaction. ACS Appl. Mater. Interfaces 2020, 12, 39074–39081. 10.1021/acsami.0c09323. 32805928

[ref369] Meng Q.; Yang J.; Ma S.; Zhai M.; Lu J. A Porous Cobalt (II) Metal-Organic Framework with Highly Efficient Electrocatalytic Activity for the Oxygen Evolution Reaction. Polymers 2017, 9, 676 10.3390/polym9120676. 30965980 PMC6418926

[ref370] Nohra B.; El Moll H.; Albelo L. M. R.; Mialane P.; Marrot J.; Mellot-Draznieks C.; O’Keeffe M.; Biboum R. N.; Lemaire J.; Keita B.; et al. Polyoxometalate-Based Metal Organic Frameworks (POMOFs): Structural Trends, Energetics, and High Electrocatalytic Efficiency for Hydrogen Evolution Reaction. J. Am. Chem. Soc. 2011, 133, 13363–13374. 10.1021/ja201165c. 21776992

[ref371] Mukhopadhyay S.; Basu O.; Kar A.; Das S. K. Efficient Electrocatalytic Water Oxidation by Fe(Salen)-MOF Composite: Effect of Modified Microenvironment. Inorg. Chem. 2020, 59, 472–483. 10.1021/acs.inorgchem.9b02745. 31815439

[ref372] Xin Z.; Wang Y.-R.; Chen Y.; Li W.-L.; Dong L.-Z.; Lan Y.-Q. Metallocene Implanted Metalloporphyrin Organic Framework for Highly Selective CO_2_ Electroreduction. Nano Energy 2020, 67, 104233 10.1016/j.nanoen.2019.104233.

[ref373] Miner E. M.; Wang L.; Dincǎ M. Modular O_2_ Electroreduction Activity in Triphenylene-Based Metal-Organic Frameworks. Chem. Sci. 2018, 9, 6286–6291. 10.1039/C8SC02049C. 30123483 PMC6063138

[ref374] Zhao Q.; Jiang J.; Zhao W.; Li S.-H.; Mi W.; Zhang C. Truxone-Based Conductive Metal-Organic Frameworks for the Oxygen Reductive Reaction. J. Phys. Chem. C 2021, 125, 12690–12698. 10.1021/acs.jpcc.1c03418.

[ref375] Wang H.; Zhang X.; Yin F.; Chu W.; Chen B. Coordinately Unsaturated Metal-Organic Framework as an Unpyrolyzed Bifunctional Electrocatalyst for Oxygen Reduction and Evolution Reactions. J. Mater. Chem. A 2020, 8, 22111–22123. 10.1039/D0TA04331A.

[ref376] Wang Y. R.; Huang Q.; He C. T.; Chen Y.; Liu J.; Shen F. C.; Lan Y. Q. Oriented Electron Transmission in Polyoxometalate-Metalloporphyrin Organic Framework for Highly Selective Electroreduction of CO_2_. Nat. Commun. 2018, 9, 4466 10.1038/s41467-018-06938-z. 30367039 PMC6203756

[ref377] Chen X.; Li G. Proton Conductive Zr-Based MOFs. Inorg. Chem. Front. 2020, 7, 3765–3784. 10.1039/D0QI00883D.

[ref378] Lim D.-W.; Kitagawa H. Proton Transport in Metal-Organic Frameworks. Chem. Rev. 2020, 120, 8416–8467. 10.1021/acs.chemrev.9b00842. 32407101

[ref379] Mukhopadhyay S.; Debgupta J.; Singh C.; Sarkar R.; Basu O.; Das S. K. Designing UiO-66-Based Superprotonic Conductor with the Highest Metal-Organic Framework Based Proton Conductivity. ACS Appl. Mater. Interfaces 2019, 11, 13423–13432. 10.1021/acsami.9b01121. 30888148

[ref380] Usov P. M.; Huffman B.; Epley C. C.; Kessinger M. C.; Zhu J.; Maza W. A.; Morris A. J. Study of Electrocatalytic Properties of Metal-Organic Framework PCN-223 for the Oxygen Reduction Reaction. ACS Appl. Mater. Interfaces 2017, 9, 33539–33543. 10.1021/acsami.7b01547. 28353341

[ref381] Zhang M.; Lin Q.; Wu W.; Ye Y.; Yao Z.; Ma X.; Xiang S.; Zhang Z. Isostructural MOFs with Higher Proton Conductivity for Improved Oxygen Evolution Reaction Performance. ACS Appl. Mater. Interfaces 2020, 12, 16367–16375. 10.1021/acsami.9b23356. 32208675

[ref382] Chen J.; Zhang H.; Li B.; Yang J.; Li X.; Zhang T.; He C.; Duan C.; Wang L. Bioinspired Carboxylate-Water Coordination Polymers with Hydrogen-Bond Clusters and Local Coordination Flexibility for Electrochemical Water Splitting. ACS Appl. Energy Mater. 2020, 3, 10515–10524. 10.1021/acsaem.0c01552.

[ref383] Li W.-H.; Lv J.; Li Q.; Xie J.; Ogiwara N.; Huang Y.; Jiang H.; Kitagawa H.; Xu G.; Wang Y. Conductive Metal-Organic Framework Nanowire Arrays for Electrocatalytic Oxygen Evolution. J. Mater. Chem. A 2019, 7, 10431–10438. 10.1039/C9TA02169H.

[ref384] Usov P. M.; Ahrenholtz S. R.; Maza W. A.; Stratakes B.; Epley C. C.; Kessinger M. C.; Zhu J.; Morris A. J. Cooperative Electrochemical Water Oxidation by Zr Nodes and Ni-Porphyrin Linkers of a PCN-224 MOF Thin Film. J. Mater. Chem. A 2016, 4, 16818–16823. 10.1039/C6TA05877A.

[ref385] Xu Q.; Li H.; Yue F.; Chi L.; Wang J. Nanoscale Cobalt Metal-Organic Framework as a Catalyst for Visible Light-Driven and Electrocatalytic Water Oxidation. New J. Chem. 2016, 40, 3032–3035. 10.1039/C5NJ03113C.

[ref386] Luo Y. C.; Chu K. L.; Shi J. Y.; Wu D. J.; Wang X. D.; Mayor M.; Su C. Y. Heterogenization of Photochemical Molecular Devices: Embedding a Metal-Organic Cage into a ZIF-8-Derived Matrix to Promote Proton and Electron Transfer. J. Am. Chem. Soc. 2019, 141, 13057–13065. 10.1021/jacs.9b03981. 31343866

[ref387] Kajiwara T.; Fujii M.; Tsujimoto M.; Kobayashi K.; Higuchi M.; Tanaka K.; Kitagawa S. Photochemical Reduction of Low Concentrations of CO_2_ in a Porous Coordination Polymer with a Ruthenium(II)-CO Complex. Angew. Chem., Int. Ed. 2016, 55, 2697–2700. 10.1002/anie.201508941. 26800222

[ref388] Zhang X. D.; Hou S. Z.; Wu J. X.; Gu Z. Y. Two-Dimensional Metal-Organic Framework Nanosheets with Cobalt-Porphyrins for High-Performance CO_2_ Electroreduction. Chem. Eur. J. 2020, 26, 1604–1611. 10.1002/chem.201904072. 31747078

[ref389] Zhong H.; Ghorbani-Asl M.; Ly K. H.; Zhang J.; Ge J.; Wang M.; Liao Z.; Makarov D.; Zschech E.; Brunner E.; et al. Synergistic Electroreduction of Carbon Dioxide to Carbon Monoxide on Bimetallic Layered Conjugated Metal-Organic Frameworks. Nat. Commun. 2020, 11, 1409 10.1038/s41467-020-15141-y. 32179738 PMC7075876

[ref390] Cheng W.; Zhao X.; Su H.; Tang F.; Che W.; Zhang H.; Liu Q. Lattice-Strained Metal-Organic-Framework Arrays for Bifunctional Oxygen Electrocatalysis. Nat. Energy 2019, 4, 115–122. 10.1038/s41560-018-0308-8.

[ref391] Yang F.; Hu W.; Yang C.; Patrick M.; Cooksy A. L.; Zhang J.; Aguiar J. A.; Fang C.; Zhou Y.; Meng Y. S.; et al. Tuning Internal Strain in Metal-Organic Frameworks via Vapor Phase Infiltration for CO_2_ Reduction. Angew. Chem., Int. Ed. 2020, 59, 4572–4580. 10.1002/anie.202000022. 31914215

[ref392] Zhou T.; Du Y.; Borgna A.; Hong J.; Wang Y.; Han J.; Zhang W.; Xu R. Post-Synthesis Modification of a Metal-Organic Framework to Construct a Bifunctional Photocatalyst for Hydrogen Production. Energy Environ. Sci. 2013, 6, 3229–3234. 10.1039/c3ee41548a.

[ref393] Cichocka M. O.; Liang Z.; Feng D.; Back S.; Siahrostami S.; Wang X.; Samperisi L.; Sun Y.; Xu H.; Hedin N.; et al. A Porphyrinic Zirconium Metal-Organic Framework for Oxygen Reduction Reaction: Tailoring the Spacing between Active-Sites through Chain-Based Inorganic Building Units. J. Am. Chem. Soc. 2020, 142, 15386–15395. 10.1021/jacs.0c06329. 32786758 PMC7498152

[ref394] Ryu U. J.; Kim S. J.; Lim H. K.; Kim H.; Choi K. M.; Kang J. K. Synergistic Interaction of Re Complex and Amine Functionalized Multiple Ligands in Metal-Organic Frameworks for Conversion of Carbon Dioxide. Sci. Rep. 2017, 7, 612 10.1038/s41598-017-00574-1. 28377611 PMC5428566

[ref395] Mao F.; Jin Y.-H.; Liu P. F.; Yang P.; Zhang L.; Chen L.; Cao X.-M.; Gu J.; Yang H. G. Accelerated Proton Transmission in Metal-Organic Frameworks for the Efficient Reduction of CO_2_ in Aqueous Solutions. J. Mater. Chem. A 2019, 7, 23055–23063. 10.1039/C9TA07967J.

[ref396] Sun F.; Chen X. Oxygen Reduction Reaction on Ni_3_(HITP)_2_: A Catalytic Site That Leads to High Activity. Electrochem. Commun. 2017, 82, 89–92. 10.1016/j.elecom.2017.07.028.

[ref397] Li N.; Liu J.; Liu J. J.; Dong L. Z.; Xin Z. F.; Teng Y. L.; Lan Y. Q. Adenine Components in Biomimetic Metal-Organic Frameworks for Efficient CO_2_ Photoconversion. Angew. Chem., Int. Ed. 2019, 58, 5226–5231. 10.1002/anie.201814729. 30656814

[ref398] Zhou Y. X.; Hu W. H.; Yang S. Z.; Zhang Y. B.; Nyakuchena J.; Duisenova K.; Lee S.; Fan D. H.; Huang J. Site-Selective Probes of Mixed-Node Metal Organic Frameworks for Photocatalytic Hydrogen Generation. J. Phys. Chem. C 2020, 124, 1405–1412. 10.1021/acs.jpcc.9b09634.

[ref399] Huang N. Y.; He H.; Li H.; Liao P. Q.; Chen X. M. A Metal-Organic Framework within Situgenerated Low-Coordinate Binuclear Cu(I) Units as a Highly Effective Catalyst for Photodriven Hydrogen Production. Chem. Commun. 2020, 56, 6700–6703. 10.1039/C9CC09589F. 32418996

[ref400] Shi D. Y.; Zheng R.; Sun M. J.; Cao X. R.; Sun C. X.; Cui C. J.; Liu C. S.; Zhao J. W.; Du M. Semiconductive Copper(I)-Organic Frameworks for Efficient Light-Driven Hydrogen Generation without Additional Photosensitizers and Cocatalysts. Angew. Chem., Int. Ed. 2017, 56, 14637–14641. 10.1002/anie.201709869. 28963739

[ref401] Nam D. H.; Bushuyev O. S.; Li J.; De Luna P.; Seifitokaldani A.; Dinh C. T.; Garcia de Arquer F. P.; Wang Y.; Liang Z.; Proppe A. H.; et al. Metal-Organic Frameworks Mediate Cu Coordination for Selective CO_2_ Electroreduction. J. Am. Chem. Soc. 2018, 140, 11378–11386. 10.1021/jacs.8b06407. 30113834

[ref402] Wang Y.; Huang N. Y.; Shen J. Q.; Liao P. Q.; Chen X. M.; Zhang J. P. Hydroxide Ligands Cooperate with Catalytic Centers in Metal-Organic Frameworks for Efficient Photocatalytic CO_2_ Reduction. J. Am. Chem. Soc. 2018, 140, 38–41. 10.1021/jacs.7b10107. 29258308

[ref403] Lian Y.; Yang W.; Zhang C.; Sun H.; Deng Z.; Xu W.; Song L.; Ouyang Z.; Wang Z.; Guo J.; et al. Unpaired 3d Electrons on Atomically Dispersed Cobalt Centres in Coordination Polymers Regulate Both Oxygen Reduction Reaction (ORR) Activity and Selectivity for Use in Zinc-Air Batteries. Angew. Chem., Int. Ed. 2020, 59, 286–294. 10.1002/anie.201910879. 31638312

[ref404] Leger C.; Elliott S. J.; Hoke K. R.; Jeuken L. J.; Jones A. K.; Armstrong F. A. Enzyme Electrokinetics: Using Protein Film Voltammetry to Investigate Redox Enzymes and Their Mechanisms. Biochemistry 2003, 42, 8653–8662. 10.1021/bi034789c. 12873124

[ref405] Hirst J. Elucidating the Mechanisms of Coupled Electron Transfer and Catalytic Reactions by Protein Film Voltammetry. Biochim. Biophys. Acta 2006, 1757, 225–239. 10.1016/j.bbabio.2006.04.002. 16730325

[ref406] Chen H.; Simoska O.; Lim K.; Grattieri M.; Yuan M.; Dong F.; Lee Y. S.; Beaver K.; Weliwatte S.; Gaffney E. M.; et al. Fundamentals, Applications, and Future Directions of Bioelectrocatalysis. Chem. Rev. 2020, 120, 12903–12993. 10.1021/acs.chemrev.0c00472. 33050699

[ref407] Yates N. D. J.; Fascione M. A.; Parkin A. Methodologies for ″Wiring″ Redox Proteins/Enzymes to Electrode Surfaces. Chem. Eur. J. 2018, 24, 12164–12182. 10.1002/chem.201800750. 29637638 PMC6120495

[ref408] Olloqui-Sariego J. L.; Calvente J. J.; Andreu R. Immobilizing Redox Enzymes at Mesoporous and Nanostructured Electrodes. Curr. Opin. Electrochem. 2021, 26, 100658 10.1016/j.coelec.2020.100658.

[ref409] Mazurenko I.; Monsalve K.; Infossi P.; Giudici-Orticoni M.-T.; Topin F.; Mano N.; Lojou E. Impact of Substrate Diffusion and Enzyme Distribution in 3D-Porous Electrodes: A Combined Electrochemical and Modelling Study of a Thermostable H_2_/O_2_ Enzymatic Fuel Cell. Energy Environ. Sci. 2017, 10, 1966–1982. 10.1039/C7EE01830D.

[ref410] Mijangos E.; Roy S.; Pullen S.; Lomoth R.; Ott S. Evaluation of Two- and Three-Dimensional Electrode Platforms for the Electrochemical Characterization of Organometallic Catalysts Incorporated in Non-Conducting Metal-Organic Frameworks. Dalton Trans. 2017, 46, 4907–4911. 10.1039/C7DT00578D. 28345708 PMC6086323

[ref411] Huang Z. H.; Xie N. H.; Zhang M.; Xu B. Q. Nonpyrolyzed Fe-N Coordination-Based Iron Triazolate Framework: An Efficient and Stable Electrocatalyst for Oxygen Reduction Reaction. ChemSusChem 2019, 12, 200–207. 10.1002/cssc.201801886. 30339329

[ref412] Dong B.-X.; Qian S.-L.; Bu F.-Y.; Wu Y.-C.; Feng L.-G.; Teng Y.-L.; Liu W.-L.; Li Z.-W. Electrochemical Reduction of CO_2_ to CO by a Heterogeneous Catalyst of Fe-Porphyrin-Based Metal-Organic Framework. ACS Appl. Energy Mater. 2018, 1, 4662–4669. 10.1021/acsaem.8b00797.

[ref413] Fang Y.; Li X.; Li F.; Lin X.; Tian M.; Long X.; An X.; Fu Y.; Jin J.; Ma J. Self-Assembly of Cobalt-Centered Metal Organic Framework and Multiwalled Carbon Nanotubes Hybrids as a Highly Active and Corrosion-Resistant Bifunctional Oxygen Catalyst. J. Power Sources 2016, 326, 50–59. 10.1016/j.jpowsour.2016.06.114.

[ref414] Micheroni D.; Lan G. X.; Lin W. B. Efficient Electrocatalytic Proton Reduction with Carbon Nanotube-Supported Metal-Organic Frameworks. J. Am. Chem. Soc. 2018, 140, 15591–15595. 10.1021/jacs.8b09521. 30392362

[ref415] Ma W.; Wu F.; Yu P.; Mao L. Carbon Support Tuned Electrocatalytic Activity of a Single-Site Metal-Organic Framework toward the Oxygen Reduction Reaction. Chem. Sci. 2021, 12, 7908–7917. 10.1039/D1SC00997D. 34168844 PMC8188507

[ref416] Jiang M.; Li L.; Zhu D.; Zhang H.; Zhao X. Oxygen Reduction in the Nanocage of Metal-Organic Frameworks with an Electron Transfer Mediator. J. Mater. Chem. A 2014, 2, 5323–5329. 10.1039/C3TA15319C.

[ref417] Jahan M.; Bao Q.; Loh K. P. Electrocatalytically Active Graphene-Porphyrin MOF Composite for Oxygen Reduction Reaction. J. Am. Chem. Soc. 2012, 134, 6707–6713. 10.1021/ja211433h. 22439970

[ref418] Jahan M.; Liu Z. L.; Loh K. P. A Graphene Oxide and Copper-Centered Metal Organic Framework Composite as a Tri-Functional Catalyst for HER, OER, and ORR. Adv. Funct. Mater. 2013, 23, 5363–5372. 10.1002/adfm.201300510.

[ref419] Sohrabi S.; Dehghanpour S.; Ghalkhani M. Three-Dimensional Metal-Organic Framework Graphene Nanocomposite as a Highly Efficient and Stable Electrocatalyst for the Oxygen Reduction Reaction in Acidic Media. ChemCatChem. 2016, 8, 2356–2366. 10.1002/cctc.201600298.

[ref420] Kang X.; Zhu Q.; Sun X.; Hu J.; Zhang J.; Liu Z.; Han B. Highly Efficient Electrochemical Reduction of CO_2_ to CH_4_ in an Ionic Liquid Using a Metal-Organic Framework Cathode. Chem. Sci. 2016, 7, 266–273. 10.1039/C5SC03291A. 29861981 PMC5952524

[ref421] Hao Y. C.; Chen L. W.; Li J.; Guo Y.; Su X.; Shu M.; Zhang Q.; Gao W. Y.; Li S.; Yu Z. L.; et al. Metal-Organic Framework Membranes with Single-Atomic Centers for Photocatalytic CO_2_ and O_2_ Reduction. Nat. Commun. 2021, 12, 2682 10.1038/s41467-021-22991-7. 33976220 PMC8113524

[ref422] Wang Y.; Suzuki H.; Xie J.; Tomita O.; Martin D. J.; Higashi M.; Kong D.; Abe R.; Tang J. Mimicking Natural Photosynthesis: Solar to Renewable H_2_ Fuel Synthesis by Z-Scheme Water Splitting Systems. Chem. Rev. 2018, 118, 5201–5241. 10.1021/acs.chemrev.7b00286. 29676566 PMC5968435

[ref423] Nishida J.; Tamimi A.; Fei H.; Pullen S.; Ott S.; Cohen S. M.; Fayer M. D. Structural Dynamics inside a Functionalized Metal-Organic Framework Probed by Ultrafast 2D IR Spectroscopy. Proc. Natl. Acad. Sci. U. S. A. 2014, 111, 18442–18447. 10.1073/pnas.1422194112. 25512539 PMC4284562

[ref424] Zhao P.; Fang H.; Mukhopadhyay S.; Li A.; Rudic S.; McPherson I. J.; Tang C. C.; Fairen-Jimenez D.; Tsang S. C. E.; Redfern S. A. T. Structural Dynamics of a Metal-Organic Framework Induced by CO_2_ Migration in Its Non-Uniform Porous Structure. Nat. Commun. 2019, 10, 999 10.1038/s41467-019-08939-y. 30824710 PMC6397191

[ref425] Lan G.; Fan Y.; Shi W.; You E.; Veroneau S. S.; Lin W. Biomimetic Active Sites on Monolayered Metal-Organic Frameworks for Artificial Photosynthesis. Nat. Catal. 2022, 5, 1006–1018. 10.1038/s41929-022-00865-5.

